# Supplementary material for: Study to evaluate the readability and visual appearance of online resources for blunt chest trauma: an evaluation of online resources using mixed methods
Source: BMJ Open. 2024 Feb 6;14(2):e078552. doi: 10.1136/bmjopen-2023-078552 (PMC10860042; doi:10.1136/bmjopen-2023-078552)
Supplement: Supplementary data [file bmjopen-2023-078552supp001.pdf]

BMJ Publishing Group Limited (BMJ) disclaims all liability and responsibility arising from any reliance placed on this supplemental material which has been supplied by the author(s)

Supplementary information. Table S1. Visual assessment of each website based on the Centers for Medicare and Medicaid Services Toolkit for Making Written Material Clear and Effective[22].

	**Website**																					
**Scoring Criteria**	**1**	**2**	**3**	**4**	**5**	**6**	**7**	**8**	**9**	**10**	**11**	**12**	**13**	**14**	**15**	**16**	**17**	**18**	**19**	**20**	**21**	**22**
Overall design and page layout	1	-1	1	1	-1	1	1	1	1	-1	1	-1	-1	1	1	1	1	1	-1	-1	1	1
The size, shape and general look of the material was designed with its purpose and users in mind	1	-1	1	1	1	1	-1	1	1	-1	1	-1	-1	1	1	1	1	1	1	-1	1	1
The material looks appealing at first glance	1	-1	1	1	-1	1	1	1	1	1	1	-1	-1	1	1	1	1	1	-1	1	1	1
A clear and obvious path has been created for the eye to follow	1	1	1	1	1	1	-1	1	1	-1	1	1	1	1	1	1	1	1	1	1	1	1
The material that has a clear and consistent style and structure	1	1	1	1	1	1	1	1	1	1	1	1	1	1	1	1	1	1	1	1	1	1
Fonts, size of print and contrast	1	1	1	1	1	1	1	1	1	1	1	1	-1	1	1	1	1	1	1	1	1	1
For the regular text, a font that is designed for ease of reading is used	1	1	1	1	1	1	1	1	1	1	1	1	-1	1	1	1	1	1	1	1	1	1
For headings, an easy-to-read font is used that contrasts with the main text	1	1	1	1	1	1	1	1	1	1	1	1	1	1	1	1	1	1	1	1	1	1
In general, no more than 2 or 3 different typefaces are used	1	1	1	-1	1	1	1	1	1	-1	1	1	1	1	1	1	-1	1	1	1	1	1
The font size is large enough for the intended audience	1	1	1	1	1	1	-1	1	1	-1	1	1	-1	1	1	1	1	1	1	1	1	1
Upper and lower case are used, not all capitals	1	1	1	1	1	1	1	1	1	1	1	-1	1	1	1	1	1	1	1	1	1	1
To emphasize words and phrases italics or bold text are used	1	1	1	-1	1	1	1	1	1	1	1	1	1	1	1	1	1	1	1	1	1	1
For ease of reading, dark-coloured text is used on a very light background	1	1	1	1	1	1	1	1	1	1	1	1	1	1	1	1	1	1	1	1	1	1
Text is not aligned sideways, on patterned or shaded background or on top of photos or other images	1	1	1	1	1	1	1	1	1	1	1	1	1	1	1	1	1	1	1	1	1	1
For ease of reading, extra line spacing has been added	1	1	1	1	1	1	1	1	1	-1	1	1	-1	1	1	1	-1	1	1	1	1	1
For ease of reading, left justification is used throughout	1	1	1	1	1	1	1	1	1	1	1	1	-1	1	1	1	-1	1	1	1	1	1
Lines of text are an appropriate length—neither too long nor too short	-1	-1	1	-1	1	1	1	1	1	-1	-1	-1	-1	1	1	1	1	-1	1	1	1	1
Hyphenation has been avoided at the end of lines	1	1	1	1	1	1	1	1	1	1	1	1	-1	1	1	1	1	1	1	1	1	1
Headings, bulleted lists, and blocks of text	1	1	1	1	1	1	1	1	1	1	-1	1	1	1	1	1	1	1	1	1	1	1
There is a clear hierarchy of prominent headings and sub-headings	1	1	1	1	1	1	1	1	1	1	1	1	1	1	1	1	-1	1	1	-1	1	1
Contrast is used to make the main points stand out	1	1	1	1	1	1	1	1	1	1	1	1	-1	1	1	1	1	1	1	1	1	1
Bulleted lists are well formatted	0	1	1	1	-1	1	1	1	0	1	0	1	0	1	1	1	1	1	1	1	1	1
Effective ways are used to emphasize important blocks of text	0	-1	1	-1	-1	1	1	1	1	1	1	1	1	1	1	1	-1	1	1	1	1	1
Use of colour	0	-1	1	1	-1	-1	-1	-1	1	1	-1	-1	-1	1	-1	-1	-1	1	-1	1	1	1
Colours used are appealing to the intended readers	0	-1	1	-1	-1	-1	-1	-1	1	-1	1	-1	-1	1	-1	-1	-1	-1	-1	-1	1	1
Colour is used sparingly and in a consistent and deliberate way	1	1	1	-1	-1	1	-1	-1	1	1	1	-1	-1	1	1	1	-1	1	1	1	1	1

**Scoring Criteria**	**1**	**2**	**3**	**4**	**5**	**6**	**7**	**8**	**9**	**10**	**11**	**12**	**13**	**14**	**15**	**16**	**17**	**18**	**19**	**20**	**21**	**22**
The colour scheme works from a design standpoint and when printed	1	1	1	-1	-1	1	-1	1	1	-1	1	1	-1	1	1	1	-1	1	1	-1	1	1
The colour scheme works for with diminished or limited colour perception	0	1	1	-1	-1	1	-1	1	1	-1	1	1	-1	1	1	1	-1	1	1	-1	1	1
Photographs and illustrations	-1	-1	-1	-1	1	-1	1	-1	1	1	-1	-1	1	-1	-1	-1	1	-1	-1	-1	-1	1
Photos and illustrations are used that relate directly to the information to reinforce key messages	0	0	0	-1	-1	0	-1	0	-1	-1	0	0	1	0	-1	0	1	0	0	-1	0	1
Images used are clear, uncluttered, and consistent in style	0	0	0	-1	-1	0	-1	0	-1	-1	-1	0	1	0	0	0	1	0	0	-1	0	1
Photos and illustrations used are culturally appropriate for the intended readers	0	0	0	-1	-1	0	1	0	0	-1	-1	0	1	0	0	0	1	0	0	-1	0	1
When images include people, they are appropriate to the situation and intended audience	0	0	0	0	0	0	0	0	0	0	0	0	-1	0	0	0	1	0	0	-1	0	1
Tables, charts, and diagrams	-1	-1	-1	1	-1	-1	1	-1	-1	-1	-1	-1	-1	-1	-1	-1	-1	-1	-1	-1	-1	1
Likely literacy levels of the reader have been considered in the use of tables, charts, and diagrams	-1	0	0	-1	0	0	0	0	1	0	-1	0	0	0	0	0	0	0	0	0	0	1
Titles, headings, and other labelling is specific and clear	1	1	0	-1	0	0	-1	1	1	1	1	1	1	1	1	1	-1	1	1	1	1	1
A clean, uncluttered layout is used with strong visual cures to guide the reader through the information	1	-1	1	1	-1	1	-1	1	-1	-1	1	-1	-1	1	1	1	-1	-1	1	-1	1	1
Numbers or calculations are carefully explained	0	0	0	0	0	0	0	0	0	0	0	0	0	0	0	0	0	0	0	0	0	0
General information about website	1	-1	1	1	-1	1	1	1	1	1	1	1	1	1	-1	1	1	1	1	1	-1	1
Last updated date given	1	1	1	-1	1	1	1	1	1	1	1	1	1	1	1	1	1	1	1	1	-1	1
Frequency of updates given	-1	-1	-1	-1	-1	1	-1	-1	-1	-1	-1	-1	-1	-1	-1	-1	-1	-1	-1	-1	-1	-1
Relevant references given	1	1	-1	1	1	-1	-1	-1	-1	-1	1	-1	-1	-1	-1	-1	-1	-1	-1	-1	-1	-1
**Overall assessment score**	22	12	27	8	6	25	11	22	26	5	20	10	-3	28	21	24	10	22	20	10	24	37

(+1 point if the statement was achieved, 0 if the statement was not applicable and -1 point if the statement was not achieved.)

	Website																					
**Scoring Criteria**	**23**	**24**	**25**	**26**	**27**	**28**	**29**	**30**	**31**	**32**	**33**	**34**	**35**	**36**	**37**	**38**	**39**	**40**	**41**	**42**	**43**	**44**
Overall design and page layout	1	1	1	1	1	-1	1	-1	1	-1	-1	1	1	-1	1	1	1	1	1	1	1	1
The size, shape and general look of the material was designed with its purpose and users in mind	1	1	1	1	1	1	1	-1	1	1	-1	1	1	-1	1	1	-1	1	1	1	1	1
The material looks appealing at first glance	1	1	1	1	1	-1	1	-1	1	1	-1	1	1	-1	1	1	1	1	-1	-1	-1	1
A clear and obvious path has been created for the eye to follow	1	1	-1	1	-1	-1	1	-1	1	1	1	1	1	1	1	1	1	1	-1	-1	-1	1
The material that has a clear and consistent style and structure	1	1	1	1	1	1	1	1	1	1	1	1	1	1	1	1	-1	1	1	1	1	1
Fonts, size of print and contrast	1	1	1	1	1	1	1	1	1	1	1	1	1	1	1	1	1	1	1	1	1	1
For the regular text, a font that is designed for ease of reading is used	1	1	1	1	1	1	1	1	1	1	1	1	1	1	1	1	1	1	1	1	1	1
For headings, an easy-to-read font is used that contrasts with the main text	1	1	1	1	1	1	1	1	1	1	1	1	1	1	1	1	1	1	1	1	1	1
In general, no more than 2 or 3 different typefaces are used	1	1	1	1	1	1	1	1	1	1	1	1	1	1	1	1	1	1	1	1	1	1
The font size is large enough for the intended audience	1	1	1	1	1	1	1	1	1	1	1	1	1	1	1	1	1	1	1	1	1	1
Upper and lower case are used, not all capitals	1	1	1	1	1	1	1	1	1	1	1	1	1	1	1	1	1	1	1	1	1	1
To emphasize words and phrases italics or bold text are used	1	1	1	1	1	1	1	1	1	1	1	1	1	1	1	1	1	1	1	1	1	1
For ease of reading, dark-coloured text is used on a very light background	1	1	1	1	1	1	1	1	1	1	-1	1	1	1	1	1	1	1	1	1	1	1
Text is not aligned sideways, on patterned or shaded background or on top of photos or other images	1	1	1	1	1	1	1	1	1	1	-1	1	1	1	1	1	1	1	1	1	1	1
For ease of reading, extra line spacing has been added	1	1	1	1	-1	1	1	1	1	1	-1	1	1	1	1	1	1	1	1	1	1	1
For ease of reading, left justification is used throughout	1	1	1	1	1	1	1	1	1	1	1	1	1	1	1	1	1	1	1	1	1	1
Lines of text are an appropriate length—neither too long nor too short	1	1	1	1	-1	1	1	1	-1	-1	-1	1	-1	1	1	1	-1	1	1	-1	-1	-1
Hyphenation has been avoided at the end of lines	1	1	1	1	1	1	1	1	1	1	1	1	1	1	1	1	1	1	1	1	1	1
Headings, bulleted lists, and blocks of text	1	1	1	1	1	1	1	1	1	1	1	1	1	1	1	1	1	1	1	1	1	1
There is a clear hierarchy of prominent headings and sub-headings	1	1	1	1	1	1	1	1	1	1	1	1	1	1	1	1	1	1	1	1	1	1
Contrast is used to make the main points stand out	1	1	1	1	1	1	-1	1	1	1	1	1	1	1	1	1	1	1	1	1	1	1
Bulleted lists are well formatted	1	1	1	1	0	1	1	1	1	1	1	1	1	1	1	1	1	1	1	1	1	1
Effective ways are used to emphasize important blocks of text	1	1	1	1	1	1	1	1	1	1	1	1	1	1	1	1	1	1	1	1	1	1
Use of colour	1	1	1	1	1	1	1	1	1	1	-1	1	-1	1	1	1	1	1	1	1	1	1
Colours used are appealing to the intended readers	1	1	1	1	-1	1	1	1	1	1	-1	1	1	1	1	1	1	1	1	1	1	1
Colour is used sparingly and in a consistent and deliberate way	1	1	1	1	-1	1	1	1	1	1	-1	1	1	1	1	1	1	1	1	1	1	-1
The colour scheme works from a design standpoint and when printed	-1	1	-1	1	-1	1	1	-1	1	1	-1	1	-1	1	1	1	1	1	1	1	1	-1

**Scoring Criteria**	**23**	**24**	**25**	**26**	**27**	**28**	**29**	**30**	**31**	**32**	**33**	**34**	**35**	**36**	**37**	**38**	**39**	**40**	**41**	**42**	**43**	**44**
The colour scheme works for with diminished or limited colour perception	1	1	-1	1	-1	1	1	-1	1	1	-1	1	-1	1	1	1	1	1	1	1	1	-1
Photographs and illustrations	1	-1	-1	-1	1	-1	1	-1	-1	1	-1	-1	1	-1	-1	-1	1	-1	-1	-1	1	1
Photos and illustrations are used that relate directly to the information to reinforce key messages	0	0	0	0	1	0	1	0	0	1	0	0	1	-1	0	0	-1	0	-1	-1	-1	-1
Images used are clear, uncluttered, and consistent in style	0	0	-1	0	1	0	1	0	0	1	0	0	1	-1	0	0	1	0	1	0	-1	-1
Photos and illustrations used are culturally appropriate for the intended readers	0	0	0	0	-1	0	1	0	0	1	0	0	1	-1	0	0	-1	0	-1	0	1	1
When images include people, they are appropriate to the situation and intended audience	0	0	0	0	0	0	0	0	0	0	0	0	1	-1	0	0	-1	0	1	0	1	0
Tables, charts, and diagrams	-1	-1	-1	-1	1	-1	-1	-1	-1	1	-1	-1	-1	-1	-1	-1	1	-1	-1	-1	-1	1
Likely literacy levels of the reader have been considered in the use of tables, charts, and diagrams	0	0	0	0	-1	0	1	0	0	1	-1	0	1	0	0	0	-1	0	0	0	0	-1
Titles, headings, and other labelling is specific and clear	1	1	1	1	1	1	1	1	1	1	1	1	1	1	1	0	1	1	1	1	1	1
A clean, uncluttered layout is used with strong visual cures to guide the reader through the information	1	1	-1	1	-1	-1	1	-1	1	-1	-1	1	1	-1	1	1	1	1	-1	1	-1	-1
Numbers or calculations are carefully explained	0	0	0	0	0	0	0	0	0	0	0	0	0	0	0	0	0	0	0	0	0	0
General information about website	1	1	-1	-1	-1	1	1	1	-1	1	-1	-1	1	1	1	-1	-1	1	-1	-1	1	-1
Last updated date given	1	1	-1	-1	-1	1	1	1	-1	1	-1	-1	1	1	1	-1	-1	1	-1	-1	1	1
Frequency of updates given	0	1	-1	-1	-1	-1	-1	-1	-1	-1	-1	-1	-1	-1	1	-1	-1	1	-1	-1	-1	-1
Relevant references given	-1	-1	-1	-1	-1	-1	-1	-1	-1	1	-1	-1	-1	-1	-1	-1	-1	-1	-1	1	-1	-1
**Overall assessment score**	29	30	15	24	11	20	32	14	22	32	-3	24	27	16	30	23	19	30	18	19	22	18

(+1 point if the statement was achieved, 0 if the statement was not applicable and -1 point if the statement was not achieved.)

	**Website**																					
**Scoring Criteria**	**45**	**46**	**47**	**48**	**49**	**50**	**51**	**52**	**53**	**54**	**55**	**56**	**57**	**58**	**59**	**60**	**61**	**62**	**63**	**64**	**65**	**66**
Overall design and page layout	1	1	1	1	1	1	1	1	1	1	1	-1	1	-1	-1	1	-1	-1	1	1	1	1
The size, shape and general look of the material was designed with its purpose and users in mind	1	1	1	1	1	1	1	1	1	1	1	1	1	-1	-1	1	1	-1	1	1	1	1
The material looks appealing at first glance	1	1	-1	-1	1	1	1	1	1	1	1	1	1	-1	-1	-1	-1	-1	1	1	1	-1
A clear and obvious path has been created for the eye to follow	1	1	1	1	1	1	1	1	1	1	1	-1	1	-1	-1	-1	-1	-1	1	1	1	-1
The material that has a clear and consistent style and structure	1	1	1	1	1	1	1	1	1	1	1	1	1	1	1	1	1	1	1	1	1	1
Fonts, size of print and contrast	1	1	1	1	1	1	1	1	1	1	1	1	1	1	1	1	1	1	1	1	-1	1
For the regular text, a font that is designed for ease of reading is used	1	1	1	1	1	1	1	1	1	1	1	1	1	1	1	1	1	1	1	1	1	1
For headings, an easy-to-read font is used that contrasts with the main text	1	1	1	1	1	1	1	1	1	1	1	1	1	1	1	1	1	1	1	1	1	1
In general, no more than 2 or 3 different typefaces are used	1	1	1	1	1	1	1	1	1	1	1	1	1	1	1	1	1	1	1	1	1	1
The font size is large enough for the intended audience	1	1	1	1	1	1	1	1	1	1	1	1	1	1	1	1	1	1	1	1	1	1
Upper and lower case are used, not all capitals	1	1	1	1	1	1	1	1	1	1	1	1	1	1	1	1	1	1	1	1	1	1
To emphasize words and phrases italics or bold text are used	1	1	1	1	1	1	1	1	1	1	1	1	1	1	1	1	1	1	1	1	-1	1
For ease of reading, dark-coloured text is used on a very light background	1	1	1	1	1	1	1	1	1	1	1	-1	1	1	1	1	1	1	1	1	-1	1
Text is not aligned sideways, on patterned or shaded background or on top of photos or other images	1	1	1	1	1	1	1	1	1	1	1	1	1	1	1	1	1	1	1	1	1	1
For ease of reading, extra line spacing has been added	1	1	1	1	1	1	1	1	1	1	1	1	1	1	1	1	1	1	1	1	1	1
For ease of reading, left justification is used throughout	1	1	1	1	1	1	1	1	1	1	1	1	1	1	1	1	1	1	1	1	1	1
Lines of text are an appropriate length—neither too long nor too short	-1	1	1	1	1	1	1	1	1	1	-1	-1	1	-1	-1	1	-1	-1	1	1	-1	1
Hyphenation has been avoided at the end of lines	1	1	1	1	1	1	1	1	1	1	1	1	1	1	1	1	1	1	1	1	1	1
Headings, bulleted lists, and blocks of text	1	1	1	1	1	1	1	1	1	1	1	1	1	1	1	1	1	1	1	1	1	1
There is a clear hierarchy of prominent headings and sub-headings	1	1	1	1	1	1	1	1	1	1	1	1	1	1	1	1	1	1	1	1	1	1
Contrast is used to make the main points stand out	1	1	1	1	1	1	1	1	1	1	1	1	1	1	1	1	1	1	1	1	-1	1
Bulleted lists are well formatted	1	1	1	1	1	1	1	1	1	1	1	1	1	1	1	1	1	-1	1	1	1	1
Effective ways are used to emphasize important blocks of text	1	1	1	1	1	1	1	1	1	1	1	1	1	1	1	1	1	1	1	1	-1	1
Use of colour	1	1	-1	-1	-1	1	1	1	1	1	1	1	1	1	1	1	1	1	1	1	1	1
Colours used are appealing to the intended readers	1	1	-1	-1	1	1	1	1	1	1	1	-1	1	1	1	1	1	1	1	1	1	1
Colour is used sparingly and in a consistent and deliberate way	1	1	1	1	1	1	1	1	1	1	1	-1	1	1	1	1	1	1	1	1	1	1
The colour scheme works from a design standpoint and when printed	1	1	1	1	1	1	1	1	1	1	1	-1	1	1	1	1	1	1	1	1	-1	1
The colour scheme works for with diminished or limited colour perception	1	1	1	1	1	1	1	1	1	1	1	-1	1	1	1	1	1	1	1	1	-1	1

**Scoring Criteria**	**45**	**46**	**47**	**48**	**49**	**50**	**51**	**52**	**53**	**54**	**55**	**56**	**57**	**58**	**59**	**60**	**61**	**62**	**63**	**64**	**65**	**66**
Photographs and illustrations	1	1	-1	-1	-1	-1	-1	1	-1	-1	-1	1	1	1	-1	1	1	-1	1	-1	-1	1
Photos and illustrations are used that relate directly to the information to reinforce key messages	-1	-1	0	0	0	0	0	-1	0	0	0	0	-1	-1	-1	-1	-1	0	1	0	0	1
Images used are clear, uncluttered, and consistent in style	1	1	0	0	0	0	0	1	0	0	0	0	1	0	-1	0	0	0	1	0	0	1
Photos and illustrations used are culturally appropriate for the intended readers	1	0	0	0	0	0	0	1	0	0	0	1	1	0	0	0	1	0	1	0	0	1
When images include people, they are appropriate to the situation and intended audience	-1	-1	0	0	0	0	0	1	0	0	0	1	1	-1	0	-1	0	0	1	0	0	-1
Tables, charts, and diagrams	-1	-1	-1	-1	-1	-1	-1	-1	-1	-1	-1	-1	-1	-1	-1	-1	-1	-1	-1	-1	-1	-1
Likely literacy levels of the reader have been considered in the use of tables, charts, and diagrams	0	0	0	0	0	0	0	0	0	0	0	0	0	0	0	0	0	0	1	0	0	0
Titles, headings, and other labelling is specific and clear	1	1	1	1	1	1	1	1	1	1	1	1	1	1	1	1	1	1	1	1	1	1
A clean, uncluttered layout is used with strong visual cures to guide the reader through the information	1	1	1	1	1	1	1	1	1	-1	1	-1	1	-1	-1	1	-1	-1	1	1	1	1
Numbers or calculations are carefully explained	0	0	0	0	0	0	0	0	0	0	0	0	0	0	0	0	0	0	0	0	0	0
General information about website	-1	-1	1	1	1	1	1	1	-1	1	1	-1	-1	-1	1	-1	1	-1	1	1	-1	1
Last updated date given	-1	-1	1	1	1	1	1	1	-1	1	1	-1	-1	-1	-1	-1	-1	-1	1	1	-1	1
Frequency of updates given	-1	-1	-1	-1	1	1	1	1	-1	1	-1	-1	-1	-1	-1	-1	-1	-1	-1	1	-1	-1
Relevant references given	-1	-1	-1	-1	-1	-1	-1	-1	-1	-1	1	-1	-1	-1	-1	-1	-1	-1	-1	-1	-1	-1
**Overall assessment score**	24	25	22	22	28	30	30	34	24	28	28	10	28	12	12	20	18	10	35	30	8	28

(+1 point if the statement was achieved, 0 if the statement was not applicable and -1 point if the statement was not achieved.)

	Website																		
**Scoring Criteria**	**67**	**68**	**69**	**70**	**71**	**72**	**73**	**74**	**75**	**76**	**77**	**78**	**79**	**80**	**81**	**82**	**83**	**84**	**85**
Overall design and page layout	-1	1	1	1	-1	1	1	1	1	1	-1	1	-1	1	1	1	-1	-1	1
The size, shape and general look of the material was designed with its purpose and users in mind	1	1	1	1	1	1	1	1	1	1	-1	1	1	1	1	1	1	-1	1
The material looks appealing at first glance	-1	1	1	-1	-1	1	1	-1	-1	-1	-1	1	-1	1	1	1	1	-1	1
A clear and obvious path has been created for the eye to follow	-1	1	1	1	1	1	1	1	1	1	-1	1	1	1	-1	1	1	-1	1
The material that has a clear and consistent style and structure	1	1	1	1	1	1	1	1	1	1	-1	1	1	1	-1	1	1	-1	1
Fonts, size of print and contrast	1	1	1	1	1	1	-1	-1	1	1	-1	1	1	1	1	1	1	1	1
For the regular text, a font that is designed for ease of reading is used	1	1	1	1	1	1	-1	1	1	1	-1	1	1	1	1	1	1	1	1
For headings, an easy-to-read font is used that contrasts with the main text	1	1	1	1	1	1	1	-1	1	1	1	1	1	1	1	1	1	1	1
In general, no more than 2 or 3 different typefaces are used	1	1	1	1	1	1	1	1	1	1	1	1	1	1	1	1	1	1	1
The font size is large enough for the intended audience	1	1	1	1	1	1	1	-1	1	1	-1	1	1	1	1	1	1	1	1
Upper and lower case are used, not all capitals	1	1	1	1	1	1	1	1	1	1	1	1	1	1	1	1	1	-1	1
To emphasize words and phrases italics or bold text are used	-1	1	1	1	-1	1	1	1	1	1	1	1	1	1	1	1	1	1	1
For ease of reading, dark-coloured text is used on a very light background	-1	1	1	1	1	1	1	1	1	1	-1	1	1	1	1	1	1	1	1
Text is not aligned sideways, on patterned or shaded background or on top of photos or other images	1	1	1	1	1	1	1	1	1	1	1	1	1	1	1	1	1	1	1
For ease of reading, extra line spacing has been added	1	1	1	1	1	1	1	1	1	1	-1	1	1	1	1	1	1	1	1
For ease of reading, left justification is used throughout	1	1	1	1	1	1	1	1	1	1	1	1	1	1	1	1	1	-1	1
Lines of text are an appropriate length—neither too long nor too short	-1	1	1	-1	-1	1	-1	1	1	1	-1	1	-1	1	-1	-1	1	1	1
Hyphenation has been avoided at the end of lines	1	1	1	1	1	1	1	1	1	1	1	1	1	1	1	1	1	1	1
Headings, bulleted lists, and blocks of text	1	1	1	1	-1	1	1	1	1	1	1	1	1	1	1	1	1	1	1
There is a clear hierarchy of prominent headings and sub-headings	1	1	1	1	-1	1	1	1	1	1	-1	1	1	1	1	1	1	-1	1
Contrast is used to make the main points stand out	1	1	1	1	-1	1	1	1	1	1	1	1	1	1	1	1	1	1	1
Bulleted lists are well formatted	1	1	1	1	-1	1	1	1	1	1	1	1	1	1	1	1	1	1	1
Effective ways are used to emphasize important blocks of text	1	1	1	1	-1	1	1	1	1	1	-1	1	1	1	1	1	1	1	1
Use of colour	1	1	1	1	-1	1	1	1	1	1	-1	1	1	1	1	1	1	1	1
Colours used are appealing to the intended readers	-1	1	1	1	-1	1	1	1	1	-1	-1	1	1	1	1	1	1	1	1
Colour is used sparingly and in a consistent and deliberate way	1	1	1	1	1	1	1	1	1	-1	-1	1	1	1	1	1	1	1	1
The colour scheme works from a design standpoint and when printed	-1	1	1	1	1	1	1	1	1	1	-1	1	1	1	1	1	1	1	1
The colour scheme works for with diminished or limited colour perception	-1	1	1	1	1	1	-1	1	1	-1	-1	1	1	1	1	1	1	1	1

**Scoring Criteria**	**67**	**68**	**69**	**70**	**71**	**72**	**73**	**74**	**75**	**76**	**77**	**78**	**79**	**80**	**81**	**82**	**83**	**84**	**85**
Photographs and illustrations	1	-1	1	1	-1	1	-1	-1	-1	1	-1	-1	1	-1	-1	-1	-1	1	1
Photos and illustrations are used that relate directly to the information to reinforce key messages	-1	0	1	1	0	-1	0	0	0	1	0	0	1	0	0	0	0	-1	1
Images used are clear, uncluttered, and consistent in style	1	0	1	-1	0	1	0	0	0	1	0	0	1	0	0	0	0	-1	1
Photos and illustrations used are culturally appropriate for the intended readers	1	0	1	1	0	0	0	0	0	1	0	0	1	0	0	0	0	1	1
When images include people, they are appropriate to the situation and intended audience	0	0	0	1	0	0	0	0	0	1	0	0	1	0	0	0	0	1	1
Tables, charts, and diagrams	-1	-1	-1	1	-1	-1	-1	-1	-1	1	-1	-1	1	-1	-1	-1	-1	-1	-1
Likely literacy levels of the reader have been considered in the use of tables, charts, and diagrams	0	0	1	-1	0	-1	0	0	0	1	0	1	1	0	0	0	0	0	0
Titles, headings, and other labelling is specific and clear	1	1	1	1	1	1	1	1	1	1	1	1	1	1	1	1	0	1	1
A clean, uncluttered layout is used with strong visual cures to guide the reader through the information	-1	1	1	1	1	1	1	1	1	1	-1	1	-1	1	-1	1	1	-1	1
Numbers or calculations are carefully explained	0	0	0	0	0	0	0	0	0	0	0	0	0	0	0	0	0	0	0
General information about website	-1	1	1	1	1	-1	-1	-1	-1	-1	-1	1	-1	1	-1	-1	-1	-1	1
Last updated date given	-1	1	1	1	1	-1	-1	-1	-1	-1	-1	1	-1	1	-1	-1	-1	-1	1
Frequency of updates given	-1	1	1	-1	-1	-1	-1	-1	-1	-1	-1	1	-1	1	-1	-1	-1	-1	-1
Relevant references given	-1	1	-1	1	-1	1	-1	-1	-1	-1	-1	-1	-1	-1	-1	-1	1	-1	-1
**Overall assessment score**	7	32	36	31	6	27	16	16	22	25	-14	31	25	30	16	22	23	8	34

(+1 point if the statement was achieved, 0 if the statement was not applicable and -1 point if the statement was not achieved.)

Supplementary information. Table S2. Narrative overview assessment of the 85 identified websites.

Name of Website	Website address	Website assessment
1. Medscape	https://emedicine.medscape.com/article/428723-overview?form=fpf	The Medscape website is clearly laid out, with bold headings, sub-headings and regular use of paragraphs. This makes it aesthetically appealing, with clear links to move between different sections of the article. However, there is heavy use of medical terminology and information that is not relevant or useful to the public as it details complex anatomical and physiological structures. There is no use of diagrams, only an image of an x-ray. The website does include information on causes, symptoms and treatments of BCT. This website appears more focused on use by clinicians than by the general public.
2. UpToDate	https://www.uptodate.com/contents/initial-evaluation-and-management-of-blunt-thoracic-trauma-in-adults	The UpToDate website page contains very informative content regarding BCT. There are links to further information on specific injuries, however this website focuses more on the anatomical nature of these injuries rather than symptoms and how to diagnose them. In general, this website seems to focus on information relevant to a clinician rather than a member of the public, with detailed descriptions of medical techniques. The layout is poor, as the text area only takes up around half of the website page. There are links to images, although they are all scientific and of little use when educating the public.
3. Health Direct	https://www.healthdirect.gov.au/chest-injuries	The Health Direct website provides good information on chest injury, for both BCT and penetrating injuries. Headings are effectively used to segregate the information for blunt and penetrating wounds. The page is well laid out with bullet points highlighting key points. Among a list of possible chest injuries, there is extra information given on the most common ones. This shows that the primary aim of this website is to educate members of the public on potential BCT injuries, and use of non-technical language allows the page to do this effectively. An area in which the website

Name of Website	Website address	Website assessment
		lacks is the lack of images and diagrams, which can be effective in communicating some potential visible symptoms of chest injuries.
4. DynaMed	https://www.dynamed.com/management/blunt-chest-trauma-emergency-management#GUID-E3A89009-4D66-4D2B-9ABE-DF2E70EE0CFA	The general appearance of the DynaMed website is appealing, but the full content is not available as it requires a subscription for more detailed information. There are some colourful pictures but the explanation of these and the text in general is very scientific and appears to be more appropriate for clinicians than patients. A medical and or radiological understanding would be needed to interpret the x-ray photos.
5. Wikipedia	https://en.wikipedia.org/wiki/Chest_injury	While Wikipedia does not have a designated website page solely for BCT, it has a general blunt trauma page in which a section is designated to the thoracic region. While there is not a large volume of information on BCT, it contains causes and symptoms for certain chest injuries. However, the majority of the text contains medical terminology. The layout is confusing, with no subheadings and poor spacing. Some positives of the page included the use of tables that clearly outline some types of BCT accidents and what injuries they could cause, which was an effective tool for education on BCT. There were also a variety of links taking the reader to further information if they needed it.
6. North Bristol NHS Trust	https://www.nbt.nhs.uk/our-services/a-z-services/emergency-zone/ed-miu-patient-informationtt-information/chest-injury-advice	This is badged with an NHS and hospital logo which immediately gives credibility to the webpage. It is coloured in the usual blue and white NHS colour scheme. The webpage is titled "Chest injury advice- what happens when you're admitted to hospital with a chest injury', which allows the person accessing the site to determine the relevancy of the information. The language is very straightforward and there is clear delineation of sections. Bold text is used to highlight key headings in a large font. Dark font is used on a white background to aid contrast. The information is however very brief and there are no figures/diagrams to aid understanding. Useful contact information is included.

Name of Website	Website address	Website assessment
7. Drugs.com	https://www.drugs.com/cg/blunt-chest-trauma.html	The Drugs.com webpage has several adverts placed throughout the page. These take up a large part of the screen, including throughout the text sections. The information is largely to do with treatment and care for BCT injuries, with little information about causes and symptoms. Headings and bullet-points make the text appealing to read, but the lack of images hinders the visual appearance overall. Some abbreviations are used without reference to what they mean, which could confuse readers with no background knowledge. There are links to further information at the bottom of the page.
8. Gloucestershire Hospitals NHS Trust	https://www.gloshospitals.nhs.uk/media/documents/ Chest_trauma_GHPI0653_01_22_4EXEhZ5.pdf	This web address provides a link to a patient information leaflet about chest trauma. It is badged with the Gloucester Hospital logo and the NHS which immediately improves credibility. It is very clean and clear and has major headings that introduce chest trauma, what it is and how it can be managed. It also gives advice on when the patient should seek more help and contact information. The sentences are short and clear. There are no figures, tables and graphics which could potentially help aid understanding.
9. VeryWellHealth	https://www.verywellhealth.com/chest-trauma-3913241	This website has several adverts at the top and down the side of the page. The text itself focuses on all chest injuries rather than just BCT, with very little mention of BCT. The layout of text is good, with subheadings and images used well, as well as links to further information present. The language used is appropriate for most readers.
10. MSD Manual	https://www.msdmanuals.com/en-gb/home/injuries-and-poisoning/chest-injuries/blunt-injury-to-the-heart	This website contains information on chest injuries, with only some relevant to BCT. The introduction contains appropriate sections on symptoms, diagnosis, treatment for individuals with no clinical knowledge. However, the following sections use more advanced terminology that is not suitable for education of the public. There is limited use of images, and the text only makes use of around half of the page.

Name of Website	Website address	Website assessment
11. The American Association for the Surgery of Trauma	https://www.aast.org/resources-detail/blunt-cardiac-injury	The website visually looks very professional on first inspection. There are some adverts which distract from the information provided. The language is very clinical as would be expected of a professional body. The information also relies of medical knowledge to understand it. There are no figures/photographs or tables which could potentially aid understanding.
12. LifeInTheFastlane	https://litfl.com/thoracic-trauma/	This website is visually unappealing, with little use of colour, adverts down one side of the page, and too much empty white space. The information contained in the page appears aimed at medical professionals, as medical terminology is used throughout. The information also seems to be solely based on types of injury, and how they can be identified and treated by a medical professional. No images are used to accompany the text. There are no links to further information.
13. Radiology key	https://radiologykey.com/blunt-chest-trauma/	This website is organised in a very simple manner, with no adverts or pop-ups. The information contained however is too advanced, due to the use of medical terminology and images of x-rays and CT scans that would only be helpful to a medical professional. The layout of the text is also poor, with long sentences and paragraphs. There is no easy way to navigate through the website, due to the length of the text and lack of links to specific sections.
14. Broken or bruised ribs (NHS): multiple sites	https://www.eastenfieldmedicalpractice.nhs.uk/conditions/broken-or-bruised-ribs/	Many identified links in the NHS (in primary and secondary care) used the same standard text, under their name, with a clear badge that identified that they were an NHS site. This text was simple and clear and written in a language appropriate for a lay person. The focus was only on bruised or broken ribs rather than blunt chest trauma specifically. There was a brief summary to help patients identify if they had bruised/broken ribs, followed by self help information and how to get further advice or make contact. There were no distracting adverts, but also no figures/diagrams to aid understanding.

Name of Website	Website address	Website assessment
15. Oxford University Hospitals NHS Trust	https://www.ouh.nhs.uk/patient-guide/leaflets/files/10110Pchestinjury.pdf	This web address provides a link to a patient information leaflet about chest trauma. It is badged with the Oxford Hospital logo and the NHS which immediately improves credibility. It is very clean and clear and has major headings that introduce the relevant information. Bold headings are used to emphasise key information. There is limited use of colour and no figures/diagrams to illustrate the information. Contact details are included if the patient needs more information.
16. National Institute for Health and Care Excellence (NICE)	https://www.nice.org.uk/guidance/ng39/ifp/chapter/treating-chest-injuries	The NICE website contains information on all types of trauma injuries, making it only partially relevant to BCT. The page is visually appealing, with no adverts taking up space, and the text is large enough to read easily. A section is specified as 'information for the public', making it very clear to the reader which section is intended for their education. No images are used, but clear sub-headings and short paragraph lengths make the text visibly appealing and easy to navigate. Links to further information on care are clearly present.
17. Aneurin Bevan University Health Board	https://abuhb.nhs.wales/files/patient-information-leaflets1/accidents-and-minor-injuries/chest-injury-advice-sheet-pdf/	The web address links to a patient information leaflet about chest injury advice. The NHS and hospital logos increase credibility of the information. The language is fairly clear and straightforward but it looks quite cluttered with limited use of white space and as such is not very appealing. Red text and coloured text are sometimes used to used to highlight important areas but the colours are not appealing and may be difficult to see if printed. Some figures have been used to aid understanding.
18. My Health Alberta	https://myhealth.alberta.ca/Health/aftercareinformation/pages/conditions.aspx?hwid=uf7538	The MyHealth.Alberta website is focused on chest contusions, so again not solely on BCT. The information contained is quite brief, being mainly focused on care instructions for someone with a chest contusion. Further links to specific chest conditions are provided. There is no use of images or diagrams in addition to the text, however the page is clearly laid out with headings and bullet-points. The text could take up more of the page, with large areas of empty

Name of Website	Website address	Website assessment
		space. The language used is appropriate for someone with no medical background, due to almost no medical terminology used.
19. Salisbury NHS Foundation Trust	https://www.google.com/search?q=blunt+chest+trauma+salisbury&rlz=1C1CHBF_en-GBGB987GB987&oq=blunt+chest+trauma+salisbury&gs_lcrp=EgZjaHJvbWUyBggAEEUYOTIHCAEQIRigATIHCAIQIRigATIHCAMQIRigAdIBCDU1MTFqMWo5qAIAsAIA&sourceid=chrome&ie=UTF-8	The web address links to a patient information leaflet about blunt chest trauma. The language is very clear and straightforward but the information is brief and the leaflet is not particularly appealing with only black text on a white background. There are no NHS or hospital logos but the name of the NHS Trust provides a level of credibility. There are no figures to help illustrate but there basic management information is provided as well as contact information if required.
20. WebMD	https://www.webmd.com/heart/what-is-myocardial-contusion	There are lots of adverts throughout the text and to the right which distract from information provided. Many of the adverts are not relevant to the clinical information. The information itself is fairly easy to read and links are provided to linked information. There are however a large number of medical terms used which may limit understanding.
21. After Trauma	https://www.aftertrauma.org/diagnosis-and-treatment/managing-chest-wall-injuries	This website contains two pages dedicated to chest injuries. It focuses on the management of injuries, treatment and recovery, although some information was included on signs and symptoms. The page is well laid out, with good use of headings, sub-headings and bullet-points. There are no distracting adverts or unnecessary information, and there is good use of white space, making it easy to read. One clear diagram is used to show the anatomy of the chest, which complements the text. One negative is that there is medical terminology used throughout.
22. Royal Devon University Healthcare NHS Foundation Trust & Northern Devon Healthcare NHS Trust	https://www.royaldevon.nhs.uk/media/z51hk2mn/advice-after-a-chest-wall-injury.pdf	The web address links to a patient information leaflet about chest wall trauma. There are NHS and hospital logos which provides a level of credibility. The language is very clear and straightforward and provides background and self-help guidance as well as further contact information. It also includes clear figures that aid understanding. The language is clear.

Name of Website	Website address	Website assessment
23. South Tees Hospitals NHS Foundation Trust	https://www.southtees.nhs.uk/resources/chest-injury/	The website is badged with the hospital and NHS logos. It uses the blue and white NHS colouring so immediately appears credible. The information is all organised on one page and the language is straightforward. It provides a brief overview of chest injuires with symptoms, self-help guidance and contact information about seeking further help if needed. There is one generic image but nothing to help understand the information. It is clean and uncluttered with no advertisements.
24. Sirona care and health	https://www.sirona-cic.org.uk/wp-content/uploads/2022/02/0050_How-to-treat-a-Chest-Wall-web.pdf	This link takes you to a patient information leaflet titled 'how to treat a chest wall injury'. It is very professional looking and is badged with the NHS logo, thus improving credibility. There is limited use of colour on a plain white background. It provides an overview of symptoms, dos and don'ts, management and contacts in an emergency. There are no figures or tables and no adverts. The language is clear.
25. eMedicineHealth	https://www.emedicinehealth.com/wilderness_chest_injuries/article_em.htm	The emedicinehealth website contains information on chest injuries of all description, but specifically discusses BCT causes and injuries. The layout of the page is good, with white space and sub-headings used effectively to allow easy navigation of the text. Bullet-points are used to highlight key pieces of information, and pictures complement the text. The language used is appropriate, and there are links to access further information. However, there are some adverts present that may distract the reader.
26. Better Health Channel	https://www.betterhealth.vic.gov.au/health/conditionsandtreatments/rib-injuries	This is a very clean, uncluttered and professional looking website that focuses on rib injuries. Colour is used sparingly and with lots of white space. The information is broken down into clear headings. Some of the language used is a bit technical. There are no figures or tables which could aid understanding, but also no adverts to distract.
27. Rib Injury Clinic	https://www.ribinjuryclinic.com/conditions/complex-chest-wall-injuries/	On initial inspection this website looks quite appealing as it contains lots of images and photographs. It uses dark text on a light background but could benefit from more white space. Further

Name of Website	Website address	Website assessment
		inspection identifies that the conext is very complex as are the photographs and figures. It would be very difficult for a patient to understand the information. The language used is also highly technical. On the positive side there are no distracting adverts.
28. Hull University Teaching Hospitals NHS Trust	https://www.hey.nhs.uk/patient-leaflet/__trashed-35/	The link to this information first directs you to an 'advert' for accessibility testers, which is a little distracting and has to be closed before further information can be accessed. Once the information is accessed it is badged as the hospital and NHS which adds credibility. There is 'admin' information at the top of the page and the website menu to the right which makes the flow of information difficult. The information itself is a little complex but provides background, self-help guidance and where to go for more help. No figures or tables.
29. Mount Nittany Health	https://mountnittany.org/wellness-article/chest-wall-contusion	This website provides some basic information about 'chest contusion/brusing. It provides some background including a simple diagram of the chest. The language used is not too technical and it provides information about management and where to go for further help. No distracting adverts are included.
30. MedlinePlus	https://medlineplus.gov/chestinjuriesanddisorders.html	The website feels rather cluttered as there is admin information and a menu to the right. There are no adverts. The information provided is fairly simple and there are links to further information including how various chest conditions are diagnosed. Limited technical language is used in the main narrative, although many links use technical language.
31. European Society of Thoracic Surgeons	https://www.ests.org/about_ests/patient_information/diseases/chest_trauma.aspx#googtrans/en/en	This website has information spread over several pages. Information is not solely BCT related, however a large amount is relevant. The layout of the page is clear, with no adverts and good use of white space. Key pieces of information are highlighted using bullet-points. Some of the language used is too advanced for use by the public, and there is a lack of images giving a visual representation of the information in the text. There are links to further information.

Name of Website	Website address	Website assessment
32. Cleveland Clinic	https://my.clevelandclinic.org/health/diseases/23994-flail-chest	The website contains adverts at the top and to the right which distracts from the information provided. Generally the information looks fairly appealing and includes a simple figure. It focuses on 'flail chest'. It summarises key information but uses technical terms that make the information more difficult to understand.
33. mactheknife.org	www.mactheknife.org/Chest_Trauma/Mechanisms.html	This website provide some detail about the mechanisms of chest injory. There is a lot of text presented with limited line spacing on a patterned background. Visually this makes the information difficult to read especially for those with sight impairments. There are no figures or photos but also no distracting adverts. The content is very complex and lots of technical terms are used.
34. World Rugby Passport	https://passport.world.rugby/player-welfare-medical/first-aid-in-rugby/chapter-6-breathing-assessment-and-chest-injuries/potential-causes-of-rapid-breathing/chest-wall-or-lung-injuries/	This website presents a brief page of information about how to manage chest wall or lung injuries in rugby players. The website itself is very professional looking but the information is very limited and appears to be focused of how clinicians manage injuries. There are no figures or photos but equally no distracting adverts. The information presented is very clear and non-technical.
35. Total Vitality Medical Group	https://totalvitalitymedical.com/news/what-you-need-to-know-about-chest-contusions/	This website focuses on chest contusions/bruising and the page begins with a photograph of a bruised chest. The website looks very professional but the colours used could be improved as they may be problematic with some people with visual impairments and may not print well. The website gives a good overview of chest brusing, how it may be caused and how it can be managed. There are no distracting adverts. Generally is reads well, but some complex medical terms are used which make it more difficult to read.
36. Healthline	https://www.healthline.com/health/flail-chest	The Healthline website is organised in a visually appealing way with large text, easy-to-read font used and clear sub-headings. Adverts are present throughout the page, including pop-up windows, which are distracting and block the information from being read. No images are used, but bullet-points show key facts and highlighted

Name of Website	Website address	Website assessment
		phrases contain links to further information. Some language used is too advanced to be read by someone with no medical knowledge.
37. NHS Lanarkshire	https://www.nhslanarkshire.scot.nhs.uk/patient-information-leaflets/emergency-department/pil-chsinj-67661-l/	This website provides very brief information to patients who have presented with a chest injury to hospital. It includes the main signs and symptoms to consider and signposting should they need further advice. It is badged with the hospital name and NHS so adds credibility. There are no figures or photos but also no adverts. The language is straightforward and east to understand.
38. Cambridge University Hospitals NHS Foundation Trust	https://www.cuh.nhs.uk/patient-information/chest-injury-advice-sheet-for-patients/	This website is badged with the NHS and hospital logos which adds credibility. It provides very brief information about how patients should manage their chest injury after presenting to hospital. It mainly focuses on pain management and who to contact in an emergency. The language is very clear and simple. No images or adverts.
39. National University Hospital; National University Hospital Health System	https://www.nuh.com.sg/Health-Information/Diseases-Conditions/Pages/Chest-Injuries.aspx	The website is clean and uncluttered but the information contained within it is not really patient focused and uses lots of technical terms and diagrams where anatomy knowldege is assumed. There is limited helpful information for patients. There are no distracting adverts.
40. The Rotherham NHS Foundation Trust	https://www.therotherhamft.nhs.uk/patients-and-visitors/patient-information/chest-trauma-and-fractured-ribs#:~:text=If%20you%20do%20experience%20pain,please%20contact%20your%20GP%20practice.	This website is badged with the NHS and hospital logos which adds credibility. It provides comprehensive information about chest wall trauma and rib injuries and how patients should manage their injury after presenting to hospital. It is written in a very simple way and avoids using technical language. There are no figures to help illustrate the information but also no adverts.
41. Healthcare Associates of Texas	https://healthcareassociates.com/strained-chest-muscle-causes-symptoms-and-treatment/	The website feels rather cluttered as there is a menu on the right hand side and regular adverts interspersed with text to encourage users to 'book an appointment' which distracts from the information provided. There are no other adverts and some photos of people with possible chest injuries but no accomanying information. The information focuses of strained muscles, how you

Name of Website	Website address	Website assessment
		can get a muscle strain and treatment. The information is fairly clear and avoids the use of medical terminology.
42. Physio-pedia	https://www.physio-pedia.com/Sternal_fracture	Although this website does not contain external adverts, it feels rather cluttered as it advertises various physiotherapy courses before presenting the information on sternal fractures. There is a picture of the ribs, but no information to aid understanding. The information is fairly comprehensive but uses lots of medical terminology and long sentences which make understanding difficult.
43. Medical News Today	https://www.medicalnewstoday.com/articles/32453 4	Visually, the page is well laid-out, with good use of sub-headings, pictures and bullet-points to allow key information to be identified easily. The pictures are non-scientific, and work in conjunction with the text to demonstrate causes and treatments. Links at the top of the page allow easy movement between sections, which are concise and use space well. However, medical terminology is used throughout, and adverts and pop-ups may distract the reader.
44. My Family Physio	https://myfamilyphysio.com.au/common-upper-back-and-chest-injuries/#:~:text=Rib%20fractures%20and%20cartilage%20strains,pull%20starting%20a%20lawn%20mower).	The link to this website opens with a pop up message about still accepting appointments. This has to be closed before accessing the information which is distracting. The information provided is about common back and chest injuries so only parts of it are relevant. It includes some nice figures but limited explanation of them. The text contains a lot of medical terminology which makes it difficult to read.
45. Banner Health	https://www.bannerhealth.com/healthcareblog/better-me/what-to-know-about-strained-chest-muscles#:~:text=In%20most%20mild%20to%20moderate,area%20to%20help%20reduce%20pain.	This website focuses of the differences between chest injuries and a heart attack. It includes some photos but these do not really help to illustrate the text. There are no adverts. The article is largely clear but uses a lot of medical terminology.
46. Luna Physical Therapy	https://blog.getluna.com/pulled-chest-muscle-causes	This website provides a brief overview about pulled chest muscles, causes and treatment. The focus of the website is on physical therapy and the content largely reflects this. There are some photos but these do not aid the understanding of the content. The

Name of Website	Website address	Website assessment
		website is fairly clear and for the most part avoids the use of medical terminology.
47. Healthwise	https://www.cham.org/HealthwiseArticle.aspx?id=sig52119	This website is fairly bland with limited badging to identify the source, no figures, tables or photos. It does not have any adverts. Despite this it provides a brief summary about types of chest injuries as well as links to other conditions. The text avoids or explains medical terminology as far as possible which aids understanding.
48. Wirral Community Health and Care NHS Foundation Trust	https://www.wchc.nhs.uk/wp-content/uploads/2020/03/Chest-Injury_V1.pdf	This link takes you to a patient information leaflet titled 'chest injury'. It is very professional looking and is badged with the NHS and hospital logos, thus improving credibility. There is limited use of colour on a plain white background. There is a brief background to the injury as well as self-help guidance and how to get further help. No pictures or diagrams used. Medical terminology is avoided or explained and the language is very simple.
49. Barnsley Hospital NHS Foundation Trust	https://www.barnsleyhospital.nhs.uk/services/a-and-e/chest-injury	This website provides a very brief paragraph mainly to guide patients on its management but also to signpost for further help. It is badged with the hospital and NHS logos which improves credibility. The language is simple and clear. No figures or photos used. No adverts.
50. The Dudley Group NHS Foundation Trust	https://www.dgft.nhs.uk/wp-content/uploads/2023/09/Chest-injury-V3.pdf	This link takes you to a patient information sheet that describes chest injuries. It is badged with the hospital and NHS logos which improves credibility. It describes the causes, symptoms, how to manage it and where to go for further help. There are no figures or photos but the language is clear and limited medical terminology is used. There are no adverts.
51. York Hospitals	https://www.yorkhospitals.nhs.uk/seecmsfile/?id=6844	This link takes you to a patient information sheet that describes rib/chest injuries. It is badged with the hospital and NHS logos which improves credibility. It describes the causes, symptoms, how to manage it and where to go for further help. There are no figures or photos but the language is clear and limited medical terminology is used. There are no adverts.

Name of Website	Website address	Website assessment
52. Bradford Teaching Hospitals NHS Foundation Trust	https://digitalpatientinformationhub.bradfordhospitals.nhs.uk/leaflets/chest-injury/	This website provides very brief information mainly to guide patients on management of chest injuries following a visit to hospital. It also signposts for further help. It is badged with the hospital and NHS logos which improves credibility. The language is simple and clear. A simple illustration is used to illustrate a chest injury but otherwise there are no figures or photos used to help illustrate the text. No adverts.
53. Milton Keynes University Hospital	https://www.mkuh.nhs.uk/patient-information-leaflet/rib-or-chest-injury	This website provides a very brief paragraph mainly to guide patients on the management of rib or chest injuries but also to signpost for further help. It is badged with the hospital and NHS logos which improves credibility. The language is simple and clear. No figures or photos used. No adverts.
54. Dorset County Hospital	https://www.dchft.nhs.uk/wp-content/uploads/2020/10/ED-Chest-and-Rib-Injury-0414.pdf	This link takes you to a patient information sheet that describes how to manage rib/chest injuries onced discharged from hospital. It is badged with the hospital and NHS logos which improves credibility. It looks a little cluttered. There are no figures or photos but the language is clear and limited medical terminology is used. There are no adverts.
55. Mount Sinai	https://www.mountsinai.org/health-library/selfcare-instructions/rib-fracture-aftercar	This website provides information on rib fracture after care following presentation. It is nicely laid out and gives information about the injury, pain releif and what to expect. It also provides links to other conditions and references for further reading. The information is fairly straightforward although this is interspersed with medical terminology. There are no photos or illustrations and no external adverts although there is an 'internal advert' to book and appointment
56. Yale Medicine	https://www.yalemedicine.org/conditions/rib-fracture	This website provides an overview about rib fractures. The page itself looks rather cluttered as it has a menu on the left of the page. The colouring of some of the website may cause issues for those with sight impairments. The website itself looks fairly professional but the information is rather complex and includes lots of medical

Name of Website	Website address	Website assessment
		terminology. There is a photo included but this does not aid understanding of the text. No distracting adverts
57. Oxford Health NHS Foundation Trust	https://www.oxfordhealth.nhs.uk/wp-content/uploads/2014/08/OP-144.15-Rib-injury-advice.pdf	This link takes you to a patient information sheet that describes how to manage rib injuries. It is badged with the hospital and NHS logos which improves credibility. It is very brief but describes what a rib injury is and how it manage it. It also signposts contact information. There are no figures or photos but the language is clear and limited medical terminology is used. There are no adverts.
58. Brigham and Women's Hospital	https://www.brighamandwomens.org/lung-center/diseases-and-conditions/rib-fractures	This website looks rather cluttered as it immediately advertises appointments for patients which distracts from the main information about rib fractures. There is also a menu on the left of the page which is distracting. It includes a photograph at the top of the page but it is not relevant to rib fractures. The information itself is rather complex with lots of medical terminology. No other relevant illustrations are included but also no adverts.
59. topdoctors.co.uk	https://www.topdoctors.co.uk/medical-articles/rib-fractures-a-comprehensive-guide-for-patients	The website is rather cluttered with lots of pop-up messages before the information can be acccessed. It also includes information and prices to book an appointment which distracts from the information about rib fractures. There is a photo of the rib cage but this does not aid understanding of the text. It gives a reasonable summary about rib fractures but there is a lot of medical terminology used.
60. Ventura Orthopedics	https://venturaortho.com/difference-between-bruised-and-broken-ribs/	This website appears fairly professional initially. Further inspection however shows a ribbon of information including contact numbers, appointment booking and social media pages. There is a photo at the top of the page but it doesn't help to illustrate the content in any way. The information itself is fairly straightforward on the whole but does include some medical terminology. No adverts or other illustrations are included.
61. Nebraska Medicine	https://www.nebraskamed.com/trauma/help-broken-ribs-heal-faster	This website doesn't have dynamic adverts but includes bold information about booking an appointment which distracts from the information provided. It provides a brief overview about rib

Name of Website	Website address	Website assessment
		fractures which includes an x-ray photo of the ribcage. The photo does not aid in understanding the narrative provided. A lot of medical terminology and drug names are used which makes the content complex.
62. Dr Marco Scarci	https://www.marcoscarci.co.uk/what-are-the-long-term-effects-of-broken-ribs	The website looks cluttered with lots of photos and links to other articles to the right of the page. There are also interruptions to the text where links to make an appointment can be made. This distracts from the information provided. The text is quite small and there are no figures to aid understanding of the text. The information itself is a bit complex despite limited use of use of medical terminology. No adverts.
63. Doncaster and Bassetlaw Teaching Hospitals	https://www.dbth.nhs.uk/wp-content/uploads/2022/05/WPR48780-Rib-injury-leaflet.pdf	This link takes you to a patient information sheet that describes how to manage bruised or broken ribs. It provides a brief overview of do's and don'ts and where to get more advice. It also provides information about breathing exercises including photographs to illustrate. It is badged with the hospital and NHS logos which improves credibility. The language is clear and limited medical terminology is used. There are no adverts.
64. University Hospitals Coventry and Warwickshire	https://www.uhcw.nhs.uk/download/clientfiles/files/Patient%20Information%20Leaflets/Emergency%20Medicine/Emergency%20Department/118694_Emergency_Department_-_Bruised_or_broken_ribs_(1720)_-_March_2019.pdf	This link takes you to a patient information sheet that describes how to manage bruised or broken ribs following hospital discharge. It is badged with the hospital and NHS logos which improves credibility. It is very brief but describes what a rib injury is and how it manage it. It also signposts contact information. There are no figures or photos but the language is clear and limited medical terminology is used. There are no adverts.
65. Orthopaedics and Trauma London	http://www.orthopaedicsandtraumalondon.co.uk/conditions/rib-fractures/	This website looks fairly professional but gives the appearance of a sports website rather than a medical one, with photographs and sporting situations on the top of the page. It provides brief information about rib fractures, signs and symptoms, diagnosis and treatment. It provides a reasonable overview but the language is rather complex and there is a lot of medical terminology. No relevant illustrations and no adverts.

Name of Website	Website address	Website assessment
66. NCH Healthcare System	https://nchmd.org/health-library/diseases-and-conditions/con-20155268/	The top of this website is a bit cluttered and includes information about booking an appointment and paying bills etc. It also has a photo that is not related to the content on 'broken ribs' which appears lower down the page. The information contained is fairly well presented and includes an illustration of broken ribs. The page includes symptoms, causes, complications and treatment. The information is simple and avoids medical terminology. No distracting adverts.
67. Physiocheck	https://www.physiocheck.co.uk/condition/80/bruised-or-broken-rib	This website is very brightly coloured, but also feels very cluttered. There is a menu on the right hand side containing links to further information and how to make contact but this is quite distracting. It also includes active links for checking symptoms and asking a physio. There are a couple of illustrations of the ribs but these are difficult to understand for a lay person. The information provided on bruised or broken ribs is brief and includes a description, causes, signs and symptoms and treatment. It includes some medical terminology which makes comprehension more difficult but does try to explain these terms. No adverts.
68. Dr Gallagher and Partners	https://www.drgallagherandpartners.org.uk/website/M84035/files/rib%20inju.pdf	This link takes you to a patient information sheet that describes how to care for rib injuries. It looks very professional and describes the nature of the injuries, treatment and diagnosis. Some complex medical terminology is used but this is explained and the language is fairly simple. No illustrations to aid understanding but no adverts.
69. Agency for Clinical Innovation	https://aci.health.nsw.gov.au/networks/eci/clinical/ed-factsheets/rib-fracture	This website provides a fact sheet of information for people who have presented to the emergency department with rib fractures. Language is simple but a number of medical terms are used which may limit understanding. It provides a very brief overview of the condition and includes treatment and management. It also signposts to further contacts. It is clean and uncluttered and includes one illustration about breathing exercises. No adverts.

Name of Website	Website address	Website assessment
70. SportsMD.com	https://www.sportsmd.com/sports-injuries/chest-rib-injuries/rib-fracture-pneumothorax-complication/	The appearance of this website is generally ok, although the right of the page shows a large 'internal' advert promoting appointment bookings which is slightly distracting. There are a couple of illsurations that help with understanding of the text. The text is fairly comprehensive and includes types of injuries, complications, symptoms, causes and treatment. The page also includes a video. Some of the language is quite complex and includes a lot of medical terminology. No external adverts.
71. GP notebook	https://gpnotebook.com/simplepage.cfm?ID=-1120600051	This website opens with a number of pop-ups which are immediately distracting. Only limited information can be acccessed without subscription. The site is clearly labelled as a resource for healthcare professionals and the content reflects this as there is a lot of medical terminology and the language is very complex. The information provided is very brief. There are no illustrations. Some of the pages include dynamic adverts which are distracting.
72. Upstate University Hospital	https://www.upstate.edu/thoracic-surgery/conditions/rib-fracture.php	This website is clean and uncluttered and shows an image of the rib cage. It provides very brief information about rib fractures including causes, risk factors, symptoms and treatment. The information is very simple and brief and avoids medical terminology. There are no adverts to distract.
73. Fracture and Orthopaedic Clinic Ltd	https://www.faoconline.com/home/conditions/general/fracture-rib	This is a very professional looking website that provides information about fractured ribs including causes, symptoms and treatment. It is mainly black text on a white background and although the text size is adequate the text itself is quite light so may cause problems for people with sight issues. The language is a straightforward on the whole but includes medical terminology which may make comprehension difficult. There are no illustrations or adverts.
74. HealthyWA	https://www.healthywa.health.wa.gov.au/Articles/A_E/Chest-injuries-and-rib-fractures	This website is badged with the Department of Health Western Australia which adds credibility to the information. It provides very brief information about chest injuries and rib fractures and do's and don'ts but mainly signposts to further information if needed. There

Name of Website	Website address	Website assessment
		are no illustrations and no adverts. The language is very basic with limited used of medical terminology.
75. St James's Hospital	https://www.stjames.ie/media/Rib%20fracture%20Advice%20Leaflet.pdf	This link takes you to a patient information sheet that describes how to manage rib fractures. It is badged with the hospital logo which adds credibility to the information, however the quality of the logo is poor. It provides an overview, a list of do's and don'ts, but is primarily focused on signposting. The language is simple and clear with limited use of medical terms. There are no illustrations and no adverts.
76. International Centre for Thoracic Surgery	https://www.icts.com.sg/rib-fracture-fixation-from-trauma/	This website is a little overwhelming initially due its use of strong colours at the top of the page. The context itself is well laid out and comprehensive and includes some nice illustrations and photos that help to reinforce the messages in the text. There are no distracting adverts but the text is quite complex and includes a lot of medical terminology.
77. ISK Institute	https://www.iskinstitute.com/kc/abdomen/rib_fractures/rib_fractures.html	This website is very text heavy and not particularly appealing. The font size and colouring make reading the content difficult. There are error messages indicating some of the content is not supported. There are no images to help illustrate the text and no adverts. The language is very complex and includes a lot of medical terms.
78. University Hospitals Plymouth NHS Trust	https://www.plymouthhospitals.nhs.uk/display-pil/pil-chest-wall-injury-6063/	This website is badged with the hospital and NHS logos which adds credibility to the information. The website provides a brief overview of chest wall injuries and how they are managed. There is also signposting information to make further contacts if required. There are no illustrations and no adverts. The language is clear and simple with limited medical terminology used.
79. Masnad Health Clinic	https://www.masnad.com.au/knowledge-centre/injury-hub/middle-back/rib-cartilage-injury/	There are a number of pop-ups on this page advertising for appointments and to leave reviews, which are immediately distracting. There are some illustrations, including one of the rib cage and some photos illustrating breathing and stretching exercises which are useful. The information focuses on rib cartilages injuries.

Name of Website	Website address	Website assessment
		The information is quite comprehensive but the language is sometimes complex and includes lot of medical terminology.
80. Royal Berkshire Hospital	https://www.royalberkshire.nhs.uk/media/jfcjgxef/chest-wall-injury_dec21.pdf	This weblinks takes you to a patient information leaflet about chest wall injury. It provides information to patients who have been discharged. It is badged with the hospital and NHS logos which adds credibility to the information. The information provides a brief overview of chest wall injuries and how they are managed. There is also signposting information to make further contacts if required. There are no illustrations and no adverts. The information is simple and avoid medical terminology.
81. Chest Wall Injury Society	https://cwisociety.org/patientportal/	This website is a patient portal for those who have had a chest wall injury. There is an 'open area' that provides information about considerations before having treatment. The focus is very patient focused and the majority of the information is provided in the form of individual patient video stories. There is also a member only area. There are no adverts or other illustrations. Despite being a patient portal, the text information provided is limited and although limited medical terminology is used the language is quite complex.
82. UMRC Rochester	https://www.urmc.rochester.edu/encyclopedia/content.aspx?ContentTypeID=22&ContentID=FlailChest	This website is linked to a US Medical Center, which adds credibility to the information provided about 'flail chest'. The webpage is clean and uncluttered but does not include any illustrations. The information provided is very complex and includes lots of medical terminology. No adverts.
83. Tufts Medical Center Community Care	https://hhma.org/healthadvisor/aha-ribinjur-sma/	This website provides brief information about rib injuries, symptoms and treatment. The webpage is largely clear an uncluttered but the image is not available. The language is simple and avoids the use of medical terminology. There are no distracting adverts.
84. Farrell Physiotherapy	https://farrellphysiotherapy.co.uk/conditions-treated/thoracic-spine/rib-fractures/	The top half of this website is mostly black with an underlying x-ray image, but it is very difficult to see. The information about rib fractures begins about half way down the page and a brief overview is provided on the causes and symptoms. Most of the information

Name of Website	Website address	Website assessment
		is focused on physiotherapy treatments provided by the clinic to help manage rib fractures. There is an image of a back showing the rib cage within, but the image is not helpful in supportiong the text content. The orientation of the text and the use of capitals makes navigation of the website difficult. The text itself is fairly simple and medical terminology use is limited. No adverts.
85. healthinfo.org.nz	https://www.healthinfo.org.nz/patientinfo/439381.pdf	The link takes you to a patient information leaflet about rib injuries. The leaflet is badged with health board logos which adds credibility to the information. It provides a brief overview about symptoms and self care and where to go for more help. The information is clear and simple and avoids the use of medical terminology. There is a single photo of a fallen skiier. There are no adverts.

Supplementary information. Table S3. Readability assessment of the 85 websites identified on Google™, Yahoo™ and Bing™.

**Readability Assessments**	**Website**														
	1. Medscape			2. UptoDate			3. Health Direct			4. Dynamed			5. Wikipedia		
	Grade (US)	Grade (UK)	Age	Grade (US)	Grade (UK)	Age	Grade (US)	Grade (UK)	Age	Grade (US)	Grade (UK)	Age	Grade (US)	Grade (UK)	Age
Flesch-Kincaid	13.4	14.4	17-18	14.7	15.7	18+	6.5	7.5	11-12	11.2	12.2	18+	11.9	12.9	16-17
Gunning Fog	16.6	17.6	18+	17.4	18.4	18+	9.9	10.9	14-15	10.9	11.9	18+	13.6	14.6	18+
Coleman-Liau	15.1	16.1	18+	15.8	16.8	18+	8.8	9.8	13-14	13.9	14.8	18+	15.2	16.2	18+
SMOG	15.6	16.6	18+	16.4	17.4	18+	10.3	11.3	15-16	10.7	11.7	18+	11.9	12.9	16-17
Automated Readability	12.5	13.5	17-18	14.4	15.4	18+3	6.1	7.1	11-12	9.4	10.4	18+	10.3	11.3	15-16
Median readability grade	15.1	16.1	17-18	15.8	16.8	18+	8.8	9.8	13-14	10.9	11.9	18+	11.9	12.9	16-17
Flesch Reading Ease	22.5	Very difficult		21.1	Very difficult		68.6	Plain English		24.4	Very difficult		21.8	Very Difficult	
% of general public readable to	59%			50%			100%			76%			71%		
**Readability Assessments**	**Website**														
	6. North Bristol NHS Trust			7. Drugs.com			8. Gloucestershire Hospitals NHS Trust			9. Very well health			10. MSD Manual consumer version		
	Grade (US)	Grade (UK)	Age	Grade (US)	Grade (UK)	Age	Grade (US)	Grade (UK)	Age	Grade (US)	Grade (UK)	Age	Grade (US)	Grade (UK)	Age
Flesch-Kincaid	6.7	7.7	12-13	5.6	6.6	11-12	4.6	5.6	10-11	4.8	5.8	10-11	8.7	9.7	14-15
Gunning Fog	9.0	10.0	14-15	8.1	8.2	13-14	6.4	7.4	11-12	6.4	7.4	11-12	10.8	11.8	16-17
Coleman-Liau	9.6	10.6	15-16	9.0	10.0	14-15	8.1	9.1	13-14	7.4	8.4	13-14	11.5	12.5	17-18
SMOG	10.0	11.0	15-16	9.3	10.3	14-15	7.9	8.9	13-14	8.4	9.4	13-14	8.0	9.0	13-14
Automated Readability	6.5	7.5	11-12	5.5	6.5	10-11	4.5	5.5	9-10	4.6	5.6	9-10	11.2	12.2	16-17
Median readability grade	9.0	10.0	14-15	8.1	9.1	13-14	6.4	7.4	11-12	6.4	7.4	11-12	10.8	11.8	16-17
Flesch Reading Ease	65.7	Plain English		69.4	Easy		71.3	Fairly easy		79.37	Fairly easy		51.4	Fairly difficult	
% of general public readable to	100%			100%			100%			100%			95%		
**Readability Assessments**	**Website**														
	11. The American Association for the Surgery of Trauma			12. LITFL			13. Radiology Key			14. NHS			15. Oxford University Hospitals NHS Trust		
	Grade (US)	Grade (UK)	Age	Grade (US)	Grade (UK)	Age	Grade (US)	Grade (UK)	Age	Grade (US)	Grade (UK)	Age	Grade (US)	Grade (UK)	Age
Flesch-Kincaid	12.5	13.5	17-18	12.0	13.0	17-18	12.6	13.6	17-18	4.5	4.5	10-11	6.7	7.7	12-13

Gunning Fog	16.0	17.0	18+	13.1	14.1	18+	15.4	16.4	18+	7.2	8.2	12-13	9.2	10.2	14-15
Coleman-Liau	14.7	15.7	18+	15.3	16.3	18+	14.1	15.1	18+	6.9	7.9	12-13	9.0	10.0	14-15
SMOG	14.5	15.5	18+	12.3	13.3	18+	14.4	15.4	18+	8.2	9.21	13-14	10.0	11.0	15-16
Automated Readability	11.4	12.4	16-17	10.5	11.5	15-16	11.9	12.9	17-18	4.2	5.2	9-10	7.0	8.0	12-13
Median readability grade	14.5	15.5	18+	12.3	13.3	18+	14.1	12.8	18+	6.9	7.9	12-13	9.0	10.0	14-15
Flesch Reading Ease	28.8	Difficult		24.1	Very difficult		32.6	Difficult		81.09	Easy		71.0	Fairly easy	
% of general public readable to	66%			70%			66%			100%			100%		
**Readability Assessments**	**Websites**														
	16. NICE			17. Aneurin Bevan University Health Board			18. My Health Alberta			19.Salisbury NHS Foundation Trust			20. Web MD		
	Grade (US)	Grade (UK)	Age	Grade (US)	Grade (UK)	Age	Grade (US)	Grade (UK)	Age	Grade (US)	Grade (UK)	Age	Grade (US)	Grade (UK)	Age
Flesch-Kincaid	7.5	8.5	12-13	6.8	7.8	12-13	4.7	5.7	12-13	7.2	8.2	12-13	7.5	8.5	13-14
Gunning Fog	10.7	11.7	16-17	9.2	10.2	14-15	7.1	8.2	14-15	9.2	10.2	14-15	7.7	8.7	13-14
Coleman-Liau	8.6	7.6	14-15	8.9	9.9	14-15	6.9	7.9	14-15	9.7	10.7	15-16	11.2	10.2	16-17
SMOG	10.9	11.9	16-17	10.0	11.0	15-16	8.3	9.3	15-16	10.3	11.3	15-16	10.1	11.1	15-16
Automated Readability	7.8	8.8	13-14	7.0	8.0	12-13	4.5	5.5	12-13	7.1	8.1	12-13	7.1	8.1	12-13
Median readability grade	8.6	9.6	14-15	8.9	9.9	14-15	6.9	7.9	14-15	9.2	10.2	14-15	7.7	8.7	13-14
Flesch Reading Ease	70.9	Fairly easy		71.0	Fairly easy		81.7	Easy		65.5	Plain English		54.9	Fairly difficult	
% of general public readable to	100%			100%			100%			100%			100%		
**Readability Assessments**	**Websites**														
	21. After Trauma			22. Royal Devon University Healthcare			23.South Tees Hospitals NHS Foundation Trust			24. Sirona Health and care			25. eMedicinehealth		
	Grade (US)	Grade (UK)	Age	Grade (US)	Grade (UK)	Age	Grade (US)	Grade (UK)	Age	Grade (US)	Grade (UK)	Age	Grade (US)	Grade (UK)	Age
Flesch-Kincaid	13.3	14.3	18+	5.0	6.0	10-11	7.3	8.3	12-13	4.7	5.7	10-11	7.1	8.1	12-13
Gunning Fog	15.4	16.4	18+	7.4	8.4	12-13	9.1	10.1	14-15	6.6	7.6	12-13	8.3	9.3	13-14
Coleman-Liau	12.4	13.4	18+	8.3	9.3	13-14	11.2	12.2	16-17	6.5	7.5	12-13	9.0	10.0	14-15
SMOG	14.1	15.1	18+	8.6	9.6	14-15	10.4	11.4	15-16	7.9	8.9	13-14	10.7	11.7	16-17
Automated Readability	11.1	12.1	18+	7.8	8.8	13-14	7.8	8.8	13-14	3.6	4.6	9-10	5.7	6.7	11-12
Median readability grade	13.3	14.3	18+	7.8	8.8	13-14	9.1	10.1	14-15	6.5	7.5	12-13	8.3	9.3	13-14
Flesch Reading Ease	42.0	Difficult		73.4	Fairly easy		61.6	Plain English		71.0	Fairly easy		61.6	Plain English	
% of general public readable to	100%			100%			100%			100%			100%		

Readability Assessments	Website														
	26. Better Health Channel			27. Rib Injury Clinic			28. Hull University Teaching Hospitals NHS Trust			29. Mount Nittany Health			30. MedlinePlus		
	Grade (US)	Grade (UK)	Age	Grade (US)	Grade (UK)	Age	Grade (US)	Grade (UK)	Age	Grade (US)	Grade (UK)	Age	Grade (US)	Grade (UK)	Age
Flesch-Kincaid	6.9	7.9	12-13	10.7	11.7	16-17	9.7	10.7	15-16	5.2	6.2	10-11	7.0	8.0	12-13
Gunning Fog	9.2	10.2	13-14	12.4	13.4	18+	11.3	12.3	16-17	7.2	8.2	12-13	7.1	8.1	12-13
Coleman-Liau	9.9	10.9	14-15	12.2	13.2	18+	11.4	12.4	16-17	7.8	8.8	13-14	10.7	11.7	16-17
SMOG	10.2	11.2	16-17	15.5	16.5	18+	12.6	13.6	18+	8.7	9.7	14-15	9.8	10.8	15-16
Automated Readability	6.6	7.6	11-12	10.4	11.4	13-17	10.0	11.0	15-16	4.7	5.7	10-11	6.7	7.7	12-13
Median readability grade	9.2	10.2	13-14	12.2	13.2	18+	11.3	12.3	16-17	7.2	8.2	12-13	7.1	8.1	12-13
Flesch Reading Ease	64.3	Plain English		47.0	Difficult		54.3	Fairly difficult		74.5	Fairly easy		55.9	Fairly difficult	
% of general public readable to	100%			80%			88%			100%%			100%		
**Readability Assessments**	**Website**														
	31. European Society of Thoracic Surgeons			32. Cleveland Clinic			33. mactheknife			34. World Rugby Passport			35. Total Vitality Medical Group		
	Grade (US)	Grade (UK)	Age	Grade (US)	Grade (UK)	Age	Grade (US)	Grade (UK)	Age	Grade (US)	Grade (UK)	Age	Grade (US)	Grade (UK)	Age
Flesch-Kincaid	11.0	12.0	16-17	6.3	7.3	11-12	12.7	13.7	18+	6.2	7.2	11-12	10.3	11.3	15-16
Gunning Fog	13.4	14.4	18+	8.6	9.6	14-15	16.0	17.0	18+	10.0	11.0	15-16	12.4	13.4	18+
Coleman-Liau	12.8	13.8	18+	9.6	10.6	15-16	13.4	14.4	18+	7.6	8.6	13-14	12.2	13.2	18+
SMOG	12.8	13.8	18+	9.8	10.8	15-16	15.0	16.0	18+	10.4	11.4	15-16	13.0	14.0	18+
Automated Readability	11.0	12.0	16-17	6.1	7.1	11-12	12.1	13.1	18+	5.9	6.9	11-12	10.9	11.9	16-17
Median readability grade	12.5	13.5	18+	8.6	9.6	14-15	13.4	14.4	18+	7.6	8.6	13-14	12.2	13.2	18+
Flesch Reading Ease	37.4	Difficult		66.4	Plain English		34.8	Difficult		75.5	Fairly easy		50.3	Fairly difficult	
% of general public readable to	78%			100%			65%			100%			83%		
**Readability Assessments**	**Website**														
	36. Healthline			37. NHS Lanarkshire			38. Cambridge University Hospitals NHS Foundation Trust			39. National University Hospital; National University Hospital System			40. The Rotherham NHS Foundation Trust		
	Grade (US)	Grade (UK)	Age	Grade (US)	Grade (UK)	Age	Grade (US)	Grade (UK)	Age	Grade (US)	Grade (UK)	Age	Grade (US)	Grade (UK)	Age
Flesch-Kincaid	8.6	9.6	14-15	6.0	7.0	11-12	6.9	7.9	12-13	8.1	9.1	13-14	8.1	9.1	13-14

Gunning Fog	11.4	12.4	16-17	7.8	8.8	13-14	10.1	11.2	15-16	9.1	10.1	14-15	10.5	11.5	16-17
Coleman-Liau	10.2	11.2	15-16	8.7	9.7	14-15	7.9	8.9	13-14	11.7	12.7	17-18	9.9	10.9	15-16
SMOG	11.7	12.7	17-18	9.7	10.7	15-16	10.4	11.4	15-16	11.1	12.1	16-17	11.1	12.1	16-17
Automated Readability	8.3	9.3	13-14	5.1	6.1	10-11	6.6	7.6	12-13	8.4	9.4	13-14	8.1	9.1	13-14
Median readability grade	10.2	11.2	15-16	7.8	8.8	13-14	7.9	8.9	13-14	9.1	10.1	14-15	9.9	10.9	15-16
Flesch Reading Ease	59.5	Fairly difficult		65.7	Plain English		71.7	Plain English		57.4	Fairly difficult		61.7	Plain English	
% of general public readable to	95%			100%			100%			99%			99%		
**Readability Assessments**	**Website**														
	41. Healthcare Associates of Texas			42. Physio-pedia			43. Medical News Today			44. My Family Physio			45. Banner Health		
	Grade (US)	Grade (UK)	Age	Grade (US)	Grade (UK)	Age	Grade (US)	Grade (UK)	Age	Grade (US)	Grade (UK)	Age	Grade (US)	Grade (UK)	Age
Flesch-Kincaid	7.8	8.8	13-14	11.9	12.9	17-18	7.4	8.4	12-13	10.3	11.3	15-16	8.1	9.1	13-14
Gunning Fog	9.2	10.2	14-15	13.4	14.4	18+	9.2	10.2	14-15	12.1	13.1	18+	10.2	11.2	15-16
Coleman-Liau	11.2	12.2	16-17	15.3	16.3	18+	10.3	11.3	15-16	13.0	14.0	18+	10.1	11.1	15.-16
SMOG	10.3	11.3	15-16	13.2	14.2	18+	10.1	11.1	15-16	12.7	13.7	18+	10.6	11.6	16-17
Automated Readability	8.0	9.0	13-14	11.9	12.9	17-18	6.8	7.8	12-13	10.2	11.2	15-16	8.5	9.5	14-15
Median readability grade	9.2	10.2	14-15	13.2	14.2		9.2	10.2	14-15	12.1	13.1	18+	10.1	11.1	15-16
Flesch Reading Ease	59.1			33.3	Difficult		59.0	Fairly difficult		45.6	Difficult		65.3	Plain English	
% of general public readable to	100%	Fairly difficult		71%			100%			83%			99%		
**Readability Assessments**	**Website**														
	46. Luna Physical Therapy			47. Healthwise			48. Wirral Community Health and Care NHS Foundation Trust			49. Barnsley Hospital NHS Foundation Trust			50. The Dudley Group NHS Foundation Trust		
	Grade (US)	Grade (UK)	Age	Grade (US)	Grade (UK)	Age	Grade (US)	Grade (UK)	Age	Grade (US)	Grade (UK)	Age	Grade (US)	Grade (UK)	Age
Flesch-Kincaid	8.2	9.2	13-14	5.9	6.9	11-12	5.2	6.2	10-11	5.0	6.0	10-11	4.8	5.8	10-11
Gunning Fog	10.7	11.7	16-17	8.2	9.2	13-14	7.4	8.4	12-13	7.4	8.4	12-13	6.5	7.5	11-12
Coleman-Liau	10.5	11.5	15-16	6.6	7.6	12-13	8.1	9.1	13-14	8.3	9.3	13-14	7.0	8.0	12-13
SMOG	11.0	12.0	16-17	8.9	9.9	14-15	9.0	10.0	14-15	8.8	9.8	14-15	8.3	9.3	13-14
Automated Readability	8.5	9.5	13-14	4.9	5.9	10-11	4.7	5.7	10-11	5.6	6.6	11-12	3.7	4.7	9-10
Median readability grade	10.5	11.5	15-16	6.6	7.6	12-13	7.4	8.4	12-13	7.4	8.4	12-13	6.5	7.5	11-12
Flesch Reading Ease	61.8	Plain English		76.9	Plain English		72.5	Fairly easy		77.7	Fairly easy		72.4	Plain English	

% of general public readable to	98%			100%			100%			100%			100%		
**Readability Assessments**	**Website**														
	51. York Hospitals			52. Bradford Teaching Hospitals NHS Foundation Trust			53. Milton Keynes University Hospital			54. Dorset County Hospital			55. Mount Sinai		
	Grade (US)	Grade (UK)	Age	Grade (US)	Grade (UK)	Age	Grade (US)	Grade (UK)	Age	Grade (US)	Grade (UK)	Age	Grade (US)	Grade (UK)	Age
Flesch-Kincaid	4.5	5.5	10-11	4.5	5.5	10-11	5.8	6.8	11-12	3.9	4.9	9-10	7.2	8.2	12-13
Gunning Fog	7.4	8.4	12-13	6.1	7.1	11-12	8.6	9.6	14-15	6.2	7.2	11-12	9.6	10.6	15-16
Coleman-Liau	7.8	8.8	13-14	7.0	8.0	12-13	8.3	9.3	13-14	6.1	7.1	11-12	9.6	10.6	15-16
SMOG	8.5	9.5	13-14	8.3	9.3	13-14	9.8	10.8	15-16	7.8	8.8	13-14	10.3	11.3	15-16
Automated Readability	4.6	5.6	10-11	4.1	5.1	9-10	5.0	6.0	10-11	3.0	4.0	8.9	6.9	7.9	12-13
Median readability grade	7.4	8.4	12-13	6.1	7.1	11-12	8.3	9.3	13-14	6.1	7.1	11-12	9.6	10.6	15-16
Flesch Reading Ease	78.8	Fairly easy		79.2	Fairly easy		68.9	Plain English		78.9	Fairly easy		65.2	Plain English	
% of general public readable to	100%			100%			100%			100%			100%		
**Readability Assessments**	**Website**														
	56. Yale Medicine			57. Oxford Health NHS Foundation Trust			58. Brigham and Women's Hospital			59. topdoctors.co.uk			60. Ventura Orthopedics		
	Grade (US)	Grade (UK)	Age	Grade (US)	Grade (UK)	Age	Grade (US)	Grade (UK)	Age	Grade (US)	Grade (UK)	Age	Grade (US)	Grade (UK)	Age
Flesch-Kincaid	7.6	8.6	13-14	4.5	5.5	10-11	9.8	10.8	15-15	9.0	10.0	14-15	7.0	8.0	12-13
Gunning Fog	9.3	10.3	14-15	6.8	7.8	12-13	12.9	13.9	18+	11.6	12.6	17-18	9.3	10.3	14-15
Coleman-Liau	9.9	10.9	15-16	5.7	6.7	11-12	12.3	13.3	18+	10.1	11.1	15-16	9.3	10.3	14-15
SMOG	10.3	11.3	15-16	7.8	8.8	13-14	12.6	13.6	18+	11.6	12.6	17-18	10.0	11.0	15-15
Automated Readability	7.5	8.5	13-14	2.8	3.8	8-9	9.3	10.3	14-15	8.9	9.9	14-15	7.0	8.0	12-13
Median readability grade	8.5	9.5	14-15	5.7	6.7	11-12	12.3	13.3	18+	10.1	11.1	15-16	9.3	10.3	14-15
Flesch Reading Ease	60.7	Plain English		72.6	Fairly easy		47.5	Difficult		59.2	Fairly difficult		68.2	Plain English	
% of general public readable to	95%			100%			86%			92%			100%		
**Readability Assessments**	**Website**														
	61. Nebraska Medicine			62. Dr Marco Scarci			63. Doncaster and Bassetlaw Teaching Hospitals			64. University Hospitals Coventry and Warwickshire			65. Orthopaedics and Trauma London		
	Grade (US)	Grade (UK)	Age	Grade (US)	Grade (UK)	Age	Grade (US)	Grade (UK)	Age	Grade (US)	Grade (UK)	Age	Grade (US)	Grade (UK)	Age

Flesch-Kincaid	8.9	9.9	14-15	9.9	10.9	15-15	3.1	4.1	8-9	4.9	5.9	10-11	10.2	11.2	15-16
Gunning Fog	11.2	12.2	16-17	12.3	13.3	18+	4.9	5.9	10-11	7.0	8.0	12-13	13.1	14.1	18+
Coleman-Liau	10.6	11.6	16-17	11.1	12.1	16-17	5.5	6.5	10-11	8.4	9.4	13-14	12.2	13.2	18+
SMOG	11.5	12.5	17-18	12.5	13.5	18+	6.9	7.9	12-13	9.0	10.0	14-15	12.8	13.8	18+
Automated Readability	9.0	10.0	14-15	9.9	10.9	15-16	2.5	3.5	8-9	5.1	6.1	10-11	10.1	11.1	15-15
Median readability grade	10.6	11.6	16-17	11.1	12.1	16-17	4.9	5.9	10-11	7.0	8.0	12-13	12.2	13.2	18+
Flesch Reading Ease	58.9	Fairly difficult		53.5	Fairly difficult		85.3	Easy		73.2	Fairly easy		48.9	Difficult	
% of general public readable to	93%			93%			100%			100%			84%		
**Readability Assessments**	**Website**														
	66. NCH Healthcare System			67. Physiocheck			68. Dr Gallagher and Partners			69. Agency for Clinical Innovation			70. SportsMD.com		
	Grade (US)	Grade (UK)	Age	Grade (US)	Grade (UK)	Age	Grade (US)	Grade (UK)	Age	Grade (US)	Grade (UK)	Age	Grade (US)	Grade (UK)	Age
Flesch-Kincaid	4.9	5.9	10-11	6.2	7.2	11-12	6.5	7.5	11-12	7.6	8.6	13-14	9.3	10.3	14-15
Gunning Fog	7.2	8.2	12-13	8.5	9.5	14-15	9.0	10.0	14-15	9.9	10.9	15-16	11.6	12.6	17-18
Coleman-Liau	7.2	8.2	12-13	7.7	8.7	13-14	9.2	10.2	14-15	11.2	12.2	16-17	11.8	12.8	17-18
SMOG	8.6	9.6	14-15	9.7	10.7	15-16	9.6	10.6	15-16	10.4	11.4	15-16	12.0	13.0	17-18
Automated Readability	4.3	5.3	9-10	5.1	6.1	10-11	5.6	6.6	11-12	7.5	8.5	13-14	9.6	10.6	15-16
Median readability grade	7.2	8.2	12-13	7.7	8.7	13-14	9.0	10.0	14-15	9.9	10.9	15-16	11.6	12.6	17-18
Flesch Reading Ease	76.8	Fairly easy		70.1	Fairly easy		63.9	Plain English		57.6	Fairly difficult		54.5	Fairly difficult	
% of general public readable to	100%			100%			100%			100%			90%		
**Readability Assessments**	**Website**														
	71. GP notebook			72. Upstate University Hospital			73. Fracture and Orthopedic Clinic			74. HealthyWA			75. St James's Hospital		
	Grade (US)	Grade (UK)	Age	Grade (US)	Grade (UK)	Age	Grade (US)	Grade (UK)	Age	Grade (US)	Grade (UK)	Age	Grade (US)	Grade (UK)	Age
Flesch-Kincaid	7.8	8.8	13-14	4.4	5.4	9-10	4.4	5.4	9-10	10.2	11.2	15-16	4.0	5.0	9-10
Gunning Fog	9.3	10.3	15-16	5.2	6.2	10-11	5.2	6.2	10-11	13.1	14.1	18+	6.6	7.6	12-13
Coleman-Liau	9.5	10.5	15-16	7.1	8.1	12-13	7.1	8.1	12-13	12.2	13.2	18+	7.3	8.3	12-13
SMOG	10.1	11.1	15-16	7.7	8.7	13-14	7.7	8.7	13-14	12.8	13.8	18+	8.0	9.0	13-14
Automated Readability	6.9	7.9	12-13	3.9	4.9	9-10	3.7	4.7	9-10	10.1	11.1	15-16	4.0	5.0	9-10
Median readability grade	9.3	10.3	15-16	5.2	6.2	10-11	5.2	6.2	10-11	12.2	13.2	18+	6.6	7.6	12-13
Flesch Reading Ease	59.3	Fairly difficult		74.5	Fairly easy		74.5	Fairly easy		48.9	Difficult		79.3	Fairly easy	

% of general public readable to	96%			100%			100%			84%			100%		
**Readability Assessments**	**Website**														
	76. International Centre for Thoracic Surgery			77. ISK Institute			78. University Hospitals Plymouth NHS Trust			79. Masnad Health Clinic			80. Royal Berkshire Hospital		
	Grade (US)	Grade (UK)	Age	Grade (US)	Grade (UK)	Age	Grade (US)	Grade (UK)	Age	Grade (US)	Grade (UK)	Age	Grade (US)	Grade (UK)	Age
Flesch-Kincaid	7.8	8.8	13-14	9.1	10.1	14-15	6.7	7.7	12-13	8.2	9.2	13-14	5.2	6.2	10-11
Gunning Fog	9.7	10.7	15-16	11.4	12.4	16-17	8.9	9.9	14-15	9.8	10.8	15-16	7.8	8.8	13-14
Coleman-Liau	10.4	11.4	15-16	10.7	11.7	16-17	8.5	9.5	13-14	10.1	11.1	15-16	8.3	9.3	13-14
SMOG	10.6	11.6	16-17	11.7	12.7	17-18	9.9	10.9	14-15	11.5	12.5	17-18	8.8	9.8	14-15
Automated Readability	7.7	8.7	13-14	9.7	10.7	15-16	5.7	6.7	11-12	7.9	8.9	13-14	5.0	6.0	10-11
Median readability grade	9.7	10.7	15-16	10.7	11.7	16-17	8.5	9.5	13-14	9.8	10.8	15-16	7.8	8.8	13-14
Flesch Reading Ease	61.00	Plain English		59.8	Fairly difficult		67.2	Plain English		60.8	Plain English		73.5	Fairly easy	
% of general public readable to	100%			92%			99%			99%					
**Readability Assessments**	**Website**														
	81. Chest Wall Injury Society			82. UMRC Rochester			83. Tufts Medical Center Community Care			84. Farrell Physiotherapy			85. healthinfo.org.nz		
	Grade (US)	Grade (UK)	Age	Grade (US)	Grade (UK)	Age	Grade (US)	Grade (UK)	Age	Grade (US)	Grade (UK)	Age	Grade (US)	Grade (UK)	Age
Flesch-Kincaid	8.9	9.9	14-15	9.8	10.8	15-16	6.2	7.2	11-12	7.6	8.6	13-14	4.7	5.7	10-11
Gunning Fog	10.7	11.7	16-17	12.7	13.7	18+	9.3	10.3	14-15	8.8	9.8	14-15	7.7	8.7	13-14
Coleman-Liau	13.6	14.6	18+	11.5	12.5	16-17	8.3	9.3	13-14	10.1	11.1	15-16	6.9	7.9	12-13
SMOG	12.3	13.3	18+	12.5	13.5	18+	10.0	11.0	15-16	9.9	10.9	15-16	8.6	9.6	14-15
Automated Readability	10.4	11.4	15-16	9.9	10.9	15-16	5.5	6.5	11-12	7.2	8.2	12-13	3.7	4.7	9-10
Median readability grade	10.7	11.7	16-17	11.5	12.5	16-17	8.3	9.3	13-14	8.8	9.8	14-15	6.9	7.9	12-13
Flesch Reading Ease	51.1	Fairly difficult		53.2	Fairly difficult		69.9	Plain English		61.8	Plain English		74.0	Fairly easy	
% of general public readable to	93%			87%			100%			100%			100%		
