# Supplementary material for: Study to evaluate the readability and visual appearance of online resources for blunt chest trauma: an evaluation of online resources using mixed methods
Source: BMJ Open. 2024 Feb 6;14(2):e078552. doi: 10.1136/bmjopen-2023-078552 (PMC10860042; doi:10.1136/bmjopen-2023-078552)
Supplement: Author's manuscript [file bmjopen-2023-078552.draft_revisions.pdf]

BMJ Open is committed to open peer review. As part of this commitment we make the peer review history of every article we publish publicly available.

When an article is published we post the peer reviewers' comments and the authors' responses online. We also post the versions of the paper that were used during peer review. These are the versions that the peer review comments apply to.

The versions of the paper that follow are the versions that were submitted during the peer review process. They are not the versions of record or the final published versions. They should not be cited or distributed as the published version of this manuscript.

BMJ Open is an open access journal and the full, final, typeset and author-corrected version of record of the manuscript is available on our site with no access controls, subscription charges or pay-per-view fees (http://bmjopen.bmj.com).

If you have any questions on BMJ Open's open peer review process please email info.bmjopen@bmj.com

BMJ Open

A study to evaluate the readability and visual appearance of online resources for blunt chest trauma

Journal:	*BMJ Open*
Manuscript ID	bmjopen-2023-078552
Article Type:	Original research
Date Submitted by the Author:	04-Aug-2023
Complete List of Authors:	Hutchings, Hayley; Faculty of Medicine Health and Life Science, School of Medicine<br>Cochrane, Max; Faculty of Medicine Health and Life Science, School of Medicine<br>Battle, Ceri; Faculty of Medicine Health and Life Science, School of Medicine; Swansea Bay University Health Board, Physiotherapy Department
Keywords:	World Wide Web technology < BIOTECHNOLOGY & BIOINFORMATICS, Health Literacy, TRAUMA MANAGEMENT

SCHOLARONE™
Manuscripts

I, the Submitting Author has the right to grant and does grant on behalf of all authors of the Work (as defined in the below author licence), an exclusive licence and/or a non-exclusive licence for contributions from authors who are: i) UK Crown employees; ii) where BMJ has agreed a CC-BY licence shall apply, and/or iii) in accordance with the terms applicable for US Federal Government officers or employees acting as part of their official duties; on a worldwide, perpetual, irrevocable, royalty-free basis to BMJ Publishing Group Ltd ("BMJ") its licensees and where the relevant Journal is co-owned by BMJ to the co-owners of the Journal, to publish the Work in this journal and any other BMJ products and to exploit all rights, as set out in our licence.

The Submitting Author accepts and understands that any supply made under these terms is made by BMJ to the Submitting Author unless you are acting as an employee on behalf of your employer or a postgraduate student of an affiliated institution which is paying any applicable article publishing charge ("APC") for Open Access articles. Where the Submitting Author wishes to make the Work available on an Open Access basis (and intends to pay the relevant APC), the terms of reuse of such Open Access shall be governed by a Creative Commons licence – details of these licences and which Creative Commons licence will apply to this Work are set out in our licence referred to above.

Other than as permitted in any relevant BMJ Author's Self Archiving Policies, I confirm this Work has not been accepted for publication elsewhere, is not being considered for publication elsewhere and does not duplicate material already published. I confirm all authors consent to publication of this Work and authorise the granting of this licence.

For peer review only

A study to evaluate the readability and visual appearance of online resources for blunt chest trauma

Hayley A Hutchings[1*], Max Cochrane[1], Ceri E Battle[1,2].

[1]**Hayley Anne Hutchings**

https://orcid.org/0000-0003-4155-1741

Telephone: +44 (0) 1792 513412

Email: h.a.hutchings@swansea.ac.uk

[1]School of Medicine, Faculty of Medicine Health and Life Science, Singleton Park, Swansea SA2 8PP, U.K.

*Corresponding author

[1]**Max Cochrane**

Email: cochranemax1@gmail.com

[1]School of Medicine, Faculty of Medicine Health and Life Science, Singleton Park, Swansea SA2 8PP, U.K.

[1,2]**Ceri Elisabeth Battle**

Email: Ceri.Battle@wales.nhs.uk

https://orcid.org/0000-0002-7503-1931.

[2]Physiotherapy Department, Morriston Hospital, Swansea Bay University Health Board, Swansea, U.K.

For peer review only - http://bmjopen.bmj.com/site/about/guidelines.xhtml

Abstract

Objectives

Blunt chest trauma (BCT) is characterised by forceful and non-penetrative impact to the chest region. Increased access to the internet in recent years has led to online healthcare resources becoming used by the public to educate themselves on medical conditions. This study aimed to determine whether online resources for BCT are at an appropriate readability level and visual appearance for the public.

Design

We undertook a qualitative visual assessment of the identified website materials based on an adapted framework of pre-determined key criteria based on the Centers for Medicare and Medicaid Services toolkit and a readability assessment using Readable™ software.

Data Sources

Using a range of key search terms, we searched Google™, Bing™ and Yahoo™ websites for online resources about blunt chest trauma.

Results

We assessed 30 websites containing information on BCT. The median visual assessment score for the identified websites was 7, with a range of 1-9. The median readability score generated was 10.9 (15-16 years), with a range of 6.4-16.4. There was a significant association between the visual assessment and readability scores ($p<0.001$). The median score for Flesch reading ease was 49.5 (difficult). The study found that the readability level of the identified resources was much higher than the recommended level.

Conclusions

The visual appearance of websites was generally good, although better use of images and diagrams would improve the appearance of websites further. Less medical jargon and

For peer review only

shorter word and sentence length would allow the public to comprehend the contained information more easily.

Word count: 247

Strengths and Limitations

- This is the first study that has explored the readability and visual appearance of online resources for blunt chest trauma.
- We undertook a rigorous assessment of identified websites containing information on blunt chest trauma.
- We calculated extensive readability statistics for each website using five different readability scores.
- We supplemented the readability assessment with a qualitative visual assessment using defined criteria adapted from the Centers for Medicare and Medicaid Services toolkit.
- We searched for relevant website materials using three search engines therefore findings are limited to resources identified using these search engines.

Key words: world wide web technology, health literacy, trauma management

Funding Statement

This research received no specific grant from any funding agency in the public, commercial or not-for-profits sectors.

Conflicts of Interest

For peer review only - http://bmjopen.bmj.com/site/about/guidelines.xhtml

All authors confirm that there are no conflicts of interest to declare.

For peer review only

For peer review only - http://bmjopen.bmj.com/site/about/guidelines.xhtml

Introduction

Blunt chest trauma (BCT) is the leading cause of death among young adults, aged 15-44, and is second only to head trauma as the most common cause of death for all age groups[1]. The UK major trauma population is now reported to be more elderly, and the predominant mechanism is a fall from less than two metres[2].

Since the start of the century, internet usage across the globe has increased by 1,355%, with more than 5 billion internet users in the world as of 2023[3]. This huge increase in access to the internet has led to people using it as a healthcare resource, with 61% of Americans having looked up health related information[4]. A study found that 62% of people considered healthcare information on the internet to be "excellent" or "very good", and over half of people in the study felt they did not feel the need to share their findings with a doctor[5]. This suggests that many people see the internet as a valuable and accurate tool for healthcare. This number is likely to keep growing and this reliance on internet resources for all forms of healthcare means it is essential that information is accessible, accurate and readable.

Health literacy is defined as 'a person's ability to understand and use information to make decisions about their health' and is believed to have a vital impact on public health due to access and use of health services[6 7]. Between 43 and 61% of adults in the UK do not routinely understand health information[8], with more than 7 million adults having 'very poor literacy skills'[9]. This means that they experience difficulties reading from unfamiliar sources. Similarly in the USA, the average person can only read at an 8th grade level (age 12-13 years), meaning that they cannot interpret and evaluate any information that requires inference[10].

For peer review only - http://bmjopen.bmj.com/site/about/guidelines.xhtml

Low health literacy levels are a potential barrier to the use of online health resources. If the information is not able to be understood by a person without clinical knowledge, then this could hinder early diagnosis and treatment of potentially life-threatening conditions. For this reason, it is essential that online resources are written at a low readability level, meaning that they are simple to interpret and understand.

Previous studies have found that online resources for healthcare are at a high readability level and are of poor quality[11-14 15], showing that they contain medical jargon and are not visually appealing. For BCT, a study assessing the readability of a trauma surgery website found that the readability level was too high, and therefore difficult to comprehend[16].

Apart from the study assessing the readability of the trauma surgery website, there has been no previous research undertaken assessing the quality and readability of online resources for BCT. The aim of this study was therefore to carry out an assessment of the readability and visual appearance of online resources for BCT.

Methods

Identifying online resources

To identify relevant websites we used the top three most popular search engines in the UK: Google™, Bing™ and Yahoo™[17]. In order to capture the most relevant websites, we used a range of search terms: 'blunt chest trauma', 'blunt chest injury', 'chest impact injury', 'chest trauma', 'thoracic contusion' and 'forceful chest injury'. We identified the top ten websites from each search engine. We assessed the quality of each website, considering both the readability and the visual layout. Both qualitative and quantitative data were collected.

For peer review only - http://bmjopen.bmj.com/site/about/guidelines.xhtml

In order to evaluate the quality of online resources for BCT, we aimed to identify relevant websites that contained information intended to educate the public. We therefore excluded discussion sites, scientific papers, and any websites that required payment. This is because discussion sites and scientific papers are not resources put in place specifically to educate and inform the public about BCT, while websites that require payment receive low traffic, with previous research finding that 80% of people who encounter a paywall when looking for health information will choose to search elsewhere[3]. We included Wikipedia, despite the fact that its content can be changed and hence may not contain accurate information[18]. We decided to include it as it is a commonly used site and therefore it is important to assess its quality, due to the high level of traffic it receives.

Visual assessment

We undertook a qualitative visual assessment of the data that were collected from each website. We utilised the 'Guidelines for effective writing'[19], which details key areas that may affect the readability websites. These included headings, content organisation and language use, amongst others. We produced a short written summary for each website, outlining areas that were good or poor.

We scored the visual assessment for each website. We compiled a list of 10 criteria for the assessment based on the 'Guidelines for effective writing'[19] . We selected these criteria as we judged them to be the most important elements when assessing the written content and visual appearance of the webpage. For each of these 10 criteria we assigned a score of 1 point if the website performed well in that area, and a score of 0 if it performed poorly. These points added to give a cumulative score out of a possible 10 for each website.

For peer review only - http://bmjopen.bmj.com/site/about/guidelines.xhtml

Readability assessment

When assessing the readability of each website, we used the website Readable (https://readable.com) to generate several readability scores for each online BCT resource. To do this, we entered each page of text that was considered relevant to BCT into Readable. Readable then generated various readability statistics. All text that appeared on any relevant page was entered into readable, including headings and lists. However, all images, graphs and navigation areas were removed for the purposes of calculating readability. These were however considered as part of the visual assessment of the websites.

When analysing the content of our chosen websites, we assessed all the relevant pages, up to a maximum of 10 pages. We only included pages that specifically discussed BCT or related injuries and disregarded any that contained other healthcare information or contact details.

We used five different readability formulae to give a wide evaluation of each website. These were: Flesch-Kincaid Grade Index[20], Coleman-Liau Index[21], Simplified Measure of Gobbledygook Index[22], Gunning-Fog Index[23] and the Automated Readability Index[24]. We chose these readability formulae as they are widely used across media and have become an important tool in medicine for developing resources for the public (Readable, 2023). The most accurate way to score readability is to use several formulae, hence why we chose to use 5 in this study (Readable, 2023). Each formula is based on an equation that includes items such as words, characters, and syllables[12].

In addition to calculating the readability formulae, we also used Readable to generate Flesch Reading Ease scores[25] and to identify the percentage of the population the text could be read by. Readable generated scores as a school grade level, which could then be translated into a

For peer review only - http://bmjopen.bmj.com/site/about/guidelines.xhtml

corresponding age. This is the age that could be expected to comprehend the piece of writing. Readable suggests that writing aimed at the general public should score lower than a UK school grade 8, which translates to a reading age of 12-13 years. This means that the text is accessible to 85% of the population (Readable, 2023).

The readability scores were generated for each page of the website used, and aggregated to give an average score for each formula that was representative of the entire website.

Statistical analysis

We calculated median (range) readability scores for the five formulae for each website. We compared readability scores with the cumulative score from the visual assessment to determine if there was any correlation between readability and visual appearance, using non-parametric Spearman correlation. A p-value of less than 0.05 was regarded as being statistically significant.

Results

Identification and written assessment of online resources

Using the search terms across the three search engines, we identified a large amount of repetition of websites. We found the top 10 websites for each search engine were within the first five pages of results. There were a large number of scientific papers identified, especially when using search terms such as ‘thoracic contusion’. When using search terms containing less scientific language, more online resources that appeared were suitable for use by the public. Many of the resources that we identified were not solely based on BCT. Instead, they contained sections that were relevant to BCT as well as information on either other types of chest injury, or trauma injuries affecting the head or abdomen. Similarly, some of the resources were focused on types of injury caused by BCT, such as pneumothorax, flail chest and rib fractures.

For peer review only - http://bmjopen.bmj.com/site/about/guidelines.xhtml

For peer review only

Overall, there was very limited, patient focused information identified for BCT. Table 1 details the online resources identified, with a brief written assessment of their content.

Visual assessment

Details of the visual assessment for each online resource are shown in Table 2. The highest a website scored was 9, with four online resources achieving this score. There was a large amount of variability across the websites, with the scores ranging from 1 to 9. The lowest scoring resource was the Radiology key website, with an overall score of 1 from a possible 10. The median score was 7, indicating that most websites are well laid out and visually appealing.

Only three of the identified websites did not contain clearly BCT relevant information, instead focusing on a wider range of chest and trauma injuries. The second highest scoring factor was the use of text that was an appropriate size and font, which 25 of the online resources did. The worst areas across all the resources included the use of appropriate images or diagrams, which only 11 websites did, and the absence of medical jargon. Only 10 websites contained no medical jargon and used appropriate language for people with no previous medical or scientific knowledge.

Four of the 30 websites scored 9 for the visual appearance of their online resources (Health Direct, MSD, MedicalNewsToday, and EMedicinehealth). Positive characteristics across these resources were: the effective use of white space, with short sentences and paragraphs; clear sub-headings; bullet-pointed information that allowed the reader to browse the content easily; and further links to more specific information when appropriate. At first glance the online resources were visually appealing, with the use of images to aid the text and appropriate font to allow the content to be easily read. There was also a lack of advertisements and pop-ups

For peer review only - http://bmjopen.bmj.com/site/about/guidelines.xhtml

distracting the reader from key information. However, medical jargon was still used throughout three of these websites, which could potentially hinder users from being able to understand the information contained in the online resources.

The Radiology Key website only succeeded in providing accurate and relevant information, failing in all other areas that are key to providing effective writing. The text was long, with long paragraphs and little to break up the written information, like sub-headings or images. There was prolific use of scientific language and the information appeared to be aimed more at professionals rather than members of the public. Small text made the page visually unappealing, and there were no links to any further information that could be helpful to readers.

Readability assessment

There was a wide variation in the number of pages of relevant information regarding BCT on the identified websites. Most websites only had one page.

Table 3 illustrates the different readability scores for the identified websites along with the median scores for each online resource. The median score across all websites was 10.9, which equates to a reading age of 15-16 years of age. The range of scores was from 6.4 (11-12 years of age) to 16.4 (18+ years of age, or University level).

The range of Flesch Reading Ease scores was between 18.2 (very difficult) for the website UpToDate, to 81.9 (easy) for the website MyHealth.Alberta. The median Flesch Reading Ease score for the 30 online resources was 49.5 (difficult), with half of the websites (15/30) being scored as very difficult or difficult to read. Only two websites, MyHealth.Alberta and VeryWellHealth were classified as easy to read.

For peer review only - http://bmjopen.bmj.com/site/about/guidelines.xhtml

The percentage of the population that each resource would be readable to ranged from 46% for the website UpToDate, to 100%, which was scored by more than a third of the websites (12/30).

Comparison between visual assessment and readability scores

Figure 1 shows both the median readability score and the visual assessment score for each online resource. There was a large amount of variation between the websites for both categories. Some websites performed well in the readability assessment but badly in the visual assessment and vice versa. There was a tendency for websites with a higher readability score (i.e. more difficult to read) to have a lower visual assessment score. This suggests that there is a correlation between websites being difficult to read and being visually unappealing. A low readability score and a high visual assessment score is most desirable. There was a statistically significant moderate correlation between median readability score and visual assessment score (Spearman Rho = -0.633; p<0.001).

Discussion

Overall, we found that online resources for BCT were generally visually appealing, but the content was written at a level too high for the public. This shows resources available for public access are not providing accessible information. This may lead to is misunderstanding of the information being provided, and consequentially could result in slower diagnosis and greater risk of hospitalisation.

To be deemed readable for the general public, Readable suggests that a grade score of less than 8 should be achieved, which equates to a reading age of 12-13 years (Readable, 2023). 8th grade is the average reading age of an American adult[10], so if the readability score of online resources

For peer review only - http://bmjopen.bmj.com/site/about/guidelines.xhtml

are any higher than this, the information contained in the online resource will be inaccessible to more than half of the general public. We calculated a median readability score for the identified BCT resources of 10.9 (15-16 years), which is significantly higher than the recommended score of 8. Only four websites scored under 8, which suggests that online resources for BCT are not suitable for educating the public. This is supported by the fact that only 2 websites were classified as easy to read when considering the Flesh Reading Ease score[25], with half being difficult or very difficult to read.

Our findings concur with previous research assessing readability of online resources for other health conditions, such as phenylketonuria, fibroadenoma, otolaryngology and parathyroidectomy, which all identified that readability levels were too high[11-14]. This suggests that readability issues are common in online health resources, which could be a reason for up to 61% of UK adults not routinely understanding health information[8].

These finding suggest that it is essential for those writing online resources for BCT to consider their target audience, and to ensure that the content of the website is accessible and understandable for the public. Possible considerations to improve public accessibility include the use of shorter words and sentences and using less complex words and medical jargon throughout the text. Previous studies have demonstrated that shortening sentences to less than 15 words led to an improvement in readability[26]. In certain scenarios where using more complex terms is necessary, brief explanations or definitions can also be helpful to the reader. All these factors can contribute to the production of more readable online resources for BCT, leading to increased levels of education for the public regarding their health.

For peer review only - http://bmjopen.bmj.com/site/about/guidelines.xhtml

For the visual assessment of BCT resources, a median score of 7/10 was recorded, showing that the websites were generally well laid out and visually appealing. However, with no website scoring 10/10, there are still ways in which the visual appearance of these websites can be improved. Nearly half (14/16) of the websites were not visually appealing on first glance, for a number of reasons such as adverts taking up a large amount of room and either no colour or too much colour used.

It is important for the online resource to be appealing as this is the first thing a user will see and may impact whether they choose to continue to read the information present on the webpage. Even if a resource for BCT contains accurate and helpful information written at an appropriate readability level for a member of the public, not having a visually appealing page may cause the reader to look elsewhere.

In designing a website for BCT education, guidance can be taken from the 'guidelines for effective writing' from the Centres for Medicare and Medicaid services[19]. This will provide guidance on how best to structure text, with the use of paragraphing and headings essential to break up text into more manageable sections for readers. Using bullet-points to highlight key information is also beneficial to the reader, being clear and concise[27]. This is more appealing than having to search for information in a large block of text.

It is important to consider making information accessible to people who may have poor eyesight or have problems with reading. The use of images and audio aids can be effective in communicating BCT information to these audiences. Only 11/30 of the online resources assessed in this study used images or diagrams effectively, suggesting that less importance is placed on visual aids compared to written information. Previous research has found that the use

For peer review only - http://bmjopen.bmj.com/site/about/guidelines.xhtml

of audio and visual aids have a significant impact on learning[28]. This further highlights the importance of using these aids in future online resources for BCT.

It is also worth noting that there was a lack of online resources specifically aimed at educating the public on BCT. Many websites only partially discussed BCT or focused more on injuries that could be caused by BCT. In addition, several websites were aimed at audiences with more advanced scientific knowledge, however this was not clearly identifiable at first glance. These issues could both lead to confusion for the reader and dissuade them from looking for online information for BCT in the future.

Therefore, it is important for future online resources to not only be visually appealing and written at an appropriate level, but to be clearly identifiable as resources intended for use by the public. It is also essential for these websites to be focused entirely on BCT, so that any readers know that all the information contained is relevant.

There are several limitations to this study. A larger range of search terms could have been used, which may have identified more online resources. A larger number of resources could also have been identified for use. However, since the search engines we chose to use receive over 95% of traffic, and that 95% of people do not go past the first page of results[29] we believe that we identified the websites that would receive almost all the traffic.

When assessing readability and visual quality of the chosen resources, we only used 5 readability formulae, all of which assessed the readability based on sentence and word length and complexity. This did not consider other factors such as tables and diagrams that could also affect how easily a website can be read. Even though these were considered in the visual

For peer review only - http://bmjopen.bmj.com/site/about/guidelines.xhtml

assessment, this was only to a brief degree. A specific examination of visual and audio aids would be useful for future research to consider when assessing the quality of online resources.

Some websites with only a small amount of information on BCT may demonstrate an overly high readability score, and not be as representative of the overall readability of the website as those in which multiple pages of information were assessed. However, if someone is searching for BCT information, it is less important to them how readable other sections of the websites are. Further research is necessary, assessing specific aspects of visual appearance such as images in detail. Deeper analysis into the accuracy of the scientific content of the websites could also be useful, and other online resources such as videos or audio content should be considered.

Conclusions

We found that online resources for BCT were written at a level too advanced for use by the public, with a reading age greater than recommended. The visual appearance of these resources was generally good, although some areas such as the use of images could be improved. This suggests that BCT online resources are not as effective as they could be.

Total word count: 3381

Ethics Statement

<u>Patient Consent for Publication</u>

Not applicable

Ethics Approval

For peer review only - http://bmjopen.bmj.com/site/about/guidelines.xhtml

Not applicable.

Patient and Public Involvement statement

Due to the limited time available to complete this project and lack of funding, it was not possible to involve patients or the public in the design, or conduct, or reporting, or dissemination plans of our research.

Author Contributions

HAH designed the original study, quality assured the data collection and analysis, provided supervisory support and drafted the manuscript. MC adapted the study design, undertook the data searches, analysed the data, drafted data outputs and reviewed the final manuscript. CEB provided input into study design, provided clinical advisory support to the project, quality assured the outputs and reviewed the final manuscript.

Data availability

All original data from the study will be made available in the zenodo repository.

For peer review only

References

1. Fantus RJ, Clark DE. NTDB(R) data points: Annual report 2007: more than 2.7 in 2007. *Bull Am Coll Surg* 2008;93(1):62-3.
2. Kehoe A, Smith JE, Edwards A, et al. The changing face of major trauma in the UK. *Emerg Med J* 2015;32(12):911-5. doi: 10.1136/emermed-2015-205265
3. Broadband Search. Key internet statistics in 2023 (including mobile). 2023 [Available from: https://www.broadbandsearch.net/blog/internet-statistics accessed 28 July 2023.
4. Fox S, Jones S. The Social Life of Health Information. Americans' pursuit of health takes place within a widening network of both online and offline sources Washington: Pew Internet and American Life Project; 2009 [Available from: https://www.pewresearch.org/internet/2009/06/11/the-social-life-of-health-information/ accessed 28 July 2023.
5. Diaz JA, Griffith RA, Ng JJ, et al. Patients' use of the Internet for medical information. *J Gen Intern Med* 2002;17(3):180-5. doi: 10.1046/j.1525-1497.2002.10603.x
6. NHS Digital Service Manual. Design and build digital services for the NHS. 2023 [Available from: https://service-manual.nhs.uk accessed 28 July 2023.
7. Vandenbosch J, Van den Broucke S, Vancorenland S, et al. Health literacy and the use of healthcare services in Belgium. *J Epidemiol Community Health* 2016;70(10):1032-8. doi: 10.1136/jech-2015-206910 [published Online First: 20160426]
8. Berry J. Does health literacy matter? : NHS England; 2016 [Available from: https://www.england.nhs.uk/blog/jonathan-berry/ accessed 28 July 2023.
9. National Literacy Trust. What is Literacy? [Available from: https://literacytrust.org.uk/information/what-is-literacy/ accessed 28 July 2023.
10. National Centre for Education Statistics. Program for the International Assessment of Adult Competencies (PIAAC) 2023 [Available from: https://nces.ed.gov/surveys/piaac/ accessed 28 July 2023.
11. Byrne J, Keogh S, Cullinane C, et al. Readability and Quality of Online Health Information Regarding Parathyroidectomy. *OTO Open* 2022;6(4):2473974X221133308. doi: 10.1177/2473974X221133308 [published Online First: 20221020]
12. Hutchings HA, Remesh A. An evaluation of the readability and visual appearance of online patient resources for fibroadenoma. *PLoS One* 2022;17(11):e0277823. doi: 10.1371/journal.pone.0277823 [published Online First: 20221118]
13. Marsh JM, Dobbs TD, Hutchings HA. The readability of online health resources for phenylketonuria. *J Community Genet* 2020;11(4):451-59. doi: 10.1007/s12687-020-00461-9 [published Online First: 20200327]
14. Grose EM, Holmes CP, Aravinthan KA, et al. Readability and quality assessment of internet-based patient education materials related to nasal septoplasty. *J Otolaryngol Head Neck Surg* 2021;50(1):16. doi: 10.1186/s40463-021-00507-z [published Online First: 20210317]
15. Martin CA, Khan S, Lee R, et al. Readability and Suitability of Online Patient Education Materials for Glaucoma. *Ophthalmol Glaucoma* 2022;5(5):525-30. doi: 10.1016/j.ogla.2022.03.004 [published Online First: 20220314]
16. Eltorai AE, Ghanian S, Adams CA, Jr., et al. Readability of patient education materials on the american association for surgery of trauma website. *Arch Trauma Res* 2014;3(2):e18161. doi: 10.5812/atr.18161 [published Online First: 20140430]
17. Search Engine Insight. Top 10 Search Engines In The World (2022 Update) [Available from: https://www.searchengineinsight.com/top-10-leading-search-engines-uk/ accessed 31 July 2023.
18. Azer SA. Is Wikipedia a reliable learning resource for medical students? Evaluating respiratory topics. *Adv Physiol Educ* 2015;39(1):5-14. doi: 10.1152/advan.00110.2014

For peer review only - http://bmjopen.bmj.com/site/about/guidelines.xhtml

19. CMS.gov, Centers for Medicare and Medicaid Services. Toolkit for Making Written Material Clear and Effective. [Available from: https://www.cms.gov/outreach-and-education/outreach/writtenmaterialstoolkit accessed 31 July 2023.
20. Kincaid JP, Fishburne RP, Rogers RL, et al. Derivation of new readability formulas (automated readability index, fog count, and flesch reading ease formula) for Navy enlisted personnel. Research Branch Report 8–75. Available from: https://apps.dtic.mil/dtic/tr/fulltext/u2/a006655.pdf (access date 22 January 2020). Chief of Naval Technical Training. Naval Air Station Memphis, 1975.
21. Coleman M, Liau TL. A computer readability formula designed for machine scoring. *Journal of Applied Psychology* 1975;60:283-84.
22. McLaughlin GH. SMOG Grading — a New Readability Formula. *Journal of Reading* 1969;12(8):639-46.
23. Gunning R. The Technique of Clear Writing: McGraw-Hill 1952.
24. Smith EA, Senter RJ. Automated Readability Index. AMRI.-TR-66-220. Available from: https://apps.dtic.mil/dtic/tr/fulltext/u2/667273.pdf (access date 13 January 2020). Defense Documentation Center, Virginia: Wright-Patterson Air Force Base, Ohio, 1967.
25. Interpretation of Flesch Reading Ease Score (Access date 16 August 2022) [Available from: https://writingstudio.com/blog/flesch-reading-ease/.
26. Sheppard ED, Hyde Z, Florence MN, et al. Improving the readability of online foot and ankle patient education materials. *Foot Ankle Int* 2014;35(12):1282-6. doi: 10.1177/1071100714550650 [published Online First: 20140919]
27. Gov.uk. Style guide [Available from: https://www.gov.uk/guidance/style-guide/a-to-z-of-gov-uk-style accessed 3 August 2023.
28. Carey M, Schofield P, Jefford M, et al. The development of audio-visual materials to prepare patients for medical procedures: an oncology application. *Eur J Cancer Care (Engl)* 2007;16(5):417-23. doi: 10.1111/j.1365-2354.2006.00772.x
29. Protofuse. The First Page Of Google, By The Numbers [Available from: https://protofuse.com/articles/first-page-of-google-stats/ accessed 3 August 2023.

For peer review only

For peer review only - http://bmjopen.bmj.com/site/about/guidelines.xhtml

Table 1. Written assessment of each identified BCT online resource

Name of Website	Website address	Website assessment
1. Medscape	https://emedicine.medscape.com/article/428723-overview?form=fpf	The Medscape website is clearly laid out, with bold headings, sub-headings and regular use of paragraphs. This makes it aesthetically appealing, with clear links to move between different sections of the article. However, there is heavy use of medical jargon and information that is not relevant or useful to the public as it details complex anatomical and physiological structures. There is no use of diagrams, only an image of an x-ray. The website does include information on causes, symptoms and treatments of BCT. In conclusion, this website appears more focused on use by clinicians than by the general public.
2. National Health Service (NHS) North Bristol NHS Trust	https://www.nbt.nhs.uk/our-services/a-z-services/emergency-zone/ed-miu-patient-informationtt-information/chest-injury-advice	This website gives information to patients who have suffered from BCT injuries. It is informative about different comorbidities associated with BCT, including treatments and symptoms. The document is clearly laid out, with easy-to-read terminology. Key points were highlighted, and bullet pointed, with information kept to a minimum level that is suitable for its aimed audience. A negative feature of this website is that it is aimed at people who had already been given a diagnosis, and so even though it contains relevant information around BCT, it assumes the individual already is aware of any injuries they have.
3. UpToDate	https://www.uptodate.com/contents/initial-evaluation-and-management-of-blunt-thoracic-trauma-in-adults	The UpToDate website page contains very informative content regarding BCT. There are links to further information on specific injuries, however this website focuses more on the anatomical nature of these injuries rather than symptoms and how to diagnose them. In general, this website seems to focus on information relevant to a clinician rather than a member of the public, with detailed descriptions of medical techniques. The layout is poor, as the text area only takes up around half of the website page. There are links to images, although they are all scientific and of little use when educating the public.
4. Health Direct	https://www.healthdirect.gov.au/chest-injuries	The Health Direct website provides good information on chest injury, for both BCT and penetrating injuries. Headings are effectively used to segregate the information for blunt and penetrating wounds. The page is well laid out with bullet points highlighting key points. Among a list of possible chest injuries, there is extra information given on the most common ones. This shows that the primary aim of this website is to educate members of the public on potential BCT injuries, and use of non-technical language allows the page to do this effectively. An area in which the website is poor is the lack of images and diagrams, which can be effective in communicating some potential visible symptoms of chest injuries.

For peer review only - http://bmjopen.bmj.com/site/about/guidelines.xhtml

5. Wikipedia	https://en.wikipedia.org/wiki/Blunt_trauma	While Wikipedia does not have a designated page solely for BCT, it has a general blunt trauma page in which a section is designated to the thoracic region. While there is not a large volume of information on BCT, it contains causes and symptoms for certain chest injuries. However, the majority of the text contains medical jargon. The layout is confusing, with no subheadings and poor spacing. Some positives of the page include the use of tables that clearly outline some types of BCT accidents and what injuries they could cause, which is an effective tool for education on BCT. There are also a variety of links taking the reader to further information if they needed it.
6. Science Direct Topics	https://www.sciencedirect.com/topics/medicine-and-dentistry/thorax-blunt-trauma	The Science Direct website does not appear to be aimed at use by those without a scientific or medical background. It contains pieces of text from multiple different books or pages surrounding BCT, with links to take you to each full text. The information is primarily based on injuries to the chest. The page is well laid out with bullet-points showing key information and clear space used between pieces of text allowing easy differentiation between sections. This page is not suitable to educate those without a medical background, with advanced language used.
7. MSD Manual	https://www.msdmanuals.com/en-gb/home/injuries-and-poisoning/chest-injuries/introduction-to-chest-injuries	This website contains information on chest injuries, with only some relevant to BCT. The introduction contains appropriate sections on symptoms, diagnosis, treatment for individuals without any clinical knowledge. However, the following sections use more advanced terminology that is not suitable for education of the public. There is limited use of images, and the text only makes use of around half of the page. Again, this website is also not solely dedicated to BCT.
8. After Trauma	https://www.aftertrauma.org/diagnosis-and-treatment/managing-chest-wall-injuries	This website contains one page dedicated to chest injuries, again not being solely dedicated to BCT. It focuses on the management of injuries, treatment and recovery, although some information was included on signs and symptoms. The page is well laid out, with good use of headings, sub-headings and bullet-points. There are no distracting adverts or unnecessary information, and there is good use of white space, making it easy to read. One negative is that there is medical jargon used throughout.
9. MyHealth.Alberta	https://myhealth.alberta.ca/Health/aftercareinformation/pages/conditions.aspx?hwid=uf7538	The MyHealth.Alberta website is focused on chest contusions, so again not solely on BCT. The information contained is quite brief, being mainly focused on care instructions for someone with a chest contusion. Further links to specific chest conditions are provided. There is no use of images or diagrams in addition to the text, however the page is clearly laid out with headings and bullet-points. The text could take up more of the page, with large areas of empty space. The language used is appropriate for someone without a medical background, due to almost no jargon used.
10. Impact Medical Group	https://www.impactmedicalgroup.com/2022/03/11/how-serious-is-a-chest-contusion-and-how-can-i-treat-it/	This website is primarily focused on chest contusions and assumes that the injury has been linked to a car accident. This makes this resource less useful for anyone with BCT

For peer review only - http://bmjopen.bmj.com/site/about/guidelines.xhtml

		injuries from other causes. The layout is clear, with headings and short paragraphs used. Key points are highlighted and there are links to further information at the bottom of the page, with little to no medical jargon used within the text. However, the main aim of the page seems to be to advertise the medical service provided by Impact Medical Group, so it calls into question the priorities of the website. There are also no images used.
11. Healthlink	https://www.healthlinkbc.ca/illnesses-conditions/injuries/types-chest-injuries	The focus of this webpage is chest injuries in general, not solely BCT. However, it still contains relevant information around injuries that could be caused by blunt trauma. The layout is clear, with the use of headings and bullet-points, as well as links to further information regarding chest problems. There are several negative features associated with this page, including lack of images or diagrams, some use of medical jargon and in general a shortage of information on treatments and causes of injury.
12. Osmosis	https://www.osmosis.org/answers/blunt-trauma	This website is focused on blunt trauma across the whole body but contains relevant information for BCT. The information is thorough and up to date. Links to further information are highlighted throughout the text, which is easy to read due to large text size and good use of spacing. Pictures are used to demonstrate associated conditions effectively, being clear and simple. Some adverts are present throughout the website. Educational videos are also used in addition to the text and pictures, giving a beneficial visual and auditary asset to the information.
13. Drugs.com	https://www.drugs.com/cg/blunt-chest-trauma.html	The Drugs.com webpage has several adverts placed throughout the page. These take up a large part of the screen, including throughout the text sections. The information is largely to do with treatment and care for BCT injuries, with little information about causes and symptoms. Headings and bullet-points make the text appealing to read, but the lack of images hinders the visual appearance overall. Some abbreviations are used without reference to what they mean, which could confuse readers with no background knowledge. There are links to further information at the bottom of the page.
14. VeryWellHealth	https://www.verywellhealth.com/chest-trauma-3913241	This website has several adverts at the top and down the side of the page. The text itself focuses on all chest injuries rather than just BCT, with very little mention of BCT. Overall, the information is not of much use when regarding BCT in isolation. The layout of text is good, with subheadings and images used well, as well as links to further information present. The language used is appropriate for most readers.
15. Healthline	https://www.healthline.com/health/bruised-lung-pulmonary-contusion	The Healthline website mainly aims at addressing pulmonary contusions, which is a common injury for those who suffer BCT. It is organised in a visually appealing way with large text, easy-to-read font used and clear sub-headings. Adverts are present throughout the page, including pop-up windows, which are distracting and block the information from being read. No images are used, but bullet-points show key facts and

For peer review only - http://bmjopen.bmj.com/site/about/guidelines.xhtml

		highlighted phrases contain links to further information. Some language used is too advanced to be read by someone without any medical knowledge.
16. Britannica	https://www.britannica.com/science/impact-injury	At first glance this website is very confusing, with adverts down the side of the page, at the bottom of the screen and throughout the text. The page focuses on all types of impact injuries, which is a far broader topic than just BCT. No headings or images are used, with long paragraphs and medical jargon making the text difficult to read. Related articles are shown beneath this one, with links in the text allowing access to further information.
17. MedicalNewsToday	https://www.medicalnewstoday.com/articles/322817#causes	This website contains information regarding breast trauma, which falls under the category of a BCT injury. Visually, the page is well laid-out, with good use of sub-headings, pictures and bullet-points to allow key information to be identified easily. The pictures are non-scientific, and work in conjunction with the text to demonstrate causes and treatments. Links at the top of the page allow easy movement between sections, which are concise and use space well. However, medical jargon is used throughout, and adverts and pop-ups may distract the reader.
18. Trauma Victoria	https://trauma.reach.vic.gov.au/guidelines/thoracic-trauma/introduction	The first impression of this website is good, with no adverts distracting the reader and a clear title to the page. An image of the chest is used to articulate the focus of the page, and the text uses most of the available space. However, the text itself has no sub-headings dividing it into sections, and long paragraphs make it unappealing to read. The over-use of statistics throughout the text, coupled with the use of medical jargon, makes it difficult for the average reader to comprehend. Links are given to further information.
19. National Institute for Health and Care Excellence (NICE)	https://www.nice.org.uk/guidance/ng39/ifp/chapter/treating-chest-injuries	The NICE website contains information on all types of trauma injuries, making it only partially relevant to BCT. The page is visually appealing, with no adverts taking up space, and the text is large enough to read easily. A section is specified as 'information for the public', making it very clear to the reader which section is intended for their education. No images are used, but clear sub-headings and short paragraphs make the text visibly appealing and easy to navigate. Links to further information on care are clearly present.
20. SteadyHealth.com	https://www.steadyhealth.com/medical-answers/have-you-suffered-a-punch-to-the-chest-signs-you-need-to-see-a-doctor	This page is poorly laid out, with a large amount of empty space and adverts present. Only a small amount of information is given, mostly regarding complications from BCT that affect the heart and lungs. A picture of a man holding his chest shows the reader the area affected by BCT, aiding the text in describing chest injuries. Bullet-points highlight key information. Links to further information are difficult to see as they are surrounded by adverts. Medical jargon is used occasionally.

For peer review only - http://bmjopen.bmj.com/site/about/guidelines.xhtml

21. AICA orthopaedics	https://aica.com/what-does-chest-pain-mean-after-a-car-accident/	This website is clearly laid out, with a clear title outlining the purpose of the page. Good use of colours and sub-headings make the page visually appealing, although shorter paragraphs would have benefitted the text. The information contained is mostly regarding BCT in the context of a car accident. BCT is not explicitly mentioned. The language used is appropriate for someone without any medical background to understand, and links to further information are included within the text. Overall, this website contains useful information in an appropriate format for someone with BCT injuries sustained through a car accident.
22. Boohoff Law	https://www.boohofflaw.com/chest-injury-from-car-accident/	At first look, this website appears to be aimed at providing a service to people involved in car accidents, with a large amount of space dedicated to promoting legal help. However, there is still a large section of text containing relevant and accurate information around chest contusions and BCT. The page is visually appealing, with good use of colour, images and sub-headings. Bullet points highlight key points. Some areas that could be improved include shortening of paragraphs and more use of white space. Some medical jargon is also used.
23. LifeInTheFastlane	https://litfl.com/thoracic-trauma/	This website is visually unappealing, with little use of colour, adverts down one side of the page, and too much empty white space. The information contained in the page appears aimed at medical professionals, as medical jargon is used throughout. The information also seems to be solely based on types of injury, and how they can be identified and treated by a medical professional. No images are used to accompany the text. There are no links to further information on BCT.
24. Firstresponse	https://www.firstresponse.org.uk/first-aid-az/3-general/first-aid/62-chest-injuries	This page primarily focuses on how to deal with chest trauma injuries as a first responder. It has information on BCT and penetrative chest trauma. The layout of the page is simple, with good use of sub-headings and bullet-points to allow the reader to find information easily. One image is used; however, it is unclear what it shows and is therefore unhelpful. The language used is appropriate for someone without any medical knowledge to read, although some terms that are used could benefit from more explanation of what they mean. There are no links to further information.
25. Oxford University Hospitals NHS Trust	https://www.ouh.nhs.uk/patient-guide/leaflets/files/10110Pchestinjury.pdf	This page is unique since it is a pdf document instead of a website. However, it contains accurate information regarding BCT. There are no distracting adverts, and good use of language and bullet-points makes the text easy to read. The text uses headings and paragraphs well, spacing the text out to make it more visually appealing. Some negative aspects are the lack of images to aid the text in describing types of injury, and there are no links to further information given.

For peer review only - http://bmjopen.bmj.com/site/about/guidelines.xhtml

26. EMS World	https://www.hmpgloballearningnetwork.com/site/emsworld/article/10727945/thoracic-trauma-what-you-need-know	This website addresses thoracic trauma, encompassing both penetrative injuries and BCT. Visually, several adverts appear as soon as the page loads. There is also a lack of colour and images, which makes the website unappealing to read. This is coupled with large amounts of text, and long paragraphs. While the information is accurate regarding the injuries and treatment of chest trauma, it needs to be more condensed and contain less medical jargon.
27. European Society of Thoracic Surgeons	https://www.ests.org/about_ests/patient_information/diseases/chest_trauma.aspx#googtrans/en/en	This website has information contained across several pages. Information is not solely BCT related, however a large amount is relevant. The layout of the page is clear, with no adverts and good use of white space. Key pieces of information are highlighted using bullet-points. Some of the language used is too advanced for use by the public, and there is a lack of images giving a visual representation of the information in the text. There are links to further information.
28. Emedicinehealth	https://www.emedicinehealth.com/wilderness_chest_injuries/article_em.htm	The emedicinehealth website contains information on chest injuries of all description, but specifically discusses BCT causes and injuries. The layout of the page is good, with white space and sub-headings used effectively to allow easy navigation of the text. Bullet-points are used to highlight key pieces of information, and pictures complement the text. The language used is appropriate, and there are links to access further information. However, there are some adverts present that may distract the reader.
29. Radiology key	https://radiologykey.com/blunt-chest-trauma/	This BCT resource is laid out very simply, with no adverts or pop-ups. The information contained is however too advanced, due to the use of medical jargon and images of x-rays and CT scans that would only be helpful to a medical professional. The layout of the text is also poor, with long sentences and paragraphs. There is no easy way to navigate through the website, due to the length of the text and lack of links to specific sections.
30. Nurseinfo.in	https://nurseinfo.in/chest-injuries/	This website is aimed at nurses. It focuses on how best to diagnose and treat chest injuries, including those from BCT. It contains some information that would be useful to the public, however too much medical jargon is used when describing these. The text is well laid out, with good use of sub-headings and bullet-points to highlight key information in each section. No images are used to aid the text, and there are no links to any further information. The layout of the page is cluttered, with several adverts placed throughout the text.

For peer review only - http://bmjopen.bmj.com/site/about/guidelines.xhtml

Table 2. Visual assessment of the 30 identified websites.

Visual Assessment Criteria	Website number																													
	1	2	3	4	5	6	7	8	9	10	11	12	13	14	13	16	17	18	19	20	21	22	23	24	25	26	27	28	29	30
Information is clear, relevant and up to date	1	1	1	1	1	1	1	1	1	1	0	1	1	0	1	0	1	1	1	0	1	1	1	1	1	1	1	1	1	1
Text size is appropriate, and in a clear font	1	1	1	1	0	1	1	1	1	1	1	1	1	1	1	1	1	0	1	0	1	1	1	0	1	1	1	1	0	1
Images/Diagrams are appropriate and aid text	0	1	0	0	1	0	1	1	0	0	0	1	0	1	0	0	1	0	0	1	1	1	0	0	0	0	0	1	0	0
Headings and sub-headings are used effectively	1	1	1	1	0	0	1	1	1	1	1	1	1	1	1	0	1	0	1	1	1	1	1	1	1	0	1	1	0	1
Good use of white space and paragraphing	1	1	0	1	0	0	1	1	1	1	1	0	1	0	1	0	1	0	1	0	0	0	1	1	1	0	1	1	0	1
At first glance, website is clear and visually appealing	1	1	0	1	0	1	1	1	0	1	0	1	0	0	0	0	1	1	1	0	1	1	0	1	1	0	1	0	0	0
Good use of links to further information	1	0	1	1	1	1	1	0	0	0	1	1	0	1	1	1	1	1	1	1	1	0	0	0	0	0	1	1	0	0
Key information is highlighted or bullet-pointed	1	1	1	1	0	1	1	1	1	1	1	0	1	0	1	0	1	0	0	1	0	1	1	1	1	0	1	1	0	1
Language used is appropriate for the public	0	0	0	1	0	0	0	0	1	1	1	0	0	1	0	0	0	0	1	0	1	0	0	1	1	0	0	1	0	0
The public is clearly the focus group of the website	0	1	0	1	1	0	1	1	1	1	1	0	1	1	1	0	1	0	1	1	1	1	0	0	1	0	0	1	0	0
**Total Score**	7	8	5	9	4	5	9	8	7	8	7	5	6	6	7	2	9	3	8	5	8	7	5	6	8	2	7	9	1	5

For peer review only - http://bmjopen.bmj.com/site/about/guidelines.xhtml

Table 3. Readability assessment of the 30 websites identified on Google™, Yahoo™ and Bing™.

**Readability Assessments**	**Website**														
	Medscape			North Bristol NHS Trust			UpToDate			Health Direct			Wikipedia		
	Grade (US)	Grade (UK)	Age	Grade (US)	Grade (UK)	Age	Grade (US)	Grade (UK)	Age	Grade (US)	Grade (UK)	Age	Grade (US)	Grade (UK)	Age
Flesch-Kincaid	13.3	14.3	18+	5.1	6.1	10-11	15.2	16.2	18+	6.8	7.8	11-12	12.9	13.9	17-18
Gunning Fog	16.4	17.4	18+	7.3	8.3	12-13	17.8	18.8	18+	10.1	11.1	15-16	16.5	17.5	18+
Coleman-Liau	14.9	15.9	18+	7.3	8.3	12-13	16.4	17.4	18+	8.8	9.8	13-14	13.0	14.0	18+
SMOG	15.2	16.2	18+	8.6	9.6	13-14	16.7	17.7	18+	10.5	11.5	15-16	15.0	16.0	18+
Automated Readability	12.9	13.9	18+	4.0	5.0	12-13	15.0	16.0	18+	6.4	7.4	11-12	12.8	13.8	17-18
Median readability grade	14.4	15.4	18+	7.3	8.3	12-13	16.7	17.4	18+	8.8	9.8	13-14	13.0	14.0	18+
Flesch Reading Ease	28.9	Very difficult		70.5	Fairly easy		18.2	Very difficult		68.5	Plain English		37.7	Difficult	
% of general public readable to	60%			100%			46%			100%			63%		
**Readability Assessments**	**Website**														
	Science Direct Topics			MSD Manual			After Trauma			MyHealth.Alberta			Impact Medical Group		
	Grade (US)	Grade (UK)	Age	Grade (US)	Grade (UK)	Age	Grade (US)	Grade (UK)	Age	Grade (US)	Grade (UK)	Age	Grade (US)	Grade (UK)	Age
Flesch-Kincaid	12.7	13.7	17-18	9.7	10.7	14-15	7.1	8.1	12-13	4.7	5.7	9-10	8.5	9.5	13-14
Gunning Fog	14.9	15.9	18+	11.5	12.5	16-17	9.2	10.2	14-15	7.3	8.3	12-13	11.1	12.1	16-17
Coleman-Liau	15.0	16.0	18+	11.8	12.8	16-17	10.3	11.3	15-16	6.7	7.7	11-12	10.9	11.9	15-16
SMOG	14.3	15.3	18+	12.2	13.2	17-18	10.4	11.4	15-16	8.3	9.3	13-14	12.0	13.0	17-18
Automated Readability	11.8	12.8	16-17	9.1	10.1	14-15	7.2	8.2	12-13	4.5	5.5	9-10	8.6	9.6	13-14
Median readability grade	14.3	15.3	18+	11.5	12.5	16-17	9.2	10.2	14-15	6.7	7.7	11-12	10.9	11.9	15-16
Flesch Reading Ease	28.4	Very difficult		49.5	Difficult		64.6	Plain English		81.9	Easy		58.5	Fairly difficult	
% of general public readable to	65%			87%			100%			100%			100%		
**Readability Assessments**	**Website**														
	Healthlink			Osmosis			Drugs.com			VeryWellHealth			Healthline		
	Grade (US)	Grade (UK)	Age	Grade (US)	Grade (UK)	Age	Grade (US)	Grade (UK)	Age	Grade (US)	Grade (UK)	Age	Grade (US)	Grade (UK)	Age
Flesch-Kincaid	6.0	7.0	11-12	11.6	12.6	16-17	5.2	6.2	10-11	4.6	5.6	9-10	7.3	8.3	12-13
Gunning Fog	8.3	9.3	13-14	14.9	15.9	18+	8.1	9.1	13-14	6.4	7.4	11-12	10.3	11.3	15-16
Coleman-Liau	6.6	7.6	11-12	11.8	12.8	16-17	8.2	9.2	13-14	7.2	8.2	12-12	10.1	11.1	15-16

For peer review only - http://bmjopen.bmj.com/site/about/guidelines.xhtml

SMOG	8.9	9.9	13-14	14.1	15.1	18+	9.1	10.1	14-15	8.1	9.1	13-14	10.4	11.4	15-16
Automated Readability	5.1	6.1	10-11	11.0	12.0	16-17	5.1	6.1	10-11	4.5	5.5	9-10	6.7	7.7	11-12
Median readability grade	8.3	9.3	13-14	11.8	12.8	16-17	8.1	9.1	13-14	6.4	7.4	11-12	10.1	11.1	15-16
Flesch Reading Ease	76.5	Fairly easy		51.08	Fairly difficult		75.3	Fairly easy		80.9	Easy		60.9	Plain English	
% of general public readable to	100%			85%			100%			100%			100%		
**Readability Assessments**	**Websites**														
	Britannica			MedicalNewsToday			Trauma Victoria			National Institute for Health and Care Excellence (NICE)			SteadyHealth.com		
	Grade (US)	Grade (UK)	Age	Grade (US)	Grade (UK)	Age	Grade (US)	Grade (UK)	Age	Grade (US)	Grade (UK)	Age	Grade (US)	Grade (UK)	Age
Flesch-Kincaid	10.5	11.5	15-16	8.2	9.2	13-14	13.5	14.5	18+	8.3	9.3	13-14	13.3	14.3	18+
Gunning Fog	12.8	13.8	17-18	11.0	12.0	16-17	16.3	17.3	18+	11.9	12.9	16-17	15.4	16.4	18+
Coleman-Liau	12.7	13.7	17-18	10.3	11.3	15-16	15.2	16.2	18+	8.7	9.7	13-14	12.4	13.4	18+
SMOG	13.5	14.5	18+	11.4	12.4	16-17	15.3	16.3	18+	9.0	10.0	14-15	14.1	15.1	18+
Automated Readability	11.2	12.3	16-17	8.1	9.1	13-14	13.4	14.4	18+	8.8	9.8	13-14	11.1	12.1	18+
Median readability grade	12.7	13.7	17-18	10.3	11.3	15-16	15.2	16.2	18+	8.8	9.8	13-14	13.3	14.3	18+
Flesch Reading Ease	46.9	Difficult		60.7	Plain English		28.5	Very difficult		69.6	Plain English		42.0	Difficult	
% of general public readable to	81%			99%			59%			98%			60%		
**Readability Assessments**	**Websites**														
	AICA orthopaedics			Boohoff Law			LifeInTheFastlane			Firstresponse			Oxford University Hospitals NHS Trust		
	Grade (US)	Grade (UK)	Age	Grade (US)	Grade (UK)	Age	Grade (US)	Grade (UK)	Age	Grade (US)	Grade (UK)	Age	Grade (US)	Grade (UK)	Age
Flesch-Kincaid	9.4	10.4	14-15	11.0	12.0	16-17	12.0	13.0	17-18	8.0	9.0	13-14	6.1	7.1	11-12
Gunning Fog	12.2	13.2	17-18	14.3	15.3	18+	13.2	14.2	18+	9.5	10.5	14-15	8.3	9.3	13-14
Coleman-Liau	9.3	10.3	14-15	11.4	12.4	16-17	15.4	16.4	18+	10.0	11.0	15-16	8.6	9.6	13-14
SMOG	12.0	13.0	17-18	13.9	14.9	18+	12.4	13.4	17-18	10.7	11.7	15-17	9.6	10.6	14-15
Automated Readability	9.6	10.6	14-15	10.7	11.7	15-16	10.6	11.06	15-16	6.2	7.2	11-12	5.8	6.8	10-11
Median readability grade	9.6	10.6	14-15	11.4	12.4	16-17	12.4	13.4	17-18	9.5	10.5	14-15	8.3	9.3	13-14
Flesch Reading Ease	61.8	Plain English		48.0	Difficult		24.0	Very difficult		52.7	Fairly difficult		71.1	Fairly easy	
% of general public readable to	90%			78%			70%			100%			100%		

For peer review only

For peer review only - http://bmjopen.bmj.com/site/about/guidelines.xhtml

**Readability Assessments**	**Website**														
	EMS World			European Society of Thoracic Surgeons			Emedicinehealth			Radiology key			Nurseinfo.in		
	Grade (US)	Grade (UK)	Age	Grade (US)	Grade (UK)	Age	Grade (US)	Grade (UK)	Age	Grade (US)	Grade (UK)	Age	Grade (US)	Grade (UK)	Age
Flesch-Kincaid	13.8	14.8	18+	9.4	10.4	14-15	6.7	7.7	11-12	12.5	13.5	17-18	12.2	13.2	17-18
Gunning Fog	16.7	17.7	18+	11.8	12.8	16-17	8.9	9.9	13-14	15.4	16.4	18+	13.1	14.1	18+
Coleman-Liau	15.1	16.1	18+	12.9	13.9	17-18	8.9	9.9	13-14	14.1	15.1	18+	15.7	16.7	18+
SMOG	15.9	16.9	18+	11.6	12.6	16-17	10.2	11.2	15-16	14.4	15.4	18+	12.3	13.3	17-18
Automated Readability	14.3	15.3	18+	8.6	9.6	13-14	5.6	6.6	10-11	11.8	12.8	16-17	10.7	11.7	15.16
Median readability grade	15.1	16.1	18+	11.6	12.6	16-17	8.9	9.9	13-14	14.1	15.1	18+	12.3	13.3	17-18
Flesch Reading Ease	28.5	Very difficult		42.9	Difficult		64.0	Plain English		33.0	Difficult		21.5	Very difficult	
% of general public readable to	57%			90%			100%			66%			69%		

For peer review only - http://bmjopen.bmj.com/site/about/guidelines.xhtml

Figure 1. Comparison of Visual Assessment and Readability scores for each website

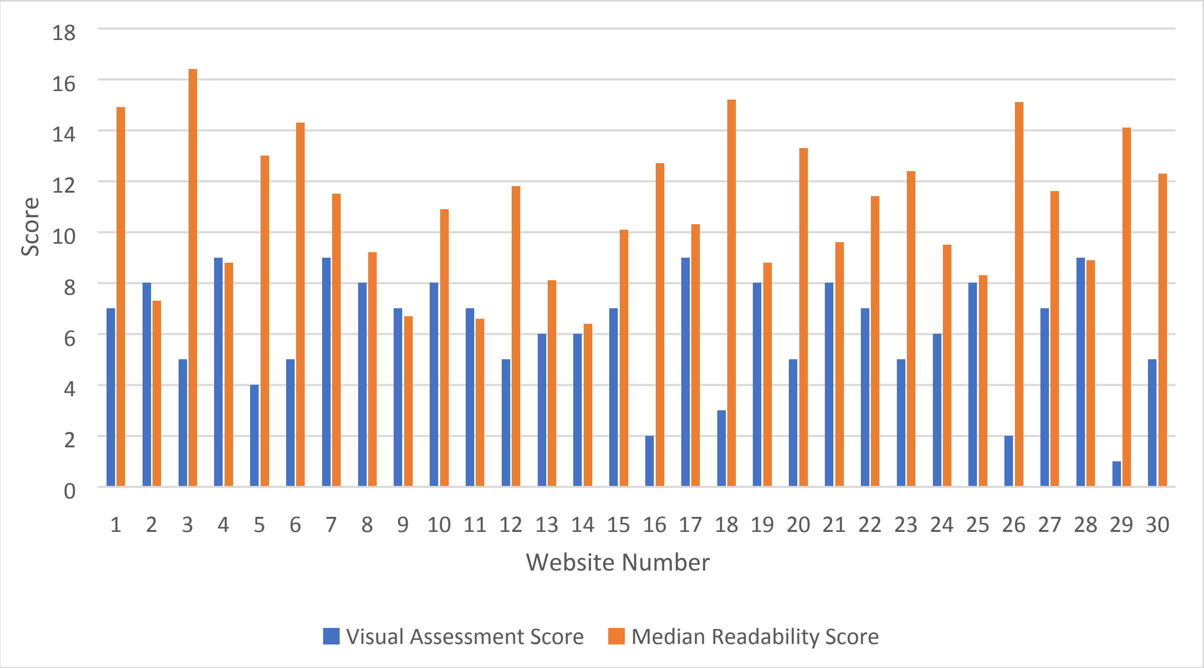

BMJ Open

A study to evaluate the readability and visual appearance of online resources for blunt chest trauma: an evaluation of online resources using mixed methods

Journal:	*BMJ Open*
Manuscript ID	bmjopen-2023-078552.R1
Article Type:	Original research
Date Submitted by the Author:	27-Nov-2023
Complete List of Authors:	Hutchings, Hayley; Swansea University, School of Medicine<br>Cochrane, Max; Swansea University, School of Medicine<br>Battle, Ceri; Swansea University, School of Medicine; Swansea Bay University Health Board, Physiotherapy Department
**Primary Subject Heading**:	Emergency medicine
Secondary Subject Heading:	Health services research
Keywords:	World Wide Web technology < BIOTECHNOLOGY & BIOINFORMATICS, Health Literacy, TRAUMA MANAGEMENT

SCHOLARONE™
Manuscripts

I, the Submitting Author has the right to grant and does grant on behalf of all authors of the Work (as defined in the below author licence), an exclusive licence and/or a non-exclusive licence for contributions from authors who are: i) UK Crown employees; ii) where BMJ has agreed a CC-BY licence shall apply, and/or iii) in accordance with the terms applicable for US Federal Government officers or employees acting as part of their official duties; on a worldwide, perpetual, irrevocable, royalty-free basis to BMJ Publishing Group Ltd ("BMJ") its licensees and where the relevant Journal is co-owned by BMJ to the co-owners of the Journal, to publish the Work in this journal and any other BMJ products and to exploit all rights, as set out in our licence.

The Submitting Author accepts and understands that any supply made under these terms is made by BMJ to the Submitting Author unless you are acting as an employee on behalf of your employer or a postgraduate student of an affiliated institution which is paying any applicable article publishing charge ("APC") for Open Access articles. Where the Submitting Author wishes to make the Work available on an Open Access basis (and intends to pay the relevant APC), the terms of reuse of such Open Access shall be governed by a Creative Commons licence – details of these licences and which Creative Commons licence will apply to this Work are set out in our licence referred to above.

Other than as permitted in any relevant BMJ Author's Self Archiving Policies, I confirm this Work has not been accepted for publication elsewhere, is not being considered for publication elsewhere and does not duplicate material already published. I confirm all authors consent to publication of this Work and authorise the granting of this licence.

For peer review only

A study to evaluate the readability and visual appearance of online resources for blunt chest trauma: an evaluation of online resources using mixed methods

Hayley A Hutchings[1*], Max Cochrane[1], Ceri E Battle[1,2].

[1]**Hayley Anne Hutchings**

https://orcid.org/0000-0003-4155-1741

Telephone: +44 (0) 1792 513412

Email: h.a.hutchings@swansea.ac.uk

[1]School of Medicine, Faculty of Medicine Health and Life Science, Singleton Park, Swansea SA2 8PP, U.K.

*Corresponding author

[1]**Max Cochrane**

Email: cochranemax1@gmail.com

[1]School of Medicine, Faculty of Medicine Health and Life Science, Singleton Park, Swansea SA2 8PP, U.K.

[1,2]**Ceri Elisabeth Battle**

Email: Ceri.Battle@wales.nhs.uk

https://orcid.org/0000-0002-7503-1931.

[2]Physiotherapy Department, Morriston Hospital, Swansea Bay University Health Board, Swansea, U.K.

For peer review only - http://bmjopen.bmj.com/site/about/guidelines.xhtml

Abstract

Objectives

Blunt chest trauma (BCT) is characterised by forceful and non-penetrative impact to the chest region. Increased access to the internet has led to online healthcare resources becoming used by the public to educate themselves about medical conditions. This study aimed to determine whether online resources for BCT are at an appropriate readability level and visual appearance for the public.

Design

We undertook a 1) a narrative overview assessment of the website; 2) a visual assessment of the identified website material content using an adapted framework of pre-determined key criteria based on the Centers for Medicare and Medicaid Services toolkit; and 3) a readability assessment utilising five readability scores and the Flesh reading ease score using Readable software.

Data Sources

Using a range of key search terms, we searched Google™, Bing™ and Yahoo™ websites on 9 October 2023 for online resources about BCT.

Results

We identified and assessed 85 websites. The median visual assessment score for the identified websites was 22, with a range of -14 to 37. The median readability score generated was 9 (14-15 years), with a range of 4.9 to 15.8. There was a significant association between the visual assessment and readability scores with a tendency for websites with lower readability scores having higher scores for the visual assessment (Spearman's rho= -0.485; $p<0.01$). The median score for Flesch reading ease was 63.9 (plain English) with a range of 21.1 to 85.3.

Conclusions

Although the readability levels and visual appearance was acceptable for the public for many websites, many of the resources had much higher readability scores than the recommended level (8-10) and visually were poor.

For peer review only - http://bmjopen.bmj.com/site/about/guidelines.xhtml

Better use of images would improve the appearance of websites further. Less medical terminology and shorter word and sentence length would also allow the public to comprehend the contained information more easily.

Strengths and Limitations

- We undertook a rigorous assessment of identified websites containing information on blunt chest trauma.
- We calculated extensive readability statistics for each website using five different readability scores and the Flesch reading ease score.
- We supplemented the readability assessment with a visual assessment using defined criteria adapted from the Centers for Medicare and Medicaid Services toolkit.
- We searched for relevant website materials using three search engines and restricted assessment to those websites identified on the first search page, therefore findings are limited to resources identified using these search engines.

Key words: world wide web technology, health literacy, trauma management

Funding Statement

This research received no specific grant from any funding agency in the public, commercial or not-for-profits sectors.

Conflicts of Interest

All authors confirm that there are no conflicts of interest to declare.

For peer review only - http://bmjopen.bmj.com/site/about/guidelines.xhtml

Introduction

Blunt chest trauma (BCT) is the leading cause of death among young adults, aged 15-44, and is second only to head trauma as the most common cause of death for all age groups[1]. The UK major trauma population is now reported to be more elderly, and the predominant mechanism is a fall from less than two metres[2].

Since the start of the century, internet usage across the globe has increased by 1,355%, with more than 5 billion internet users in the world as of 2023[3]. This huge increase in access to the internet has led to people using it as a healthcare resource, with 61% of Americans having looked up health-related information[4]. A study found that 62% of people considered healthcare information on the internet to be “excellent” or “very good”, and over half of people in the study felt they did not feel the need to share their findings with a doctor[5]. This suggests that many people see the internet as a valuable and accurate tool for healthcare. This number is likely to keep growing and this reliance on internet resources for all forms of healthcare means it is essential that information is accessible, accurate and readable.

Health literacy is defined as ‘a person’s ability to understand and use information to make decisions about their health’ and is believed to have a vital impact on public health due to access and use of health services[6, 7]. Between 43 and 61% of adults in the UK do not routinely understand health information[8], with more than 7 million adults having ‘very poor literacy skills’[9]. This means that they experience difficulties reading from unfamiliar sources. Similarly in the USA, the reading level of the average person is at an 8th grade level (age 12-13 years), meaning that they cannot interpret and evaluate any information that requires inference[10].

Low health literacy levels are a potential barrier to the use of online health resources. If the information is not able to be understood by a person without clinical knowledge, then this could

For peer review only - http://bmjopen.bmj.com/site/about/guidelines.xhtml

hinder early diagnosis and treatment of potentially life-threatening conditions. For this reason, it is essential that online resources are written at a low readability level, meaning that they are simple to interpret and understand.

Previous studies have found that online resources for healthcare are at a high readability level and are of poor quality[11-14] [15], showing that they contain medical terminology and are not visually appealing. For BCT, a study assessing the readability of a trauma surgery website found that the readability level was too high, and therefore difficult to comprehend[16].

One of the authors (CEB) has developed a predictive risk tool to help manage patients with BCT[17, 18] with one of the management pathways being to be sent home. It is important that patients understand any materials they are given. It is likely that such patients will access more materials online and as such we thought it was important to assess such materials. Apart from the study assessing the readability of the trauma surgery website, there has been no previous research undertaken assessing the quality and readability of online resources for BCT. The aim of this study was therefore to carry out an assessment of the readability and visual appearance of online resources for BCT.

Methods

Identifying online resources

To identify relevant websites we used the three most popular search engines in the UK: Google™, Bing™ and Yahoo™[19]. In order to capture the most relevant websites, we used a range of search terms. These were based on terms used in the existing literature. In order to capture more colloquial terms that would be familiar to patients, we also consulted with clinical colleagues who manage patients with chest trauma. Table 1 presents details of the full list of search terms used.

For peer review only - http://bmjopen.bmj.com/site/about/guidelines.xhtml

We identified all the websites that appeared on the first page of each search engine. We undertook: 1) a narrative overview assessment; 2) a readability assessment; and 3) a visual assessment of each website,

In order to evaluate the accessibility of the identified materials in terms of readability and visual aesthetics of online resources for BCT, we aimed to identify relevant websites that contained information intended to educate the public. We therefore excluded: research publications; books/chapters; clinical guidelines; teaching resources; clinical case studies; risk calculators; conference papers; newspaper articles; legal support services; referral information for clinicians; radio transcripts; sites solely for advertisement only; and sites where contact information only was provided. We further excluded sites where the content was clearly not relevant to the condition; private websites with restricted access; and sites with only video resources. Scientific papers, teaching resources and clinical guidelines are not resources developed specifically to educate and inform the public about BCT, while websites that require payment receive low traffic, with previous research finding that 80% of people who encounter a paywall when looking for health information will choose to search elsewhere[3]. We included Wikipedia, despite the fact that its content can be changed and hence may not contain accurate information[20]. We decided to include it as it is one of the most commonly accessed websites (currently seventh) and therefore it is important to assess its quality, due to the high level of traffic it receives[20, 21].

Narrative overview assessment We firstly produced a narrative overview assessment of each of the identified websites. We produced a paragraph that summarised our initial impressions of the

For peer review only - http://bmjopen.bmj.com/site/about/guidelines.xhtml

website outlining areas that were good or poor, prior to undertaking more detailed visual and readability assessments.

Visual assessment

We utilised the 'Guidelines for effective writing'[22], which details key areas that may affect the readability of websites in order to visually assess each website. These included headings, content organisation and language use, text size and colour, use of white space, and illustrations (for full list, see online Supplementary information Table S1).

We scored the visual assessment for each website. We compiled a list of 42 criteria for the assessment based on the 'Guidelines for effective writing'[22] . We selected these criteria as we judged them to be the most important elements when assessing the written content and visual appearance of the webpage. For each of these 42 criteria we assigned a score of +1 point if the criterion was achieved, 0 if the criterion was not applicable and -1 point if the criterion was not achieved. These points were added to give a maximum cumulative score of 42 for each website.

Readability assessment

When assessing the readability of each website, we used the website Readable (https://readable.com) to generate several readability scores for each online BCT resource. To do this, we entered each page of text that was considered relevant to BCT into Readable. Readable then generated various readability statistics. All text that appeared on any relevant page was entered into readable, including headings and lists. However, all images, graphs and navigation areas were removed for the purposes of calculating readability. These were however considered as part of the visual assessment of the websites.

For peer review only - http://bmjopen.bmj.com/site/about/guidelines.xhtml

When analysing the content of our chosen websites, we assessed all the relevant pages, up to a maximum of 10 pages. We only included pages that specifically discussed BCT or related injuries and disregarded any that contained other healthcare information or contact details.

We used five different readability formulae to give a wide evaluation of each website. There is currently no consensus regarding which readability formula is the most appropriate for assessing patient materials and it is therefore recommended that more than one formula is used to assess such materials[23] Each formula assesses the text in a different way and includes items such as words, characters, and syllables[12]. Employing a range of scores therefore improves the validity of the results[23]. We used the readability formulae: Flesch-Kincaid Grade Index[24], Coleman-Liau Index[25], Simplified Measure of Gobbledygook Index[26], Gunning-Fog Index[27] and the Automated Readability Index[28]. We chose these readability formulae as they have previously been used to assess readability within the medical field[15, 16, 23, 29-31].

In addition to calculating the readability formulae, we also calculated the percentage of the population the text could be read by and the Flesch Reading Ease score[32]. The Flesch Reading Ease (FRE) score is the earliest of the commonly used tools to assess readability[23, 31] and gives a score on a scale ranging from 0–100. A score of 0 is classified as being unreadable and that of 100 the most readable[31, 32]. It is based on the average number of syllables per word and the average number of words per sentence. Content with a score of 70 is easy to read for most of the population. Text with shorter sentences and simpler words will have a higher score than text with longer sentences and more complex words[32] .

Readable generated scores as a school grade level, which was then translated into a corresponding age. This is the age that could be expected to comprehend the piece of writing. Various healthcare

For peer review only - http://bmjopen.bmj.com/site/about/guidelines.xhtml

organisations have recommended that readability levels should be between 6th and no more than 8th grade (age 11-14 years)[33-35].

Statistical analysis

The readability statistics were generated for each page of the website used. A median (range) readability score was calculated based on the five readability formulae. Where more than one page was assessed for a website, scores were aggregated to give a median score for each formula that was representative of the entire website.

We compared readability scores with the cumulative score from the visual assessment to determine if there was any correlation between readability and visual appearance, using non-parametric Spearman correlation. A p-value of less than 0.05 was regarded as being statistically significant[36].

Patient and Public Involvement

Due to the limited time available to complete this project and lack of funding, it was not possible to involve patients or the public in the design, or conduct, or reporting, or dissemination plans of our research.

Results

Identification of online resources

Using the search terms across the first page of the three search engines, we identified a total of 258 websites. We identified a large amount of repetition of websites and a large number of websites were excluded based on applying our pre-defined search criteria (see Figure 1). There were a large number of scientific papers identified, especially when using more clinical search terms such as ‘thoracic contusion’. When using search terms that were less scientific such as ‘bruised’ or ‘broken

For peer review only - http://bmjopen.bmj.com/site/about/guidelines.xhtml

ribs', more online resources that appeared that were more suitable for use by the public. After applying exclusions, we identified a total of 85 websites for further assessment.

Narrative overview assessment

Supplementary information Table S2 details the online resources identified, with a brief written assessment of their content. Many of the resources that we identified were not solely based on BCT. Instead, they contained sections that were relevant to BCT as well as information on either other types of chest injury, or trauma injuries affecting the head or abdomen. Similarly, some of the resources were focused on types of injury caused by BCT, such as pneumothorax, flail chest and rib fractures. Most of the information identified was contained within a single webpage. There was limited use of photographs and illustrations that could have potentially improved the visual aesthetics of the website and understanding of the information. Many of the websites linked to simple patient information leaflets that focused on advice for patients to help manage chest injuries/rib fractures/bruising with signposting for further help should they need it. Many sites linked to the National Health Services (NHS) (for example general practice surgeries and secondary care hospitals) simply replicated the information from the NHS site and added their own branding. Overall, there was very limited, patient focused information identified for BCT.

Visual assessment

Details of the visual assessment for each of the 85 online resources are shown in Supplementary information Table S1. There was a large amount of variability across the websites. The median visual assessment score was 22 with a range from -14 to 37 out of a maximum possible score of 42. More than 50% of the websites achieved a score of 22 or less (45/85 websites; 52.9%). This means that most websites either did not consider or scored negatively on almost half of the criteria identified as important based on the Centers for Medicare and Medicaid Services Toolkit for Making Written

For peer review only - http://bmjopen.bmj.com/site/about/guidelines.xhtml

Material Clear and Effective[22] . This indicates that most websites could do more to make their websites more visually appealing.

The lowest scoring resource was the ISK Institute website, with an overall score of -14from a possible 42. Other poorly scoring websites with a score of less than 10 were: World Rugby Passport (-3); GP notebook (6); Wikipedia (6); Physiocheck (7); Farrell Physiotherapy (8); Orthopaedics and Trauma London (8); and DynaMed (8). Poorly scoring websites appeared cluttered with too much complex information and extensive use of medical terminology, poor use of colouring and contrast, distracting adverts, and limited or inappropriate use of illustrations. Many of these websites seem to be advertising services and therefore appear less credible.

The highest scoring websites was the Royal Devon University Healthcare NHS Foundation Trust & Northern Devon Healthcare NHS Trust with 37. There were five other websites that scored highly on their visual assessment with a score of 32 or more (Cleveland Clinic (32); Dr Gallagher and Partners (32); Bradford Teaching Hospitals NHS Foundation Trust (34); healthinfo.org.nz (34); Agency for Clinical Innovation (36)). Highly scoring websites had a clean and uncluttered appearance with lots of white space, sparing use of colour and good contrast of colours, easy to understand information with limited use of medical terminology, no adverts, and appropriate use of illustrations that supported the text content. These websites largely were associated with credible organisations such as the NHS or other government bodies, which helps to reassure the public that the information is more credible.

Readability assessment

Supplementary information Table S3 provides detailed information on the readability assessment. Most websites only had one page of information on which to undertake a readability assessment.

For peer review only - http://bmjopen.bmj.com/site/about/guidelines.xhtml

The median readability score across all websites was 9 , which equates to a reading age of 14-15 years of age. The range of scores was from 4.9 (10-11 years of age) to 15.8 (18+ years of age, or University level). Aiming for a readability grade of a maximum of 8[33-35], only 30 of the 85 websites identified (35.3%) were at an appropriate reading level for the general public.

The median Flesch Reading Ease score was 63.9 (Plain English) with a range between 21.1 (very difficult) for the UpToDate website, to 85.3 (easy) for the Doncaster and Bassetlaw Teaching Hospitals website. This is understandable given that the UpToDate website uses more medical terminology and seems to be directed at clinicians, whereas the Doncaster and Bassetlaw Teaching Hospitals website is a resource created for patients/the public. Only two additional websites were also classified as easy to read with a Flesch Reading Ease Score of more than 80 (NHS (81.1); My Health Alberta (81.7)). Aiming for a Flesch reading ease score of greater than 70[32] only 29/85 (34.1%) were accessible for most of the population.

In terms of reach and the percentage of the addressable audience that each website resource was readable to, scores ranged from 50% for the website UpToDate, to 100%, which was scored by more over 60% (52/85) of the websites.

Comparison between visual assessment and readability scores

Figure 2 shows the median readability score and the visual assessment score for each online resource. There was a large amount of variation between the websites for both categories. Some websites performed well in the readability assessment but badly in the visual assessment and vice versa. There was a statistically significant association between the visual assessment and readability scores with a tendency for websites with lower readability scores (i.e. easier to read) having higher (i.e. better) scores for the visual assessment (Spearman's rho= -0.485; p<0.01). This suggests that

For peer review only - http://bmjopen.bmj.com/site/about/guidelines.xhtml

there is a correlation between websites being difficult to read and being visually unappealing. A low readability score and a high visual assessment score is most desirable.

Discussion

Overall, we found that online resources for BCT were generally visually appealing, but the content was written at a level too high for the public to understand. This shows resources available for public access are not providing accessible information. This may lead to a misunderstanding of the information being provided, and consequentially could result in slower diagnosis and greater risk of hospitalisation.

To be deemed readable for the general public, a readability grade score of between 6 and 8 should be achieved, which equates to a reading age of between 11 and 14 years. Eighth grade is the average reading age of an American adult[10], so if the readability score of online resources are any higher than this, the information contained in the online resource will be inaccessible to more than half of the general public. We calculated a median readability score for the identified BCT resources of 9 (age 14-15 years), which is higher than the recommended score of 8. Only 35% of the website had a readability of less than 8[34, 35], which suggests that online resources for BCT are not suitable for educating the public. This is supported by the fact that only three websites were classified as easy to read with a score of more than 80 when considering the Flesh Reading Ease score[32], with only 34% being accessible for most of the population.

Our findings concur with previous research assessing readability of online resources for other health conditions, such as phenylketonuria, fibroadenoma, otolaryngology and parathyroidectomy, which all identified that readability levels were too high[11-14]. This suggests that readability issues are common in online health resources, which could be a reason for up to 61% of UK adults not routinely understanding health information[8].

For peer review only - http://bmjopen.bmj.com/site/about/guidelines.xhtml

These finding suggest that it is essential for those writing online resources for BCT to consider their target audience, and to ensure that the content of the website is accessible and understandable for the public. Possible considerations to improve public accessibility include the use of shorter words and sentences and the use of less complex words and medical terminology throughout the text. Previous studies have demonstrated that shortening sentences to less than 15 words led to an improvement in readability[37]. In certain scenarios where using more complex terms is necessary, brief explanations or definitions can also be helpful to the reader. All these factors can contribute to the production of more readable online resources for BCT, leading to increased levels of education for the public regarding their health.

For the visual assessment of BCT resources, a median score of 22/42 was recorded and a large number of the websites were generally well laid out and visually appealing. However, with no website scoring 42 and with more than 50% of websites scoring less than 22 this indicates that there are still ways in which the visual appearance of these websites can be improved.

It is important for the online resource to be appealing as this is the first thing a user will see and may impact whether they choose to continue to read the information present on the webpage[38-40]. Even if a resource for BCT contains accurate and helpful information written at an appropriate readability level for a member of the public, not having a visually appealing page may cause the reader to look elsewhere.

In designing a website for BCT education, guidance can be taken from the 'guidelines for effective writing' from the Centers for Medicare and Medicaid Services[22]. This will provide guidance on how best to structure text, with the use of paragraphing and headings essential to break up text into more manageable sections for readers. Using bullet-points to highlight key information is also

For peer review only - http://bmjopen.bmj.com/site/about/guidelines.xhtml

beneficial to the reader, being clear and concise[41]. This is more appealing than having to search for information in a large block of text.

It is important to consider making information accessible to people who may have poor eyesight or have problems with reading. The use of images and audio aids can be effective in communicating BCT information to these audiences. The online resources assessed in this study demonstrated limited use of images or diagrams, suggesting that less importance is placed on visual aids compared to written information. Previous research has found that the use of audio and visual aids have a significant impact on learning[42]. This further highlights the importance of using these aids in future online resources for BCT.

Many websites only partially discussed BCT or focused more on injuries that could be caused by BCT. In addition, several websites were aimed at audiences with more advanced scientific knowledge, however this was not clearly identifiable when the website was first accessed. These issues could both lead to confusion for the reader and dissuade them from looking for online information for BCT in the future. Therefore, it is important for future online resources to not only be visually appealing and written at an appropriate level, but to be clearly identifiable as resources intended for use by the public.

There are some limitations to this study. We employed a range of search terms, including many that would be more familiar to the public. However, a larger range of search terms could have been used, which may have identified more online resources. A larger number of resources could also have been assessed. However, since the three search engines we chose to use receive over 95% of traffic, and that 95% of people do not go past the first page of results[43] we believe that we identified the websites that would receive almost all the traffic. We also identified a lot of replication in the 258 identified websites within the first page search, which is likely to increased

For peer review only - http://bmjopen.bmj.com/site/about/guidelines.xhtml

further past the first page of searches. Apart from assessing some criteria relating to content accuracy as part of the visual assessment (such as author, use of references and website updates), we did not undertake a full assessment of the accuracy of the website content. The purpose of the study was to undertake an initial assessment of the readability and visual appearance of online materials for access by members of the public/patients. It was beyond the scope of the study to assess website content and therefore further work is therefore needed to assess the accuracy of the materials.

When assessing readability and visual quality of the chosen resources, we only used 5 readability formulae, all of which assessed the readability based on sentence and word length and complexity. This did not consider other factors such as tables and diagrams that could also affect how easily a website can be read. Even though these were considered in the visual assessment, evaluating tables and figures only formed a small part of the visual assessment. A specific examination of visual and audio aids would be useful for future research to consider when assessing the quality of online resources.

Some websites with only a small amount of information may demonstrate an overly high readability score, and not be as representative of the overall readability of the website as those in which multiple pages of information were assessed. However, if someone is searching for BCT information, it is less important to them how readable other sections of the websites are.
Further research is necessary, assessing specific aspects of visual appearance such as images in detail. Deeper analysis into the accuracy of the scientific content of the websites could also be useful, and other online resources such as videos or audio content should be considered.

Conclusions

For peer review only - http://bmjopen.bmj.com/site/about/guidelines.xhtml

We found that online resources for BCT were written at a level too advanced for use by the public, with a reading age greater than recommended. The visual appearance of these resources was generally at a level acceptable to the public. BCT online resources could however be made more accessible and improved for public use by reducing the reading age of the textual content, and by considering additional criteria to improve visual aesthetics, such as the use of images

Total word count: 3957

Ethics Statement

Patient Consent for Publication

Not applicable

Ethics Approval

Not applicable.

Author Contributions

HAH designed the original study, quality assured the data collection and analysis, provided supervisory support and drafted the manuscript. MC adapted the study design, undertook the data searches, analysed the data, drafted data outputs and reviewed the final manuscript. CEB provided input into study design, provided clinical advisory support to the project, quality assured the outputs and reviewed the final manuscript.

Data availability

All original data from the study are available in the zenodo repository[36].

For peer review only - http://bmjopen.bmj.com/site/about/guidelines.xhtml

References

1. Fantus RJ, Clark DE. NTDB(R) data points: Annual report 2007: more than 2.7 in 2007. Bull Am Coll Surg. 2008;93(1):62-3. PubMed PMID: 23505678.
2. Kehoe A, Smith JE, Edwards A, Yates D, Lecky F. The changing face of major trauma in the UK. Emerg Med J. 2015;32(12):911-5. doi: 10.1136/emermed-2015-205265. PubMed PMID: 26598629; PubMed Central PMCID: PMCPMC4717354.
3. Broadband Search. Key internet statistics in 2023 (including mobile). 2023 [28 July 2023]. Available from: https://www.broadbandsearch.net/blog/internet-statistics
4. Fox S, Jones S. The Social Life of Health Information. Americans' pursuit of health takes place within a widening network of both online and offline sources Washington: Pew Internet and American Life Project; 2009 [28 July 2023]. Available from: https://www.pewresearch.org/internet/2009/06/11/the-social-life-of-health-information/
5. Diaz JA, Griffith RA, Ng JJ, Reinert SE, Friedmann PD, Moulton AW. Patients' use of the Internet for medical information. J Gen Intern Med. 2002;17(3):180-5. doi: 10.1046/j.1525-1497.2002.10603.x. PubMed PMID: 11929503; PubMed Central PMCID: PMCPMC1495021.
6. NHS Digital Service Manual. Design and build digital services for the NHS. 2023 [28 July 2023]. Available from: https://service-manual.nhs.uk.
7. Vandenbosch J, Van den Broucke S, Vancorenland S, Avalosse H, Verniest R, Callens M. Health literacy and the use of healthcare services in Belgium. J Epidemiol Community Health. 2016;70(10):1032-8. Epub 20160426. doi: 10.1136/jech-2015-206910. PubMed PMID: 27116951.
8. Berry J. Does health literacy matter? : NHS England; 2016 [28 July 2023]. Available from: https://www.england.nhs.uk/blog/jonathan-berry/.
9. National Literacy Trust. What is Literacy? [28 July 2023]. Available from: https://literacytrust.org.uk/information/what-is-literacy/
10. National Centre for Education Statistics. Program for the International Assessment of Adult Competencies (PIAAC) 2023 [28 July 2023]. Available from: https://nces.ed.gov/surveys/piaac/
11. Byrne J, Keogh S, Cullinane C, Razzaq Z, Redmond HP. Readability and Quality of Online Health Information Regarding Parathyroidectomy. OTO Open. 2022;6(4):2473974X221133308. Epub 20221020. doi: 10.1177/2473974X221133308. PubMed PMID: 36311181; PubMed Central PMCID: PMCPMC9597036.
12. Hutchings HA, Remesh A. An evaluation of the readability and visual appearance of online patient resources for fibroadenoma. PLoS One. 2022;17(11):e0277823. Epub 20221118. doi: 10.1371/journal.pone.0277823. PubMed PMID: 36399450; PubMed Central PMCID: PMCPMC9674151.
13. Marsh JM, Dobbs TD, Hutchings HA. The readability of online health resources for phenylketonuria. J Community Genet. 2020;11(4):451-9. Epub 20200327. doi: 10.1007/s12687-020-00461-9. PubMed PMID: 32221843; PubMed Central PMCID: PMCPMC7475157.
14. Grose EM, Holmes CP, Aravinthan KA, Wu V, Lee JM. Readability and quality assessment of internet-based patient education materials related to nasal septoplasty. J Otolaryngol Head Neck Surg. 2021;50(1):16. Epub 20210317. doi: 10.1186/s40463-021-00507-z. PubMed PMID: 33731227; PubMed Central PMCID: PMCPMC7970817.
15. Martin CA, Khan S, Lee R, Do AT, Sridhar J, Crowell EL, et al. Readability and Suitability of Online Patient Education Materials for Glaucoma. Ophthalmol Glaucoma. 2022;5(5):525-30. Epub 20220314. doi: 10.1016/j.ogla.2022.03.004. PubMed PMID: 35301989.
16. Eltorai AE, Ghanian S, Adams CA, Jr., Born CT, Daniels AH. Readability of patient education materials on the american association for surgery of trauma website. Arch Trauma Res. 2014;3(2):e18161. Epub 20140430. doi: 10.5812/atr.18161. PubMed PMID: 25147778; PubMed Central PMCID: PMCPMC4139691.

For peer review only - http://bmjopen.bmj.com/site/about/guidelines.xhtml

17. Battle CE, Hutchings H, Evans PA. Risk factors that predict mortality in patients with blunt chest wall trauma: a systematic review and meta-analysis. Injury. 2012;43(1):8-17. Epub 20110122. doi: 10.1016/j.injury.2011.01.004. PubMed PMID: 21256488.
18. Battle C, Hutchings H, Lovett S, Bouamra O, Jones S, Sen A, et al. Predicting outcomes after blunt chest wall trauma: development and external validation of a new prognostic model. Crit Care. 2014;18(3):R98. Epub 20140514. doi: 10.1186/cc13873. PubMed PMID: 24887537; PubMed Central PMCID: PMCPMC4095687.
19. Search Engine Insight. Top 10 Search Engines In The World (2022 Update) [31 July 2023]. Available from: https://www.searchengineinsight.com/top-10-leading-search-engines-uk/.
20. Azer SA. Is Wikipedia a reliable learning resource for medical students? Evaluating respiratory topics. Adv Physiol Educ. 2015;39(1):5-14. doi: 10.1152/advan.00110.2014. PubMed PMID: 25727464.
21. Similarweb. Top Websites Ranking. Available from: https://www.similarweb.com/top-websites/ (Access date 20 November 2023) 2023.
22. CMS.gov, Centers for Medicare and Medicaid Services. Toolkit for Making Written Material Clear and Effective. [31 July 2023]. Available from: https://www.cms.gov/outreach-and-education/outreach/writtenmaterialstoolkit.
23. Badarudeen S, Sabharwal S. Assessing readability of patient education materials: current role in orthopaedics. Clin Orthop Relat Res. 2010;468(10):2572-80. doi: 10.1007/s11999-010-1380-y. PubMed PMID: 20496023; PubMed Central PMCID: PMCPMC3049622.
24. Kincaid JP, Fishburne RP, Rogers RL, Chissom BS. Derivation of new readability formulas (automated readability index, fog count, and flesch reading ease formula) for Navy enlisted personnel. Research Branch Report 8–75. Available from: https://apps.dtic.mil/dtic/tr/fulltext/u2/a006655.pdf (access date 22 January 2020). Naval Air Station Memphis: 1975.
25. Coleman M, Liau TL. A computer readability formula designed for machine scoring. Journal of Applied Psychology. 1975;60:283-4.
26. McLaughlin GH. SMOG Grading — a New Readability Formula. Journal of Reading. 1969;12(8):639-46.
27. Gunning R. The Technique of Clear Writing: McGraw-Hill; 1952.
28. Smith EA, Senter RJ. Automated Readability Index. AMRI.-TR-66-220. Available from: https://apps.dtic.mil/dtic/tr/fulltext/u2/667273.pdf (access date 13 January 2020). Defense Documentation Center, Virginia: Wright-Patterson Air Force Base, Ohio, 1967.
29. Dobbs T, Neal G, Hutchings HA, Whitaker IS, Milton J. The Readability of Online Patient Resources for Skin Cancer Treatment. Oncol Ther. 2017;5:149-60. doi: 10.1007/s40498-017-0051-5.
30. Phillips NA, Vargas CR, Chuang DJ, Lee BT. Readability Assessment of Online Patient Abdominoplasty Resources. Aesthetic Plast Surg. 2015;39(1):147-53. doi: 10.1007/s00266-014-0425-0. PubMed PMID: 25476602.
31. Friedman DB, Hoffman-Goetz L. A systematic review of readability and comprehension instruments used for print and web-based cancer information. Health Educ Behav. 2006;33(3):352-73. doi: 10.1177/1090198105277329. PubMed PMID: 16699125.
32. Interpretation of Flesch Reading Ease Score (Access date 16 August 2022). Available from: https://writingstudio.com/blog/flesch-reading-ease/.
33. Wolf MS, Cooper Bailey S. The Role of Health Literacy in Patient Safety. Available from: https://psnet.ahrq.gov/perspective/role-health-literacy-patient-safety (Acces date 20 November 2023): PS Net; 2009.
34. Cotugna N, Vickery CE, Carpenter-Haefele KM. Evaluation of literacy level of patient education pages in health-related journals. J Community Health. 2005;30(3):213-9. doi: 10.1007/s10900-004-1959-x. PubMed PMID: 15847246.
35. The Joint Commission. "What Did the Doctor Say?:" Improving Health Literacy to Protect Patient Safety. Available from:

For peer review only - http://bmjopen.bmj.com/site/about/guidelines.xhtml

https://swselfmanagement.ca/uploads/ResourceDocuments/The%20Joint%20Commission%20(2007)%20Improving%20Health%20Literacy%20for%20Patient%20Safety.pdf (Access date 20 November 2023). 2007.
[dataset]36. Hutchings HA, Cochrane M, Battle CE. A study to evaluate the readability and visual appearance of online resources for blunt chest trauma: an evaluation of online resources using mixed methods Available from: https://zenodo.org. 2023.
37. Sheppard ED, Hyde Z, Florence MN, McGwin G, Kirchner JS, Ponce BA. Improving the readability of online foot and ankle patient education materials. Foot Ankle Int. 2014;35(12):1282-6. Epub 20140919. doi: 10.1177/1071100714550650. PubMed PMID: 25239196.
38. Hesse BW, Moser RP, Rutten LJ. Surveys of physicians and electronic health information. N Engl J Med. 2010;362(9):859-60. doi: 10.1056/NEJMc0909595. PubMed PMID: 20200398.
39. Woloshin S, Yang Y, Fischhoff B. Communicating health information with visual displays. Nat Med. 2023;29(5):1085-91. Epub 20230508. doi: 10.1038/s41591-023-02328-1. PubMed PMID: 37156935.
40. Szmuda T, Ozdemir C, Ali S, Singh A, Syed MT, Sloniewski P. Readability of online patient education material for the novel coronavirus disease (COVID-19): a cross-sectional health literacy study. Public Health. 2020;185:21-5. Epub 20200530. doi: 10.1016/j.puhe.2020.05.041. PubMed PMID: 32516624; PubMed Central PMCID: PMCPMC7260546.
41. Gov.uk. Style guide [3 August 2023]. Available from: https://www.gov.uk/guidance/style-guide/a-to-z-of-gov-uk-style.
42. Carey M, Schofield P, Jefford M, Krishnasamy M, Aranda S. The development of audio-visual materials to prepare patients for medical procedures: an oncology application. Eur J Cancer Care (Engl). 2007;16(5):417-23. doi: 10.1111/j.1365-2354.2006.00772.x. PubMed PMID: 17760928.
43. Protofuse. The First Page Of Google, By The Numbers [3 August 2023]. Available from: https://protofuse.com/articles/first-page-of-google-stats/.

For peer review only

Figure 1. Flow diagram illustrating number of websites identified and reasons for exclusions.

Figure 2. Median readability score vs visual assessment score for the 85 assessed websites.

For peer review only

For peer review only - http://bmjopen.bmj.com/site/about/guidelines.xhtml

Tables

For peer review only

For peer review only - http://bmjopen.bmj.com/site/about/guidelines.xhtml

Table 1. List of search terms used to undertake searches in Google™, Bing™ and Yahoo™.

blunt chest trauma
blunt chest injury
chest impact injury
chest trauma
thoracic contusion
forceful chest injury
chest injury
broken ribs
rib fracture
chest and rib injury
chest wall injury
rib injuries
bruised ribs

For peer review only

For peer review only - http://bmjopen.bmj.com/site/about/guidelines.xhtml

Figure 1. Flow diagram illustrating number of websites identified and reasons for exclusions.

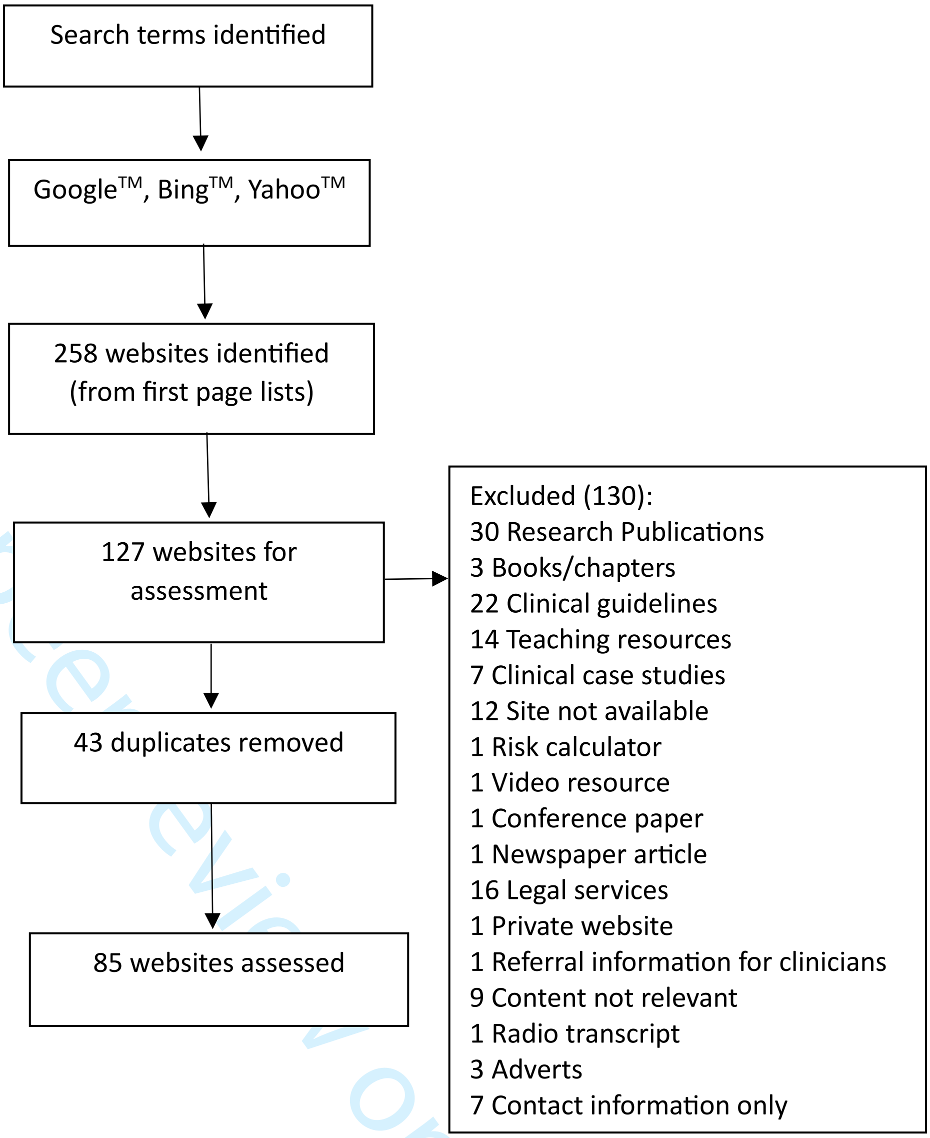

For peer review only

Figure 2. Median readability score vs visual assessment score for the 85 assessed websites.

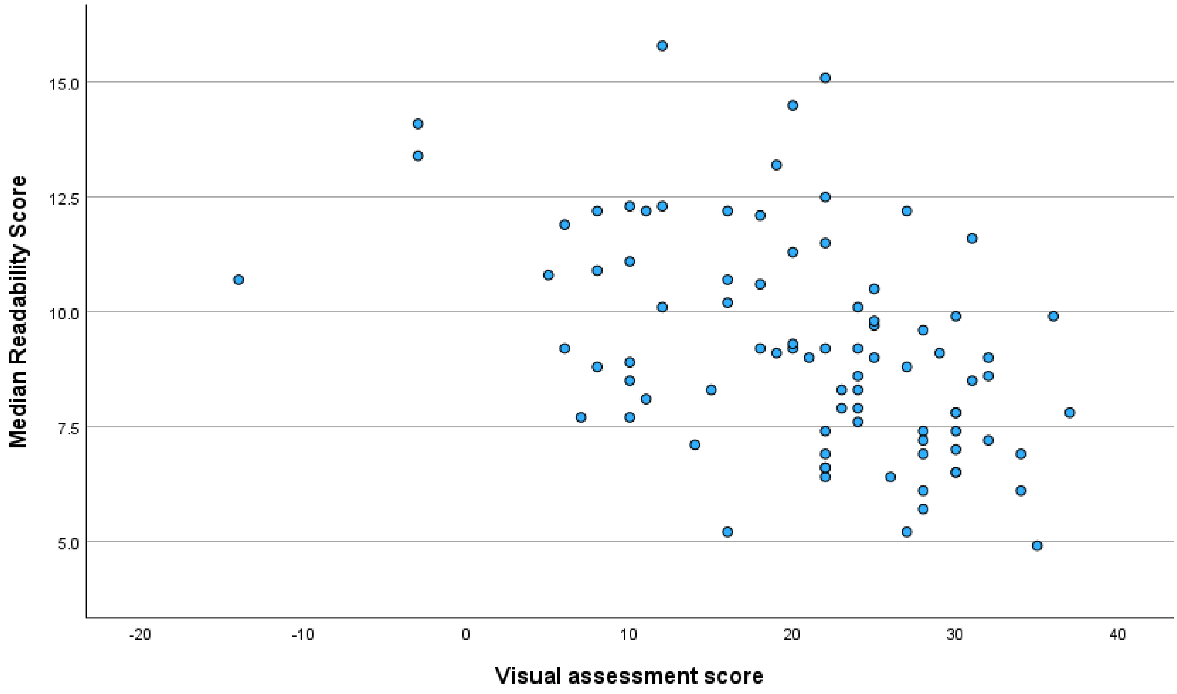

Spearman's rho= -0.485; p<0.01

For peer review only

Supplementary information. Table S1. Visual assessment of each website based on the Centers for Medicare and Medicaid Services Toolkit for Making Written Material Clear and Effective[22].

	Website																					
**Scoring Criteria**	**1**	**2**	**3**	**4**	**5**	**6**	**7**	**8**	**9**	**10**	**11**	**12**	**13**	**14**	**15**	**16**	**17**	**18**	**19**	**20**	**21**	**22**
Overall design and page layout	1	-1	1	1	-1	1	1	1	1	-1	1	-1	-1	1	1	1	1	1	-1	-1	1	1
The size, shape and general look of the material was designed with its purpose and users in mind	1	-1	1	1	1	1	-1	1	1	-1	1	-1	-1	1	1	1	1	1	1	-1	1	1
The material looks appealing at first glance	1	-1	1	1	-1	1	1	1	1	1	1	-1	-1	1	1	1	1	1	-1	1	1	1
A clear and obvious path has been created for the eye to follow	1	1	1	1	1	1	-1	1	1	-1	1	1	1	1	1	1	1	1	1	1	1	1
The material that has a clear and consistent style and structure	1	1	1	1	1	1	1	1	1	1	1	1	1	1	1	1	1	1	1	1	1	1
Fonts, size of print and contrast	1	1	1	1	1	1	1	1	1	1	1	1	-1	1	1	1	1	1	1	1	1	1
For the regular text, a font that is designed for ease of reading is used	1	1	1	1	1	1	1	1	1	1	1	1	-1	1	1	1	1	1	1	1	1	1
For headings, an easy-to-read font is used that contrasts with the main text	1	1	1	1	1	1	1	1	1	1	1	1	1	1	1	1	1	1	1	1	1	1
In general, no more than 2 or 3 different typefaces are used	1	1	1	-1	1	1	1	1	1	-1	1	1	1	1	1	1	-1	1	1	1	1	1
The font size is large enough for the intended audience	1	1	1	1	1	1	-1	1	1	-1	1	1	-1	1	1	1	1	1	1	1	1	1
Upper and lower case are used, not all capitals	1	1	1	1	1	1	1	1	1	1	1	-1	1	1	1	1	1	1	1	1	1	1
To emphasize words and phrases italics or bold text are used	1	1	1	-1	1	1	1	1	1	1	1	1	1	1	1	1	1	1	1	1	1	1
For ease of reading, dark-coloured text is used on a very light background	1	1	1	1	1	1	1	1	1	1	1	1	1	1	1	1	1	1	1	1	1	1
Text is not aligned sideways, on patterned or shaded background or on top of photos or other images	1	1	1	1	1	1	1	1	1	1	1	1	1	1	1	1	1	1	1	1	1	1
For ease of reading, extra line spacing has been added	1	1	1	1	1	1	1	1	1	-1	1	1	-1	1	1	1	-1	1	1	1	1	1
For ease of reading, left justification is used throughout	1	1	1	1	1	1	1	1	1	1	1	1	-1	1	1	1	-1	1	1	1	1	1
Lines of text are an appropriate length—neither too long nor too short	-1	-1	1	-1	1	1	1	1	1	-1	-1	-1	-1	1	1	1	1	-1	1	1	1	1
Hyphenation has been avoided at the end of lines	1	1	1	1	1	1	1	1	1	1	1	1	-1	1	1	1	1	1	1	1	1	1
Headings, bulleted lists, and blocks of text	1	1	1	1	1	1	1	1	1	1	-1	1	1	1	1	1	1	1	1	1	1	1
There is a clear hierarchy of prominent headings and sub-headings	1	1	1	1	1	1	1	1	1	1	1	1	1	1	1	1	-1	1	1	-1	1	1
Contrast is used to make the main points stand out	1	1	1	1	1	1	1	1	1	1	1	1	-1	1	1	1	1	1	1	1	1	1
Bulleted lists are well formatted	0	1	1	1	-1	1	1	1	0	1	0	1	0	1	1	1	1	1	1	1	1	1
Effective ways are used to emphasize important blocks of text	0	-1	1	-1	-1	1	1	1	1	1	1	1	1	1	1	1	-1	1	1	1	1	1
Use of colour	0	-1	1	1	-1	-1	-1	-1	1	1	-1	-1	-1	1	-1	-1	-1	1	-1	1	1	1
Colours used are appealing to the intended readers	0	-1	1	-1	-1	-1	-1	-1	1	-1	1	-1	-1	1	-1	-1	-1	-1	-1	-1	1	1
Colour is used sparingly and in a consistent and deliberate way	1	1	1	-1	-1	1	-1	-1	1	1	1	-1	-1	1	1	1	-1	1	1	1	1	1

For peer review only

Scoring Criteria	1	2	3	4	5	6	7	8	9	10	11	12	13	14	15	16	17	18	19	20	21	22
The colour scheme works from a design standpoint and when printed	1	1	1	-1	-1	1	-1	1	1	-1	1	1	-1	1	1	1	-1	1	1	-1	1	1
The colour scheme works for with diminished or limited colour perception	0	1	1	-1	-1	1	-1	1	1	-1	1	1	-1	1	1	1	-1	1	1	-1	1	1
Photographs and illustrations	-1	-1	-1	-1	1	-1	1	-1	1	1	-1	-1	1	-1	-1	-1	1	-1	-1	-1	-1	1
Photos and illustrations are used that relate directly to the information to reinforce key messages	0	0	0	-1	-1	0	-1	0	-1	-1	0	0	1	0	-1	0	1	0	0	-1	0	1
Images used are clear, uncluttered, and consistent in style	0	0	0	-1	-1	0	-1	0	-1	-1	-1	0	1	0	0	0	1	0	0	-1	0	1
Photos and illustrations used are culturally appropriate for the intended readers	0	0	0	-1	-1	0	1	0	0	-1	-1	0	1	0	0	0	1	0	0	-1	0	1
When images include people, they are appropriate to the situation and intended audience	0	0	0	0	0	0	0	0	0	0	0	0	-1	0	0	0	1	0	0	-1	0	1
Tables, charts, and diagrams	-1	-1	-1	1	-1	-1	1	-1	-1	-1	-1	-1	-1	-1	-1	-1	-1	-1	-1	-1	-1	1
Likely literacy levels of the reader have been considered in the use of tables, charts, and diagrams	-1	0	0	-1	0	0	0	0	1	0	-1	0	0	0	0	0	0	0	0	0	0	1
Titles, headings, and other labelling is specific and clear	1	1	0	-1	0	0	-1	1	1	1	1	1	1	1	1	1	-1	1	1	1	1	1
A clean, uncluttered layout is used with strong visual cures to guide the reader through the information	1	-1	1	1	-1	1	-1	1	-1	-1	1	-1	-1	1	1	1	-1	-1	1	-1	1	1
Numbers or calculations are carefully explained	0	0	0	0	0	0	0	0	0	0	0	0	0	0	0	0	0	0	0	0	0	0
General information about website	1	-1	1	1	-1	1	1	1	1	1	1	1	1	1	-1	1	1	1	1	1	-1	1
Last updated date given	1	1	1	-1	1	1	1	1	1	1	1	1	1	1	1	1	1	1	1	1	-1	1
Frequency of updates given	-1	-1	-1	-1	-1	1	-1	-1	-1	-1	-1	-1	-1	-1	-1	-1	-1	-1	-1	-1	-1	-1
Relevant references given	1	1	-1	1	1	-1	-1	-1	-1	-1	1	-1	-1	-1	-1	-1	-1	-1	-1	-1	-1	-1
**Overall assessment score**	22	12	27	8	6	25	11	22	26	5	20	10	-3	28	21	24	10	22	20	10	24	37

(+1 point if the statement was achieved, 0 if the statement was not applicable and -1 point if the statement was not achieved.)

	Website																					
**Scoring Criteria**	**23**	**24**	**25**	**26**	**27**	**28**	**29**	**30**	**31**	**32**	**33**	**34**	**35**	**36**	**37**	**38**	**39**	**40**	**41**	**42**	**43**	**44**
Overall design and page layout	1	1	1	1	1	-1	1	-1	1	-1	-1	1	1	-1	1	1	1	1	1	1	1	1
The size, shape and general look of the material was designed with its purpose and users in mind	1	1	1	1	1	1	1	-1	1	1	-1	1	1	-1	1	1	-1	1	1	1	1	1
The material looks appealing at first glance	1	1	1	1	1	-1	1	-1	1	1	-1	1	1	-1	1	1	1	1	-1	-1	-1	1
A clear and obvious path has been created for the eye to follow	1	1	-1	1	-1	-1	1	-1	1	1	1	1	1	1	1	1	1	1	-1	-1	-1	1
The material that has a clear and consistent style and structure	1	1	1	1	1	1	1	1	1	1	1	1	1	1	1	1	-1	1	1	1	1	1
Fonts, size of print and contrast	1	1	1	1	1	1	1	1	1	1	1	1	1	1	1	1	1	1	1	1	1	1
For the regular text, a font that is designed for ease of reading is used	1	1	1	1	1	1	1	1	1	1	1	1	1	1	1	1	1	1	1	1	1	1
For headings, an easy-to-read font is used that contrasts with the main text	1	1	1	1	1	1	1	1	1	1	1	1	1	1	1	1	1	1	1	1	1	1
In general, no more than 2 or 3 different typefaces are used	1	1	1	1	1	1	1	1	1	1	1	1	1	1	1	1	1	1	1	1	1	1
The font size is large enough for the intended audience	1	1	1	1	1	1	1	1	1	1	1	1	1	1	1	1	1	1	1	1	1	1
Upper and lower case are used, not all capitals	1	1	1	1	1	1	1	1	1	1	1	1	1	1	1	1	1	1	1	1	1	1
To emphasize words and phrases italics or bold text are used	1	1	1	1	1	1	1	1	1	1	1	1	1	1	1	1	1	1	1	1	1	1
For ease of reading, dark-coloured text is used on a very light background	1	1	1	1	1	1	1	1	1	1	-1	1	1	1	1	1	1	1	1	1	1	1
Text is not aligned sideways, on patterned or shaded background or on top of photos or other images	1	1	1	1	1	1	1	1	1	1	-1	1	1	1	1	1	1	1	1	1	1	1
For ease of reading, extra line spacing has been added	1	1	1	1	-1	1	1	1	1	1	-1	1	1	1	1	1	1	1	1	1	1	1
For ease of reading, left justification is used throughout	1	1	1	1	1	1	1	1	1	1	1	1	1	1	1	1	1	1	1	1	1	1
Lines of text are an appropriate length—neither too long nor too short	1	1	1	1	-1	1	1	1	-1	-1	-1	1	-1	1	1	1	-1	1	1	-1	-1	-1
Hyphenation has been avoided at the end of lines	1	1	1	1	1	1	1	1	1	1	1	1	1	1	1	1	1	1	1	1	1	1
Headings, bulleted lists, and blocks of text	1	1	1	1	1	1	1	1	1	1	1	1	1	1	1	1	1	1	1	1	1	1
There is a clear hierarchy of prominent headings and sub-headings	1	1	1	1	1	1	1	1	1	1	1	1	1	1	1	1	1	1	1	1	1	1
Contrast is used to make the main points stand out	1	1	1	1	1	1	-1	1	1	1	1	1	1	1	1	1	1	1	1	1	1	1
Bulleted lists are well formatted	1	1	1	1	0	1	1	1	1	1	1	1	1	1	1	1	1	1	1	1	1	1
Effective ways are used to emphasize important blocks of text	1	1	1	1	1	1	1	1	1	1	1	1	1	1	1	1	1	1	1	1	1	1
Use of colour	1	1	1	1	1	1	1	1	1	1	-1	1	-1	1	1	1	1	1	1	1	1	1
Colours used are appealing to the intended readers	1	1	1	1	-1	1	1	1	1	1	-1	1	1	1	1	1	1	1	1	1	1	1
Colour is used sparingly and in a consistent and deliberate way	1	1	1	1	-1	1	1	1	1	1	-1	1	1	1	1	1	1	1	1	1	1	-1
The colour scheme works from a design standpoint and when printed	-1	1	-1	1	-1	1	1	-1	1	1	-1	1	-1	1	1	1	1	1	1	1	1	-1

For peer review only

**Scoring Criteria**	**23**	**24**	**25**	**26**	**27**	**28**	**29**	**30**	**31**	**32**	**33**	**34**	**35**	**36**	**37**	**38**	**39**	**40**	**41**	**42**	**43**	**44**
The colour scheme works for with diminished or limited colour perception	1	1	-1	1	-1	1	1	-1	1	1	-1	1	-1	1	1	1	1	1	1	1	1	-1
Photographs and illustrations	1	-1	-1	-1	1	-1	1	-1	-1	1	-1	-1	1	-1	-1	-1	1	-1	-1	-1	1	1
Photos and illustrations are used that relate directly to the information to reinforce key messages	0	0	0	0	1	0	1	0	0	1	0	0	1	-1	0	0	-1	0	-1	-1	-1	-1
Images used are clear, uncluttered, and consistent in style	0	0	-1	0	1	0	1	0	0	1	0	0	1	-1	0	0	1	0	1	0	-1	-1
Photos and illustrations used are culturally appropriate for the intended readers	0	0	0	0	-1	0	1	0	0	1	0	0	1	-1	0	0	-1	0	-1	0	1	1
When images include people, they are appropriate to the situation and intended audience	0	0	0	0	0	0	0	0	0	0	0	0	1	-1	0	0	-1	0	1	0	1	0
Tables, charts, and diagrams	-1	-1	-1	-1	1	-1	-1	-1	-1	1	-1	-1	-1	-1	-1	-1	1	-1	-1	-1	-1	1
Likely literacy levels of the reader have been considered in the use of tables, charts, and diagrams	0	0	0	0	-1	0	1	0	0	1	-1	0	1	0	0	0	-1	0	0	0	0	-1
Titles, headings, and other labelling is specific and clear	1	1	1	1	1	1	1	1	1	1	1	1	1	1	1	0	1	1	1	1	1	1
A clean, uncluttered layout is used with strong visual cures to guide the reader through the information	1	1	-1	1	-1	-1	1	-1	1	-1	-1	1	1	-1	1	1	1	1	-1	1	-1	-1
Numbers or calculations are carefully explained	0	0	0	0	0	0	0	0	0	0	0	0	0	0	0	0	0	0	0	0	0	0
General information about website	1	1	-1	-1	-1	1	1	1	-1	1	-1	-1	1	1	1	-1	-1	1	-1	-1	1	-1
Last updated date given	1	1	-1	-1	-1	1	1	1	-1	1	-1	-1	1	1	1	-1	-1	1	-1	-1	1	1
Frequency of updates given	0	1	-1	-1	-1	-1	-1	-1	-1	-1	-1	-1	-1	-1	1	-1	-1	1	-1	-1	-1	-1
Relevant references given	-1	-1	-1	-1	-1	-1	-1	-1	-1	1	-1	-1	-1	-1	-1	-1	-1	-1	-1	1	-1	-1
**Overall assessment score**	29	30	15	24	11	20	32	14	22	32	-3	24	27	16	30	23	19	30	18	19	22	18

(+1 point if the statement was achieved, 0 if the statement was not applicable and -1 point if the statement was not achieved.)

For peer review only

	Website																					
**Scoring Criteria**	**45**	**46**	**47**	**48**	**49**	**50**	**51**	**52**	**53**	**54**	**55**	**56**	**57**	**58**	**59**	**60**	**61**	**62**	**63**	**64**	**65**	**66**
Overall design and page layout	1	1	1	1	1	1	1	1	1	1	1	-1	1	-1	-1	1	-1	-1	1	1	1	1
The size, shape and general look of the material was designed with its purpose and users in mind	1	1	1	1	1	1	1	1	1	1	1	1	1	-1	-1	1	1	-1	1	1	1	1
The material looks appealing at first glance	1	1	-1	-1	1	1	1	1	1	1	1	1	1	-1	-1	-1	-1	-1	1	1	1	-1
A clear and obvious path has been created for the eye to follow	1	1	1	1	1	1	1	1	1	1	1	-1	1	-1	-1	-1	-1	-1	1	1	1	-1
The material that has a clear and consistent style and structure	1	1	1	1	1	1	1	1	1	1	1	1	1	1	1	1	1	1	1	1	1	1
Fonts, size of print and contrast	1	1	1	1	1	1	1	1	1	1	1	1	1	1	1	1	1	1	1	1	-1	1
For the regular text, a font that is designed for ease of reading is used	1	1	1	1	1	1	1	1	1	1	1	1	1	1	1	1	1	1	1	1	1	1
For headings, an easy-to-read font is used that contrasts with the main text	1	1	1	1	1	1	1	1	1	1	1	1	1	1	1	1	1	1	1	1	1	1
In general, no more than 2 or 3 different typefaces are used	1	1	1	1	1	1	1	1	1	1	1	1	1	1	1	1	1	1	1	1	1	1
The font size is large enough for the intended audience	1	1	1	1	1	1	1	1	1	1	1	1	1	1	1	1	1	1	1	1	1	1
Upper and lower case are used, not all capitals	1	1	1	1	1	1	1	1	1	1	1	1	1	1	1	1	1	1	1	1	1	1
To emphasize words and phrases italics or bold text are used	1	1	1	1	1	1	1	1	1	1	1	1	1	1	1	1	1	1	1	1	-1	1
For ease of reading, dark-coloured text is used on a very light background	1	1	1	1	1	1	1	1	1	1	1	-1	1	1	1	1	1	1	1	1	-1	1
Text is not aligned sideways, on patterned or shaded background or on top of photos or other images	1	1	1	1	1	1	1	1	1	1	1	1	1	1	1	1	1	1	1	1	1	1
For ease of reading, extra line spacing has been added	1	1	1	1	1	1	1	1	1	1	1	1	1	1	1	1	1	1	1	1	1	1
For ease of reading, left justification is used throughout	1	1	1	1	1	1	1	1	1	1	1	1	1	1	1	1	1	1	1	1	1	1
Lines of text are an appropriate length—neither too long nor too short	-1	1	1	1	1	1	1	1	1	1	-1	-1	1	-1	-1	1	-1	-1	1	1	-1	1
Hyphenation has been avoided at the end of lines	1	1	1	1	1	1	1	1	1	1	1	1	1	1	1	1	1	1	1	1	1	1
Headings, bulleted lists, and blocks of text	1	1	1	1	1	1	1	1	1	1	1	1	1	1	1	1	1	1	1	1	1	1
There is a clear hierarchy of prominent headings and sub-headings	1	1	1	1	1	1	1	1	1	1	1	1	1	1	1	1	1	1	1	1	1	1
Contrast is used to make the main points stand out	1	1	1	1	1	1	1	1	1	1	1	1	1	1	1	1	1	1	1	1	-1	1
Bulleted lists are well formatted	1	1	1	1	1	1	1	1	1	1	1	1	1	1	1	1	1	-1	1	1	1	1
Effective ways are used to emphasize important blocks of text	1	1	1	1	1	1	1	1	1	1	1	1	1	1	1	1	1	1	1	1	-1	1
Use of colour	1	1	-1	-1	-1	1	1	1	1	1	1	1	1	1	1	1	1	1	1	1	1	1
Colours used are appealing to the intended readers	1	1	-1	-1	1	1	1	1	1	1	1	-1	1	1	1	1	1	1	1	1	1	1
Colour is used sparingly and in a consistent and deliberate way	1	1	1	1	1	1	1	1	1	1	1	-1	1	1	1	1	1	1	1	1	1	1
The colour scheme works from a design standpoint and when printed	1	1	1	1	1	1	1	1	1	1	1	-1	1	1	1	1	1	1	1	1	-1	1
The colour scheme works for with diminished or limited colour perception	1	1	1	1	1	1	1	1	1	1	1	-1	1	1	1	1	1	1	1	1	-1	1

For peer review only

Scoring Criteria	45	46	47	48	49	50	51	52	53	54	55	56	57	58	59	60	61	62	63	64	65	66
Photographs and illustrations	1	1	-1	-1	-1	-1	-1	1	-1	-1	-1	1	1	1	-1	1	1	-1	1	-1	-1	1
Photos and illustrations are used that relate directly to the information to reinforce key messages	-1	-1	0	0	0	0	0	-1	0	0	0	0	-1	-1	-1	-1	-1	0	1	0	0	1
Images used are clear, uncluttered, and consistent in style	1	1	0	0	0	0	0	1	0	0	0	0	1	0	-1	0	0	0	1	0	0	1
Photos and illustrations used are culturally appropriate for the intended readers	1	0	0	0	0	0	0	1	0	0	0	1	1	0	0	0	1	0	1	0	0	1
When images include people, they are appropriate to the situation and intended audience	-1	-1	0	0	0	0	0	1	0	0	0	1	1	-1	0	-1	0	0	1	0	0	-1
Tables, charts, and diagrams	-1	-1	-1	-1	-1	-1	-1	-1	-1	-1	-1	-1	-1	-1	-1	-1	-1	-1	-1	-1	-1	-1
Likely literacy levels of the reader have been considered in the use of tables, charts, and diagrams	0	0	0	0	0	0	0	0	0	0	0	0	0	0	0	0	0	0	1	0	0	0
Titles, headings, and other labelling is specific and clear	1	1	1	1	1	1	1	1	1	1	1	1	1	1	1	1	1	1	1	1	1	1
A clean, uncluttered layout is used with strong visual cures to guide the reader through the information	1	1	1	1	1	1	1	1	1	-1	1	-1	1	-1	-1	1	-1	-1	1	1	1	1
Numbers or calculations are carefully explained	0	0	0	0	0	0	0	0	0	0	0	0	0	0	0	0	0	0	0	0	0	0
General information about website	-1	-1	1	1	1	1	1	1	-1	1	1	-1	-1	-1	1	-1	1	-1	1	1	-1	1
Last updated date given	-1	-1	1	1	1	1	1	1	-1	1	1	-1	-1	-1	-1	-1	-1	-1	1	1	-1	1
Frequency of updates given	-1	-1	-1	-1	1	1	1	1	-1	1	-1	-1	-1	-1	-1	-1	-1	-1	-1	1	-1	-1
Relevant references given	-1	-1	-1	-1	-1	-1	-1	-1	-1	-1	1	-1	-1	-1	-1	-1	-1	-1	-1	-1	-1	-1
**Overall assessment score**	24	25	22	22	28	30	30	34	24	28	28	10	28	12	12	20	18	10	35	30	8	28

(+1 point if the statement was achieved, 0 if the statement was not applicable and -1 point if the statement was not achieved.)

For peer review only

	Website																		
**Scoring Criteria**	**67**	**68**	**69**	**70**	**71**	**72**	**73**	**74**	**75**	**76**	**77**	**78**	**79**	**80**	**81**	**82**	**83**	**84**	**85**
Overall design and page layout	-1	1	1	1	-1	1	1	1	1	1	-1	1	-1	1	1	1	-1	-1	1
The size, shape and general look of the material was designed with its purpose and users in mind	1	1	1	1	1	1	1	1	1	1	-1	1	1	1	1	1	1	-1	1
The material looks appealing at first glance	-1	1	1	-1	-1	1	1	-1	-1	-1	-1	1	-1	1	1	1	1	-1	1
A clear and obvious path has been created for the eye to follow	-1	1	1	1	1	1	1	1	1	1	-1	1	1	1	-1	1	1	-1	1
The material that has a clear and consistent style and structure	1	1	1	1	1	1	1	1	1	1	-1	1	1	1	-1	1	1	-1	1
Fonts, size of print and contrast	1	1	1	1	1	1	-1	-1	1	1	-1	1	1	1	1	1	1	1	1
For the regular text, a font that is designed for ease of reading is used	1	1	1	1	1	1	-1	1	1	1	-1	1	1	1	1	1	1	1	1
For headings, an easy-to-read font is used that contrasts with the main text	1	1	1	1	1	1	1	-1	1	1	1	1	1	1	1	1	1	1	1
In general, no more than 2 or 3 different typefaces are used	1	1	1	1	1	1	1	1	1	1	1	1	1	1	1	1	1	1	1
The font size is large enough for the intended audience	1	1	1	1	1	1	1	-1	1	1	-1	1	1	1	1	1	1	1	1
Upper and lower case are used, not all capitals	1	1	1	1	1	1	1	1	1	1	1	1	1	1	1	1	1	-1	1
To emphasize words and phrases italics or bold text are used	-1	1	1	1	-1	1	1	1	1	1	1	1	1	1	1	1	1	1	1
For ease of reading, dark-coloured text is used on a very light background	-1	1	1	1	1	1	1	1	1	1	-1	1	1	1	1	1	1	1	1
Text is not aligned sideways, on patterned or shaded background or on top of photos or other images	1	1	1	1	1	1	1	1	1	1	1	1	1	1	1	1	1	1	1
For ease of reading, extra line spacing has been added	1	1	1	1	1	1	1	1	1	1	-1	1	1	1	1	1	1	1	1
For ease of reading, left justification is used throughout	1	1	1	1	1	1	1	1	1	1	1	1	1	1	1	1	1	-1	1
Lines of text are an appropriate length—neither too long nor too short	-1	1	1	-1	-1	1	-1	1	1	1	-1	1	-1	1	-1	-1	1	1	1
Hyphenation has been avoided at the end of lines	1	1	1	1	1	1	1	1	1	1	1	1	1	1	1	1	1	1	1
Headings, bulleted lists, and blocks of text	1	1	1	1	-1	1	1	1	1	1	1	1	1	1	1	1	1	1	1
There is a clear hierarchy of prominent headings and sub-headings	1	1	1	1	-1	1	1	1	1	1	-1	1	1	1	1	1	1	-1	1
Contrast is used to make the main points stand out	1	1	1	1	-1	1	1	1	1	1	1	1	1	1	1	1	1	1	1
Bulleted lists are well formatted	1	1	1	1	-1	1	1	1	1	1	1	1	1	1	1	1	1	1	1
Effective ways are used to emphasize important blocks of text	1	1	1	1	-1	1	1	1	1	1	-1	1	1	1	1	1	1	1	1
Use of colour	1	1	1	1	-1	1	1	1	1	1	-1	1	1	1	1	1	1	1	1
Colours used are appealing to the intended readers	-1	1	1	1	-1	1	1	1	1	-1	-1	1	1	1	1	1	1	1	1
Colour is used sparingly and in a consistent and deliberate way	1	1	1	1	1	1	1	1	1	-1	-1	1	1	1	1	1	1	1	1
The colour scheme works from a design standpoint and when printed	-1	1	1	1	1	1	1	1	1	1	-1	1	1	1	1	1	1	1	1
The colour scheme works for with diminished or limited colour perception	-1	1	1	1	1	1	-1	1	1	-1	-1	1	1	1	1	1	1	1	1

For peer review only

Scoring Criteria	67	68	69	70	71	72	73	74	75	76	77	78	79	80	81	82	83	84	85
Photographs and illustrations	1	-1	1	1	-1	1	-1	-1	-1	1	-1	-1	1	-1	-1	-1	-1	1	1
Photos and illustrations are used that relate directly to the information to reinforce key messages	-1	0	1	1	0	-1	0	0	0	1	0	0	1	0	0	0	0	-1	1
Images used are clear, uncluttered, and consistent in style	1	0	1	-1	0	1	0	0	0	1	0	0	1	0	0	0	0	-1	1
Photos and illustrations used are culturally appropriate for the intended readers	1	0	1	1	0	0	0	0	0	1	0	0	1	0	0	0	0	1	1
When images include people, they are appropriate to the situation and intended audience	0	0	0	1	0	0	0	0	0	1	0	0	1	0	0	0	0	1	1
Tables, charts, and diagrams	-1	-1	-1	1	-1	-1	-1	-1	-1	1	-1	-1	1	-1	-1	-1	-1	-1	-1
Likely literacy levels of the reader have been considered in the use of tables, charts, and diagrams	0	0	1	-1	0	-1	0	0	0	1	0	1	1	0	0	0	0	0	0
Titles, headings, and other labelling is specific and clear	1	1	1	1	1	1	1	1	1	1	1	1	1	1	1	1	0	1	1
A clean, uncluttered layout is used with strong visual cures to guide the reader through the information	-1	1	1	1	1	1	1	1	1	1	-1	1	-1	1	-1	1	1	-1	1
Numbers or calculations are carefully explained	0	0	0	0	0	0	0	0	0	0	0	0	0	0	0	0	0	0	0
General information about website	-1	1	1	1	1	-1	-1	-1	-1	-1	-1	1	-1	1	-1	-1	-1	-1	1
Last updated date given	-1	1	1	1	1	-1	-1	-1	-1	-1	-1	1	-1	1	-1	-1	-1	-1	1
Frequency of updates given	-1	1	1	-1	-1	-1	-1	-1	-1	-1	-1	1	-1	1	-1	-1	-1	-1	-1
Relevant references given	-1	1	-1	1	-1	1	-1	-1	-1	-1	-1	-1	-1	-1	-1	-1	1	-1	-1
**Overall assessment score**	7	32	36	31	6	27	16	16	22	25	-14	31	25	30	16	22	23	8	34

(+1 point if the statement was achieved, 0 if the statement was not applicable and -1 point if the statement was not achieved.)

For peer review only

Supplementary information. Table S2. Narrative overview assessment of the 85 identified websites.

Name of Website	Website address	Website assessment
1. Medscape	https://emedicine.medscape.com/article/428723-overview?form=fpf	The Medscape website is clearly laid out, with bold headings, sub-headings and regular use of paragraphs. This makes it aesthetically appealing, with clear links to move between different sections of the article. However, there is heavy use of medical terminology and information that is not relevant or useful to the public as it details complex anatomical and physiological structures. There is no use of diagrams, only an image of an x-ray. The website does include information on causes, symptoms and treatments of BCT. This website appears more focused on use by clinicians than by the general public.
2. UpToDate	https://www.uptodate.com/contents/initial-evaluation-and-management-of-blunt-thoracic-trauma-in-adults	The UpToDate website page contains very informative content regarding BCT. There are links to further information on specific injuries, however this website focuses more on the anatomical nature of these injuries rather than symptoms and how to diagnose them. In general, this website seems to focus on information relevant to a clinician rather than a member of the public, with detailed descriptions of medical techniques. The layout is poor, as the text area only takes up around half of the website page. There are links to images, although they are all scientific and of little use when educating the public.
3. Health Direct	https://www.healthdirect.gov.au/chest-injuries	The Health Direct website provides good information on chest injury, for both BCT and penetrating injuries. Headings are effectively used to segregate the information for blunt and penetrating wounds. The page is well laid out with bullet points highlighting key points. Among a list of possible chest injuries, there is extra information given on the most common ones. This shows that the primary aim of this website is to educate members of the public on potential BCT injuries, and use of non-technical language allows the page to do this effectively. An area in which the website

For peer review only

Name of Website	Website address	Website assessment
		lacks is the lack of images and diagrams, which can be effective in communicating some potential visible symptoms of chest injuries.
4. DynaMed	https://www.dynamed.com/management/blunt-chest-trauma-emergency-management#GUID-E3A89009-4D66-4D2B-9ABE-DF2E70EE0CFA	The general appearance of the DynaMed website is appealing, but the full content is not available as it requires a subscription for more detailed information. There are some colourful pictures but the explanation of these and the text in general is very scientific and appears to be more appropriate for clinicians than patients. A medical and or radiological understanding would be needed to interpret the x-ray photos.
5. Wikipedia	https://en.wikipedia.org/wiki/Chest_injury	While Wikipedia does not have a designated website page solely for BCT, it has a general blunt trauma page in which a section is designated to the thoracic region. While there is not a large volume of information on BCT, it contains causes and symptoms for certain chest injuries. However, the majority of the text contains medical terminology. The layout is confusing, with no subheadings and poor spacing. Some positives of the page included the use of tables that clearly outline some types of BCT accidents and what injuries they could cause, which was an effective tool for education on BCT. There were also a variety of links taking the reader to further information if they needed it.
6. North Bristol NHS Trust	https://www.nbt.nhs.uk/our-services/a-z-services/emergency-zone/ed-miu-patient-informationtt-information/chest-injury-advice	This is badged with an NHS and hospital logo which immediately gives credibility to the webpage. It is coloured in the usual blue and white NHS colour scheme. The webpage is titled "Chest injury advice- what happens when you're admitted to hospital with a chest injury', which allows the person accessing the site to determine the relevancy of the information. The language is very straightforward and there is clear delineation of sections. Bold text is used to highlight key headings in a large font. Dark font is used on a white background to aid contrast. The information is however very brief and there are no figures/diagrams to aid understanding. Useful contact information is included.

For peer review only

Name of Website	Website address	Website assessment
7. Drugs.com	https://www.drugs.com/cg/blunt-chest-trauma.html	The Drugs.com webpage has several adverts placed throughout the page. These take up a large part of the screen, including throughout the text sections. The information is largely to do with treatment and care for BCT injuries, with little information about causes and symptoms. Headings and bullet-points make the text appealing to read, but the lack of images hinders the visual appearance overall. Some abbreviations are used without reference to what they mean, which could confuse readers with no background knowledge. There are links to further information at the bottom of the page.
8. Gloucestershire Hospitals NHS Trust	https://www.gloshospitals.nhs.uk/media/documents/ Chest_trauma_GHPI0653_01_22_4EXEhZ5.pdf	This web address provides a link to a patient information leaflet about chest trauma. It is badged with the Gloucester Hospital logo and the NHS which immediately improves credibility. It is very clean and clear and has major headings that introduce chest trauma, what it is and how it can be managed. It also gives advice on when the patient should seek more help and contact information. The sentences are short and clear. There are no figures, tables and graphics which could potentially help aid understanding.
9. VeryWellHealth	https://www.verywellhealth.com/chest-trauma-3913241	This website has several adverts at the top and down the side of the page. The text itself focuses on all chest injuries rather than just BCT, with very little mention of BCT. The layout of text is good, with subheadings and images used well, as well as links to further information present. The language used is appropriate for most readers.
10. MSD Manual	https://www.msdmanuals.com/en-gb/home/injuries-and-poisoning/chest-injuries/blunt-injury-to-the-heart	This website contains information on chest injuries, with only some relevant to BCT. The introduction contains appropriate sections on symptoms, diagnosis, treatment for individuals with no clinical knowledge. However, the following sections use more advanced terminology that is not suitable for education of the public. There is limited use of images, and the text only makes use of around half of the page.

For peer review only

Name of Website	Website address	Website assessment
11. The American Association for the Surgery of Trauma	https://www.aast.org/resources-detail/blunt-cardiac-injury	The website visually looks very professional on first inspection. There are some adverts which distract from the information provided. The language is very clinical as would be expected of a professional body. The information also relies of medical knowledge to understand it. There are no figures/photographs or tables which could potentially aid understanding.
12. LifeInTheFastlane	https://litfl.com/thoracic-trauma/	This website is visually unappealing, with little use of colour, adverts down one side of the page, and too much empty white space. The information contained in the page appears aimed at medical professionals, as medical terminology is used throughout. The information also seems to be solely based on types of injury, and how they can be identified and treated by a medical professional. No images are used to accompany the text. There are no links to further information.
13. Radiology key	https://radiologykey.com/blunt-chest-trauma/	This website is organised in a very simple manner, with no adverts or pop-ups. The information contained however is too advanced, due to the use of medical terminology and images of x-rays and CT scans that would only be helpful to a medical professional. The layout of the text is also poor, with long sentences and paragraphs. There is no easy way to navigate through the website, due to the length of the text and lack of links to specific sections.
14. Broken or bruised ribs (NHS): multiple sites	https://www.eastenfieldmedicalpractice.nhs.uk/conditions/broken-or-bruised-ribs/	Many identified links in the NHS (in primary and secondary care) used the same standard text, under their name, with a clear badge that identified that they were an NHS site. This text was simple and clear and written in a language appropriate for a lay person. The focus was only on bruised or broken ribs rather than blunt chest trauma specifically. There was a brief summary to help patients identify if they had bruised/broken ribs, followed by self help information and how to get further advice or make contact. There were no distracting adverts, but also no figures/diagrams to aid understanding.

For peer review only

Name of Website	Website address	Website assessment
15. Oxford University Hospitals NHS Trust	https://www.ouh.nhs.uk/patient-guide/leaflets/files/10110Pchestinjury.pdf	This web address provides a link to a patient information leaflet about chest trauma. It is badged with the Oxford Hospital logo and the NHS which immediately improves credibility. It is very clean and clear and has major headings that introduce the relevant information. Bold headings are used to emphasise key information. There is limited use of colour and no figures/diagrams to illustrate the information. Contact details are included if the patient needs more information.
16. National Institute for Health and Care Excellence (NICE)	https://www.nice.org.uk/guidance/ng39/ifp/chapter/treating-chest-injuries	The NICE website contains information on all types of trauma injuries, making it only partially relevant to BCT. The page is visually appealing, with no adverts taking up space, and the text is large enough to read easily. A section is specified as 'information for the public', making it very clear to the reader which section is intended for their education. No images are used, but clear sub-headings and short paragraph lengths make the text visibly appealing and easy to navigate. Links to further information on care are clearly present.
17. Aneurin Bevan University Health Board	https://abuhb.nhs.wales/files/patient-information-leaflets1/accidents-and-minor-injuries/chest-injury-advice-sheet-pdf/	The web address links to a patient information leaflet about chest injury advice. The NHS and hospital logos increase credibility of the information. The language is fairly clear and straightforward but it looks quite cluttered with limited use of white space and as such is not very appealing. Red text and coloured text are sometimes used to used to highlight important areas but the colours are not appealing and may be difficult to see if printed. Some figures have been used to aid understanding.
18. My Health Alberta	https://myhealth.alberta.ca/Health/aftercareinformation/pages/conditions.aspx?hwid=uf7538	The MyHealth.Alberta website is focused on chest contusions, so again not solely on BCT. The information contained is quite brief, being mainly focused on care instructions for someone with a chest contusion. Further links to specific chest conditions are provided. There is no use of images or diagrams in addition to the text, however the page is clearly laid out with headings and bullet-points. The text could take up more of the page, with large areas of empty

For peer review only

Name of Website	Website address	Website assessment
		space. The language used is appropriate for someone with no medical background, due to almost no medical terminology used.
19. Salisbury NHS Foundation Trust	https://www.google.com/search?q=blunt+chest+trauma+salisbury&rlz=1C1CHBF_en-GBGB987GB987&oq=blunt+chest+trauma+salisbury&gs_lcrp=EgZjaHJvbWUyBggAEEUYOTIHCAEQIRigATIHCAIQIRigATIHCAMQIRigAdIBCDU1MTFqMWo5qAIAsAIA&sourceid=chrome&ie=UTF-8	The web address links to a patient information leaflet about blunt chest trauma. The language is very clear and straightforward but the information is brief and the leaflet is not particularly appealing with only black text on a white background. There are no NHS or hospital logos but the name of the NHS Trust provides a level of credibility. There are no figures to help illustrate but there basic management information is provided as well as contact information if required.
20. WebMD	https://www.webmd.com/heart/what-is-myocardial-contusion	There are lots of adverts throughout the text and to the right which distract from information provided. Many of the adverts are not relevant to the clinical information. The information itself is fairly easy to read and links are provided to linked information. There are however a large number of medical terms used which may limit understanding.
21. After Trauma	https://www.aftertrauma.org/diagnosis-and-treatment/managing-chest-wall-injuries	This website contains two pages dedicated to chest injuries. It focuses on the management of injuries, treatment and recovery, although some information was included on signs and symptoms. The page is well laid out, with good use of headings, sub-headings and bullet-points. There are no distracting adverts or unnecessary information, and there is good use of white space, making it easy to read. One clear diagram is used to show the anatomy of the chest, which complements the text. One negative is that there is medical terminology used throughout.
22. Royal Devon University Healthcare NHS Foundation Trust & Northern Devon Healthcare NHS Trust	https://www.royaldevon.nhs.uk/media/z51hk2mn/advice-after-a-chest-wall-injury.pdf	The web address links to a patient information leaflet about chest wall trauma. There are NHS and hospital logos which provides a level of credibility. The language is very clear and straightforward and provides background and self-help guidance as well as further contact information. It also includes clear figures that aid understanding. The language is clear.

For peer review only

Name of Website	Website address	Website assessment
23. South Tees Hospitals NHS Foundation Trust	https://www.southtees.nhs.uk/resources/chest-injury/	The website is badged with the hospital and NHS logos. It uses the blue and white NHS colouring so immediately appears credible. The information is all organised on one page and the language is straightforward. It provides a brief overview of chest injuires with symptoms, self-help guidance and contact information about seeking further help if needed. There is one generic image but nothing to help understand the information. It is clean and uncluttered with no advertisements.
24. Sirona care and health	https://www.sirona-cic.org.uk/wp-content/uploads/2022/02/0050_How-to-treat-a-Chest-Wall-web.pdf	This link takes you to a patient information leaflet titled 'how to treat a chest wall injury'. It is very professional looking and is badged with the NHS logo, thus improving credibility. There is limited use of colour on a plain white background. It provides an overview of symptoms, dos and don'ts, management and contacts in an emergency. There are no figures or tables and no adverts. The language is clear.
25. eMedicineHealth	https://www.emedicinehealth.com/wilderness_chest_injuries/article_em.htm	The emedicinehealth website contains information on chest injuries of all description, but specifically discusses BCT causes and injuries. The layout of the page is good, with white space and sub-headings used effectively to allow easy navigation of the text. Bullet-points are used to highlight key pieces of information, and pictures complement the text. The language used is appropriate, and there are links to access further information. However, there are some adverts present that may distract the reader.
26. Better Health Channel	https://www.betterhealth.vic.gov.au/health/conditionsandtreatments/rib-injuries	This is a very clean, uncluttered and professional looking website that focuses on rib injuries. Colour is used sparingly and with lots of white space. The information is broken down into clear headings. Some of the language used is a bit technical. There are no figures or tables which could aid understanding, but also no adverts to distract.
27. Rib Injury Clinic	https://www.ribinjuryclinic.com/conditions/complex-chest-wall-injuries/	On initial inspection this website looks quite appealing as it contains lots of images and photographs. It uses dark text on a light background but could benefit from more white space. Further

For peer review only

Name of Website	Website address	Website assessment
		inspection identifies that the conext is very complex as are the photographs and figures. It would be very difficult for a patient to understand the information. The language used is also highly technical. On the positive side there are no distracting adverts.
28. Hull University Teaching Hospitals NHS Trust	https://www.hey.nhs.uk/patient-leaflet/__trashed-35/	The link to this information first directs you to an 'advert' for accessibility testers, which is a little distracting and has to be closed before further information can be accessed. Once the information is accessed it is badged as the hospital and NHS which adds credibility. There is 'admin' information at the top of the page and the website menu to the right which makes the flow of information difficult. The information itself is a little complex but provides background, self-help guidance and where to go for more help. No figures or tables.
29. Mount Nittany Health	https://mountnittany.org/wellness-article/chest-wall-contusion	This website provides some basic information about 'chest contusion/brusing. It provides some background including a simple diagram of the chest. The language used is not too technical and it provides information about management and where to go for further help. No distracting adverts are included.
30. MedlinePlus	https://medlineplus.gov/chestinjuriesanddisorders.html	The website feels rather cluttered as there is admin information and a menu to the right. There are no adverts. The information provided is fairly simple and there are links to further information including how various chest conditions are diagnosed. Limited technical language is used in the main narrative, although many links use technical language.
31. European Society of Thoracic Surgeons	https://www.ests.org/about_ests/patient_information/diseases/chest_trauma.aspx#googtrans/en/en	This website has information spread over several pages. Information is not solely BCT related, however a large amount is relevant. The layout of the page is clear, with no adverts and good use of white space. Key pieces of information are highlighted using bullet-points. Some of the language used is too advanced for use by the public, and there is a lack of images giving a visual representation of the information in the text. There are links to further information.

For peer review only

Name of Website	Website address	Website assessment
32. Cleveland Clinic	https://my.clevelandclinic.org/health/diseases/23994-flail-chest	The website contains adverts at the top and to the right which distracts from the information provided. Generally the information looks fairly appealing and includes a simple figure. It focuses on 'flail chest'. It summarises key information but uses technical terms that make the information more difficult to understand.
33. mactheknife.org	www.mactheknife.org/Chest_Trauma/Mechanisms.html	This website provide some detail about the mechanisms of chest injory. There is a lot of text presented with limited line spacing on a patterned background. Visually this makes the information difficult to read especially for those with sight impairments. There are no figures or photos but also no distracting adverts. The content is very complex and lots of technical terms are used.
34. World Rugby Passport	https://passport.world.rugby/player-welfare-medical/first-aid-in-rugby/chapter-6-breathing-assessment-and-chest-injuries/potential-causes-of-rapid-breathing/chest-wall-or-lung-injuries/	This website presents a brief page of information about how to manage chest wall or lung injuries in rugby players. The website itself is very professional looking but the information is very limited and appears to be focused of how clinicians manage injuries. There are no figures or photos but equally no distracting adverts. The information presented is very clear and non-technical.
35. Total Vitality Medical Group	https://totalvitalitymedical.com/news/what-you-need-to-know-about-chest-contusions/	This website focuses on chest contusions/bruising and the page begins with a photograph of a bruised chest. The website looks very professional but the colours used could be improved as they may be problematic with some people with visual impairments and may not print well. The website gives a good overview of chest brusing, how it may be caused and how it can be managed. There are no distracting adverts. Generally is reads well, but some complex medical terms are used which make it more difficult to read.
36. Healthline	https://www.healthline.com/health/flail-chest	The Healthline website is organised in a visually appealing way with large text, easy-to-read font used and clear sub-headings. Adverts are present throughout the page, including pop-up windows, which are distracting and block the information from being read. No images are used, but bullet-points show key facts and highlighted

For peer review only

Name of Website	Website address	Website assessment
		phrases contain links to further information. Some language used is too advanced to be read by someone with no medical knowledge.
37. NHS Lanarkshire	https://www.nhslanarkshire.scot.nhs.uk/patient-information-leaflets/emergency-department/pil-chsinj-67661-l/	This website provides very brief information to patients who have presented with a chest injury to hospital. It includes the main signs and symptoms to consider and signposting should they need further advice. It is badged with the hospital name and NHS so adds credibility. There are no figures or photos but also no adverts. The language is straightforward and east to understand.
38. Cambridge University Hospitals NHS Foundation Trust	https://www.cuh.nhs.uk/patient-information/chest-injury-advice-sheet-for-patients/	This website is badged with the NHS and hospital logos which adds credibility. It provides very brief information about how patients should manage their chest injury after presenting to hospital. It mainly focuses on pain management and who to contact in an emergency. The language is very clear and simple. No images or adverts.
39. National University Hospital; National University Hospital Health System	https://www.nuh.com.sg/Health-Information/Diseases-Conditions/Pages/Chest-Injuries.aspx	The website is clean and uncluttered but the information contained within it is not really patient focused and uses lots of technical terms and diagrams where anatomy knowldege is assumed. There is limited helpful information for patients. There are no distracting adverts.
40. The Rotherham NHS Foundation Trust	https://www.therotherhamft.nhs.uk/patients-and-visitors/patient-information/chest-trauma-and-fractured-ribs#:~:text=If%20you%20do%20experience%20pain,please%20contact%20your%20GP%20practice.	This website is badged with the NHS and hospital logos which adds credibility. It provides comprehensive information about chest wall trauma and rib injuries and how patients should manage their injury after presenting to hospital. It is written in a very simple way and avoids using technical language. There are no figures to help illustrate the information but also no adverts.
41. Healthcare Associates of Texas	https://healthcareassociates.com/strained-chest-muscle-causes-symptoms-and-treatment/	The website feels rather cluttered as there is a menu on the right hand side and regular adverts interspersed with text to encourage users to 'book an appointment' which distracts from the information provided. There are no other adverts and some photos of people with possible chest injuries but no accompanying information. The information focuses of strained muscles, how you

For peer review only

Name of Website	Website address	Website assessment
		can get a muscle strain and treatment. The information is fairly clear and avoids the use of medical terminology.
42. Physio-pedia	https://www.physio-pedia.com/Sternal_fracture	Although this website does not contain external adverts, it feels rather cluttered as it advertises various physiotherapy courses before presenting the information on sternal fractures. There is a picture of the ribs, but no information to aid understanding. The information is fairly comprehensive but uses lots of medical terminology and long sentences which make understanding difficult.
43. Medical News Today	https://www.medicalnewstoday.com/articles/324534	Visually, the page is well laid-out, with good use of sub-headings, pictures and bullet-points to allow key information to be identified easily. The pictures are non-scientific, and work in conjunction with the text to demonstrate causes and treatments. Links at the top of the page allow easy movement between sections, which are concise and use space well. However, medical terminology is used throughout, and adverts and pop-ups may distract the reader.
44. My Family Physio	https://myfamilyphysio.com.au/common-upper-back-and-chest-injuries/#:~:text=Rib%20fractures%20and%20cartilage%20strains,pull%20starting%20a%20lawn%20mower).	The link to this website opens with a pop up message about still accepting appointments. This has to be closed before accessing the information which is distracting. The information provided is about common back and chest injuries so only parts of it are relevant. It includes some nice figures but limited explanation of them. The text contains a lot of medical terminology which makes it difficult to read.
45. Banner Health	https://www.bannerhealth.com/healthcareblog/better-me/what-to-know-about-strained-chest-muscles#:~:text=In%20most%20mild%20to%20moderate,area%20to%20help%20reduce%20pain.	This website focuses of the differences between chest injuries and a heart attack. It includes some photos but these do not really help to illustrate the text. There are no adverts. The article is largely clear but uses a lot of medical terminology.
46. Luna Physical Therapy	https://blog.getluna.com/pulled-chest-muscle-causes	This website provides a brief overview about pulled chest muscles, causes and treatment. The focus of the website is on physical therapy and the content largely reflects this. There are some photos but these do not aid the understanding of the content. The

For peer review only

Name of Website	Website address	Website assessment
		website is fairly clear and for the most part avoids the use of medical terminology.
47. Healthwise	https://www.cham.org/HealthwiseArticle.aspx?id=sig52119	This website is fairly bland with limited badging to identify the source, no figures, tables or photos. It does not have any adverts. Despite this it provides a brief summary about types of chest injuries as well as links to other conditions. The text avoids or explains medical terminology as far as possible which aids understanding.
48. Wirral Community Health and Care NHS Foundation Trust	https://www.wchc.nhs.uk/wp-content/uploads/2020/03/Chest-Injury_V1.pdf	This link takes you to a patient information leaflet titled 'chest injury'. It is very professional looking and is badged with the NHS and hospital logos, thus improving credibility. There is limited use of colour on a plain white background. There is a brief background to the injury as well as self-help guidance and how to get further help. No pictures or diagrams used. Medical terminology is avoided or explained and the language is very simple.
49. Barnsley Hospital NHS Foundation Trust	https://www.barnsleyhospital.nhs.uk/services/a-and-e/chest-injury	This website provides a very brief paragraph mainly to guide patients on its management but also to signpost for further help. It is badged with the hospital and NHS logos which improves credibility. The language is simple and clear. No figures or photos used. No adverts.
50. The Dudley Group NHS Foundation Trust	https://www.dgft.nhs.uk/wp-content/uploads/2023/09/Chest-injury-V3.pdf	This link takes you to a patient information sheet that describes chest injuries. It is badged with the hospital and NHS logos which improves credibility. It describes the causes, symptoms, how to manage it and where to go for further help. There are no figures or photos but the language is clear and limited medical terminology is used. There are no adverts.
51. York Hospitals	https://www.yorkhospitals.nhs.uk/seecmsfile/?id=6844	This link takes you to a patient information sheet that describes rib/chest injuries. It is badged with the hospital and NHS logos which improves credibility. It describes the causes, symptoms, how to manage it and where to go for further help. There are no figures or photos but the language is clear and limited medical terminology is used. There are no adverts.

For peer review only

Name of Website	Website address	Website assessment
52. Bradford Teaching Hospitals NHS Foundation Trust	https://digitalpatientinformationhub.bradfordhospitals.nhs.uk/leaflets/chest-injury/	This website provides very brief information mainly to guide patients on management of chest injuries following a visit to hospital. It also signposts for further help. It is badged with the hospital and NHS logos which improves credibility. The language is simple and clear. A simple illustration is used to illustrate a chest injury but otherwise there are no figures or photos used to help illustrate the text. No adverts.
53. Milton Keynes University Hospital	https://www.mkuh.nhs.uk/patient-information-leaflet/rib-or-chest-injury	This website provides a very brief paragraph mainly to guide patients on the management of rib or chest injuries but also to signpost for further help. It is badged with the hospital and NHS logos which improves credibility. The language is simple and clear. No figures or photos used. No adverts.
54. Dorset County Hospital	https://www.dchft.nhs.uk/wp-content/uploads/2020/10/ED-Chest-and-Rib-Injury-0414.pdf	This link takes you to a patient information sheet that describes how to manage rib/chest injuries onced discharged from hospital. It is badged with the hospital and NHS logos which improves credibility. It looks a little cluttered. There are no figures or photos but the language is clear and limited medical terminology is used. There are no adverts.
55. Mount Sinai	https://www.mountsinai.org/health-library/selfcare-instructions/rib-fracture-aftercar	This website provides information on rib fracture after care following presentation. It is nicely laid out and gives information about the injury, pain releif and what to expect. It also provides links to other conditions and references for further reading. The information is fairly straightforward although this is interspersed with medical terminology. There are no photos or illustrations and no external adverts although there is an 'internal advert' to book and appointment
56. Yale Medicine	https://www.yalemedicine.org/conditions/rib-fracture	This website provides an overview about rib fractures. The page itself looks rather cluttered as it has a menu on the left of the page. The colouring of some of the website may cause issues for those with sight impairments. The website itself looks fairly professional but the information is rather complex and includes lots of medical

For peer review only

Name of Website	Website address	Website assessment
		terminology. There is a photo included but this does not aid understanding of the text. No distracting adverts
57. Oxford Health NHS Foundation Trust	https://www.oxfordhealth.nhs.uk/wp-content/uploads/2014/08/OP-144.15-Rib-injury-advice.pdf	This link takes you to a patient information sheet that describes how to manage rib injuries. It is badged with the hospital and NHS logos which improves credibility. It is very brief but describes what a rib injury is and how it manage it. It also signposts contact information. There are no figures or photos but the language is clear and limited medical terminology is used. There are no adverts.
58. Brigham and Women's Hospital	https://www.brighamandwomens.org/lung-center/diseases-and-conditions/rib-fractures	This website looks rather cluttered as it immediately advertises appointments for patients which distracts from the main information about rib fractures. There is also a menu on the left of the page which is distracting. It includes a photograph at the top of the page but it is not relevant to rib fractures. The information itself is rather complex with lots of medical terminology. No other relevant illustrations are included but also no adverts.
59. topdoctors.co.uk	https://www.topdoctors.co.uk/medical-articles/rib-fractures-a-comprehensive-guide-for-patients	The website is rather cluttered with lots of pop-up messages before the information can be acccessed. It also includes information and prices to book an appointment which distracts from the information about rib fractures. There is a photo of the rib cage but this does not aid understanding of the text. It gives a reasonable summary about rib fractures but there is a lot of medical terminology used.
60. Ventura Orthopedics	https://venturaortho.com/difference-between-bruised-and-broken-ribs/	This website appears fairly professional initially. Further inspection however shows a ribbon of information including contact numbers, appointment booking and social media pages. There is a photo at the top of the page but it doesn't help to illustrate the content in any way. The information itself is fairly straightforward on the whole but does include some medical terminology. No adverts or other illustrations are included.
61. Nebraska Medicine	https://www.nebraskamed.com/trauma/help-broken-ribs-heal-faster	This website doesn't have dynamic adverts but includes bold information about booking an appointment which distracts from the information provided. It provides a brief overview about rib

For peer review only

Name of Website	Website address	Website assessment
		fractures which includes an x-ray photo of the ribcage. The photo does not aid in understanding the narrative provided. A lot of medical terminology and drug names are used which makes the content complex.
62. Dr Marco Scarci	https://www.marcoscarci.co.uk/what-are-the-long-term-effects-of-broken-ribs	The website looks cluttered with lots of photos and links to other articles to the right of the page. There are also interruptions to the text where links to make an appointment can be made. This distracts from the information provided. The text is quite small and there are no figures to aid understanding of the text. The information itself is a bit complex despite limited use of use of medical terminology. No adverts.
63. Doncaster and Bassetlaw Teaching Hospitals	https://www.dbth.nhs.uk/wp-content/uploads/2022/05/WPR48780-Rib-injury-leaflet.pdf	This link takes you to a patient information sheet that describes how to manage bruised or broken ribs. It provides a brief overview of do's and don'ts and where to get more advice. It also provides information about breathing exercises including photographs to illustrate. It is badged with the hospital and NHS logos which improves credibility. The language is clear and limited medical terminology is used. There are no adverts.
64. University Hospitals Coventry and Warwickshire	https://www.uhcw.nhs.uk/download/clientfiles/files/Patient%20Information%20Leaflets/Emergency%20Medicine/Emergency%20Department/118694_Emergency_Department_-_Bruised_or_broken_ribs_(1720)_-_March_2019.pdf	This link takes you to a patient information sheet that describes how to manage bruised or broken ribs following hospital discharge. It is badged with the hospital and NHS logos which improves credibility. It is very brief but describes what a rib injury is and how it manage it. It also signposts contact information. There are no figures or photos but the language is clear and limited medical terminology is used. There are no adverts.
65. Orthopaedics and Trauma London	http://www.orthopaedicsandtraumalondon.co.uk/conditions/rib-fractures/	This website looks fairly professional but gives the appearance of a sports website rather than a medical one, with photographs and sporting situations on the top of the page. It provides brief information about rib fractures, signs and symptoms, diagnosis and treatment. It provides a reasonable overview but the language is rather complex and there is a lot of medical terminology. No relevant illustrations and no adverts.

For peer review only

Name of Website	Website address	Website assessment
66. NCH Healthcare System	https://nchmd.org/health-library/diseases-and-conditions/con-20155268/	The top of this website is a bit cluttered and includes information about booking an appointment and paying bills etc. It also has a photo that is not related to the content on 'broken ribs' which appears lower down the page. The information contained is fairly well presented and includes an illustration of broken ribs. The page includes symptoms, causes, complications and treatment. The information is simple and avoids medical terminology. No distracting adverts.
67. Physiocheck	https://www.physiocheck.co.uk/condition/80/bruised-or-broken-rib	This website is very brightly coloured, but also feels very cluttered. There is a menu on the right hand side containing links to further information and how to make contact but this is quite distracting. It also includes active links for checking symptoms and asking a physio. There are a couple of illustrations of the ribs but these are difficult to understand for a lay person. The information provided on bruised or broken ribs is brief and includes a description, causes, signs and symptoms and treatment. It includes some medical terminology which makes comprehension more difficult but does try to explain these terms. No adverts.
68. Dr Gallagher and Partners	https://www.drgallagherandpartners.org.uk/website/M84035/files/rib%20inju.pdf	This link takes you to a patient information sheet that describes how to care for rib injuries. It looks very professional and describes the nature of the injuries, treatment and diagnosis. Some complex medical terminology is used but this is explained and the language is fairly simple. No illustrations to aid understanding but no adverts.
69. Agency for Clinical Innovation	https://aci.health.nsw.gov.au/networks/eci/clinical/ed-factsheets/rib-fracture	This website provides a fact sheet of information for people who have presented to the emergency department with rib fractures. Language is simple but a number of medical terms are used which may limit understanding. It provides a very brief overview of the condition and includes treatment and management. It also signposts to further contacts. It is clean and uncluttered and includes one illustration about breathing exercises. No adverts.

For peer review only

Name of Website	Website address	Website assessment
70. SportsMD.com	https://www.sportsmd.com/sports-injuries/chest-rib-injuries/rib-fracture-pneumothorax-complication/	The appearance of this website is generally ok, although the right of the page shows a large 'internal' advert promoting appointment bookings which is slightly distracting. There are a couple of illsurations that help with understanding of the text. The text is fairly comprehensive and includes types of injuries, complications, symptoms, causes and treatment. The page also includes a video. Some of the language is quite complex and includes a lot of medical terminology. No external adverts.
71. GP notebook	https://gpnotebook.com/simplepage.cfm?ID=-1120600051	This website opens with a number of pop-ups which are immediately distracting. Only limited information can be acccessed without subscription. The site is clearly labelled as a resource for healthcare professionals and the content reflects this as there is a lot of medical terminology and the language is very complex. The information provided is very brief. There are no illustrations. Some of the pages include dynamic adverts which are distracting.
72. Upstate University Hospital	https://www.upstate.edu/thoracic-surgery/conditions/rib-fracture.php	This website is clean and uncluttered and shows an image of the rib cage. It provides very brief information about rib fractures including causes, risk factors, symptoms and treatment. The information is very simple and brief and avoids medical terminology. There are no adverts to distract.
73. Fracture and Orthopaedic Clinic Ltd	https://www.faoconline.com/home/conditions/general/fracture-rib	This is a very professional looking website that provides information about fractured ribs including causes, symptoms and treatment. It is mainly black text on a white background and although the text size is adequate the text itself is quite light so may cause problems for people with sight issues. The language is a straightforward on the whole but includes medical terminology which may make comprehension difficult. There are no illustrations or adverts.
74. HealthyWA	https://www.healthywa.health.wa.gov.au/Articles/A_E/Chest-injuries-and-rib-fractures	This website is badged with the Department of Health Western Australia which adds credibility to the information. It provides very brief information about chest injuries and rib fractures and do's and don'ts but mainly signposts to further information if needed. There

For peer review only

Name of Website	Website address	Website assessment
		are no illustrations and no adverts. The language is very basic with limited used of medical terminology.
75. St James's Hospital	https://www.stjames.ie/media/Rib%20fracture%20Advice%20Leaflet.pdf	This link takes you to a patient information sheet that describes how to manage rib fractures. It is badged with the hospital logo which adds credibility to the information, however the quality of the logo is poor. It provides an overview, a list of do's and don'ts, but is primarily focused on signposting. The language is simple and clear with limited use of medical terms. There are no illustrations and no adverts.
76. International Centre for Thoracic Surgery	https://www.icts.com.sg/rib-fracture-fixation-from-trauma/	This website is a little overwhelming initially due its use of strong colours at the top of the page. The context itself is well laid out and comprehensive and includes some nice illustrations and photos that help to reinforce the messages in the text. There are no distracting adverts but the text is quite complex and includes a lot of medical terminology.
77. ISK Institute	https://www.iskinstitute.com/kc/abdomen/rib_fractures/rib_fractures.html	This website is very text heavy and not particularly appealing. The font size and colouring make reading the content difficult. There are error messages indicating some of the content is not supported. There are no images to help illustrate the text and no adverts. The language is very complex and includes a lot of medical terms.
78. University Hospitals Plymouth NHS Trust	https://www.plymouthhospitals.nhs.uk/display-pil/pil-chest-wall-injury-6063/	This website is badged with the hospital and NHS logos which adds credibility to the information. The website provides a brief overview of chest wall injuries and how they are managed. There is also signposting information to make further contacts if required. There are no illustrations and no adverts. The language is clear and simple with limited medical terminology used.
79. Masnad Health Clinic	https://www.masnad.com.au/knowledge-centre/injury-hub/middle-back/rib-cartilage-injury/	There are a number of pop-ups on this page advertising for appointments and to leave reviews, which are immediately distracting. There are some illustrations, including one of the rib cage and some photos illustrating breathing and stretching exercises which are useful. The information focuses on rib cartilages injuries.

For peer review only

Name of Website	Website address	Website assessment
		The information is quite comprehensive but the language is sometimes complex and includes lot of medical terminology.
80. Royal Berkshire Hospital	https://www.royalberkshire.nhs.uk/media/jfcjgxef/chest-wall-injury_dec21.pdf	This weblinks takes you to a patient information leaflet about chest wall injury. It provides information to patients who have been discharged. It is badged with the hospital and NHS logos which adds credibility to the information. The information provides a brief overview of chest wall injuries and how they are managed. There is also signposting information to make further contacts if required. There are no illustrations and no adverts. The information is simple and avoid medical terminology.
81. Chest Wall Injury Society	https://cwisociety.org/patientportal/	This website is a patient portal for those who have had a chest wall injury. There is an 'open area' that provides information about considerations before having treatment. The focus is very patient focused and the majority of the information is provided in the form of individual patient video stories. There is also a member only area. There are no adverts or other illustrations. Despite being a patient portal, the text information provided is limited and although limited medical terminology is used the language is quite complex.
82. UMRC Rochester	https://www.urmc.rochester.edu/encyclopedia/content.aspx?ContentTypeID=22&ContentID=FlailChest	This website is linked to a US Medical Center, which adds credibility to the information provided about 'flail chest'. The webpage is clean and uncluttered but does not include any illustrations. The information provided is very complex and includes lots of medical terminology. No adverts.
83. Tufts Medical Center Community Care	https://hhma.org/healthadvisor/aha-ribinjur-sma/	This website provides brief information about rib injuries, symptoms and treatment. The webpage is largely clear an uncluttered but the image is not available. The language is simple and avoids the use of medical terminology. There are no distracting adverts.
84. Farrell Physiotherapy	https://farrellphysiotherapy.co.uk/conditions-treated/thoracic-spine/rib-fractures/	The top half of this website is mostly black with an underlying x-ray image, but it is very difficult to see. The information about rib fractures begins about half way down the page and a brief overview is provided on the causes and symptoms. Most of the information

For peer review only

Name of Website	Website address	Website assessment
		is focused on physiotherapy treatments provided by the clinic to help manage rib fractures. There is an image of a back showing the rib cage within, but the image is not helpful in supportiong the text content. The orientation of the text and the use of capitals makes navigation of the website difficult. The text itself is fairly simple and medical terminology use is limited. No adverts.
85. healthinfo.org.nz	https://www.healthinfo.org.nz/patientinfo/439381.pdf	The link takes you to a patient information leaflet about rib injuries. The leaflet is badged with health board logos which adds credibility to the information. It provides a brief overview about symptoms and self care and where to go for more help. The information is clear and simple and avoids the use of medical terminology. There is a single photo of a fallen skiier. There are no adverts.

For peer review only

Supplementary information. Table S3. Readability assessment of the 85 websites identified on Google™, Yahoo™ and Bing™.

Readability Assessments	Website														
	1. Medscape			2. UptoDate			3. Health Direct			4. Dynamed			5. Wikipedia		
	Grade (US)	Grade (UK)	Age	Grade (US)	Grade (UK)	Age	Grade (US)	Grade (UK)	Age	Grade (US)	Grade (UK)	Age	Grade (US)	Grade (UK)	Age
Flesch-Kincaid	13.4	14.4	17-18	14.7	15.7	18+	6.5	7.5	11-12	11.2	12.2	18+	11.9	12.9	16-17
Gunning Fog	16.6	17.6	18+	17.4	18.4	18+	9.9	10.9	14-15	10.9	11.9	18+	13.6	14.6	18+
Coleman-Liau	15.1	16.1	18+	15.8	16.8	18+	8.8	9.8	13-14	13.9	14.8	18+	15.2	16.2	18+
SMOG	15.6	16.6	18+	16.4	17.4	18+	10.3	11.3	15-16	10.7	11.7	18+	11.9	12.9	16-17
Automated Readability	12.5	13.5	17-18	14.4	15.4	18+3	6.1	7.1	11-12	9.4	10.4	18+	10.3	11.3	15-16
Median readability grade	15.1	16.1	17-18	15.8	16.8	18+	8.8	9.8	13-14	10.9	11.9	18+	11.9	12.9	16-17
Flesch Reading Ease	22.5	Very difficult		21.1	Very difficult		68.6	Plain English		24.4	Very difficult		21.8	Very Difficult	
% of general public readable to	59%			50%			100%			76%			71%		
**Readability Assessments**	**Website**														
	6. North Bristol NHS Trust			7. Drugs.com			8. Gloucestershire Hospitals NHS Trust			9. Very well health			10. MSD Manual consumer version		
	Grade (US)	Grade (UK)	Age	Grade (US)	Grade (UK)	Age	Grade (US)	Grade (UK)	Age	Grade (US)	Grade (UK)	Age	Grade (US)	Grade (UK)	Age
Flesch-Kincaid	6.7	7.7	12-13	5.6	6.6	11-12	4.6	5.6	10-11	4.8	5.8	10-11	8.7	9.7	14-15
Gunning Fog	9.0	10.0	14-15	8.1	8.2	13-14	6.4	7.4	11-12	6.4	7.4	11-12	10.8	11.8	16-17
Coleman-Liau	9.6	10.6	15-16	9.0	10.0	14-15	8.1	9.1	13-14	7.4	8.4	13-14	11.5	12.5	17-18
SMOG	10.0	11.0	15-16	9.3	10.3	14-15	7.9	8.9	13-14	8.4	9.4	13-14	8.0	9.0	13-14
Automated Readability	6.5	7.5	11-12	5.5	6.5	10-11	4.5	5.5	9-10	4.6	5.6	9-10	11.2	12.2	16-17
Median readability grade	9.0	10.0	14-15	8.1	9.1	13-14	6.4	7.4	11-12	6.4	7.4	11-12	10.8	11.8	16-17
Flesch Reading Ease	65.7	Plain English		69.4	Easy		71.3	Fairly easy		79.37	Fairly easy		51.4	Fairly difficult	
% of general public readable to	100%			100%			100%			100%			95%		
**Readability Assessments**	**Website**														
	11. The American Association for the Surgery of Trauma			12. LITFL			13. Radiology Key			14. NHS			15. Oxford University Hospitals NHS Trust		
	Grade (US)	Grade (UK)	Age	Grade (US)	Grade (UK)	Age	Grade (US)	Grade (UK)	Age	Grade (US)	Grade (UK)	Age	Grade (US)	Grade (UK)	Age
Flesch-Kincaid	12.5	13.5	17-18	12.0	13.0	17-18	12.6	13.6	17-18	4.5	4.5	10-11	6.7	7.7	12-13

Gunning Fog	16.0	17.0	18+	13.1	14.1	18+	15.4	16.4	18+	7.2	8.2	12-13	9.2	10.2	14-15
Coleman-Liau	14.7	15.7	18+	15.3	16.3	18+	14.1	15.1	18+	6.9	7.9	12-13	9.0	10.0	14-15
SMOG	14.5	15.5	18+	12.3	13.3	18+	14.4	15.4	18+	8.2	9.21	13-14	10.0	11.0	15-16
Automated Readability	11.4	12.4	16-17	10.5	11.5	15-16	11.9	12.9	17-18	4.2	5.2	9-10	7.0	8.0	12-13
Median readability grade	14.5	15.5	18+	12.3	13.3	18+	14.1	12.8	18+	6.9	7.9	12-13	9.0	10.0	14-15
Flesch Reading Ease	28.8	Difficult		24.1	Very difficult		32.6	Difficult		81.09	Easy		71.0	Fairly easy	
% of general public readable to	66%			70%			66%			100%			100%		
**Readability Assessments**	**Websites**														
	16. NICE			17. Aneurin Bevan University Health Board			18. My Health Alberta			19.Salisbury NHS Foundation Trust			20. Web MD		
	Grade (US)	Grade (UK)	Age	Grade (US)	Grade (UK)	Age	Grade (US)	Grade (UK)	Age	Grade (US)	Grade (UK)	Age	Grade (US)	Grade (UK)	Age
Flesch-Kincaid	7.5	8.5	12-13	6.8	7.8	12-13	4.7	5.7	12-13	7.2	8.2	12-13	7.5	8.5	13-14
Gunning Fog	10.7	11.7	16-17	9.2	10.2	14-15	7.1	8.2	14-15	9.2	10.2	14-15	7.7	8.7	13-14
Coleman-Liau	8.6	7.6	14-15	8.9	9.9	14-15	6.9	7.9	14-15	9.7	10.7	15-16	11.2	10.2	16-17
SMOG	10.9	11.9	16-17	10.0	11.0	15-16	8.3	9.3	15-16	10.3	11.3	15-16	10.1	11.1	15-16
Automated Readability	7.8	8.8	13-14	7.0	8.0	12-13	4.5	5.5	12-13	7.1	8.1	12-13	7.1	8.1	12-13
Median readability grade	8.6	9.6	14-15	8.9	9.9	14-15	6.9	7.9	14-15	9.2	10.2	14-15	7.7	8.7	13-14
Flesch Reading Ease	70.9	Fairly easy		71.0	Fairly easy		81.7	Easy		65.5	Plain English		54.9	Fairly difficult	
% of general public readable to	100%			100%			100%			100%			100%		
**Readability Assessments**	**Websites**														
	21. After Trauma			22. Royal Devon University Healthcare			23.South Tees Hospitals NHS Foundation Trust			24. Sirona Health and care			25. eMedicinehealth		
	Grade (US)	Grade (UK)	Age	Grade (US)	Grade (UK)	Age	Grade (US)	Grade (UK)	Age	Grade (US)	Grade (UK)	Age	Grade (US)	Grade (UK)	Age
Flesch-Kincaid	13.3	14.3	18+	5.0	6.0	10-11	7.3	8.3	12-13	4.7	5.7	10-11	7.1	8.1	12-13
Gunning Fog	15.4	16.4	18+	7.4	8.4	12-13	9.1	10.1	14-15	6.6	7.6	12-13	8.3	9.3	13-14
Coleman-Liau	12.4	13.4	18+	8.3	9.3	13-14	11.2	12.2	16-17	6.5	7.5	12-13	9.0	10.0	14-15
SMOG	14.1	15.1	18+	8.6	9.6	14-15	10.4	11.4	15-16	7.9	8.9	13-14	10.7	11.7	16-17
Automated Readability	11.1	12.1	18+	7.8	8.8	13-14	7.8	8.8	13-14	3.6	4.6	9-10	5.7	6.7	11-12
Median readability grade	13.3	14.3	18+	7.8	8.8	13-14	9.1	10.1	14-15	6.5	7.5	12-13	8.3	9.3	13-14
Flesch Reading Ease	42.0	Difficult		73.4	Fairly easy		61.6	Plain English		71.0	Fairly easy		61.6	Plain English	
% of general public readable to	100%			100%			100%			100%			100%		

For peer review only

**Readability Assessments**	**Website**														
	26. Better Health Channel			27. Rib Injury Clinic			28. Hull University Teaching Hospitals NHS Trust			29. Mount Nittany Health			30. MedlinePlus		
	Grade (US)	Grade (UK)	Age	Grade (US)	Grade (UK)	Age	Grade (US)	Grade (UK)	Age	Grade (US)	Grade (UK)	Age	Grade (US)	Grade (UK)	Age
Flesch-Kincaid	6.9	7.9	12-13	10.7	11.7	16-17	9.7	10.7	15-16	5.2	6.2	10-11	7.0	8.0	12-13
Gunning Fog	9.2	10.2	13-14	12.4	13.4	18+	11.3	12.3	16-17	7.2	8.2	12-13	7.1	8.1	12-13
Coleman-Liau	9.9	10.9	14-15	12.2	13.2	18+	11.4	12.4	16-17	7.8	8.8	13-14	10.7	11.7	16-17
SMOG	10.2	11.2	16-17	15.5	16.5	18+	12.6	13.6	18+	8.7	9.7	14-15	9.8	10.8	15-16
Automated Readability	6.6	7.6	11-12	10.4	11.4	13-17	10.0	11.0	15-16	4.7	5.7	10-11	6.7	7.7	12-13
Median readability grade	9.2	10.2	13-14	12.2	13.2	18+	11.3	12.3	16-17	7.2	8.2	12-13	7.1	8.1	12-13
Flesch Reading Ease	64.3	Plain English		47.0	Difficult		54.3	Fairly difficult		74.5	Fairly easy		55.9	Fairly difficult	
% of general public readable to	100%			80%			88%			100%%			100%		
**Readability Assessments**	**Website**														
	31. European Society of Thoracic Surgeons			32. Cleveland Clinic			33. mactheknife			34. World Rugby Passport			35. Total Vitality Medical Group		
	Grade (US)	Grade (UK)	Age	Grade (US)	Grade (UK)	Age	Grade (US)	Grade (UK)	Age	Grade (US)	Grade (UK)	Age	Grade (US)	Grade (UK)	Age
Flesch-Kincaid	11.0	12.0	16-17	6.3	7.3	11-12	12.7	13.7	18+	6.2	7.2	11-12	10.3	11.3	15-16
Gunning Fog	13.4	14.4	18+	8.6	9.6	14-15	16.0	17.0	18+	10.0	11.0	15-16	12.4	13.4	18+
Coleman-Liau	12.8	13.8	18+	9.6	10.6	15-16	13.4	14.4	18+	7.6	8.6	13-14	12.2	13.2	18+
SMOG	12.8	13.8	18+	9.8	10.8	15-16	15.0	16.0	18+	10.4	11.4	15-16	13.0	14.0	18+
Automated Readability	11.0	12.0	16-17	6.1	7.1	11-12	12.1	13.1	18+	5.9	6.9	11-12	10.9	11.9	16-17
Median readability grade	12.5	13.5	18+	8.6	9.6	14-15	13.4	14.4	18+	7.6	8.6	13-14	12.2	13.2	18+
Flesch Reading Ease	37.4	Difficult		66.4	Plain English		34.8	Difficult		75.5	Fairly easy		50.3	Fairly difficult	
% of general public readable to	78%			100%			65%			100%			83%		
**Readability Assessments**	**Website**														
	36. Healthline			37. NHS Lanarkshire			38. Cambridge University Hospitals NHS Foundation Trust			39. National University Hospital; National University Hospital System			40. The Rotherham NHS Foundation Trust		
	Grade (US)	Grade (UK)	Age	Grade (US)	Grade (UK)	Age	Grade (US)	Grade (UK)	Age	Grade (US)	Grade (UK)	Age	Grade (US)	Grade (UK)	Age
Flesch-Kincaid	8.6	9.6	14-15	6.0	7.0	11-12	6.9	7.9	12-13	8.1	9.1	13-14	8.1	9.1	13-14

For peer review only

Gunning Fog	11.4	12.4	16-17	7.8	8.8	13-14	10.1	11.2	15-16	9.1	10.1	14-15	10.5	11.5	16-17
Coleman-Liau	10.2	11.2	15-16	8.7	9.7	14-15	7.9	8.9	13-14	11.7	12.7	17-18	9.9	10.9	15-16
SMOG	11.7	12.7	17-18	9.7	10.7	15-16	10.4	11.4	15-16	11.1	12.1	16-17	11.1	12.1	16-17
Automated Readability	8.3	9.3	13-14	5.1	6.1	10-11	6.6	7.6	12-13	8.4	9.4	13-14	8.1	9.1	13-14
Median readability grade	10.2	11.2	15-16	7.8	8.8	13-14	7.9	8.9	13-14	9.1	10.1	14-15	9.9	10.9	15-16
Flesch Reading Ease	59.5	Fairly difficult		65.7	Plain English		71.7	Plain English		57.4	Fairly difficult		61.7	Plain English	
% of general public readable to	95%			100%			100%			99%			99%		
**Readability Assessments**	Website														
	41. Healthcare Associates of Texas			42. Physio-pedia			43. Medical News Today			44. My Family Physio			45. Banner Health		
	Grade (US)	Grade (UK)	Age	Grade (US)	Grade (UK)	Age	Grade (US)	Grade (UK)	Age	Grade (US)	Grade (UK)	Age	Grade (US)	Grade (UK)	Age
Flesch-Kincaid	7.8	8.8	13-14	11.9	12.9	17-18	7.4	8.4	12-13	10.3	11.3	15-16	8.1	9.1	13-14
Gunning Fog	9.2	10.2	14-15	13.4	14.4	18+	9.2	10.2	14-15	12.1	13.1	18+	10.2	11.2	15-16
Coleman-Liau	11.2	12.2	16-17	15.3	16.3	18+	10.3	11.3	15-16	13.0	14.0	18+	10.1	11.1	15.-16
SMOG	10.3	11.3	15-16	13.2	14.2	18+	10.1	11.1	15-16	12.7	13.7	18+	10.6	11.6	16-17
Automated Readability	8.0	9.0	13-14	11.9	12.9	17-18	6.8	7.8	12-13	10.2	11.2	15-16	8.5	9.5	14-15
Median readability grade	9.2	10.2	14-15	13.2	14.2		9.2	10.2	14-15	12.1	13.1	18+	10.1	11.1	15-16
Flesch Reading Ease	59.1			33.3	Difficult		59.0	Fairly difficult		45.6	Difficult		65.3	Plain English	
% of general public readable to	100%	Fairly difficult		71%			100%			83%			99%		
**Readability Assessments**	Website														
	46. Luna Physical Therapy			47. Healthwise			48. Wirral Community Health and Care NHS Foundation Trust			49. Barnsley Hospital NHS Foundation Trust			50. The Dudley Group NHS Foundation Trust		
	Grade (US)	Grade (UK)	Age	Grade (US)	Grade (UK)	Age	Grade (US)	Grade (UK)	Age	Grade (US)	Grade (UK)	Age	Grade (US)	Grade (UK)	Age
Flesch-Kincaid	8.2	9.2	13-14	5.9	6.9	11-12	5.2	6.2	10-11	5.0	6.0	10-11	4.8	5.8	10-11
Gunning Fog	10.7	11.7	16-17	8.2	9.2	13-14	7.4	8.4	12-13	7.4	8.4	12-13	6.5	7.5	11-12
Coleman-Liau	10.5	11.5	15-16	6.6	7.6	12-13	8.1	9.1	13-14	8.3	9.3	13-14	7.0	8.0	12-13
SMOG	11.0	12.0	16-17	8.9	9.9	14-15	9.0	10.0	14-15	8.8	9.8	14-15	8.3	9.3	13-14
Automated Readability	8.5	9.5	13-14	4.9	5.9	10-11	4.7	5.7	10-11	5.6	6.6	11-12	3.7	4.7	9-10
Median readability grade	10.5	11.5	15-16	6.6	7.6	12-13	7.4	8.4	12-13	7.4	8.4	12-13	6.5	7.5	11-12
Flesch Reading Ease	61.8	Plain English		76.9	Plain English		72.5	Fairly easy		77.7	Fairly easy		72.4	Plain English	

For peer review only

% of general public readable to	98%			100%			100%			100%			100%		
**Readability Assessments**	**Website**														
	51. York Hospitals			52. Bradford Teaching Hospitals NHS Foundation Trust			53. Milton Keynes University Hospital			54. Dorset County Hospital			55. Mount Sinai		
	Grade (US)	Grade (UK)	Age	Grade (US)	Grade (UK)	Age	Grade (US)	Grade (UK)	Age	Grade (US)	Grade (UK)	Age	Grade (US)	Grade (UK)	Age
Flesch-Kincaid	4.5	5.5	10-11	4.5	5.5	10-11	5.8	6.8	11-12	3.9	4.9	9-10	7.2	8.2	12-13
Gunning Fog	7.4	8.4	12-13	6.1	7.1	11-12	8.6	9.6	14-15	6.2	7.2	11-12	9.6	10.6	15-16
Coleman-Liau	7.8	8.8	13-14	7.0	8.0	12-13	8.3	9.3	13-14	6.1	7.1	11-12	9.6	10.6	15-16
SMOG	8.5	9.5	13-14	8.3	9.3	13-14	9.8	10.8	15-16	7.8	8.8	13-14	10.3	11.3	15-16
Automated Readability	4.6	5.6	10-11	4.1	5.1	9-10	5.0	6.0	10-11	3.0	4.0	8.9	6.9	7.9	12-13
Median readability grade	7.4	8.4	12-13	6.1	7.1	11-12	8.3	9.3	13-14	6.1	7.1	11-12	9.6	10.6	15-16
Flesch Reading Ease	78.8	Fairly easy		79.2	Fairly easy		68.9	Plain English		78.9	Fairly easy		65.2	Plain English	
% of general public readable to	100%			100%			100%			100%			100%		
**Readability Assessments**	**Website**														
	56. Yale Medicine			57. Oxford Health NHS Foundation Trust			58. Brigham and Women's Hospital			59. topdoctors.co.uk			60. Ventura Orthopedics		
	Grade (US)	Grade (UK)	Age	Grade (US)	Grade (UK)	Age	Grade (US)	Grade (UK)	Age	Grade (US)	Grade (UK)	Age	Grade (US)	Grade (UK)	Age
Flesch-Kincaid	7.6	8.6	13-14	4.5	5.5	10-11	9.8	10.8	15-15	9.0	10.0	14-15	7.0	8.0	12-13
Gunning Fog	9.3	10.3	14-15	6.8	7.8	12-13	12.9	13.9	18+	11.6	12.6	17-18	9.3	10.3	14-15
Coleman-Liau	9.9	10.9	15-16	5.7	6.7	11-12	12.3	13.3	18+	10.1	11.1	15-16	9.3	10.3	14-15
SMOG	10.3	11.3	15-16	7.8	8.8	13-14	12.6	13.6	18+	11.6	12.6	17-18	10.0	11.0	15-15
Automated Readability	7.5	8.5	13-14	2.8	3.8	8-9	9.3	10.3	14-15	8.9	9.9	14-15	7.0	8.0	12-13
Median readability grade	8.5	9.5	14-15	5.7	6.7	11-12	12.3	13.3	18+	10.1	11.1	15-16	9.3	10.3	14-15
Flesch Reading Ease	60.7	Plain English		72.6	Fairly easy		47.5	Difficult		59.2	Fairly difficult		68.2	Plain English	
% of general public readable to	95%			100%			86%			92%			100%		
**Readability Assessments**	**Website**														
	61. Nebraska Medicine			62. Dr Marco Scarci			63. Doncaster and Bassetlaw Teaching Hospitals			64. University Hospitals Coventry and Warwickshire			65. Orthopaedics and Trauma London		
	Grade (US)	Grade (UK)	Age	Grade (US)	Grade (UK)	Age	Grade (US)	Grade (UK)	Age	Grade (US)	Grade (UK)	Age	Grade (US)	Grade (UK)	Age

For peer review only

Flesch-Kincaid	8.9	9.9	14-15	9.9	10.9	15-15	3.1	4.1	8-9	4.9	5.9	10-11	10.2	11.2	15-16
Gunning Fog	11.2	12.2	16-17	12.3	13.3	18+	4.9	5.9	10-11	7.0	8.0	12-13	13.1	14.1	18+
Coleman-Liau	10.6	11.6	16-17	11.1	12.1	16-17	5.5	6.5	10-11	8.4	9.4	13-14	12.2	13.2	18+
SMOG	11.5	12.5	17-18	12.5	13.5	18+	6.9	7.9	12-13	9.0	10.0	14-15	12.8	13.8	18+
Automated Readability	9.0	10.0	14-15	9.9	10.9	15-16	2.5	3.5	8-9	5.1	6.1	10-11	10.1	11.1	15-15
Median readability grade	10.6	11.6	16-17	11.1	12.1	16-17	4.9	5.9	10-11	7.0	8.0	12-13	12.2	13.2	18+
Flesch Reading Ease	58.9	Fairly difficult		53.5	Fairly difficult		85.3	Easy		73.2	Fairly easy		48.9	Difficult	
% of general public readable to	93%			93%			100%			100%			84%		
**Readability Assessments**	**Website**														
	66. NCH Healthcare System			67. Physiocheck			68. Dr Gallagher and Partners			69. Agency for Clinical Innovation			70. SportsMD.com		
	Grade (US)	Grade (UK)	Age	Grade (US)	Grade (UK)	Age	Grade (US)	Grade (UK)	Age	Grade (US)	Grade (UK)	Age	Grade (US)	Grade (UK)	Age
Flesch-Kincaid	4.9	5.9	10-11	6.2	7.2	11-12	6.5	7.5	11-12	7.6	8.6	13-14	9.3	10.3	14-15
Gunning Fog	7.2	8.2	12-13	8.5	9.5	14-15	9.0	10.0	14-15	9.9	10.9	15-16	11.6	12.6	17-18
Coleman-Liau	7.2	8.2	12-13	7.7	8.7	13-14	9.2	10.2	14-15	11.2	12.2	16-17	11.8	12.8	17-18
SMOG	8.6	9.6	14-15	9.7	10.7	15-16	9.6	10.6	15-16	10.4	11.4	15-16	12.0	13.0	17-18
Automated Readability	4.3	5.3	9-10	5.1	6.1	10-11	5.6	6.6	11-12	7.5	8.5	13-14	9.6	10.6	15-16
Median readability grade	7.2	8.2	12-13	7.7	8.7	13-14	9.0	10.0	14-15	9.9	10.9	15-16	11.6	12.6	17-18
Flesch Reading Ease	76.8	Fairly easy		70.1	Fairly easy		63.9	Plain English		57.6	Fairly difficult		54.5	Fairly difficult	
% of general public readable to	100%			100%			100%			100%			90%		
**Readability Assessments**	**Website**														
	71. GP notebook			72. Upstate University Hospital			73. Fracture and Orthopedic Clinic			74. HealthyWA			75. St James's Hospital		
	Grade (US)	Grade (UK)	Age	Grade (US)	Grade (UK)	Age	Grade (US)	Grade (UK)	Age	Grade (US)	Grade (UK)	Age	Grade (US)	Grade (UK)	Age
Flesch-Kincaid	7.8	8.8	13-14	4.4	5.4	9-10	4.4	5.4	9-10	10.2	11.2	15-16	4.0	5.0	9-10
Gunning Fog	9.3	10.3	15-16	5.2	6.2	10-11	5.2	6.2	10-11	13.1	14.1	18+	6.6	7.6	12-13
Coleman-Liau	9.5	10.5	15-16	7.1	8.1	12-13	7.1	8.1	12-13	12.2	13.2	18+	7.3	8.3	12-13
SMOG	10.1	11.1	15-16	7.7	8.7	13-14	7.7	8.7	13-14	12.8	13.8	18+	8.0	9.0	13-14
Automated Readability	6.9	7.9	12-13	3.9	4.9	9-10	3.7	4.7	9-10	10.1	11.1	15-16	4.0	5.0	9-10
Median readability grade	9.3	10.3	15-16	5.2	6.2	10-11	5.2	6.2	10-11	12.2	13.2	18+	6.6	7.6	12-13
Flesch Reading Ease	59.3	Fairly difficult		74.5	Fairly easy		74.5	Fairly easy		48.9	Difficult		79.3	Fairly easy	

For peer review only

% of general public readable to	96%			100%			100%			84%			100%		
**Readability Assessments**	**Website**														
	76. International Centre for Thoracic Surgery			77. ISK Institute			78. University Hospitals Plymouth NHS Trust			79. Masnad Health Clinic			80. Royal Berkshire Hospital		
	Grade (US)	Grade (UK)	Age	Grade (US)	Grade (UK)	Age	Grade (US)	Grade (UK)	Age	Grade (US)	Grade (UK)	Age	Grade (US)	Grade (UK)	Age
Flesch-Kincaid	7.8	8.8	13-14	9.1	10.1	14-15	6.7	7.7	12-13	8.2	9.2	13-14	5.2	6.2	10-11
Gunning Fog	9.7	10.7	15-16	11.4	12.4	16-17	8.9	9.9	14-15	9.8	10.8	15-16	7.8	8.8	13-14
Coleman-Liau	10.4	11.4	15-16	10.7	11.7	16-17	8.5	9.5	13-14	10.1	11.1	15-16	8.3	9.3	13-14
SMOG	10.6	11.6	16-17	11.7	12.7	17-18	9.9	10.9	14-15	11.5	12.5	17-18	8.8	9.8	14-15
Automated Readability	7.7	8.7	13-14	9.7	10.7	15-16	5.7	6.7	11-12	7.9	8.9	13-14	5.0	6.0	10-11
Median readability grade	9.7	10.7	15-16	10.7	11.7	16-17	8.5	9.5	13-14	9.8	10.8	15-16	7.8	8.8	13-14
Flesch Reading Ease	61.00	Plain English		59.8	Fairly difficult		67.2	Plain English		60.8	Plain English		73.5	Fairly easy	
% of general public readable to	100%			92%			99%			99%					
**Readability Assessments**	**Website**														
	81. Chest Wall Injury Society			82. UMRC Rochester			83. Tufts Medical Center Community Care			84. Farrell Physiotherapy			85. healthinfo.org.nz		
	Grade (US)	Grade (UK)	Age	Grade (US)	Grade (UK)	Age	Grade (US)	Grade (UK)	Age	Grade (US)	Grade (UK)	Age	Grade (US)	Grade (UK)	Age
Flesch-Kincaid	8.9	9.9	14-15	9.8	10.8	15-16	6.2	7.2	11-12	7.6	8.6	13-14	4.7	5.7	10-11
Gunning Fog	10.7	11.7	16-17	12.7	13.7	18+	9.3	10.3	14-15	8.8	9.8	14-15	7.7	8.7	13-14
Coleman-Liau	13.6	14.6	18+	11.5	12.5	16-17	8.3	9.3	13-14	10.1	11.1	15-16	6.9	7.9	12-13
SMOG	12.3	13.3	18+	12.5	13.5	18+	10.0	11.0	15-16	9.9	10.9	15-16	8.6	9.6	14-15
Automated Readability	10.4	11.4	15-16	9.9	10.9	15-16	5.5	6.5	11-12	7.2	8.2	12-13	3.7	4.7	9-10
Median readability grade	10.7	11.7	16-17	11.5	12.5	16-17	8.3	9.3	13-14	8.8	9.8	14-15	6.9	7.9	12-13
Flesch Reading Ease	51.1	Fairly difficult		53.2	Fairly difficult		69.9	Plain English		61.8	Plain English		74.0	Fairly easy	
% of general public readable to	93%			87%			100%			100%			100%		

For peer review only

BMJ Open

A study to evaluate the readability and visual appearance of online resources for blunt chest trauma: an evaluation of online resources using mixed methods

Journal:	*BMJ Open*
Manuscript ID	bmjopen-2023-078552.R2
Article Type:	Original research
Date Submitted by the Author:	22-Dec-2023
Complete List of Authors:	Hutchings, Hayley; Swansea University, School of Medicine<br>Cochrane, Max; Swansea University, School of Medicine<br>Battle, Ceri; Swansea University, School of Medicine; Swansea Bay University Health Board, Physiotherapy Department
**Primary Subject Heading**:	Emergency medicine
Secondary Subject Heading:	Health services research
Keywords:	World Wide Web technology < BIOTECHNOLOGY & BIOINFORMATICS, Health Literacy, TRAUMA MANAGEMENT

SCHOLARONE™
Manuscripts

I, the Submitting Author has the right to grant and does grant on behalf of all authors of the Work (as defined in the below author licence), an exclusive licence and/or a non-exclusive licence for contributions from authors who are: i) UK Crown employees; ii) where BMJ has agreed a CC-BY licence shall apply, and/or iii) in accordance with the terms applicable for US Federal Government officers or employees acting as part of their official duties; on a worldwide, perpetual, irrevocable, royalty-free basis to BMJ Publishing Group Ltd ("BMJ") its licensees and where the relevant Journal is co-owned by BMJ to the co-owners of the Journal, to publish the Work in this journal and any other BMJ products and to exploit all rights, as set out in our licence.

The Submitting Author accepts and understands that any supply made under these terms is made by BMJ to the Submitting Author unless you are acting as an employee on behalf of your employer or a postgraduate student of an affiliated institution which is paying any applicable article publishing charge ("APC") for Open Access articles. Where the Submitting Author wishes to make the Work available on an Open Access basis (and intends to pay the relevant APC), the terms of reuse of such Open Access shall be governed by a Creative Commons licence – details of these licences and which Creative Commons licence will apply to this Work are set out in our licence referred to above.

Other than as permitted in any relevant BMJ Author's Self Archiving Policies, I confirm this Work has not been accepted for publication elsewhere, is not being considered for publication elsewhere and does not duplicate material already published. I confirm all authors consent to publication of this Work and authorise the granting of this licence.

For peer review only

A study to evaluate the readability and visual appearance of online resources for blunt chest trauma: an evaluation of online resources using mixed methods

Hayley A Hutchings[1*], Max Cochrane[1], Ceri E Battle[1,2].

[1]**Hayley Anne Hutchings**

https://orcid.org/0000-0003-4155-1741

Telephone: +44 (0) 1792 513412

Email: h.a.hutchings@swansea.ac.uk

[1]School of Medicine, Faculty of Medicine Health and Life Science, Singleton Park, Swansea SA2 8PP, U.K.

*Corresponding author

[1]**Max Cochrane**

Email: cochranemax1@gmail.com

[1]School of Medicine, Faculty of Medicine Health and Life Science, Singleton Park, Swansea SA2 8PP, U.K.

[1,2]**Ceri Elisabeth Battle**

Email: Ceri.Battle@wales.nhs.uk

https://orcid.org/0000-0002-7503-1931.

[2]Physiotherapy Department, Morriston Hospital, Swansea Bay University Health Board, Swansea, U.K.

For peer review only

For peer review only - http://bmjopen.bmj.com/site/about/guidelines.xhtml

Abstract

Objectives

Blunt chest trauma (BCT) is characterised by forceful and non-penetrative impact to the chest region. Increased access to the internet has led to online healthcare resources becoming used by the public to educate themselves about medical conditions. This study aimed to determine whether online resources for BCT are at an appropriate readability level and visual appearance for the public.

Design

We undertook a 1) a narrative overview assessment of the website; 2) a visual assessment of the identified website material content using an adapted framework of pre-determined key criteria based on the Centers for Medicare and Medicaid Services toolkit; and 3) a readability assessment utilising five readability scores and the Flesch reading ease score using Readable software.

Data Sources

Using a range of key search terms, we searched Google™, Bing™ and Yahoo™ websites on 9 October 2023 for online resources about BCT.

Results

We identified and assessed 85 websites. The median visual assessment score for the identified websites was 22, with a range of -14 to 37. The median readability score generated was 9 (14-15 years), with a range of 4.9 to 15.8. There was a significant association between the visual assessment and readability scores with a tendency for websites with lower readability scores having higher scores for the visual assessment (Spearman's rho= -0.485; p<0.01). The median score for Flesch reading ease was 63.9 (plain English) with a range of 21.1 to 85.3.

Conclusions

Although the readability levels and visual appearance was acceptable for the public for many websites, many of the resources had much higher readability scores than the recommended level (8-10) and visually were poor.

For peer review only

Better use of images would improve the appearance of websites further. Less medical terminology and shorter word and sentence length would also allow the public to comprehend the contained information more easily.

Strengths and Limitations

- We undertook a rigorous assessment of identified websites containing information on blunt chest trauma.
- We calculated extensive readability statistics for each website using five different readability scores and the Flesch reading ease score.
- We supplemented the readability assessment with a visual assessment using defined criteria adapted from the Centers for Medicare and Medicaid Services toolkit.
- We searched for relevant website materials using three search engines and restricted assessment to those websites identified on the first search page, therefore findings are limited to resources identified using these search engines.

Key words: world wide web technology, health literacy, trauma management

Funding Statement

This research received no specific grant from any funding agency in the public, commercial or not-for-profits sectors.

Conflicts of Interest

All authors confirm that there are no conflicts of interest to declare.

For peer review only - http://bmjopen.bmj.com/site/about/guidelines.xhtml

Introduction

Blunt chest trauma (BCT) is the leading cause of death among young adults, aged 15-44, and is second only to head trauma as the most common cause of death for all age groups[1]. The UK major trauma population is now reported to be more elderly, and the predominant mechanism is a fall from less than two metres[2].

Since the start of the century, internet usage across the globe has increased by 1,355%, with more than 5 billion internet users in the world as of 2023[3]. This huge increase in access to the internet has led to people using it as a healthcare resource, with 61% of Americans having looked up health-related information[4]. A study found that 62% of people considered healthcare information on the internet to be “excellent” or “very good”, and over half of people in the study felt they did not feel the need to share their findings with a doctor[5]. This suggests that many people see the internet as a valuable and accurate tool for healthcare. This number is likely to keep growing and this reliance on internet resources for all forms of healthcare means it is essential that information is accessible, accurate and readable.

Health literacy is defined as ‘a person’s ability to understand and use information to make decisions about their health’ and is believed to have a vital impact on public health due to access and use of health services[6, 7]. Between 43 and 61% of adults in the UK do not routinely understand health information[8], with more than 7 million adults having ‘very poor literacy skills’[9]. This means that they experience difficulties reading from unfamiliar sources. Similarly in the USA, the reading level of the average person is at an 8th grade level (age 12-13 years), meaning that they cannot interpret and evaluate any information that requires inference[10].

Low health literacy levels are a potential barrier to the use of online health resources. If the information is not able to be understood by a person without clinical knowledge, then this could

For peer review only - http://bmjopen.bmj.com/site/about/guidelines.xhtml

For peer review only

hinder early diagnosis and treatment of potentially life-threatening conditions. For this reason, it is essential that online resources are written at a low readability level, meaning that they are simple to interpret and understand.

Previous studies have found that online resources for healthcare are at a high readability level and are of poor quality[11-14] [15], showing that they contain medical terminology and are not visually appealing. For BCT, a study assessing the readability of a trauma surgery website found that the readability level was too high, and therefore difficult to comprehend[16].

One of the authors (CEB) has developed a predictive risk tool to help manage patients with BCT[17, 18] with one of the management pathways being to be sent home. It is important that patients understand any materials they are given. It is likely that such patients will access more materials online and as such we thought it was important to assess such materials. Apart from the study assessing the readability of the trauma surgery website, there has been no previous research undertaken assessing the quality and readability of online resources for BCT. The aim of this study was therefore to carry out an assessment of the readability and visual appearance of online resources for BCT.

Methods

Identifying online resources

To identify relevant websites we used the three most popular search engines in the UK: Google™, Bing™ and Yahoo™[19]. In order to capture the most relevant websites, we used a range of search terms. These were based on terms used in the existing literature. In order to capture more colloquial terms that would be familiar to patients, we also consulted with clinical colleagues who manage patients with chest trauma. Table 1 presents details of the full list of search terms used.

For peer review only - http://bmjopen.bmj.com/site/about/guidelines.xhtml

We identified all the websites that appeared on the first page of each search engine. We undertook: 1) a narrative overview assessment; 2) a readability assessment; and 3) a visual assessment of each website,

In order to evaluate the accessibility of the identified materials in terms of readability and visual aesthetics of online resources for BCT, we aimed to identify relevant websites that contained information intended to educate the public. We therefore excluded: research publications; books/chapters; clinical guidelines; teaching resources; clinical case studies; risk calculators; conference papers; newspaper articles; legal support services; referral information for clinicians; radio transcripts; sites solely for advertisement only; and sites where contact information only was provided. We further excluded sites where the content was clearly not relevant to the condition; private websites with restricted access; and sites with only video resources. Scientific papers, teaching resources and clinical guidelines are not resources developed specifically to educate and inform the public about BCT, while websites that require payment receive low traffic, with previous research finding that 80% of people who encounter a paywall when looking for health information will choose to search elsewhere[3]. We included Wikipedia, despite the fact that its content can be changed and hence may not contain accurate information[20]. We decided to include it as it is one of the most commonly accessed websites (currently seventh) and therefore it is important to assess its quality, due to the high level of traffic it receives[20, 21].

Narrative overview assessment We firstly produced a narrative overview assessment of each of the identified websites. We produced a paragraph that summarised our initial impressions of the

For peer review only - http://bmjopen.bmj.com/site/about/guidelines.xhtml

website outlining areas that were good or poor, prior to undertaking more detailed visual and readability assessments.

Visual assessment

We utilised the 'Guidelines for effective writing'[22], which details key areas that may affect the readability of websites in order to visually assess each website. These included headings, content organisation and language use, text size and colour, use of white space, and illustrations (for full list, see online Supplementary information Table S1).

We scored the visual assessment for each website. We compiled a list of 42 criteria for the assessment based on the 'Guidelines for effective writing'[22] . We selected these criteria as we judged them to be the most important elements when assessing the written content and visual appearance of the webpage. For each of these 42 criteria we assigned a score of +1 point if the criterion was achieved, 0 if the criterion was not applicable and -1 point if the criterion was not achieved. These points were added to give a maximum cumulative score of 42 for each website.

Readability assessment

When assessing the readability of each website, we used the website Readable (https://readable.com) to generate several readability scores for each online BCT resource. To do this, we entered each page of text that was considered relevant to BCT into Readable. Readable then generated various readability statistics. All text that appeared on any relevant page was entered into readable, including headings and lists. However, all images, graphs and navigation areas were removed for the purposes of calculating readability. These were however considered as part of the visual assessment of the websites.

For peer review only - http://bmjopen.bmj.com/site/about/guidelines.xhtml

When analysing the content of our chosen websites, we assessed all the relevant pages, up to a maximum of 10 pages. We only included pages that specifically discussed BCT or related injuries and disregarded any that contained other healthcare information or contact details.

We used five different readability formulae to give a wide evaluation of each website. There is currently no consensus regarding which readability formula is the most appropriate for assessing patient materials and it is therefore recommended that more than one formula is used to assess such materials[23] Each formula assesses the text in a different way and includes items such as words, characters, and syllables[12]. Employing a range of scores therefore improves the validity of the results[23]. We used the readability formulae: Flesch-Kincaid Grade Index[24], Coleman-Liau Index[25], Simplified Measure of Gobbledygook Index[26], Gunning-Fog Index[27] and the Automated Readability Index[28]. We chose these readability formulae as they have previously been used to assess readability within the medical field[15, 16, 23, 29-31].

In addition to calculating the readability formulae, we also calculated the percentage of the population the text could be read by and the Flesch Reading Ease score[32]. The Flesch Reading Ease (FRE) score is the earliest of the commonly used tools to assess readability[23, 31] and gives a score on a scale ranging from 0–100. A score of 0 is classified as being unreadable and that of 100 the most readable[31, 32]. It is based on the average number of syllables per word and the average number of words per sentence. Content with a score of 70 is easy to read for most of the population. Text with shorter sentences and simpler words will have a higher score than text with longer sentences and more complex words[32]. We utilised the FRE score to provide a summary score of the accessibility of the text within the identified websites.

For peer review only - http://bmjopen.bmj.com/site/about/guidelines.xhtml

For peer review only

Readable generated scores as a school grade level, which was then translated into a corresponding age. This is the age that could be expected to comprehend the piece of writing. Various healthcare organisations have recommended that readability levels should be between 6th and no more than 8th grade (age 11-14 years)[33-35].

Changes made to original protocol

Following peer review feedback from reviewers the original protocol was expanded to increase the breadth of websites reviewed. The search terms used were expanded to include terms that were more likely to be understood and used by patients. Our original protocol only used the terms: 'blunt chest trauma', 'blunt chest injury', 'chest impact injury', 'chest trauma', 'thoracic contusion' and 'forceful chest injury'. We also assessed all relevant websites identified on the first page of each search engine rather than the top ten. In our original protocol we undertook a limited visual assessment utilising only 10 criteria. We modified this undertake a comprehensive assessment utilising 42 criteria following review.

Statistical analysis

The readability statistics were generated for each page of the website used. A median (range) readability score was calculated based on the five readability formulae. Where more than one page was assessed for a website, scores were aggregated to give a median score for each formula that was representative of the entire website.

We compared readability scores with the cumulative score from the visual assessment to determine if there was any correlation between readability and visual appearance, using non-parametric Spearman correlation. A p-value of less than 0.05 was regarded as being statistically significant[36].

Patient and Public Involvement

For peer review only - http://bmjopen.bmj.com/site/about/guidelines.xhtml

Due to the limited time available to complete this project and lack of funding, it was not possible to involve patients or the public in the design, or conduct, or reporting, or dissemination plans of our research.

Results

Identification of online resources

Using the search terms across the first page of the three search engines, we identified a total of 258 websites. We identified a large amount of repetition of websites and a large number of websites were excluded based on applying our pre-defined search criteria (see Figure 1). There were a large number of scientific papers identified, especially when using more clinical search terms such as 'thoracic contusion'. When using search terms that were less scientific such as 'bruised' or 'broken ribs', more online resources that appeared that were more suitable for use by the public. After applying exclusions, we identified a total of 85 websites for further assessment.

Narrative overview assessment

Supplementary information Table S2 details the online resources identified, with a brief written assessment of their content. Many of the resources that we identified were not solely based on BCT. Instead, they contained sections that were relevant to BCT as well as information on either other types of chest injury, or trauma injuries affecting the head or abdomen. Similarly, some of the resources were focused on types of injury caused by BCT, such as pneumothorax, flail chest and rib fractures. Most of the information identified was contained within a single webpage. There was limited use of photographs and illustrations that could have potentially improved the visual aesthetics of the website and understanding of the information. Many of the websites linked to simple patient information leaflets that focused on advice for patients to help manage chest injuries/rib fractures/bruising with signposting for further help should they need it. Many sites linked to the National Health Services (NHS) (for example general practice surgeries and secondary

For peer review only - http://bmjopen.bmj.com/site/about/guidelines.xhtml

care hospitals) simply replicated the information from the NHS site and added their own branding. Overall, there was very limited, patient focused information identified for BCT.

Visual assessment

Details of the visual assessment for each of the 85 online resources are shown in Supplementary information Table S1. There was a large amount of variability across the websites. The median visual assessment score was 22 with a range from -14 to 37 out of a maximum possible score of 42. More than 50% of the websites achieved a score of 22 or less (45/85 websites; 52.9%). This means that most websites either did not consider or scored negatively on almost half of the criteria identified as important based on the Centers for Medicare and Medicaid Services Toolkit for Making Written Material Clear and Effective[22] . This indicates that most websites could do more to make their websites more visually appealing.

The lowest scoring resource was the ISK Institute website, with an overall score of -14from a possible 42. Other poorly scoring websites with a score of less than 10 were: World Rugby Passport (-3); GP notebook (6); Wikipedia (6); Physiocheck (7); Farrell Physiotherapy (8); Orthopaedics and Trauma London (8); and DynaMed (8). Poorly scoring websites appeared cluttered with too much complex information and extensive use of medical terminology, poor use of colouring and contrast, distracting adverts, and limited or inappropriate use of illustrations. Many of these websites seem to be advertising services and therefore appear less credible.

The highest scoring websites was the Royal Devon University Healthcare NHS Foundation Trust & Northern Devon Healthcare NHS Trust with 37. There were five other websites that scored highly on their visual assessment with a score of 32 or more (Cleveland Clinic (32); Dr Gallagher and Partners (32); Bradford Teaching Hospitals NHS Foundation Trust (34); healthinfo.org.nz (34); Agency for Clinical Innovation (36)). Highly scoring websites had a clean and uncluttered appearance with lots

For peer review only - http://bmjopen.bmj.com/site/about/guidelines.xhtml

of white space, sparing use of colour and good contrast of colours, easy to understand information with limited use of medical terminology, no adverts, and appropriate use of illustrations that supported the text content. These websites largely were associated with credible organisations such as the NHS or other government bodies, which helps to reassure the public that the information is more credible.

Readability assessment

Supplementary information Table S3 provides detailed information on the readability assessment. Most websites only had one page of information on which to undertake a readability assessment. The median readability score across all websites was 9 , which equates to a reading age of 14-15 years of age. The range of scores was from 4.9 (10-11 years of age) to 15.8 (18+ years of age, or University level). Aiming for a readability grade of a maximum of 8[33-35], only 30 of the 85 websites identified (35.3%) were at an appropriate reading level for the general public.

The median Flesch Reading Ease score was 63.9 (Plain English) with a range between 21.1 (very difficult) for the UpToDate website, to 85.3 (easy) for the Doncaster and Bassetlaw Teaching Hospitals website. This is understandable given that the UpToDate website uses more medical terminology and seems to be directed at clinicians, whereas the Doncaster and Bassetlaw Teaching Hospitals website is a resource created for patients/the public. Only two additional websites were also classified as easy to read with a Flesch Reading Ease Score of more than 80 (NHS (81.1); My Health Alberta (81.7)). Aiming for a Flesch reading ease score of greater than 70[32] only 29/85 (34.1%) were accessible for most of the population.

In terms of reach and the percentage of the addressable audience that each website resource was readable to, scores ranged from 50% for the website UpToDate, to 100%, which was scored by more over 60% (52/85) of the websites.

For peer review only - http://bmjopen.bmj.com/site/about/guidelines.xhtml

Comparison between visual assessment and readability scores

Figure 2 shows the median readability score and the visual assessment score for each online resource. There was a large amount of variation between the websites for both categories. Some websites performed well in the readability assessment but badly in the visual assessment and vice versa. There was a statistically significant association between the visual assessment and readability scores with a tendency for websites with lower readability scores (i.e. easier to read) having higher (i.e. better) scores for the visual assessment (Spearman's rho= -0.485; $p<0.01$). This suggests that there is a correlation between websites being difficult to read and being visually unappealing. A low readability score and a high visual assessment score is most desirable.

Discussion

Overall, we found that online resources for BCT were generally visually appealing, but the content was written at a level too high for the public to understand. This shows resources available for public access are not providing accessible information. This may lead to a misunderstanding of the information being provided, and consequentially could result in slower diagnosis and greater risk of hospitalisation.

To be deemed readable for the general public, a readability grade score of between 6 and 8 should be achieved, which equates to a reading age of between 11 and 14 years. Eighth grade is the average reading age of an American adult[10], so if the readability score of online resources are any higher than this, the information contained in the online resource will be inaccessible to more than half of the general public. We calculated a median readability score for the identified BCT resources of 9 (age 14-15 years), which is higher than the recommended score of 8. Only 35% of the website had a readability of less than 8[34, 35], which suggests that online resources for BCT are not suitable for educating the public. This is supported by the fact that only three websites were classified as easy to

For peer review only - http://bmjopen.bmj.com/site/about/guidelines.xhtml

read with a score of more than 80 when considering the Flesch Reading Ease score[32], with only 34% being accessible for most of the population.

Our findings concur with previous research assessing readability of online resources for other health conditions, such as phenylketonuria, fibroadenoma, otolaryngology and parathyroidectomy, which all identified that readability levels were too high[11-14]. This suggests that readability issues are common in online health resources, which could be a reason for up to 61% of UK adults not routinely understanding health information[8].

These finding suggest that it is essential for those writing online resources for BCT to consider their target audience, and to ensure that the content of the website is accessible and understandable for the public. Possible considerations to improve public accessibility include the use of shorter words and sentences and the use of less complex words and medical terminology throughout the text. Previous studies have demonstrated that shortening sentences to less than 15 words led to an improvement in readability[37]. In certain scenarios where using more complex terms is necessary, brief explanations or definitions can also be helpful to the reader. All these factors can contribute to the production of more readable online resources for BCT, leading to increased levels of education for the public regarding their health.

For the visual assessment of BCT resources, a median score of 22/42 was recorded and a large number of the websites were generally well laid out and visually appealing. However, with no website scoring 42 and with more than 50% of websites scoring less than 22 this indicates that there are still ways in which the visual appearance of these websites can be improved.

It is important for the online resource to be appealing as this is the first thing a user will see and may impact whether they choose to continue to read the information present on the webpage[38-40].

For peer review only - http://bmjopen.bmj.com/site/about/guidelines.xhtml

Even if a resource for BCT contains accurate and helpful information written at an appropriate readability level for a member of the public, not having a visually appealing page may cause the reader to look elsewhere.

In designing a website for BCT education, guidance can be taken from the 'guidelines for effective writing' from the Centers for Medicare and Medicaid Services[22]. This will provide guidance on how best to structure text, with the use of paragraphing and headings essential to break up text into more manageable sections for readers. Using bullet-points to highlight key information is also beneficial to the reader, being clear and concise[41]. This is more appealing than having to search for information in a large block of text.

It is important to consider making information accessible to people who may have poor eyesight or have problems with reading. The use of images and audio aids can be effective in communicating BCT information to these audiences. The online resources assessed in this study demonstrated limited use of images or diagrams, suggesting that less importance is placed on visual aids compared to written information. Previous research has found that the use of audio and visual aids have a significant impact on learning[42]. This further highlights the importance of using these aids in future online resources for BCT.

Many websites only partially discussed BCT or focused more on injuries that could be caused by BCT. In addition, several websites were aimed at audiences with more advanced scientific knowledge, however this was not clearly identifiable when the website was first accessed. These issues could both lead to confusion for the reader and dissuade them from looking for online information for BCT in the future. Therefore, it is important for future online resources to not only be visually appealing and written at an appropriate level, but to be clearly identifiable as resources intended for use by the public.

For peer review only - http://bmjopen.bmj.com/site/about/guidelines.xhtml

For peer review only

There are some limitations to this study. We employed a range of search terms, including many that would be more familiar to the public. However, a larger range of search terms could have been used, which may have identified more online resources. A larger number of resources could also have been assessed. However, since the three search engines we chose to use receive over 95% of traffic, and that 95% of people do not go past the first page of results[43] we believe that we identified the websites that would receive almost all the traffic. We also identified a lot of replication in the 258 identified websites within the first page search, which is likely to increased further past the first page of searches. Apart from assessing some criteria relating to content accuracy as part of the visual assessment (such as author, use of references and website updates), we did not undertake a full assessment of the accuracy of the website content. The purpose of the study was to undertake an initial assessment of the readability and visual appearance of online materials for access by members of the public/patients. It was beyond the scope of the study to assess website content and therefore further work is therefore needed to assess the accuracy of the materials.

When assessing readability and visual quality of the chosen resources, we only used 5 readability formulae, all of which assessed the readability based on sentence and word length and complexity. This did not consider other factors such as tables and diagrams that could also affect how easily a website can be read. Even though these were considered in the visual assessment, evaluating tables and figures only formed a small part of the visual assessment. A specific examination of visual and audio aids would be useful for future research to consider when assessing the quality of online resources.

Some websites with only a small amount of information may demonstrate an overly high readability score, and not be as representative of the overall readability of the website as those in which

For peer review only - http://bmjopen.bmj.com/site/about/guidelines.xhtml

multiple pages of information were assessed. However, if someone is searching for BCT information, it is less important to them how readable other sections of the websites are.

Further research is necessary, assessing specific aspects of visual appearance such as images in detail. Deeper analysis into the accuracy of the scientific content of the websites could also be useful, and other online resources such as videos or audio content should be considered.

Conclusions

We found that online resources for BCT were written at a level too advanced for use by the public, with a reading age greater than recommended. The visual appearance of these resources was generally at a level acceptable to the public. BCT online resources could however be made more accessible and improved for public use by reducing the reading age of the textual content, and by considering additional criteria to improve visual aesthetics, such as the use of images

Total word count: 4131

Ethics Statement

Patient Consent for Publication

Not applicable

Ethics Approval

Not applicable.

Author Contributions

HAH designed the original study, quality assured the data collection and analysis, provided supervisory support and drafted the manuscript. MC adapted the study design, undertook the data searches, analysed the data, drafted data outputs and reviewed the final manuscript. CEB provided

For peer review only - http://bmjopen.bmj.com/site/about/guidelines.xhtml

input into study design, provided clinical advisory support to the project, quality assured the outputs and reviewed the final manuscript.

Data availability

All original data from the study are available in the zenodo repository[36].

For peer review only

For peer review only - http://bmjopen.bmj.com/site/about/guidelines.xhtml

References

1. Fantus RJ, Clark DE. NTDB(R) data points: Annual report 2007: more than 2.7 in 2007. Bull Am Coll Surg. 2008;93(1):62-3. PubMed PMID: 23505678.
2. Kehoe A, Smith JE, Edwards A, Yates D, Lecky F. The changing face of major trauma in the UK. Emerg Med J. 2015;32(12):911-5. doi: 10.1136/emermed-2015-205265. PubMed PMID: 26598629; PubMed Central PMCID: PMCPMC4717354.
3. Broadband Search. Key internet statistics in 2023 (including mobile). 2023 [28 July 2023]. Available from: https://www.broadbandsearch.net/blog/internet-statistics
4. Fox S, Jones S. The Social Life of Health Information. Americans' pursuit of health takes place within a widening network of both online and offline sources Washington: Pew Internet and American Life Project; 2009 [28 July 2023]. Available from: https://www.pewresearch.org/internet/2009/06/11/the-social-life-of-health-information/
5. Diaz JA, Griffith RA, Ng JJ, Reinert SE, Friedmann PD, Moulton AW. Patients' use of the Internet for medical information. J Gen Intern Med. 2002;17(3):180-5. doi: 10.1046/j.1525-1497.2002.10603.x. PubMed PMID: 11929503; PubMed Central PMCID: PMCPMC1495021.
6. NHS Digital Service Manual. Design and build digital services for the NHS. 2023 [28 July 2023]. Available from: https://service-manual.nhs.uk.
7. Vandenbosch J, Van den Broucke S, Vancorenland S, Avalosse H, Verniest R, Callens M. Health literacy and the use of healthcare services in Belgium. J Epidemiol Community Health. 2016;70(10):1032-8. Epub 20160426. doi: 10.1136/jech-2015-206910. PubMed PMID: 27116951.
8. Berry J. Does health literacy matter? : NHS England; 2016 [28 July 2023]. Available from: https://www.england.nhs.uk/blog/jonathan-berry/.
9. National Literacy Trust. What is Literacy? [28 July 2023]. Available from: https://literacytrust.org.uk/information/what-is-literacy/
10. National Centre for Education Statistics. Program for the International Assessment of Adult Competencies (PIAAC) 2023 [28 July 2023]. Available from: https://nces.ed.gov/surveys/piaac/
11. Byrne J, Keogh S, Cullinane C, Razzaq Z, Redmond HP. Readability and Quality of Online Health Information Regarding Parathyroidectomy. OTO Open. 2022;6(4):2473974X221133308. Epub 20221020. doi: 10.1177/2473974X221133308. PubMed PMID: 36311181; PubMed Central PMCID: PMCPMC9597036.
12. Hutchings HA, Remesh A. An evaluation of the readability and visual appearance of online patient resources for fibroadenoma. PLoS One. 2022;17(11):e0277823. Epub 20221118. doi: 10.1371/journal.pone.0277823. PubMed PMID: 36399450; PubMed Central PMCID: PMCPMC9674151.
13. Marsh JM, Dobbs TD, Hutchings HA. The readability of online health resources for phenylketonuria. J Community Genet. 2020;11(4):451-9. Epub 20200327. doi: 10.1007/s12687-020-00461-9. PubMed PMID: 32221843; PubMed Central PMCID: PMCPMC7475157.
14. Grose EM, Holmes CP, Aravinthan KA, Wu V, Lee JM. Readability and quality assessment of internet-based patient education materials related to nasal septoplasty. J Otolaryngol Head Neck Surg. 2021;50(1):16. Epub 20210317. doi: 10.1186/s40463-021-00507-z. PubMed PMID: 33731227; PubMed Central PMCID: PMCPMC7970817.
15. Martin CA, Khan S, Lee R, Do AT, Sridhar J, Crowell EL, et al. Readability and Suitability of Online Patient Education Materials for Glaucoma. Ophthalmol Glaucoma. 2022;5(5):525-30. Epub 20220314. doi: 10.1016/j.ogla.2022.03.004. PubMed PMID: 35301989.
16. Eltorai AE, Ghanian S, Adams CA, Jr., Born CT, Daniels AH. Readability of patient education materials on the american association for surgery of trauma website. Arch Trauma Res. 2014;3(2):e18161. Epub 20140430. doi: 10.5812/atr.18161. PubMed PMID: 25147778; PubMed Central PMCID: PMCPMC4139691.

For peer review only - http://bmjopen.bmj.com/site/about/guidelines.xhtml

17. Battle CE, Hutchings H, Evans PA. Risk factors that predict mortality in patients with blunt chest wall trauma: a systematic review and meta-analysis. Injury. 2012;43(1):8-17. Epub 20110122. doi: 10.1016/j.injury.2011.01.004. PubMed PMID: 21256488.
18. Battle C, Hutchings H, Lovett S, Bouamra O, Jones S, Sen A, et al. Predicting outcomes after blunt chest wall trauma: development and external validation of a new prognostic model. Crit Care. 2014;18(3):R98. Epub 20140514. doi: 10.1186/cc13873. PubMed PMID: 24887537; PubMed Central PMCID: PMCPMC4095687.
19. Search Engine Insight. Top 10 Search Engines In The World (2022 Update) [31 July 2023]. Available from: https://www.searchengineinsight.com/top-10-leading-search-engines-uk/.
20. Azer SA. Is Wikipedia a reliable learning resource for medical students? Evaluating respiratory topics. Adv Physiol Educ. 2015;39(1):5-14. doi: 10.1152/advan.00110.2014. PubMed PMID: 25727464.
21. Similarweb. Top Websites Ranking. Available from: https://www.similarweb.com/top-websites/ (Access date 20 November 2023) 2023.
22. CMS.gov, Centers for Medicare and Medicaid Services. Toolkit for Making Written Material Clear and Effective. [31 July 2023]. Available from: https://www.cms.gov/outreach-and-education/outreach/writtenmaterialstoolkit.
23. Badarudeen S, Sabharwal S. Assessing readability of patient education materials: current role in orthopaedics. Clin Orthop Relat Res. 2010;468(10):2572-80. doi: 10.1007/s11999-010-1380-y. PubMed PMID: 20496023; PubMed Central PMCID: PMCPMC3049622.
24. Kincaid JP, Fishburne RP, Rogers RL, Chissom BS. Derivation of new readability formulas (automated readability index, fog count, and flesch reading ease formula) for Navy enlisted personnel. Research Branch Report 8–75. Available from: https://apps.dtic.mil/dtic/tr/fulltext/u2/a006655.pdf (access date 22 January 2020). Naval Air Station Memphis: 1975.
25. Coleman M, Liau TL. A computer readability formula designed for machine scoring. Journal of Applied Psychology. 1975;60:283-4.
26. McLaughlin GH. SMOG Grading — a New Readability Formula. Journal of Reading. 1969;12(8):639-46.
27. Gunning R. The Technique of Clear Writing: McGraw-Hill; 1952.
28. Smith EA, Senter RJ. Automated Readability Index. AMRI.-TR-66-220. Available from: https://apps.dtic.mil/dtic/tr/fulltext/u2/667273.pdf (access date 13 January 2020). Defense Documentation Center, Virginia: Wright-Patterson Air Force Base, Ohio, 1967.
29. Dobbs T, Neal G, Hutchings HA, Whitaker IS, Milton J. The Readability of Online Patient Resources for Skin Cancer Treatment. Oncol Ther. 2017;5:149-60. doi: 10.1007/s40498-017-0051-5.
30. Phillips NA, Vargas CR, Chuang DJ, Lee BT. Readability Assessment of Online Patient Abdominoplasty Resources. Aesthetic Plast Surg. 2015;39(1):147-53. doi: 10.1007/s00266-014-0425-0. PubMed PMID: 25476602.
31. Friedman DB, Hoffman-Goetz L. A systematic review of readability and comprehension instruments used for print and web-based cancer information. Health Educ Behav. 2006;33(3):352-73. doi: 10.1177/1090198105277329. PubMed PMID: 16699125.
32. Interpretation of Flesch Reading Ease Score (Access date 16 August 2022). Available from: https://writingstudio.com/blog/flesch-reading-ease/.
33. Wolf MS, Cooper Bailey S. The Role of Health Literacy in Patient Safety. Available from: https://psnet.ahrq.gov/perspective/role-health-literacy-patient-safety (Acces date 20 November 2023): PS Net; 2009.
34. Cotugna N, Vickery CE, Carpenter-Haefele KM. Evaluation of literacy level of patient education pages in health-related journals. J Community Health. 2005;30(3):213-9. doi: 10.1007/s10900-004-1959-x. PubMed PMID: 15847246.
35. The Joint Commission. "What Did the Doctor Say?:" Improving Health Literacy to Protect Patient Safety. Available from:

For peer review only - http://bmjopen.bmj.com/site/about/guidelines.xhtml

https://swselfmanagement.ca/uploads/ResourceDocuments/The%20Joint%20Commission%20(2007)%20Improving%20Health%20Literacy%20for%20Patient%20Safety.pdf (Access date 20 November 2023). 2007.
[dataset]36. Hutchings HA, Cochrane M, Battle CE. A study to evaluate the readability and visual appearance of online resources for blunt chest trauma: an evaluation of online resources using mixed methods Available from: https://zenodo.org. 2023.
37. Sheppard ED, Hyde Z, Florence MN, McGwin G, Kirchner JS, Ponce BA. Improving the readability of online foot and ankle patient education materials. Foot Ankle Int. 2014;35(12):1282-6. Epub 20140919. doi: 10.1177/1071100714550650. PubMed PMID: 25239196.
38. Hesse BW, Moser RP, Rutten LJ. Surveys of physicians and electronic health information. N Engl J Med. 2010;362(9):859-60. doi: 10.1056/NEJMc0909595. PubMed PMID: 20200398.
39. Woloshin S, Yang Y, Fischhoff B. Communicating health information with visual displays. Nat Med. 2023;29(5):1085-91. Epub 20230508. doi: 10.1038/s41591-023-02328-1. PubMed PMID: 37156935.
40. Szmuda T, Ozdemir C, Ali S, Singh A, Syed MT, Sloniewski P. Readability of online patient education material for the novel coronavirus disease (COVID-19): a cross-sectional health literacy study. Public Health. 2020;185:21-5. Epub 20200530. doi: 10.1016/j.puhe.2020.05.041. PubMed PMID: 32516624; PubMed Central PMCID: PMCPMC7260546.
41. Gov.uk. Style guide [3 August 2023]. Available from: https://www.gov.uk/guidance/style-guide/a-to-z-of-gov-uk-style.
42. Carey M, Schofield P, Jefford M, Krishnasamy M, Aranda S. The development of audio-visual materials to prepare patients for medical procedures: an oncology application. Eur J Cancer Care (Engl). 2007;16(5):417-23. doi: 10.1111/j.1365-2354.2006.00772.x. PubMed PMID: 17760928.
43. Protofuse. The First Page Of Google, By The Numbers [3 August 2023]. Available from: https://protofuse.com/articles/first-page-of-google-stats/.

For peer review only - http://bmjopen.bmj.com/site/about/guidelines.xhtml

Figure 1. Flow diagram illustrating number of websites identified and reasons for exclusions.

Figure 2. Median readability score vs visual assessment score for the 85 assessed websites.

For peer review only

For peer review only - http://bmjopen.bmj.com/site/about/guidelines.xhtml

Tables

For peer review only

For peer review only - http://bmjopen.bmj.com/site/about/guidelines.xhtml

Table 1. List of search terms used to undertake searches in Google™, Bing™ and Yahoo™.

blunt chest trauma
blunt chest injury
chest impact injury
chest trauma
thoracic contusion
forceful chest injury
chest injury
broken ribs
rib fracture
chest and rib injury
chest wall injury
rib injuries
bruised ribs

For peer review only

For peer review only - http://bmjopen.bmj.com/site/about/guidelines.xhtml

Figure 1. Flow diagram illustrating number of websites identified and reasons for exclusions.

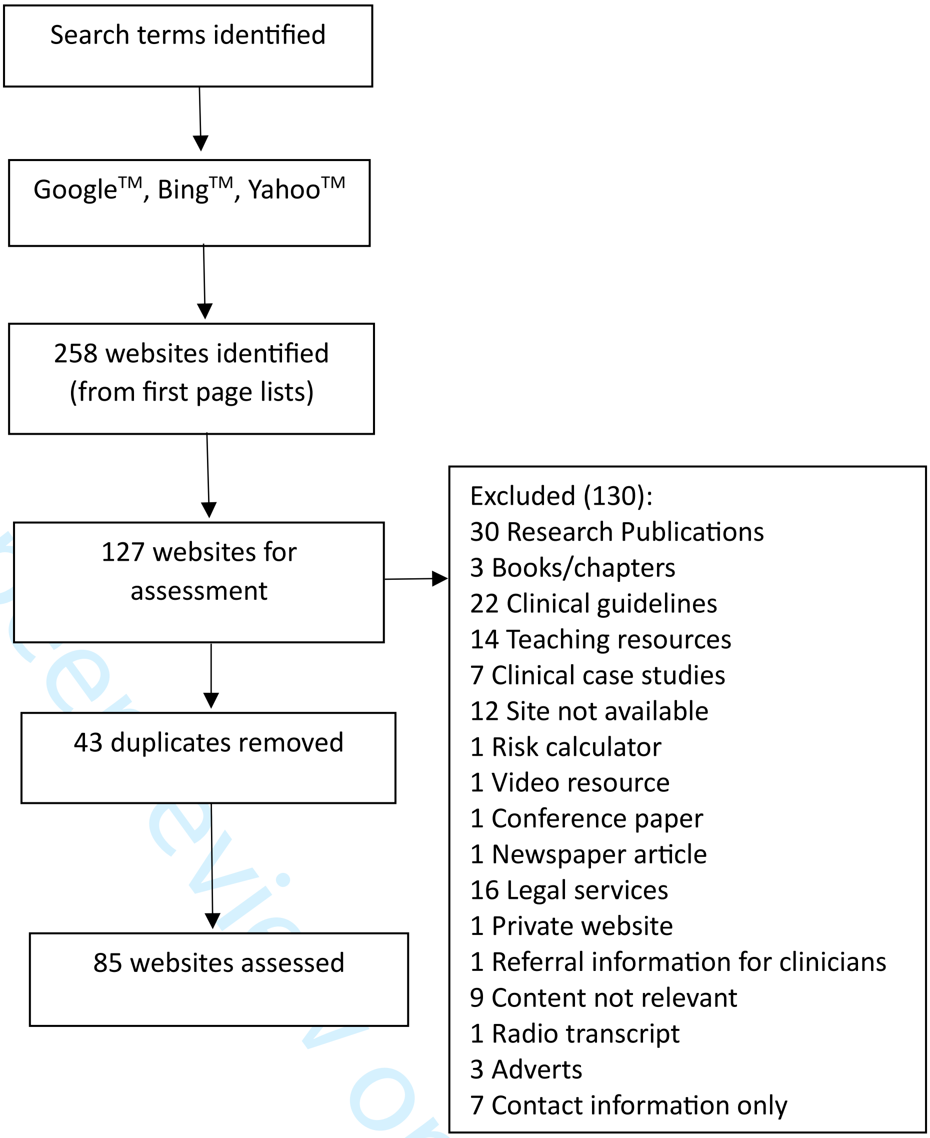

For peer review only

Figure 2. Median readability score vs visual assessment score for the 85 assessed websites.

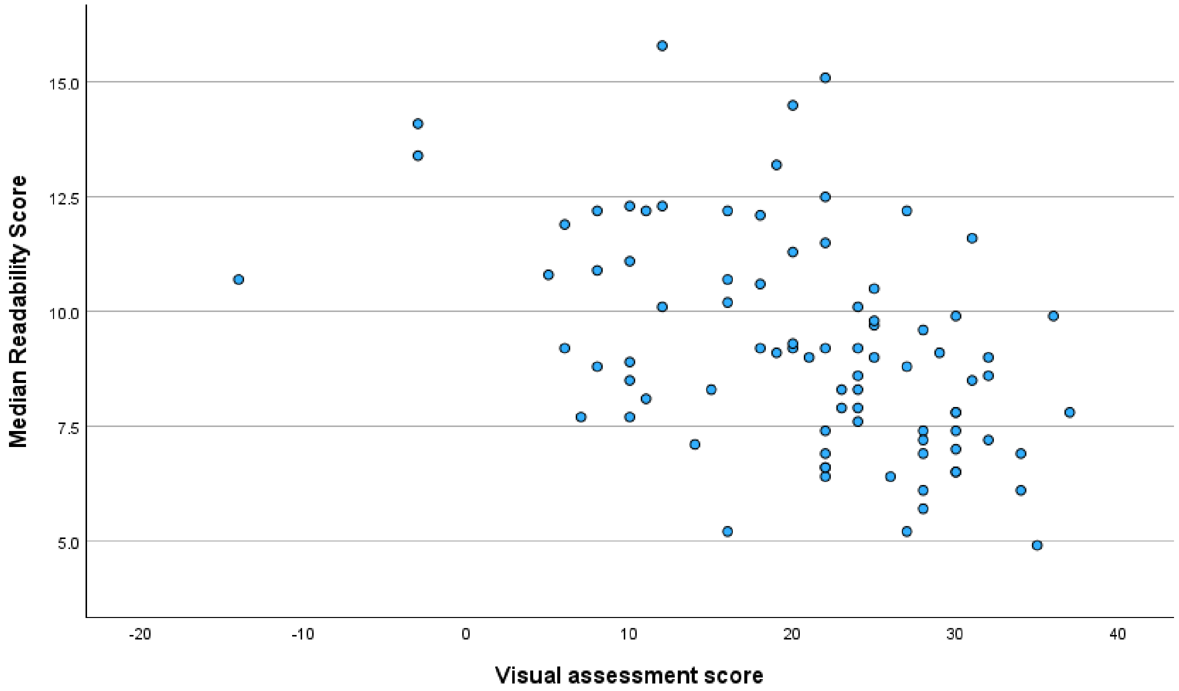

Spearman's rho= -0.485; p<0.01

For peer review only

Supplementary information. Table S1. Visual assessment of each website based on the Centers for Medicare and Medicaid Services Toolkit for Making Written Material Clear and Effective[22].

	Website																					
**Scoring Criteria**	**1**	**2**	**3**	**4**	**5**	**6**	**7**	**8**	**9**	**10**	**11**	**12**	**13**	**14**	**15**	**16**	**17**	**18**	**19**	**20**	**21**	**22**
Overall design and page layout	1	-1	1	1	-1	1	1	1	1	-1	1	-1	-1	1	1	1	1	1	-1	-1	1	1
The size, shape and general look of the material was designed with its purpose and users in mind	1	-1	1	1	1	1	-1	1	1	-1	1	-1	-1	1	1	1	1	1	1	-1	1	1
The material looks appealing at first glance	1	-1	1	1	-1	1	1	1	1	1	1	-1	-1	1	1	1	1	1	-1	1	1	1
A clear and obvious path has been created for the eye to follow	1	1	1	1	1	1	-1	1	1	-1	1	1	1	1	1	1	1	1	1	1	1	1
The material that has a clear and consistent style and structure	1	1	1	1	1	1	1	1	1	1	1	1	1	1	1	1	1	1	1	1	1	1
Fonts, size of print and contrast	1	1	1	1	1	1	1	1	1	1	1	1	-1	1	1	1	1	1	1	1	1	1
For the regular text, a font that is designed for ease of reading is used	1	1	1	1	1	1	1	1	1	1	1	1	-1	1	1	1	1	1	1	1	1	1
For headings, an easy-to-read font is used that contrasts with the main text	1	1	1	1	1	1	1	1	1	1	1	1	1	1	1	1	1	1	1	1	1	1
In general, no more than 2 or 3 different typefaces are used	1	1	1	-1	1	1	1	1	1	-1	1	1	1	1	1	1	-1	1	1	1	1	1
The font size is large enough for the intended audience	1	1	1	1	1	1	-1	1	1	-1	1	1	-1	1	1	1	1	1	1	1	1	1
Upper and lower case are used, not all capitals	1	1	1	1	1	1	1	1	1	1	1	-1	1	1	1	1	1	1	1	1	1	1
To emphasize words and phrases italics or bold text are used	1	1	1	-1	1	1	1	1	1	1	1	1	1	1	1	1	1	1	1	1	1	1
For ease of reading, dark-coloured text is used on a very light background	1	1	1	1	1	1	1	1	1	1	1	1	1	1	1	1	1	1	1	1	1	1
Text is not aligned sideways, on patterned or shaded background or on top of photos or other images	1	1	1	1	1	1	1	1	1	1	1	1	1	1	1	1	1	1	1	1	1	1
For ease of reading, extra line spacing has been added	1	1	1	1	1	1	1	1	1	-1	1	1	-1	1	1	1	-1	1	1	1	1	1
For ease of reading, left justification is used throughout	1	1	1	1	1	1	1	1	1	1	1	1	-1	1	1	1	-1	1	1	1	1	1
Lines of text are an appropriate length—neither too long nor too short	-1	-1	1	-1	1	1	1	1	1	-1	-1	-1	-1	1	1	1	1	-1	1	1	1	1
Hyphenation has been avoided at the end of lines	1	1	1	1	1	1	1	1	1	1	1	1	-1	1	1	1	1	1	1	1	1	1
Headings, bulleted lists, and blocks of text	1	1	1	1	1	1	1	1	1	1	-1	1	1	1	1	1	1	1	1	1	1	1
There is a clear hierarchy of prominent headings and sub-headings	1	1	1	1	1	1	1	1	1	1	1	1	1	1	1	1	-1	1	1	-1	1	1
Contrast is used to make the main points stand out	1	1	1	1	1	1	1	1	1	1	1	1	-1	1	1	1	1	1	1	1	1	1
Bulleted lists are well formatted	0	1	1	1	-1	1	1	1	0	1	0	1	0	1	1	1	1	1	1	1	1	1
Effective ways are used to emphasize important blocks of text	0	-1	1	-1	-1	1	1	1	1	1	1	1	1	1	1	1	-1	1	1	1	1	1
Use of colour	0	-1	1	1	-1	-1	-1	-1	1	1	-1	-1	-1	1	-1	-1	-1	1	-1	1	1	1
Colours used are appealing to the intended readers	0	-1	1	-1	-1	-1	-1	-1	1	-1	1	-1	-1	1	-1	-1	-1	-1	-1	-1	1	1
Colour is used sparingly and in a consistent and deliberate way	1	1	1	-1	-1	1	-1	-1	1	1	1	-1	-1	1	1	1	-1	1	1	1	1	1

For peer review only

Scoring Criteria	1	2	3	4	5	6	7	8	9	10	11	12	13	14	15	16	17	18	19	20	21	22
The colour scheme works from a design standpoint and when printed	1	1	1	-1	-1	1	-1	1	1	-1	1	1	-1	1	1	1	-1	1	1	-1	1	1
The colour scheme works for with diminished or limited colour perception	0	1	1	-1	-1	1	-1	1	1	-1	1	1	-1	1	1	1	-1	1	1	-1	1	1
Photographs and illustrations	-1	-1	-1	-1	1	-1	1	-1	1	1	-1	-1	1	-1	-1	-1	1	-1	-1	-1	-1	1
Photos and illustrations are used that relate directly to the information to reinforce key messages	0	0	0	-1	-1	0	-1	0	-1	-1	0	0	1	0	-1	0	1	0	0	-1	0	1
Images used are clear, uncluttered, and consistent in style	0	0	0	-1	-1	0	-1	0	-1	-1	-1	0	1	0	0	0	1	0	0	-1	0	1
Photos and illustrations used are culturally appropriate for the intended readers	0	0	0	-1	-1	0	1	0	0	-1	-1	0	1	0	0	0	1	0	0	-1	0	1
When images include people, they are appropriate to the situation and intended audience	0	0	0	0	0	0	0	0	0	0	0	0	-1	0	0	0	1	0	0	-1	0	1
Tables, charts, and diagrams	-1	-1	-1	1	-1	-1	1	-1	-1	-1	-1	-1	-1	-1	-1	-1	-1	-1	-1	-1	-1	1
Likely literacy levels of the reader have been considered in the use of tables, charts, and diagrams	-1	0	0	-1	0	0	0	0	1	0	-1	0	0	0	0	0	0	0	0	0	0	1
Titles, headings, and other labelling is specific and clear	1	1	0	-1	0	0	-1	1	1	1	1	1	1	1	1	1	-1	1	1	1	1	1
A clean, uncluttered layout is used with strong visual cures to guide the reader through the information	1	-1	1	1	-1	1	-1	1	-1	-1	1	-1	-1	1	1	1	-1	-1	1	-1	1	1
Numbers or calculations are carefully explained	0	0	0	0	0	0	0	0	0	0	0	0	0	0	0	0	0	0	0	0	0	0
General information about website	1	-1	1	1	-1	1	1	1	1	1	1	1	1	1	-1	1	1	1	1	1	-1	1
Last updated date given	1	1	1	-1	1	1	1	1	1	1	1	1	1	1	1	1	1	1	1	1	-1	1
Frequency of updates given	-1	-1	-1	-1	-1	1	-1	-1	-1	-1	-1	-1	-1	-1	-1	-1	-1	-1	-1	-1	-1	-1
Relevant references given	1	1	-1	1	1	-1	-1	-1	-1	-1	1	-1	-1	-1	-1	-1	-1	-1	-1	-1	-1	-1
**Overall assessment score**	22	12	27	8	6	25	11	22	26	5	20	10	-3	28	21	24	10	22	20	10	24	37

(+1 point if the statement was achieved, 0 if the statement was not applicable and -1 point if the statement was not achieved.)

For peer review only

	Website																					
**Scoring Criteria**	**23**	**24**	**25**	**26**	**27**	**28**	**29**	**30**	**31**	**32**	**33**	**34**	**35**	**36**	**37**	**38**	**39**	**40**	**41**	**42**	**43**	**44**
Overall design and page layout	1	1	1	1	1	-1	1	-1	1	-1	-1	1	1	-1	1	1	1	1	1	1	1	1
The size, shape and general look of the material was designed with its purpose and users in mind	1	1	1	1	1	1	1	-1	1	1	-1	1	1	-1	1	1	-1	1	1	1	1	1
The material looks appealing at first glance	1	1	1	1	1	-1	1	-1	1	1	-1	1	1	-1	1	1	1	1	-1	-1	-1	1
A clear and obvious path has been created for the eye to follow	1	1	-1	1	-1	-1	1	-1	1	1	1	1	1	1	1	1	1	1	-1	-1	-1	1
The material that has a clear and consistent style and structure	1	1	1	1	1	1	1	1	1	1	1	1	1	1	1	1	-1	1	1	1	1	1
Fonts, size of print and contrast	1	1	1	1	1	1	1	1	1	1	1	1	1	1	1	1	1	1	1	1	1	1
For the regular text, a font that is designed for ease of reading is used	1	1	1	1	1	1	1	1	1	1	1	1	1	1	1	1	1	1	1	1	1	1
For headings, an easy-to-read font is used that contrasts with the main text	1	1	1	1	1	1	1	1	1	1	1	1	1	1	1	1	1	1	1	1	1	1
In general, no more than 2 or 3 different typefaces are used	1	1	1	1	1	1	1	1	1	1	1	1	1	1	1	1	1	1	1	1	1	1
The font size is large enough for the intended audience	1	1	1	1	1	1	1	1	1	1	1	1	1	1	1	1	1	1	1	1	1	1
Upper and lower case are used, not all capitals	1	1	1	1	1	1	1	1	1	1	1	1	1	1	1	1	1	1	1	1	1	1
To emphasize words and phrases italics or bold text are used	1	1	1	1	1	1	1	1	1	1	1	1	1	1	1	1	1	1	1	1	1	1
For ease of reading, dark-coloured text is used on a very light background	1	1	1	1	1	1	1	1	1	1	-1	1	1	1	1	1	1	1	1	1	1	1
Text is not aligned sideways, on patterned or shaded background or on top of photos or other images	1	1	1	1	1	1	1	1	1	1	-1	1	1	1	1	1	1	1	1	1	1	1
For ease of reading, extra line spacing has been added	1	1	1	1	-1	1	1	1	1	1	-1	1	1	1	1	1	1	1	1	1	1	1
For ease of reading, left justification is used throughout	1	1	1	1	1	1	1	1	1	1	1	1	1	1	1	1	1	1	1	1	1	1
Lines of text are an appropriate length—neither too long nor too short	1	1	1	1	-1	1	1	1	-1	-1	-1	1	-1	1	1	1	-1	1	1	-1	-1	-1
Hyphenation has been avoided at the end of lines	1	1	1	1	1	1	1	1	1	1	1	1	1	1	1	1	1	1	1	1	1	1
Headings, bulleted lists, and blocks of text	1	1	1	1	1	1	1	1	1	1	1	1	1	1	1	1	1	1	1	1	1	1
There is a clear hierarchy of prominent headings and sub-headings	1	1	1	1	1	1	1	1	1	1	1	1	1	1	1	1	1	1	1	1	1	1
Contrast is used to make the main points stand out	1	1	1	1	1	1	-1	1	1	1	1	1	1	1	1	1	1	1	1	1	1	1
Bulleted lists are well formatted	1	1	1	1	0	1	1	1	1	1	1	1	1	1	1	1	1	1	1	1	1	1
Effective ways are used to emphasize important blocks of text	1	1	1	1	1	1	1	1	1	1	1	1	1	1	1	1	1	1	1	1	1	1
Use of colour	1	1	1	1	1	1	1	1	1	1	-1	1	-1	1	1	1	1	1	1	1	1	1
Colours used are appealing to the intended readers	1	1	1	1	-1	1	1	1	1	1	-1	1	1	1	1	1	1	1	1	1	1	1
Colour is used sparingly and in a consistent and deliberate way	1	1	1	1	-1	1	1	1	1	1	-1	1	1	1	1	1	1	1	1	1	1	-1
The colour scheme works from a design standpoint and when printed	-1	1	-1	1	-1	1	1	-1	1	1	-1	1	-1	1	1	1	1	1	1	1	1	-1

Scoring Criteria	23	24	25	26	27	28	29	30	31	32	33	34	35	36	37	38	39	40	41	42	43	44
The colour scheme works for with diminished or limited colour perception	1	1	-1	1	-1	1	1	-1	1	1	-1	1	-1	1	1	1	1	1	1	1	1	-1
Photographs and illustrations	1	-1	-1	-1	1	-1	1	-1	-1	1	-1	-1	1	-1	-1	-1	1	-1	-1	-1	1	1
Photos and illustrations are used that relate directly to the information to reinforce key messages	0	0	0	0	1	0	1	0	0	1	0	0	1	-1	0	0	-1	0	-1	-1	-1	-1
Images used are clear, uncluttered, and consistent in style	0	0	-1	0	1	0	1	0	0	1	0	0	1	-1	0	0	1	0	1	0	-1	-1
Photos and illustrations used are culturally appropriate for the intended readers	0	0	0	0	-1	0	1	0	0	1	0	0	1	-1	0	0	-1	0	-1	0	1	1
When images include people, they are appropriate to the situation and intended audience	0	0	0	0	0	0	0	0	0	0	0	0	1	-1	0	0	-1	0	1	0	1	0
Tables, charts, and diagrams	-1	-1	-1	-1	1	-1	-1	-1	-1	1	-1	-1	-1	-1	-1	-1	1	-1	-1	-1	-1	1
Likely literacy levels of the reader have been considered in the use of tables, charts, and diagrams	0	0	0	0	-1	0	1	0	0	1	-1	0	1	0	0	0	-1	0	0	0	0	-1
Titles, headings, and other labelling is specific and clear	1	1	1	1	1	1	1	1	1	1	1	1	1	1	1	0	1	1	1	1	1	1
A clean, uncluttered layout is used with strong visual cures to guide the reader through the information	1	1	-1	1	-1	-1	1	-1	1	-1	-1	1	1	-1	1	1	1	1	-1	1	-1	-1
Numbers or calculations are carefully explained	0	0	0	0	0	0	0	0	0	0	0	0	0	0	0	0	0	0	0	0	0	0
General information about website	1	1	-1	-1	-1	1	1	1	-1	1	-1	-1	1	1	1	-1	-1	1	-1	-1	1	-1
Last updated date given	1	1	-1	-1	-1	1	1	1	-1	1	-1	-1	1	1	1	-1	-1	1	-1	-1	1	1
Frequency of updates given	0	1	-1	-1	-1	-1	-1	-1	-1	-1	-1	-1	-1	-1	1	-1	-1	1	-1	-1	-1	-1
Relevant references given	-1	-1	-1	-1	-1	-1	-1	-1	-1	1	-1	-1	-1	-1	-1	-1	-1	-1	-1	1	-1	-1
**Overall assessment score**	29	30	15	24	11	20	32	14	22	32	-3	24	27	16	30	23	19	30	18	19	22	18

(+1 point if the statement was achieved, 0 if the statement was not applicable and -1 point if the statement was not achieved.)

For peer review only

	Website																					
**Scoring Criteria**	**45**	**46**	**47**	**48**	**49**	**50**	**51**	**52**	**53**	**54**	**55**	**56**	**57**	**58**	**59**	**60**	**61**	**62**	**63**	**64**	**65**	**66**
Overall design and page layout	1	1	1	1	1	1	1	1	1	1	1	-1	1	-1	-1	1	-1	-1	1	1	1	1
The size, shape and general look of the material was designed with its purpose and users in mind	1	1	1	1	1	1	1	1	1	1	1	1	1	-1	-1	1	1	-1	1	1	1	1
The material looks appealing at first glance	1	1	-1	-1	1	1	1	1	1	1	1	1	1	-1	-1	-1	-1	-1	1	1	1	-1
A clear and obvious path has been created for the eye to follow	1	1	1	1	1	1	1	1	1	1	1	-1	1	-1	-1	-1	-1	-1	1	1	1	-1
The material that has a clear and consistent style and structure	1	1	1	1	1	1	1	1	1	1	1	1	1	1	1	1	1	1	1	1	1	1
Fonts, size of print and contrast	1	1	1	1	1	1	1	1	1	1	1	1	1	1	1	1	1	1	1	1	-1	1
For the regular text, a font that is designed for ease of reading is used	1	1	1	1	1	1	1	1	1	1	1	1	1	1	1	1	1	1	1	1	1	1
For headings, an easy-to-read font is used that contrasts with the main text	1	1	1	1	1	1	1	1	1	1	1	1	1	1	1	1	1	1	1	1	1	1
In general, no more than 2 or 3 different typefaces are used	1	1	1	1	1	1	1	1	1	1	1	1	1	1	1	1	1	1	1	1	1	1
The font size is large enough for the intended audience	1	1	1	1	1	1	1	1	1	1	1	1	1	1	1	1	1	1	1	1	1	1
Upper and lower case are used, not all capitals	1	1	1	1	1	1	1	1	1	1	1	1	1	1	1	1	1	1	1	1	1	1
To emphasize words and phrases italics or bold text are used	1	1	1	1	1	1	1	1	1	1	1	1	1	1	1	1	1	1	1	1	-1	1
For ease of reading, dark-coloured text is used on a very light background	1	1	1	1	1	1	1	1	1	1	1	-1	1	1	1	1	1	1	1	1	-1	1
Text is not aligned sideways, on patterned or shaded background or on top of photos or other images	1	1	1	1	1	1	1	1	1	1	1	1	1	1	1	1	1	1	1	1	1	1
For ease of reading, extra line spacing has been added	1	1	1	1	1	1	1	1	1	1	1	1	1	1	1	1	1	1	1	1	1	1
For ease of reading, left justification is used throughout	1	1	1	1	1	1	1	1	1	1	1	1	1	1	1	1	1	1	1	1	1	1
Lines of text are an appropriate length—neither too long nor too short	-1	1	1	1	1	1	1	1	1	1	-1	-1	1	-1	-1	1	-1	-1	1	1	-1	1
Hyphenation has been avoided at the end of lines	1	1	1	1	1	1	1	1	1	1	1	1	1	1	1	1	1	1	1	1	1	1
Headings, bulleted lists, and blocks of text	1	1	1	1	1	1	1	1	1	1	1	1	1	1	1	1	1	1	1	1	1	1
There is a clear hierarchy of prominent headings and sub-headings	1	1	1	1	1	1	1	1	1	1	1	1	1	1	1	1	1	1	1	1	1	1
Contrast is used to make the main points stand out	1	1	1	1	1	1	1	1	1	1	1	1	1	1	1	1	1	1	1	1	-1	1
Bulleted lists are well formatted	1	1	1	1	1	1	1	1	1	1	1	1	1	1	1	1	1	-1	1	1	1	1
Effective ways are used to emphasize important blocks of text	1	1	1	1	1	1	1	1	1	1	1	1	1	1	1	1	1	1	1	1	-1	1
Use of colour	1	1	-1	-1	-1	1	1	1	1	1	1	1	1	1	1	1	1	1	1	1	1	1
Colours used are appealing to the intended readers	1	1	-1	-1	1	1	1	1	1	1	1	-1	1	1	1	1	1	1	1	1	1	1
Colour is used sparingly and in a consistent and deliberate way	1	1	1	1	1	1	1	1	1	1	1	-1	1	1	1	1	1	1	1	1	1	1
The colour scheme works from a design standpoint and when printed	1	1	1	1	1	1	1	1	1	1	1	-1	1	1	1	1	1	1	1	1	-1	1
The colour scheme works for with diminished or limited colour perception	1	1	1	1	1	1	1	1	1	1	1	-1	1	1	1	1	1	1	1	1	-1	1

For peer review only

Scoring Criteria	45	46	47	48	49	50	51	52	53	54	55	56	57	58	59	60	61	62	63	64	65	66
Photographs and illustrations	1	1	-1	-1	-1	-1	-1	1	-1	-1	-1	1	1	1	-1	1	1	-1	1	-1	-1	1
Photos and illustrations are used that relate directly to the information to reinforce key messages	-1	-1	0	0	0	0	0	-1	0	0	0	0	-1	-1	-1	-1	-1	0	1	0	0	1
Images used are clear, uncluttered, and consistent in style	1	1	0	0	0	0	0	1	0	0	0	0	1	0	-1	0	0	0	1	0	0	1
Photos and illustrations used are culturally appropriate for the intended readers	1	0	0	0	0	0	0	1	0	0	0	1	1	0	0	0	1	0	1	0	0	1
When images include people, they are appropriate to the situation and intended audience	-1	-1	0	0	0	0	0	1	0	0	0	1	1	-1	0	-1	0	0	1	0	0	-1
Tables, charts, and diagrams	-1	-1	-1	-1	-1	-1	-1	-1	-1	-1	-1	-1	-1	-1	-1	-1	-1	-1	-1	-1	-1	-1
Likely literacy levels of the reader have been considered in the use of tables, charts, and diagrams	0	0	0	0	0	0	0	0	0	0	0	0	0	0	0	0	0	0	1	0	0	0
Titles, headings, and other labelling is specific and clear	1	1	1	1	1	1	1	1	1	1	1	1	1	1	1	1	1	1	1	1	1	1
A clean, uncluttered layout is used with strong visual cures to guide the reader through the information	1	1	1	1	1	1	1	1	1	-1	1	-1	1	-1	-1	1	-1	-1	1	1	1	1
Numbers or calculations are carefully explained	0	0	0	0	0	0	0	0	0	0	0	0	0	0	0	0	0	0	0	0	0	0
General information about website	-1	-1	1	1	1	1	1	1	-1	1	1	-1	-1	-1	1	-1	1	-1	1	1	-1	1
Last updated date given	-1	-1	1	1	1	1	1	1	-1	1	1	-1	-1	-1	-1	-1	-1	-1	1	1	-1	1
Frequency of updates given	-1	-1	-1	-1	1	1	1	1	-1	1	-1	-1	-1	-1	-1	-1	-1	-1	-1	1	-1	-1
Relevant references given	-1	-1	-1	-1	-1	-1	-1	-1	-1	-1	1	-1	-1	-1	-1	-1	-1	-1	-1	-1	-1	-1
**Overall assessment score**	24	25	22	22	28	30	30	34	24	28	28	10	28	12	12	20	18	10	35	30	8	28

(+1 point if the statement was achieved, 0 if the statement was not applicable and -1 point if the statement was not achieved.)

For peer review only

	Website																		
**Scoring Criteria**	**67**	**68**	**69**	**70**	**71**	**72**	**73**	**74**	**75**	**76**	**77**	**78**	**79**	**80**	**81**	**82**	**83**	**84**	**85**
Overall design and page layout	-1	1	1	1	-1	1	1	1	1	1	-1	1	-1	1	1	1	-1	-1	1
The size, shape and general look of the material was designed with its purpose and users in mind	1	1	1	1	1	1	1	1	1	1	-1	1	1	1	1	1	1	-1	1
The material looks appealing at first glance	-1	1	1	-1	-1	1	1	-1	-1	-1	-1	1	-1	1	1	1	1	-1	1
A clear and obvious path has been created for the eye to follow	-1	1	1	1	1	1	1	1	1	1	-1	1	1	1	-1	1	1	-1	1
The material that has a clear and consistent style and structure	1	1	1	1	1	1	1	1	1	1	-1	1	1	1	-1	1	1	-1	1
Fonts, size of print and contrast	1	1	1	1	1	1	-1	-1	1	1	-1	1	1	1	1	1	1	1	1
For the regular text, a font that is designed for ease of reading is used	1	1	1	1	1	1	-1	1	1	1	-1	1	1	1	1	1	1	1	1
For headings, an easy-to-read font is used that contrasts with the main text	1	1	1	1	1	1	1	-1	1	1	1	1	1	1	1	1	1	1	1
In general, no more than 2 or 3 different typefaces are used	1	1	1	1	1	1	1	1	1	1	1	1	1	1	1	1	1	1	1
The font size is large enough for the intended audience	1	1	1	1	1	1	1	-1	1	1	-1	1	1	1	1	1	1	1	1
Upper and lower case are used, not all capitals	1	1	1	1	1	1	1	1	1	1	1	1	1	1	1	1	1	-1	1
To emphasize words and phrases italics or bold text are used	-1	1	1	1	-1	1	1	1	1	1	1	1	1	1	1	1	1	1	1
For ease of reading, dark-coloured text is used on a very light background	-1	1	1	1	1	1	1	1	1	1	-1	1	1	1	1	1	1	1	1
Text is not aligned sideways, on patterned or shaded background or on top of photos or other images	1	1	1	1	1	1	1	1	1	1	1	1	1	1	1	1	1	1	1
For ease of reading, extra line spacing has been added	1	1	1	1	1	1	1	1	1	1	-1	1	1	1	1	1	1	1	1
For ease of reading, left justification is used throughout	1	1	1	1	1	1	1	1	1	1	1	1	1	1	1	1	1	-1	1
Lines of text are an appropriate length—neither too long nor too short	-1	1	1	-1	-1	1	-1	1	1	1	-1	1	-1	1	-1	-1	1	1	1
Hyphenation has been avoided at the end of lines	1	1	1	1	1	1	1	1	1	1	1	1	1	1	1	1	1	1	1
Headings, bulleted lists, and blocks of text	1	1	1	1	-1	1	1	1	1	1	1	1	1	1	1	1	1	1	1
There is a clear hierarchy of prominent headings and sub-headings	1	1	1	1	-1	1	1	1	1	1	-1	1	1	1	1	1	1	-1	1
Contrast is used to make the main points stand out	1	1	1	1	-1	1	1	1	1	1	1	1	1	1	1	1	1	1	1
Bulleted lists are well formatted	1	1	1	1	-1	1	1	1	1	1	1	1	1	1	1	1	1	1	1
Effective ways are used to emphasize important blocks of text	1	1	1	1	-1	1	1	1	1	1	-1	1	1	1	1	1	1	1	1
Use of colour	1	1	1	1	-1	1	1	1	1	1	-1	1	1	1	1	1	1	1	1
Colours used are appealing to the intended readers	-1	1	1	1	-1	1	1	1	1	-1	-1	1	1	1	1	1	1	1	1
Colour is used sparingly and in a consistent and deliberate way	1	1	1	1	1	1	1	1	1	-1	-1	1	1	1	1	1	1	1	1
The colour scheme works from a design standpoint and when printed	-1	1	1	1	1	1	1	1	1	1	-1	1	1	1	1	1	1	1	1
The colour scheme works for with diminished or limited colour perception	-1	1	1	1	1	1	-1	1	1	-1	-1	1	1	1	1	1	1	1	1

**Scoring Criteria**	**67**	**68**	**69**	**70**	**71**	**72**	**73**	**74**	**75**	**76**	**77**	**78**	**79**	**80**	**81**	**82**	**83**	**84**	**85**
Photographs and illustrations	1	-1	1	1	-1	1	-1	-1	-1	1	-1	-1	1	-1	-1	-1	-1	1	1
Photos and illustrations are used that relate directly to the information to reinforce key messages	-1	0	1	1	0	-1	0	0	0	1	0	0	1	0	0	0	0	-1	1
Images used are clear, uncluttered, and consistent in style	1	0	1	-1	0	1	0	0	0	1	0	0	1	0	0	0	0	-1	1
Photos and illustrations used are culturally appropriate for the intended readers	1	0	1	1	0	0	0	0	0	1	0	0	1	0	0	0	0	1	1
When images include people, they are appropriate to the situation and intended audience	0	0	0	1	0	0	0	0	0	1	0	0	1	0	0	0	0	1	1
Tables, charts, and diagrams	-1	-1	-1	1	-1	-1	-1	-1	-1	1	-1	-1	1	-1	-1	-1	-1	-1	-1
Likely literacy levels of the reader have been considered in the use of tables, charts, and diagrams	0	0	1	-1	0	-1	0	0	0	1	0	1	1	0	0	0	0	0	0
Titles, headings, and other labelling is specific and clear	1	1	1	1	1	1	1	1	1	1	1	1	1	1	1	1	0	1	1
A clean, uncluttered layout is used with strong visual cures to guide the reader through the information	-1	1	1	1	1	1	1	1	1	1	-1	1	-1	1	-1	1	1	-1	1
Numbers or calculations are carefully explained	0	0	0	0	0	0	0	0	0	0	0	0	0	0	0	0	0	0	0
General information about website	-1	1	1	1	1	-1	-1	-1	-1	-1	-1	1	-1	1	-1	-1	-1	-1	1
Last updated date given	-1	1	1	1	1	-1	-1	-1	-1	-1	-1	1	-1	1	-1	-1	-1	-1	1
Frequency of updates given	-1	1	1	-1	-1	-1	-1	-1	-1	-1	-1	1	-1	1	-1	-1	-1	-1	-1
Relevant references given	-1	1	-1	1	-1	1	-1	-1	-1	-1	-1	-1	-1	-1	-1	-1	1	-1	-1
**Overall assessment score**	7	32	36	31	6	27	16	16	22	25	-14	31	25	30	16	22	23	8	34

(+1 point if the statement was achieved, 0 if the statement was not applicable and -1 point if the statement was not achieved.)

For peer review only

Supplementary information. Table S2. Narrative overview assessment of the 85 identified websites.

Name of Website	Website address	Website assessment
1. Medscape	https://emedicine.medscape.com/article/428723-overview?form=fpf	The Medscape website is clearly laid out, with bold headings, sub-headings and regular use of paragraphs. This makes it aesthetically appealing, with clear links to move between different sections of the article. However, there is heavy use of medical terminology and information that is not relevant or useful to the public as it details complex anatomical and physiological structures. There is no use of diagrams, only an image of an x-ray. The website does include information on causes, symptoms and treatments of BCT. This website appears more focused on use by clinicians than by the general public.
2. UpToDate	https://www.uptodate.com/contents/initial-evaluation-and-management-of-blunt-thoracic-trauma-in-adults	The UpToDate website page contains very informative content regarding BCT. There are links to further information on specific injuries, however this website focuses more on the anatomical nature of these injuries rather than symptoms and how to diagnose them. In general, this website seems to focus on information relevant to a clinician rather than a member of the public, with detailed descriptions of medical techniques. The layout is poor, as the text area only takes up around half of the website page. There are links to images, although they are all scientific and of little use when educating the public.
3. Health Direct	https://www.healthdirect.gov.au/chest-injuries	The Health Direct website provides good information on chest injury, for both BCT and penetrating injuries. Headings are effectively used to segregate the information for blunt and penetrating wounds. The page is well laid out with bullet points highlighting key points. Among a list of possible chest injuries, there is extra information given on the most common ones. This shows that the primary aim of this website is to educate members of the public on potential BCT injuries, and use of non-technical language allows the page to do this effectively. An area in which the website

For peer review only

Name of Website	Website address	Website assessment
		lacks is the lack of images and diagrams, which can be effective in communicating some potential visible symptoms of chest injuries.
4. DynaMed	https://www.dynamed.com/management/blunt-chest-trauma-emergency-management#GUID-E3A89009-4D66-4D2B-9ABE-DF2E70EE0CFA	The general appearance of the DynaMed website is appealing, but the full content is not available as it requires a subscription for more detailed information. There are some colourful pictures but the explanation of these and the text in general is very scientific and appears to be more appropriate for clinicians than patients. A medical and or radiological understanding would be needed to interpret the x-ray photos.
5. Wikipedia	https://en.wikipedia.org/wiki/Chest_injury	While Wikipedia does not have a designated website page solely for BCT, it has a general blunt trauma page in which a section is designated to the thoracic region. While there is not a large volume of information on BCT, it contains causes and symptoms for certain chest injuries. However, the majority of the text contains medical terminology. The layout is confusing, with no subheadings and poor spacing. Some positives of the page included the use of tables that clearly outline some types of BCT accidents and what injuries they could cause, which was an effective tool for education on BCT. There were also a variety of links taking the reader to further information if they needed it.
6. North Bristol NHS Trust	https://www.nbt.nhs.uk/our-services/a-z-services/emergency-zone/ed-miu-patient-informationtt-information/chest-injury-advice	This is badged with an NHS and hospital logo which immediately gives credibility to the webpage. It is coloured in the usual blue and white NHS colour scheme. The webpage is titled "Chest injury advice- what happens when you're admitted to hospital with a chest injury', which allows the person accessing the site to determine the relevancy of the information. The language is very straightforward and there is clear delineation of sections. Bold text is used to highlight key headings in a large font. Dark font is used on a white background to aid contrast. The information is however very brief and there are no figures/diagrams to aid understanding. Useful contact information is included.

For peer review only

Name of Website	Website address	Website assessment
7. Drugs.com	https://www.drugs.com/cg/blunt-chest-trauma.html	The Drugs.com webpage has several adverts placed throughout the page. These take up a large part of the screen, including throughout the text sections. The information is largely to do with treatment and care for BCT injuries, with little information about causes and symptoms. Headings and bullet-points make the text appealing to read, but the lack of images hinders the visual appearance overall. Some abbreviations are used without reference to what they mean, which could confuse readers with no background knowledge. There are links to further information at the bottom of the page.
8. Gloucestershire Hospitals NHS Trust	https://www.gloshospitals.nhs.uk/media/documents/ Chest_trauma_GHPI0653_01_22_4EXEhZ5.pdf	This web address provides a link to a patient information leaflet about chest trauma. It is badged with the Gloucester Hospital logo and the NHS which immediately improves credibility. It is very clean and clear and has major headings that introduce chest trauma, what it is and how it can be managed. It also gives advice on when the patient should seek more help and contact information. The sentences are short and clear. There are no figures, tables and graphics which could potentially help aid understanding.
9. VeryWellHealth	https://www.verywellhealth.com/chest-trauma-3913241	This website has several adverts at the top and down the side of the page. The text itself focuses on all chest injuries rather than just BCT, with very little mention of BCT. The layout of text is good, with subheadings and images used well, as well as links to further information present. The language used is appropriate for most readers.
10. MSD Manual	https://www.msdmanuals.com/en-gb/home/injuries-and-poisoning/chest-injuries/blunt-injury-to-the-heart	This website contains information on chest injuries, with only some relevant to BCT. The introduction contains appropriate sections on symptoms, diagnosis, treatment for individuals with no clinical knowledge. However, the following sections use more advanced terminology that is not suitable for education of the public. There is limited use of images, and the text only makes use of around half of the page.

For peer review only

Name of Website	Website address	Website assessment
11. The American Association for the Surgery of Trauma	https://www.aast.org/resources-detail/blunt-cardiac-injury	The website visually looks very professional on first inspection. There are some adverts which distract from the information provided. The language is very clinical as would be expected of a professional body. The information also relies of medical knowledge to understand it. There are no figures/photographs or tables which could potentially aid understanding.
12. LifeInTheFastlane	https://litfl.com/thoracic-trauma/	This website is visually unappealing, with little use of colour, adverts down one side of the page, and too much empty white space. The information contained in the page appears aimed at medical professionals, as medical terminology is used throughout. The information also seems to be solely based on types of injury, and how they can be identified and treated by a medical professional. No images are used to accompany the text. There are no links to further information.
13. Radiology key	https://radiologykey.com/blunt-chest-trauma/	This website is organised in a very simple manner, with no adverts or pop-ups. The information contained however is too advanced, due to the use of medical terminology and images of x-rays and CT scans that would only be helpful to a medical professional. The layout of the text is also poor, with long sentences and paragraphs. There is no easy way to navigate through the website, due to the length of the text and lack of links to specific sections.
14. Broken or bruised ribs (NHS): multiple sites	https://www.eastenfieldmedicalpractice.nhs.uk/conditions/broken-or-bruised-ribs/	Many identified links in the NHS (in primary and secondary care) used the same standard text, under their name, with a clear badge that identified that they were an NHS site. This text was simple and clear and written in a language appropriate for a lay person. The focus was only on bruised or broken ribs rather than blunt chest trauma specifically. There was a brief summary to help patients identify if they had bruised/broken ribs, followed by self help information and how to get further advice or make contact. There were no distracting adverts, but also no figures/diagrams to aid understanding.

For peer review only

Name of Website	Website address	Website assessment
15. Oxford University Hospitals NHS Trust	https://www.ouh.nhs.uk/patient-guide/leaflets/files/10110Pchestinjury.pdf	This web address provides a link to a patient information leaflet about chest trauma. It is badged with the Oxford Hospital logo and the NHS which immediately improves credibility. It is very clean and clear and has major headings that introduce the relevant information. Bold headings are used to emphasise key information. There is limited use of colour and no figures/diagrams to illustrate the information. Contact details are included if the patient needs more information.
16. National Institute for Health and Care Excellence (NICE)	https://www.nice.org.uk/guidance/ng39/ifp/chapter/treating-chest-injuries	The NICE website contains information on all types of trauma injuries, making it only partially relevant to BCT. The page is visually appealing, with no adverts taking up space, and the text is large enough to read easily. A section is specified as 'information for the public', making it very clear to the reader which section is intended for their education. No images are used, but clear sub-headings and short paragraph lengths make the text visibly appealing and easy to navigate. Links to further information on care are clearly present.
17. Aneurin Bevan University Health Board	https://abuhb.nhs.wales/files/patient-information-leaflets1/accidents-and-minor-injuries/chest-injury-advice-sheet-pdf/	The web address links to a patient information leaflet about chest injury advice. The NHS and hospital logos increase credibility of the information. The language is fairly clear and straightforward but it looks quite cluttered with limited use of white space and as such is not very appealing. Red text and coloured text are sometimes used to used to highlight important areas but the colours are not appealing and may be difficult to see if printed. Some figures have been used to aid understanding.
18. My Health Alberta	https://myhealth.alberta.ca/Health/aftercareinformation/pages/conditions.aspx?hwid=uf7538	The MyHealth.Alberta website is focused on chest contusions, so again not solely on BCT. The information contained is quite brief, being mainly focused on care instructions for someone with a chest contusion. Further links to specific chest conditions are provided. There is no use of images or diagrams in addition to the text, however the page is clearly laid out with headings and bullet-points. The text could take up more of the page, with large areas of empty

For peer review only

Name of Website	Website address	Website assessment
		space. The language used is appropriate for someone with no medical background, due to almost no medical terminology used.
19. Salisbury NHS Foundation Trust	https://www.google.com/search?q=blunt+chest+trauma+salisbury&rlz=1C1CHBF_en-GBGB987GB987&oq=blunt+chest+trauma+salisbury&gs_lcrp=EgZjaHJvbWUyBggAEEUYOTIHCAEQIRigATIHCAIQIRigATIHCAMQIRigAdIBCDU1MTFqMWo5qAIAsAIA&sourceid=chrome&ie=UTF-8	The web address links to a patient information leaflet about blunt chest trauma. The language is very clear and straightforward but the information is brief and the leaflet is not particularly appealing with only black text on a white background. There are no NHS or hospital logos but the name of the NHS Trust provides a level of credibility. There are no figures to help illustrate but there basic management information is provided as well as contact information if required.
20. WebMD	https://www.webmd.com/heart/what-is-myocardial-contusion	There are lots of adverts throughout the text and to the right which distract from information provided. Many of the adverts are not relevant to the clinical information. The information itself is fairly easy to read and links are provided to linked information. There are however a large number of medical terms used which may limit understanding.
21. After Trauma	https://www.aftertrauma.org/diagnosis-and-treatment/managing-chest-wall-injuries	This website contains two pages dedicated to chest injuries. It focuses on the management of injuries, treatment and recovery, although some information was included on signs and symptoms. The page is well laid out, with good use of headings, sub-headings and bullet-points. There are no distracting adverts or unnecessary information, and there is good use of white space, making it easy to read. One clear diagram is used to show the anatomy of the chest, which complements the text. One negative is that there is medical terminology used throughout.
22. Royal Devon University Healthcare NHS Foundation Trust & Northern Devon Healthcare NHS Trust	https://www.royaldevon.nhs.uk/media/z51hk2mn/advice-after-a-chest-wall-injury.pdf	The web address links to a patient information leaflet about chest wall trauma. There are NHS and hospital logos which provides a level of credibility. The language is very clear and straightforward and provides background and self-help guidance as well as further contact information. It also includes clear figures that aid understanding. The language is clear.

For peer review only

Name of Website	Website address	Website assessment
23. South Tees Hospitals NHS Foundation Trust	https://www.southtees.nhs.uk/resources/chest-injury/	The website is badged with the hospital and NHS logos. It uses the blue and white NHS colouring so immediately appears credible. The information is all organised on one page and the language is straightforward. It provides a brief overview of chest injuires with symptoms, self-help guidance and contact information about seeking further help if needed. There is one generic image but nothing to help understand the information. It is clean and uncluttered with no advertisements.
24. Sirona care and health	https://www.sirona-cic.org.uk/wp-content/uploads/2022/02/0050_How-to-treat-a-Chest-Wall-web.pdf	This link takes you to a patient information leaflet titled 'how to treat a chest wall injury'. It is very professional looking and is badged with the NHS logo, thus improving credibility. There is limited use of colour on a plain white background. It provides an overview of symptoms, dos and don'ts, management and contacts in an emergency. There are no figures or tables and no adverts. The language is clear.
25. eMedicineHealth	https://www.emedicinehealth.com/wilderness_chest_injuries/article_em.htm	The emedicinehealth website contains information on chest injuries of all description, but specifically discusses BCT causes and injuries. The layout of the page is good, with white space and sub-headings used effectively to allow easy navigation of the text. Bullet-points are used to highlight key pieces of information, and pictures complement the text. The language used is appropriate, and there are links to access further information. However, there are some adverts present that may distract the reader.
26. Better Health Channel	https://www.betterhealth.vic.gov.au/health/conditionsandtreatments/rib-injuries	This is a very clean, uncluttered and professional looking website that focuses on rib injuries. Colour is used sparingly and with lots of white space. The information is broken down into clear headings. Some of the language used is a bit technical. There are no figures or tables which could aid understanding, but also no adverts to distract.
27. Rib Injury Clinic	https://www.ribinjuryclinic.com/conditions/complex-chest-wall-injuries/	On initial inspection this website looks quite appealing as it contains lots of images and photographs. It uses dark text on a light background but could benefit from more white space. Further

For peer review only

Name of Website	Website address	Website assessment
		inspection identifies that the conext is very complex as are the photographs and figures. It would be very difficult for a patient to understand the information. The language used is also highly technical. On the positive side there are no distracting adverts.
28. Hull University Teaching Hospitals NHS Trust	https://www.hey.nhs.uk/patient-leaflet/__trashed-35/	The link to this information first directs you to an 'advert' for accessibility testers, which is a little distracting and has to be closed before further information can be accessed. Once the information is accessed it is badged as the hospital and NHS which adds credibility. There is 'admin' information at the top of the page and the website menu to the right which makes the flow of information difficult. The information itself is a little complex but provides background, self-help guidance and where to go for more help. No figures or tables.
29. Mount Nittany Health	https://mountnittany.org/wellness-article/chest-wall-contusion	This website provides some basic information about 'chest contusion/brusing. It provides some background including a simple diagram of the chest. The language used is not too technical and it provides information about management and where to go for further help. No distracting adverts are included.
30. MedlinePlus	https://medlineplus.gov/chestinjuriesanddisorders.html	The website feels rather cluttered as there is admin information and a menu to the right. There are no adverts. The information provided is fairly simple and there are links to further information including how various chest conditions are diagnosed. Limited technical language is used in the main narrative, although many links use technical language.
31. European Society of Thoracic Surgeons	https://www.ests.org/about_ests/patient_information/diseases/chest_trauma.aspx#googtrans/en/en	This website has information spread over several pages. Information is not solely BCT related, however a large amount is relevant. The layout of the page is clear, with no adverts and good use of white space. Key pieces of information are highlighted using bullet-points. Some of the language used is too advanced for use by the public, and there is a lack of images giving a visual representation of the information in the text. There are links to further information.

For peer review only

Name of Website	Website address	Website assessment
32. Cleveland Clinic	https://my.clevelandclinic.org/health/diseases/23994-flail-chest	The website contains adverts at the top and to the right which distracts from the information provided. Generally the information looks fairly appealing and includes a simple figure. It focuses on 'flail chest'. It summarises key information but uses technical terms that make the information more difficult to understand.
33. mactheknife.org	www.mactheknife.org/Chest_Trauma/Mechanisms.html	This website provide some detail about the mechanisms of chest injory. There is a lot of text presented with limited line spacing on a patterned background. Visually this makes the information difficult to read especially for those with sight impairments. There are no figures or photos but also no distracting adverts. The content is very complex and lots of technical terms are used.
34. World Rugby Passport	https://passport.world.rugby/player-welfare-medical/first-aid-in-rugby/chapter-6-breathing-assessment-and-chest-injuries/potential-causes-of-rapid-breathing/chest-wall-or-lung-injuries/	This website presents a brief page of information about how to manage chest wall or lung injuries in rugby players. The website itself is very professional looking but the information is very limited and appears to be focused of how clinicians manage injuries. There are no figures or photos but equally no distracting adverts. The information presented is very clear and non-technical.
35. Total Vitality Medical Group	https://totalvitalitymedical.com/news/what-you-need-to-know-about-chest-contusions/	This website focuses on chest contusions/bruising and the page begins with a photograph of a bruised chest. The website looks very professional but the colours used could be improved as they may be problematic with some people with visual impairments and may not print well. The website gives a good overview of chest brusing, how it may be caused and how it can be managed. There are no distracting adverts. Generally is reads well, but some complex medical terms are used which make it more difficult to read.
36. Healthline	https://www.healthline.com/health/flail-chest	The Healthline website is organised in a visually appealing way with large text, easy-to-read font used and clear sub-headings. Adverts are present throughout the page, including pop-up windows, which are distracting and block the information from being read. No images are used, but bullet-points show key facts and highlighted

For peer review only

Name of Website	Website address	Website assessment
		phrases contain links to further information. Some language used is too advanced to be read by someone with no medical knowledge.
37. NHS Lanarkshire	https://www.nhslanarkshire.scot.nhs.uk/patient-information-leaflets/emergency-department/pil-chsinj-67661-l/	This website provides very brief information to patients who have presented with a chest injury to hospital. It includes the main signs and symptoms to consider and signposting should they need further advice. It is badged with the hospital name and NHS so adds credibility. There are no figures or photos but also no adverts. The language is straightforward and east to understand.
38. Cambridge University Hospitals NHS Foundation Trust	https://www.cuh.nhs.uk/patient-information/chest-injury-advice-sheet-for-patients/	This website is badged with the NHS and hospital logos which adds credibility. It provides very brief information about how patients should manage their chest injury after presenting to hospital. It mainly focuses on pain management and who to contact in an emergency. The language is very clear and simple. No images or adverts.
39. National University Hospital; National University Hospital Health System	https://www.nuh.com.sg/Health-Information/Diseases-Conditions/Pages/Chest-Injuries.aspx	The website is clean and uncluttered but the information contained within it is not really patient focused and uses lots of technical terms and diagrams where anatomy knowldege is assumed. There is limited helpful information for patients. There are no distracting adverts.
40. The Rotherham NHS Foundation Trust	https://www.therotherhamft.nhs.uk/patients-and-visitors/patient-information/chest-trauma-and-fractured-ribs#:~:text=If%20you%20do%20experience%20pain,please%20contact%20your%20GP%20practice.	This website is badged with the NHS and hospital logos which adds credibility. It provides comprehensive information about chest wall trauma and rib injuries and how patients should manage their injury after presenting to hospital. It is written in a very simple way and avoids using technical language. There are no figures to help illustrate the information but also no adverts.
41. Healthcare Associates of Texas	https://healthcareassociates.com/strained-chest-muscle-causes-symptoms-and-treatment/	The website feels rather cluttered as there is a menu on the right hand side and regular adverts interspersed with text to encourage users to 'book an appointment' which distracts from the information provided. There are no other adverts and some photos of people with possible chest injuries but no accompanying information. The information focuses of strained muscles, how you

Name of Website	Website address	Website assessment
		can get a muscle strain and treatment. The information is fairly clear and avoids the use of medical terminology.
42. Physio-pedia	https://www.physio-pedia.com/Sternal_fracture	Although this website does not contain external adverts, it feels rather cluttered as it advertises various physiotherapy courses before presenting the information on sternal fractures. There is a picture of the ribs, but no information to aid understanding. The information is fairly comprehensive but uses lots of medical terminology and long sentences which make understanding difficult.
43. Medical News Today	https://www.medicalnewstoday.com/articles/324534	Visually, the page is well laid-out, with good use of sub-headings, pictures and bullet-points to allow key information to be identified easily. The pictures are non-scientific, and work in conjunction with the text to demonstrate causes and treatments. Links at the top of the page allow easy movement between sections, which are concise and use space well. However, medical terminology is used throughout, and adverts and pop-ups may distract the reader.
44. My Family Physio	https://myfamilyphysio.com.au/common-upper-back-and-chest-injuries/#:~:text=Rib%20fractures%20and%20cartilage%20strains,pull%20starting%20a%20lawn%20mower).	The link to this website opens with a pop up message about still accepting appointments. This has to be closed before accessing the information which is distracting. The information provided is about common back and chest injuries so only parts of it are relevant. It includes some nice figures but limited explanation of them. The text contains a lot of medical terminology which makes it difficult to read.
45. Banner Health	https://www.bannerhealth.com/healthcareblog/better-me/what-to-know-about-strained-chest-muscles#:~:text=In%20most%20mild%20to%20moderate,area%20to%20help%20reduce%20pain.	This website focuses of the differences between chest injuries and a heart attack. It includes some photos but these do not really help to illustrate the text. There are no adverts. The article is largely clear but uses a lot of medical terminology.
46. Luna Physical Therapy	https://blog.getluna.com/pulled-chest-muscle-causes	This website provides a brief overview about pulled chest muscles, causes and treatment. The focus of the website is on physical therapy and the content largely reflects this. There are some photos but these do not aid the understanding of the content. The

For peer review only

Name of Website	Website address	Website assessment
		website is fairly clear and for the most part avoids the use of medical terminology.
47. Healthwise	https://www.cham.org/HealthwiseArticle.aspx?id=sig52119	This website is fairly bland with limited badging to identify the source, no figures, tables or photos. It does not have any adverts. Despite this it provides a brief summary about types of chest injuries as well as links to other conditions. The text avoids or explains medical terminology as far as possible which aids understanding.
48. Wirral Community Health and Care NHS Foundation Trust	https://www.wchc.nhs.uk/wp-content/uploads/2020/03/Chest-Injury_V1.pdf	This link takes you to a patient information leaflet titled 'chest injury'. It is very professional looking and is badged with the NHS and hospital logos, thus improving credibility. There is limited use of colour on a plain white background. There is a brief background to the injury as well as self-help guidance and how to get further help. No pictures or diagrams used. Medical terminology is avoided or explained and the language is very simple.
49. Barnsley Hospital NHS Foundation Trust	https://www.barnsleyhospital.nhs.uk/services/a-and-e/chest-injury	This website provides a very brief paragraph mainly to guide patients on its management but also to signpost for further help. It is badged with the hospital and NHS logos which improves credibility. The language is simple and clear. No figures or photos used. No adverts.
50. The Dudley Group NHS Foundation Trust	https://www.dgft.nhs.uk/wp-content/uploads/2023/09/Chest-injury-V3.pdf	This link takes you to a patient information sheet that describes chest injuries. It is badged with the hospital and NHS logos which improves credibility. It describes the causes, symptoms, how to manage it and where to go for further help. There are no figures or photos but the language is clear and limited medical terminology is used. There are no adverts.
51. York Hospitals	https://www.yorkhospitals.nhs.uk/seecmsfile/?id=6844	This link takes you to a patient information sheet that describes rib/chest injuries. It is badged with the hospital and NHS logos which improves credibility. It describes the causes, symptoms, how to manage it and where to go for further help. There are no figures or photos but the language is clear and limited medical terminology is used. There are no adverts.

For peer review only

Name of Website	Website address	Website assessment
52. Bradford Teaching Hospitals NHS Foundation Trust	https://digitalpatientinformationhub.bradfordhospitals.nhs.uk/leaflets/chest-injury/	This website provides very brief information mainly to guide patients on management of chest injuries following a visit to hospital. It also signposts for further help. It is badged with the hospital and NHS logos which improves credibility. The language is simple and clear. A simple illustration is used to illustrate a chest injury but otherwise there are no figures or photos used to help illustrate the text. No adverts.
53. Milton Keynes University Hospital	https://www.mkuh.nhs.uk/patient-information-leaflet/rib-or-chest-injury	This website provides a very brief paragraph mainly to guide patients on the management of rib or chest injuries but also to signpost for further help. It is badged with the hospital and NHS logos which improves credibility. The language is simple and clear. No figures or photos used. No adverts.
54. Dorset County Hospital	https://www.dchft.nhs.uk/wp-content/uploads/2020/10/ED-Chest-and-Rib-Injury-0414.pdf	This link takes you to a patient information sheet that describes how to manage rib/chest injuries onced discharged from hospital. It is badged with the hospital and NHS logos which improves credibility. It looks a little cluttered. There are no figures or photos but the language is clear and limited medical terminology is used. There are no adverts.
55. Mount Sinai	https://www.mountsinai.org/health-library/selfcare-instructions/rib-fracture-aftercar	This website provides information on rib fracture after care following presentation. It is nicely laid out and gives information about the injury, pain releif and what to expect. It also provides links to other conditions and references for further reading. The information is fairly straightforward although this is interspersed with medical terminology. There are no photos or illustrations and no external adverts although there is an 'internal advert' to book and appointment
56. Yale Medicine	https://www.yalemedicine.org/conditions/rib-fracture	This website provides an overview about rib fractures. The page itself looks rather cluttered as it has a menu on the left of the page. The colouring of some of the website may cause issues for those with sight impairments. The website itself looks fairly professional but the information is rather complex and includes lots of medical

For peer review only

Name of Website	Website address	Website assessment
		terminology. There is a photo included but this does not aid understanding of the text. No distracting adverts
57. Oxford Health NHS Foundation Trust	https://www.oxfordhealth.nhs.uk/wp-content/uploads/2014/08/OP-144.15-Rib-injury-advice.pdf	This link takes you to a patient information sheet that describes how to manage rib injuries. It is badged with the hospital and NHS logos which improves credibility. It is very brief but describes what a rib injury is and how it manage it. It also signposts contact information. There are no figures or photos but the language is clear and limited medical terminology is used. There are no adverts.
58. Brigham and Women's Hospital	https://www.brighamandwomens.org/lung-center/diseases-and-conditions/rib-fractures	This website looks rather cluttered as it immediately advertises appointments for patients which distracts from the main information about rib fractures. There is also a menu on the left of the page which is distracting. It includes a photograph at the top of the page but it is not relevant to rib fractures. The information itself is rather complex with lots of medical terminology. No other relevant illustrations are included but also no adverts.
59. topdoctors.co.uk	https://www.topdoctors.co.uk/medical-articles/rib-fractures-a-comprehensive-guide-for-patients	The website is rather cluttered with lots of pop-up messages before the information can be acccessed. It also includes information and prices to book an appointment which distracts from the information about rib fractures. There is a photo of the rib cage but this does not aid understanding of the text. It gives a reasonable summary about rib fractures but there is a lot of medical terminology used.
60. Ventura Orthopedics	https://venturaortho.com/difference-between-bruised-and-broken-ribs/	This website appears fairly professional initially. Further inspection however shows a ribbon of information including contact numbers, appointment booking and social media pages. There is a photo at the top of the page but it doesn't help to illustrate the content in any way. The information itself is fairly straightforward on the whole but does include some medical terminology. No adverts or other illustrations are included.
61. Nebraska Medicine	https://www.nebraskamed.com/trauma/help-broken-ribs-heal-faster	This website doesn't have dynamic adverts but includes bold information about booking an appointment which distracts from the information provided. It provides a brief overview about rib

For peer review only

Name of Website	Website address	Website assessment
		fractures which includes an x-ray photo of the ribcage. The photo does not aid in understanding the narrative provided. A lot of medical terminology and drug names are used which makes the content complex.
62. Dr Marco Scarci	https://www.marcoscarci.co.uk/what-are-the-long-term-effects-of-broken-ribs	The website looks cluttered with lots of photos and links to other articles to the right of the page. There are also interruptions to the text where links to make an appointment can be made. This distracts from the information provided. The text is quite small and there are no figures to aid understanding of the text. The information itself is a bit complex despite limited use of use of medical terminology. No adverts.
63. Doncaster and Bassetlaw Teaching Hospitals	https://www.dbth.nhs.uk/wp-content/uploads/2022/05/WPR48780-Rib-injury-leaflet.pdf	This link takes you to a patient information sheet that describes how to manage bruised or broken ribs. It provides a brief overview of do's and don'ts and where to get more advice. It also provides information about breathing exercises including photographs to illustrate. It is badged with the hospital and NHS logos which improves credibility. The language is clear and limited medical terminology is used. There are no adverts.
64. University Hospitals Coventry and Warwickshire	https://www.uhcw.nhs.uk/download/clientfiles/files/Patient%20Information%20Leaflets/Emergency%20Medicine/Emergency%20Department/118694_Emergency_Department_-_Bruised_or_broken_ribs_(1720)_-_March_2019.pdf	This link takes you to a patient information sheet that describes how to manage bruised or broken ribs following hospital discharge. It is badged with the hospital and NHS logos which improves credibility. It is very brief but describes what a rib injury is and how it manage it. It also signposts contact information. There are no figures or photos but the language is clear and limited medical terminology is used. There are no adverts.
65. Orthopaedics and Trauma London	http://www.orthopaedicsandtraumalondon.co.uk/conditions/rib-fractures/	This website looks fairly professional but gives the appearance of a sports website rather than a medical one, with photographs and sporting situations on the top of the page. It provides brief information about rib fractures, signs and symptoms, diagnosis and treatment. It provides a reasonable overview but the language is rather complex and there is a lot of medical terminology. No relevant illustrations and no adverts.

For peer review only

Name of Website	Website address	Website assessment
66. NCH Healthcare System	https://nchmd.org/health-library/diseases-and-conditions/con-20155268/	The top of this website is a bit cluttered and includes information about booking an appointment and paying bills etc. It also has a photo that is not related to the content on 'broken ribs' which appears lower down the page. The information contained is fairly well presented and includes an illustration of broken ribs. The page includes symptoms, causes, complications and treatment. The information is simple and avoids medical terminology. No distracting adverts.
67. Physiocheck	https://www.physiocheck.co.uk/condition/80/bruised-or-broken-rib	This website is very brightly coloured, but also feels very cluttered. There is a menu on the right hand side containing links to further information and how to make contact but this is quite distracting. It also includes active links for checking symptoms and asking a physio. There are a couple of illustrations of the ribs but these are difficult to understand for a lay person. The information provided on bruised or broken ribs is brief and includes a description, causes, signs and symptoms and treatment. It includes some medical terminology which makes comprehension more difficult but does try to explain these terms. No adverts.
68. Dr Gallagher and Partners	https://www.drgallagherandpartners.org.uk/website/M84035/files/rib%20inju.pdf	This link takes you to a patient information sheet that describes how to care for rib injuries. It looks very professional and describes the nature of the injuries, treatment and diagnosis. Some complex medical terminology is used but this is explained and the language is fairly simple. No illustrations to aid understanding but no adverts.
69. Agency for Clinical Innovation	https://aci.health.nsw.gov.au/networks/eci/clinical/ed-factsheets/rib-fracture	This website provides a fact sheet of information for people who have presented to the emergency department with rib fractures. Language is simple but a number of medical terms are used which may limit understanding. It provides a very brief overview of the condition and includes treatment and management. It also signposts to further contacts. It is clean and uncluttered and includes one illustration about breathing exercises. No adverts.

For peer review only

Name of Website	Website address	Website assessment
70. SportsMD.com	https://www.sportsmd.com/sports-injuries/chest-rib-injuries/rib-fracture-pneumothorax-complication/	The appearance of this website is generally ok, although the right of the page shows a large 'internal' advert promoting appointment bookings which is slightly distracting. There are a couple of illsurations that help with understanding of the text. The text is fairly comprehensive and includes types of injuries, complications, symptoms, causes and treatment. The page also includes a video. Some of the language is quite complex and includes a lot of medical terminology. No external adverts.
71. GP notebook	https://gpnotebook.com/simplepage.cfm?ID=-1120600051	This website opens with a number of pop-ups which are immediately distracting. Only limited information can be acccessed without subscription. The site is clearly labelled as a resource for healthcare professionals and the content reflects this as there is a lot of medical terminology and the language is very complex. The information provided is very brief. There are no illustrations. Some of the pages include dynamic adverts which are distracting.
72. Upstate University Hospital	https://www.upstate.edu/thoracic-surgery/conditions/rib-fracture.php	This website is clean and uncluttered and shows an image of the rib cage. It provides very brief information about rib fractures including causes, risk factors, symptoms and treatment. The information is very simple and brief and avoids medical terminology. There are no adverts to distract.
73. Fracture and Orthopaedic Clinic Ltd	https://www.faoconline.com/home/conditions/general/fracture-rib	This is a very professional looking website that provides information about fractured ribs including causes, symptoms and treatment. It is mainly black text on a white background and although the text size is adequate the text itself is quite light so may cause problems for people with sight issues. The language is a straightforward on the whole but includes medical terminology which may make comprehension difficult. There are no illustrations or adverts.
74. HealthyWA	https://www.healthywa.health.wa.gov.au/Articles/A_E/Chest-injuries-and-rib-fractures	This website is badged with the Department of Health Western Australia which adds credibility to the information. It provides very brief information about chest injuries and rib fractures and do's and don'ts but mainly signposts to further information if needed. There

For peer review only

Name of Website	Website address	Website assessment
		are no illustrations and no adverts. The language is very basic with limited used of medical terminology.
75. St James's Hospital	https://www.stjames.ie/media/Rib%20fracture%20Advice%20Leaflet.pdf	This link takes you to a patient information sheet that describes how to manage rib fractures. It is badged with the hospital logo which adds credibility to the information, however the quality of the logo is poor. It provides an overview, a list of do's and don'ts, but is primarily focused on signposting. The language is simple and clear with limited use of medical terms. There are no illustrations and no adverts.
76. International Centre for Thoracic Surgery	https://www.icts.com.sg/rib-fracture-fixation-from-trauma/	This website is a little overwhelming initially due its use of strong colours at the top of the page. The context itself is well laid out and comprehensive and includes some nice illustrations and photos that help to reinforce the messages in the text. There are no distracting adverts but the text is quite complex and includes a lot of medical terminology.
77. ISK Institute	https://www.iskinstitute.com/kc/abdomen/rib_fractures/rib_fractures.html	This website is very text heavy and not particularly appealing. The font size and colouring make reading the content difficult. There are error messages indicating some of the content is not supported. There are no images to help illustrate the text and no adverts. The language is very complex and includes a lot of medical terms.
78. University Hospitals Plymouth NHS Trust	https://www.plymouthhospitals.nhs.uk/display-pil/pil-chest-wall-injury-6063/	This website is badged with the hospital and NHS logos which adds credibility to the information. The website provides a brief overview of chest wall injuries and how they are managed. There is also signposting information to make further contacts if required. There are no illustrations and no adverts. The language is clear and simple with limited medical terminology used.
79. Masnad Health Clinic	https://www.masnad.com.au/knowledge-centre/injury-hub/middle-back/rib-cartilage-injury/	There are a number of pop-ups on this page advertising for appointments and to leave reviews, which are immediately distracting. There are some illustrations, including one of the rib cage and some photos illustrating breathing and stretching exercises which are useful. The information focuses on rib cartilages injuries.

For peer review only

Name of Website	Website address	Website assessment
		The information is quite comprehensive but the language is sometimes complex and includes lot of medical terminology.
80. Royal Berkshire Hospital	https://www.royalberkshire.nhs.uk/media/jfcjgxef/chest-wall-injury_dec21.pdf	This weblinks takes you to a patient information leaflet about chest wall injury. It provides information to patients who have been discharged. It is badged with the hospital and NHS logos which adds credibility to the information. The information provides a brief overview of chest wall injuries and how they are managed. There is also signposting information to make further contacts if required. There are no illustrations and no adverts. The information is simple and avoid medical terminology.
81. Chest Wall Injury Society	https://cwisociety.org/patientportal/	This website is a patient portal for those who have had a chest wall injury. There is an 'open area' that provides information about considerations before having treatment. The focus is very patient focused and the majority of the information is provided in the form of individual patient video stories. There is also a member only area. There are no adverts or other illustrations. Despite being a patient portal, the text information provided is limited and although limited medical terminology is used the language is quite complex.
82. UMRC Rochester	https://www.urmc.rochester.edu/encyclopedia/content.aspx?ContentTypeID=22&ContentID=FlailChest	This website is linked to a US Medical Center, which adds credibility to the information provided about 'flail chest'. The webpage is clean and uncluttered but does not include any illustrations. The information provided is very complex and includes lots of medical terminology. No adverts.
83. Tufts Medical Center Community Care	https://hhma.org/healthadvisor/aha-ribinjur-sma/	This website provides brief information about rib injuries, symptoms and treatment. The webpage is largely clear an uncluttered but the image is not available. The language is simple and avoids the use of medical terminology. There are no distracting adverts.
84. Farrell Physiotherapy	https://farrellphysiotherapy.co.uk/conditions-treated/thoracic-spine/rib-fractures/	The top half of this website is mostly black with an underlying x-ray image, but it is very difficult to see. The information about rib fractures begins about half way down the page and a brief overview is provided on the causes and symptoms. Most of the information

For peer review only

Name of Website	Website address	Website assessment
		is focused on physiotherapy treatments provided by the clinic to help manage rib fractures. There is an image of a back showing the rib cage within, but the image is not helpful in supportiong the text content. The orientation of the text and the use of capitals makes navigation of the website difficult. The text itself is fairly simple and medical terminology use is limited. No adverts.
85. healthinfo.org.nz	https://www.healthinfo.org.nz/patientinfo/439381.pdf	The link takes you to a patient information leaflet about rib injuries. The leaflet is badged with health board logos which adds credibility to the information. It provides a brief overview about symptoms and self care and where to go for more help. The information is clear and simple and avoids the use of medical terminology. There is a single photo of a fallen skiier. There are no adverts.

For peer review only

Supplementary information. Table S3. Readability assessment of the 85 websites identified on Google™, Yahoo™ and Bing™.

**Readability Assessments**	**Website**														
	1. Medscape			2. UptoDate			3. Health Direct			4. Dynamed			5. Wikipedia		
	Grade (US)	Grade (UK)	Age	Grade (US)	Grade (UK)	Age	Grade (US)	Grade (UK)	Age	Grade (US)	Grade (UK)	Age	Grade (US)	Grade (UK)	Age
Flesch-Kincaid	13.4	14.4	17-18	14.7	15.7	18+	6.5	7.5	11-12	11.2	12.2	18+	11.9	12.9	16-17
Gunning Fog	16.6	17.6	18+	17.4	18.4	18+	9.9	10.9	14-15	10.9	11.9	18+	13.6	14.6	18+
Coleman-Liau	15.1	16.1	18+	15.8	16.8	18+	8.8	9.8	13-14	13.9	14.8	18+	15.2	16.2	18+
SMOG	15.6	16.6	18+	16.4	17.4	18+	10.3	11.3	15-16	10.7	11.7	18+	11.9	12.9	16-17
Automated Readability	12.5	13.5	17-18	14.4	15.4	18+3	6.1	7.1	11-12	9.4	10.4	18+	10.3	11.3	15-16
Median readability grade	15.1	16.1	17-18	15.8	16.8	18+	8.8	9.8	13-14	10.9	11.9	18+	11.9	12.9	16-17
Flesch Reading Ease	22.5	Very difficult		21.1	Very difficult		68.6	Plain English		24.4	Very difficult		21.8	Very Difficult	
% of general public readable to	59%			50%			100%			76%			71%		
**Readability Assessments**	**Website**														
	6. North Bristol NHS Trust			7. Drugs.com			8. Gloucestershire Hospitals NHS Trust			9. Very well health			10. MSD Manual consumer version		
	Grade (US)	Grade (UK)	Age	Grade (US)	Grade (UK)	Age	Grade (US)	Grade (UK)	Age	Grade (US)	Grade (UK)	Age	Grade (US)	Grade (UK)	Age
Flesch-Kincaid	6.7	7.7	12-13	5.6	6.6	11-12	4.6	5.6	10-11	4.8	5.8	10-11	8.7	9.7	14-15
Gunning Fog	9.0	10.0	14-15	8.1	8.2	13-14	6.4	7.4	11-12	6.4	7.4	11-12	10.8	11.8	16-17
Coleman-Liau	9.6	10.6	15-16	9.0	10.0	14-15	8.1	9.1	13-14	7.4	8.4	13-14	11.5	12.5	17-18
SMOG	10.0	11.0	15-16	9.3	10.3	14-15	7.9	8.9	13-14	8.4	9.4	13-14	8.0	9.0	13-14
Automated Readability	6.5	7.5	11-12	5.5	6.5	10-11	4.5	5.5	9-10	4.6	5.6	9-10	11.2	12.2	16-17
Median readability grade	9.0	10.0	14-15	8.1	9.1	13-14	6.4	7.4	11-12	6.4	7.4	11-12	10.8	11.8	16-17
Flesch Reading Ease	65.7	Plain English		69.4	Easy		71.3	Fairly easy		79.37	Fairly easy		51.4	Fairly difficult	
% of general public readable to	100%			100%			100%			100%			95%		
**Readability Assessments**	**Website**														
	11. The American Association for the Surgery of Trauma			12. LITFL			13. Radiology Key			14. NHS			15. Oxford University Hospitals NHS Trust		
	Grade (US)	Grade (UK)	Age	Grade (US)	Grade (UK)	Age	Grade (US)	Grade (UK)	Age	Grade (US)	Grade (UK)	Age	Grade (US)	Grade (UK)	Age
Flesch-Kincaid	12.5	13.5	17-18	12.0	13.0	17-18	12.6	13.6	17-18	4.5	4.5	10-11	6.7	7.7	12-13

For peer review only

Gunning Fog	16.0	17.0	18+	13.1	14.1	18+	15.4	16.4	18+	7.2	8.2	12-13	9.2	10.2	14-15
Coleman-Liau	14.7	15.7	18+	15.3	16.3	18+	14.1	15.1	18+	6.9	7.9	12-13	9.0	10.0	14-15
SMOG	14.5	15.5	18+	12.3	13.3	18+	14.4	15.4	18+	8.2	9.21	13-14	10.0	11.0	15-16
Automated Readability	11.4	12.4	16-17	10.5	11.5	15-16	11.9	12.9	17-18	4.2	5.2	9-10	7.0	8.0	12-13
Median readability grade	14.5	15.5	18+	12.3	13.3	18+	14.1	12.8	18+	6.9	7.9	12-13	9.0	10.0	14-15
Flesch Reading Ease	28.8	Difficult		24.1	Very difficult		32.6	Difficult		81.09	Easy		71.0	Fairly easy	
% of general public readable to	66%			70%			66%			100%			100%		
**Readability Assessments**	Websites														
	16. NICE			17. Aneurin Bevan University Health Board			18. My Health Alberta			19.Salisbury NHS Foundation Trust			20. Web MD		
	Grade (US)	Grade (UK)	Age	Grade (US)	Grade (UK)	Age	Grade (US)	Grade (UK)	Age	Grade (US)	Grade (UK)	Age	Grade (US)	Grade (UK)	Age
Flesch-Kincaid	7.5	8.5	12-13	6.8	7.8	12-13	4.7	5.7	12-13	7.2	8.2	12-13	7.5	8.5	13-14
Gunning Fog	10.7	11.7	16-17	9.2	10.2	14-15	7.1	8.2	14-15	9.2	10.2	14-15	7.7	8.7	13-14
Coleman-Liau	8.6	7.6	14-15	8.9	9.9	14-15	6.9	7.9	14-15	9.7	10.7	15-16	11.2	10.2	16-17
SMOG	10.9	11.9	16-17	10.0	11.0	15-16	8.3	9.3	15-16	10.3	11.3	15-16	10.1	11.1	15-16
Automated Readability	7.8	8.8	13-14	7.0	8.0	12-13	4.5	5.5	12-13	7.1	8.1	12-13	7.1	8.1	12-13
Median readability grade	8.6	9.6	14-15	8.9	9.9	14-15	6.9	7.9	14-15	9.2	10.2	14-15	7.7	8.7	13-14
Flesch Reading Ease	70.9	Fairly easy		71.0	Fairly easy		81.7	Easy		65.5	Plain English		54.9	Fairly difficult	
% of general public readable to	100%			100%			100%			100%			100%		
**Readability Assessments**	Websites														
	21. After Trauma			22. Royal Devon University Healthcare			23.South Tees Hospitals NHS Foundation Trust			24. Sirona Health and care			25. eMedicinehealth		
	Grade (US)	Grade (UK)	Age	Grade (US)	Grade (UK)	Age	Grade (US)	Grade (UK)	Age	Grade (US)	Grade (UK)	Age	Grade (US)	Grade (UK)	Age
Flesch-Kincaid	13.3	14.3	18+	5.0	6.0	10-11	7.3	8.3	12-13	4.7	5.7	10-11	7.1	8.1	12-13
Gunning Fog	15.4	16.4	18+	7.4	8.4	12-13	9.1	10.1	14-15	6.6	7.6	12-13	8.3	9.3	13-14
Coleman-Liau	12.4	13.4	18+	8.3	9.3	13-14	11.2	12.2	16-17	6.5	7.5	12-13	9.0	10.0	14-15
SMOG	14.1	15.1	18+	8.6	9.6	14-15	10.4	11.4	15-16	7.9	8.9	13-14	10.7	11.7	16-17
Automated Readability	11.1	12.1	18+	7.8	8.8	13-14	7.8	8.8	13-14	3.6	4.6	9-10	5.7	6.7	11-12
Median readability grade	13.3	14.3	18+	7.8	8.8	13-14	9.1	10.1	14-15	6.5	7.5	12-13	8.3	9.3	13-14
Flesch Reading Ease	42.0	Difficult		73.4	Fairly easy		61.6	Plain English		71.0	Fairly easy		61.6	Plain English	
% of general public readable to	100%			100%			100%			100%			100%		

For peer review only

**Readability Assessments**	**Website**														
	26. Better Health Channel			27. Rib Injury Clinic			28. Hull University Teaching Hospitals NHS Trust			29. Mount Nittany Health			30. MedlinePlus		
	Grade (US)	Grade (UK)	Age	Grade (US)	Grade (UK)	Age	Grade (US)	Grade (UK)	Age	Grade (US)	Grade (UK)	Age	Grade (US)	Grade (UK)	Age
Flesch-Kincaid	6.9	7.9	12-13	10.7	11.7	16-17	9.7	10.7	15-16	5.2	6.2	10-11	7.0	8.0	12-13
Gunning Fog	9.2	10.2	13-14	12.4	13.4	18+	11.3	12.3	16-17	7.2	8.2	12-13	7.1	8.1	12-13
Coleman-Liau	9.9	10.9	14-15	12.2	13.2	18+	11.4	12.4	16-17	7.8	8.8	13-14	10.7	11.7	16-17
SMOG	10.2	11.2	16-17	15.5	16.5	18+	12.6	13.6	18+	8.7	9.7	14-15	9.8	10.8	15-16
Automated Readability	6.6	7.6	11-12	10.4	11.4	13-17	10.0	11.0	15-16	4.7	5.7	10-11	6.7	7.7	12-13
Median readability grade	9.2	10.2	13-14	12.2	13.2	18+	11.3	12.3	16-17	7.2	8.2	12-13	7.1	8.1	12-13
Flesch Reading Ease	64.3	Plain English		47.0	Difficult		54.3	Fairly difficult		74.5	Fairly easy		55.9	Fairly difficult	
% of general public readable to	100%			80%			88%			100%%			100%		

**Readability Assessments**	**Website**														
	31. European Society of Thoracic Surgeons			32. Cleveland Clinic			33. mactheknife			34. World Rugby Passport			35. Total Vitality Medical Group		
	Grade (US)	Grade (UK)	Age	Grade (US)	Grade (UK)	Age	Grade (US)	Grade (UK)	Age	Grade (US)	Grade (UK)	Age	Grade (US)	Grade (UK)	Age
Flesch-Kincaid	11.0	12.0	16-17	6.3	7.3	11-12	12.7	13.7	18+	6.2	7.2	11-12	10.3	11.3	15-16
Gunning Fog	13.4	14.4	18+	8.6	9.6	14-15	16.0	17.0	18+	10.0	11.0	15-16	12.4	13.4	18+
Coleman-Liau	12.8	13.8	18+	9.6	10.6	15-16	13.4	14.4	18+	7.6	8.6	13-14	12.2	13.2	18+
SMOG	12.8	13.8	18+	9.8	10.8	15-16	15.0	16.0	18+	10.4	11.4	15-16	13.0	14.0	18+
Automated Readability	11.0	12.0	16-17	6.1	7.1	11-12	12.1	13.1	18+	5.9	6.9	11-12	10.9	11.9	16-17
Median readability grade	12.5	13.5	18+	8.6	9.6	14-15	13.4	14.4	18+	7.6	8.6	13-14	12.2	13.2	18+
Flesch Reading Ease	37.4	Difficult		66.4	Plain English		34.8	Difficult		75.5	Fairly easy		50.3	Fairly difficult	
% of general public readable to	78%			100%			65%			100%			83%		

**Readability Assessments**	**Website**														
	36. Healthline			37. NHS Lanarkshire			38. Cambridge University Hospitals NHS Foundation Trust			39. National University Hospital; National University Hospital System			40. The Rotherham NHS Foundation Trust		
	Grade (US)	Grade (UK)	Age	Grade (US)	Grade (UK)	Age	Grade (US)	Grade (UK)	Age	Grade (US)	Grade (UK)	Age	Grade (US)	Grade (UK)	Age
Flesch-Kincaid	8.6	9.6	14-15	6.0	7.0	11-12	6.9	7.9	12-13	8.1	9.1	13-14	8.1	9.1	13-14

Gunning Fog	11.4	12.4	16-17	7.8	8.8	13-14	10.1	11.2	15-16	9.1	10.1	14-15	10.5	11.5	16-17
Coleman-Liau	10.2	11.2	15-16	8.7	9.7	14-15	7.9	8.9	13-14	11.7	12.7	17-18	9.9	10.9	15-16
SMOG	11.7	12.7	17-18	9.7	10.7	15-16	10.4	11.4	15-16	11.1	12.1	16-17	11.1	12.1	16-17
Automated Readability	8.3	9.3	13-14	5.1	6.1	10-11	6.6	7.6	12-13	8.4	9.4	13-14	8.1	9.1	13-14
Median readability grade	10.2	11.2	15-16	7.8	8.8	13-14	7.9	8.9	13-14	9.1	10.1	14-15	9.9	10.9	15-16
Flesch Reading Ease	59.5	Fairly difficult		65.7	Plain English		71.7	Plain English		57.4	Fairly difficult		61.7	Plain English	
% of general public readable to	95%			100%			100%			99%			99%		
**Readability Assessments**							**Website**								
	41. Healthcare Associates of Texas			42. Physio-pedia			43. Medical News Today			44. My Family Physio			45. Banner Health		
	Grade (US)	Grade (UK)	Age	Grade (US)	Grade (UK)	Age	Grade (US)	Grade (UK)	Age	Grade (US)	Grade (UK)	Age	Grade (US)	Grade (UK)	Age
Flesch-Kincaid	7.8	8.8	13-14	11.9	12.9	17-18	7.4	8.4	12-13	10.3	11.3	15-16	8.1	9.1	13-14
Gunning Fog	9.2	10.2	14-15	13.4	14.4	18+	9.2	10.2	14-15	12.1	13.1	18+	10.2	11.2	15-16
Coleman-Liau	11.2	12.2	16-17	15.3	16.3	18+	10.3	11.3	15-16	13.0	14.0	18+	10.1	11.1	15.-16
SMOG	10.3	11.3	15-16	13.2	14.2	18+	10.1	11.1	15-16	12.7	13.7	18+	10.6	11.6	16-17
Automated Readability	8.0	9.0	13-14	11.9	12.9	17-18	6.8	7.8	12-13	10.2	11.2	15-16	8.5	9.5	14-15
Median readability grade	9.2	10.2	14-15	13.2	14.2		9.2	10.2	14-15	12.1	13.1	18+	10.1	11.1	15-16
Flesch Reading Ease	59.1			33.3	Difficult		59.0	Fairly difficult		45.6	Difficult		65.3	Plain English	
% of general public readable to	100%	Fairly difficult		71%			100%			83%			99%		
**Readability Assessments**							**Website**								
	46. Luna Physical Therapy			47. Healthwise			48. Wirral Community Health and Care NHS Foundation Trust			49. Barnsley Hospital NHS Foundation Trust			50. The Dudley Group NHS Foundation Trust		
	Grade (US)	Grade (UK)	Age	Grade (US)	Grade (UK)	Age	Grade (US)	Grade (UK)	Age	Grade (US)	Grade (UK)	Age	Grade (US)	Grade (UK)	Age
Flesch-Kincaid	8.2	9.2	13-14	5.9	6.9	11-12	5.2	6.2	10-11	5.0	6.0	10-11	4.8	5.8	10-11
Gunning Fog	10.7	11.7	16-17	8.2	9.2	13-14	7.4	8.4	12-13	7.4	8.4	12-13	6.5	7.5	11-12
Coleman-Liau	10.5	11.5	15-16	6.6	7.6	12-13	8.1	9.1	13-14	8.3	9.3	13-14	7.0	8.0	12-13
SMOG	11.0	12.0	16-17	8.9	9.9	14-15	9.0	10.0	14-15	8.8	9.8	14-15	8.3	9.3	13-14
Automated Readability	8.5	9.5	13-14	4.9	5.9	10-11	4.7	5.7	10-11	5.6	6.6	11-12	3.7	4.7	9-10
Median readability grade	10.5	11.5	15-16	6.6	7.6	12-13	7.4	8.4	12-13	7.4	8.4	12-13	6.5	7.5	11-12
Flesch Reading Ease	61.8	Plain English		76.9	Plain English		72.5	Fairly easy		77.7	Fairly easy		72.4	Plain English	

For peer review only

% of general public readable to	98%			100%			100%			100%			100%		
**Readability Assessments**	**Website**														
	51. York Hospitals			52. Bradford Teaching Hospitals NHS Foundation Trust			53. Milton Keynes University Hospital			54. Dorset County Hospital			55. Mount Sinai		
	Grade (US)	Grade (UK)	Age	Grade (US)	Grade (UK)	Age	Grade (US)	Grade (UK)	Age	Grade (US)	Grade (UK)	Age	Grade (US)	Grade (UK)	Age
Flesch-Kincaid	4.5	5.5	10-11	4.5	5.5	10-11	5.8	6.8	11-12	3.9	4.9	9-10	7.2	8.2	12-13
Gunning Fog	7.4	8.4	12-13	6.1	7.1	11-12	8.6	9.6	14-15	6.2	7.2	11-12	9.6	10.6	15-16
Coleman-Liau	7.8	8.8	13-14	7.0	8.0	12-13	8.3	9.3	13-14	6.1	7.1	11-12	9.6	10.6	15-16
SMOG	8.5	9.5	13-14	8.3	9.3	13-14	9.8	10.8	15-16	7.8	8.8	13-14	10.3	11.3	15-16
Automated Readability	4.6	5.6	10-11	4.1	5.1	9-10	5.0	6.0	10-11	3.0	4.0	8.9	6.9	7.9	12-13
Median readability grade	7.4	8.4	12-13	6.1	7.1	11-12	8.3	9.3	13-14	6.1	7.1	11-12	9.6	10.6	15-16
Flesch Reading Ease	78.8	Fairly easy		79.2	Fairly easy		68.9	Plain English		78.9	Fairly easy		65.2	Plain English	
% of general public readable to	100%			100%			100%			100%			100%		
**Readability Assessments**	**Website**														
	56. Yale Medicine			57. Oxford Health NHS Foundation Trust			58. Brigham and Women's Hospital			59. topdoctors.co.uk			60. Ventura Orthopedics		
	Grade (US)	Grade (UK)	Age	Grade (US)	Grade (UK)	Age	Grade (US)	Grade (UK)	Age	Grade (US)	Grade (UK)	Age	Grade (US)	Grade (UK)	Age
Flesch-Kincaid	7.6	8.6	13-14	4.5	5.5	10-11	9.8	10.8	15-15	9.0	10.0	14-15	7.0	8.0	12-13
Gunning Fog	9.3	10.3	14-15	6.8	7.8	12-13	12.9	13.9	18+	11.6	12.6	17-18	9.3	10.3	14-15
Coleman-Liau	9.9	10.9	15-16	5.7	6.7	11-12	12.3	13.3	18+	10.1	11.1	15-16	9.3	10.3	14-15
SMOG	10.3	11.3	15-16	7.8	8.8	13-14	12.6	13.6	18+	11.6	12.6	17-18	10.0	11.0	15-15
Automated Readability	7.5	8.5	13-14	2.8	3.8	8-9	9.3	10.3	14-15	8.9	9.9	14-15	7.0	8.0	12-13
Median readability grade	8.5	9.5	14-15	5.7	6.7	11-12	12.3	13.3	18+	10.1	11.1	15-16	9.3	10.3	14-15
Flesch Reading Ease	60.7	Plain English		72.6	Fairly easy		47.5	Difficult		59.2	Fairly difficult		68.2	Plain English	
% of general public readable to	95%			100%			86%			92%			100%		
**Readability Assessments**	**Website**														
	61. Nebraska Medicine			62. Dr Marco Scarci			63. Doncaster and Bassetlaw Teaching Hospitals			64. University Hospitals Coventry and Warwickshire			65. Orthopaedics and Trauma London		
	Grade (US)	Grade (UK)	Age	Grade (US)	Grade (UK)	Age	Grade (US)	Grade (UK)	Age	Grade (US)	Grade (UK)	Age	Grade (US)	Grade (UK)	Age

For peer review only

Flesch-Kincaid	8.9	9.9	14-15	9.9	10.9	15-15	3.1	4.1	8-9	4.9	5.9	10-11	10.2	11.2	15-16
Gunning Fog	11.2	12.2	16-17	12.3	13.3	18+	4.9	5.9	10-11	7.0	8.0	12-13	13.1	14.1	18+
Coleman-Liau	10.6	11.6	16-17	11.1	12.1	16-17	5.5	6.5	10-11	8.4	9.4	13-14	12.2	13.2	18+
SMOG	11.5	12.5	17-18	12.5	13.5	18+	6.9	7.9	12-13	9.0	10.0	14-15	12.8	13.8	18+
Automated Readability	9.0	10.0	14-15	9.9	10.9	15-16	2.5	3.5	8-9	5.1	6.1	10-11	10.1	11.1	15-15
Median readability grade	10.6	11.6	16-17	11.1	12.1	16-17	4.9	5.9	10-11	7.0	8.0	12-13	12.2	13.2	18+
Flesch Reading Ease	58.9	Fairly difficult		53.5	Fairly difficult		85.3	Easy		73.2	Fairly easy		48.9	Difficult	
% of general public readable to	93%			93%			100%			100%			84%		
**Readability Assessments**	**Website**														
	66. NCH Healthcare System			67. Physiocheck			68. Dr Gallagher and Partners			69. Agency for Clinical Innovation			70. SportsMD.com		
	Grade (US)	Grade (UK)	Age	Grade (US)	Grade (UK)	Age	Grade (US)	Grade (UK)	Age	Grade (US)	Grade (UK)	Age	Grade (US)	Grade (UK)	Age
Flesch-Kincaid	4.9	5.9	10-11	6.2	7.2	11-12	6.5	7.5	11-12	7.6	8.6	13-14	9.3	10.3	14-15
Gunning Fog	7.2	8.2	12-13	8.5	9.5	14-15	9.0	10.0	14-15	9.9	10.9	15-16	11.6	12.6	17-18
Coleman-Liau	7.2	8.2	12-13	7.7	8.7	13-14	9.2	10.2	14-15	11.2	12.2	16-17	11.8	12.8	17-18
SMOG	8.6	9.6	14-15	9.7	10.7	15-16	9.6	10.6	15-16	10.4	11.4	15-16	12.0	13.0	17-18
Automated Readability	4.3	5.3	9-10	5.1	6.1	10-11	5.6	6.6	11-12	7.5	8.5	13-14	9.6	10.6	15-16
Median readability grade	7.2	8.2	12-13	7.7	8.7	13-14	9.0	10.0	14-15	9.9	10.9	15-16	11.6	12.6	17-18
Flesch Reading Ease	76.8	Fairly easy		70.1	Fairly easy		63.9	Plain English		57.6	Fairly difficult		54.5	Fairly difficult	
% of general public readable to	100%			100%			100%			100%			90%		
**Readability Assessments**	**Website**														
	71. GP notebook			72. Upstate University Hospital			73. Fracture and Orthopedic Clinic			74. HealthyWA			75. St James's Hospital		
	Grade (US)	Grade (UK)	Age	Grade (US)	Grade (UK)	Age	Grade (US)	Grade (UK)	Age	Grade (US)	Grade (UK)	Age	Grade (US)	Grade (UK)	Age
Flesch-Kincaid	7.8	8.8	13-14	4.4	5.4	9-10	4.4	5.4	9-10	10.2	11.2	15-16	4.0	5.0	9-10
Gunning Fog	9.3	10.3	15-16	5.2	6.2	10-11	5.2	6.2	10-11	13.1	14.1	18+	6.6	7.6	12-13
Coleman-Liau	9.5	10.5	15-16	7.1	8.1	12-13	7.1	8.1	12-13	12.2	13.2	18+	7.3	8.3	12-13
SMOG	10.1	11.1	15-16	7.7	8.7	13-14	7.7	8.7	13-14	12.8	13.8	18+	8.0	9.0	13-14
Automated Readability	6.9	7.9	12-13	3.9	4.9	9-10	3.7	4.7	9-10	10.1	11.1	15-16	4.0	5.0	9-10
Median readability grade	9.3	10.3	15-16	5.2	6.2	10-11	5.2	6.2	10-11	12.2	13.2	18+	6.6	7.6	12-13
Flesch Reading Ease	59.3	Fairly difficult		74.5	Fairly easy		74.5	Fairly easy		48.9	Difficult		79.3	Fairly easy	

For peer review only

% of general public readable to	96%			100%			100%			84%			100%		
**Readability Assessments**	**Website**														
	76. International Centre for Thoracic Surgery			77. ISK Institute			78. University Hospitals Plymouth NHS Trust			79. Masnad Health Clinic			80. Royal Berkshire Hospital		
	Grade (US)	Grade (UK)	Age	Grade (US)	Grade (UK)	Age	Grade (US)	Grade (UK)	Age	Grade (US)	Grade (UK)	Age	Grade (US)	Grade (UK)	Age
Flesch-Kincaid	7.8	8.8	13-14	9.1	10.1	14-15	6.7	7.7	12-13	8.2	9.2	13-14	5.2	6.2	10-11
Gunning Fog	9.7	10.7	15-16	11.4	12.4	16-17	8.9	9.9	14-15	9.8	10.8	15-16	7.8	8.8	13-14
Coleman-Liau	10.4	11.4	15-16	10.7	11.7	16-17	8.5	9.5	13-14	10.1	11.1	15-16	8.3	9.3	13-14
SMOG	10.6	11.6	16-17	11.7	12.7	17-18	9.9	10.9	14-15	11.5	12.5	17-18	8.8	9.8	14-15
Automated Readability	7.7	8.7	13-14	9.7	10.7	15-16	5.7	6.7	11-12	7.9	8.9	13-14	5.0	6.0	10-11
Median readability grade	9.7	10.7	15-16	10.7	11.7	16-17	8.5	9.5	13-14	9.8	10.8	15-16	7.8	8.8	13-14
Flesch Reading Ease	61.00	Plain English		59.8	Fairly difficult		67.2	Plain English		60.8	Plain English		73.5	Fairly easy	
% of general public readable to	100%			92%			99%			99%					
**Readability Assessments**	**Website**														
	81. Chest Wall Injury Society			82. UMRC Rochester			83. Tufts Medical Center Community Care			84. Farrell Physiotherapy			85. healthinfo.org.nz		
	Grade (US)	Grade (UK)	Age	Grade (US)	Grade (UK)	Age	Grade (US)	Grade (UK)	Age	Grade (US)	Grade (UK)	Age	Grade (US)	Grade (UK)	Age
Flesch-Kincaid	8.9	9.9	14-15	9.8	10.8	15-16	6.2	7.2	11-12	7.6	8.6	13-14	4.7	5.7	10-11
Gunning Fog	10.7	11.7	16-17	12.7	13.7	18+	9.3	10.3	14-15	8.8	9.8	14-15	7.7	8.7	13-14
Coleman-Liau	13.6	14.6	18+	11.5	12.5	16-17	8.3	9.3	13-14	10.1	11.1	15-16	6.9	7.9	12-13
SMOG	12.3	13.3	18+	12.5	13.5	18+	10.0	11.0	15-16	9.9	10.9	15-16	8.6	9.6	14-15
Automated Readability	10.4	11.4	15-16	9.9	10.9	15-16	5.5	6.5	11-12	7.2	8.2	12-13	3.7	4.7	9-10
Median readability grade	10.7	11.7	16-17	11.5	12.5	16-17	8.3	9.3	13-14	8.8	9.8	14-15	6.9	7.9	12-13
Flesch Reading Ease	51.1	Fairly difficult		53.2	Fairly difficult		69.9	Plain English		61.8	Plain English		74.0	Fairly easy	
% of general public readable to	93%			87%			100%			100%			100%		

For peer review only

For peer review only - http://bmjopen.bmj.com/site/about/guidelines.xhtml
